# Supplementary material for: Pesticide residues alter taxonomic and functional biodiversity in soils
Source: Nature. 2026 Jan 28;650(8101):367–73. doi: 10.1038/s41586-025-09991-z (PMC12965876; doi:10.1038/s41586-025-09991-z)
Supplement: Supplementary file 1 — This file contains Supplementary Table 1, Supplementary Results 1–4, Supplementary Figs. 1–5, Supplementary Discussion, additional information about Supplementary Data 1–8 and references. [file 41586_2025_9991_MOESM1_ESM.docx]

**Supplementary Information**

**Supplementary Table S1. Pesticide data.** Information on the type, limit of quantification (LOQ, in mg/kg), analytical method (AM) used and regulatory information for the 63 pesticides detected. Analytical method: 1=LC-MS/MS, 2=GC-MS/MS, 3=Glyphosate/AMPA, m=metabolite

| **Pesticide** | **Type^93^** | **Chemical group^94,95,96^** | **Mode of action^94,95,96^** | **LOQ ^97^** | **AM ^97^** | **Regulation status in 2018^98^** | **Regulation status in 2024^99^** | **Year of ban^99^** |
| --- | --- | --- | --- | --- | --- | --- | --- | --- |
| AMPA | Herbicide (m) | Glycine | - | 0.010 | 3 | - | - | - |
| Atrazine | Herbicide | Triazines | Inhibition of Photosynthesis at PSll - Serine 264 Binders | 0.001 | 1 | Not approved | Not approved | 10/03/2004 |
| Azoxystrobin | Fungicide | Methoxy-acrylates | Respiration | 0.001 | 1 | Approved | Approved | - |
| Bixafen | Fungicide | Pyrazole-4-carboxamides | Respiration | 0.001 | 1 | Approved | Approved | - |
| Boscalid | Fungicide | Pyridine-carboxamides | Respiration | 0.001 | 1 | Approved | Approved | - |
| Bromuconazole | Fungicide | Triazoles | Sterol biosynthesis in membranes | 0.005 | 1 | Approved | Approved | - |
| Carbendazim | Fungicide | Benzimidazoles | Cytoskeleton and motor protein | 0.001 | 1 | Not approved | Not approved | 30/11/2014 |
| Chloridazon | Herbicide | Pyridazinone | Inhibition of Photosynthesis at PSll - Serine 264 Binders | 0.001 | 1 | Not approved | Not approved | 31/12/2018 |
| Chlorpyrifos | Insecticide | Organophosphate | Acetylcholinesterase (AChE) inhibitors | 0.001 | 1 | Approved | Not approved | 16/01/2020 |
| Clothianidin | Insecticide | Neonicotinoide | Nicotinic acetylcholine receptor (nAChR) competitive modulators | 0.001 | 1 | Not approved | Not approved | 31/01/2019 |
| Cyproconazole | Fungicide | Triazoles | Sterol biosynthesis in membranes | 0.005 | 1 | Approved | Not approved | 31/05/2021 |
| Cyprodinil | Fungicide | Anilino-pyrimidine | Amino acids and protein synthesis | 0.001 | 1 | Approved | Approved | - |
| Diazinon | Insecticide | Organophosphate | Acetylcholinesterase (AChE) inhibitors | 0.001 | 1 | Not approved | Not approved | 29/09/2006 |
| Dieldrin | Insecticide | Organochlorines | GABA-gated chloride channel blockers | 0.005 | 2 | Not approved | Not approved | 1981 (79/117/EEC) |
| Difenoconazole | Fungicide | Triazoles | Sterol biosynthesis in membranes | 0.001 | 1 | Approved | Approved | - |
| Diflufenican | Herbicide | Phenylethers | Inhibition of Phytoene Desaturase | 0.001 | 1 | Approved | Approved | - |
| Dimethenamid^a^^[[1]](#footnote-1)^ | Herbicide | α-Chloroacetamides | Inhibition of Very Long-Chain Fatty Acid Synthesis | 0.001 | 1 | Not approved | Not approved | 23/05/2006 |
| Dimethomorph | Fungicide | Cinnamic acid amides | Cell wall biosynthesis | 0.001 | 1 | Approved | Not approved | 20/05/2024 |
| Dimoxystrobin | Fungicide | Oxamino-acetamides | Respiration | 0.001 | 1 | Approved | Not approved | 31/07/2023 |
| Diuron | Herbicide | Ureas | Inhibition of Photosynthesis at PSll - Serine 264 Binders | 0.001 | 1 | Approved | Not approved | 30/09/2020 |
| Epoxiconazole | Fungicide | Triazoles | Sterol biosynthesis in membranes | 0.001 | 1 | Approved | Not approved | 30/04/2020 |
| Fenpropidin | Fungicide | Piperidines | Sterol biosynthesis in membranes | 0.001 | 1 | Approved | Approved | - |
| Fenpropimorph | Fungicide | Morpholines | Sterol biosynthesis in membranes | 0.001 | 1 | Approved | Not approved | 30/04/2019 |
| Fluazinam | Fungicide | 2,6-dinitroanilines | Respiration | 0.001 | 1 | Approved | Approved | - |
| Fludioxonil | Fungicide | Phenylpyrrole | Signal transduction | 0.005 | 1 | Approved | Approved | - |
| Flufenacet | Herbicide | α-Oxyacetamides | Inhibition of Very Long-Chain Fatty Acid Synthesis | 0.001 | 1 | Approved | Approved | - |
| Fluopicolide | Fungicide | Pyridinylmethylbenzamides | Cytoskeleton and motor protein | 0.001 | 1 | Approved | Approved | - |
| Fluopyram | Fungicide | Pyridinyl-ethylbenzamides | Respiration | 0.001 | 1 | Approved | Approved | - |
| Fluoxastrobin | Fungicide | Dihydro-dioxazines | Respiration | 0.001 | 1 | Approved | Approved | - |
| Fluquinconazole | Fungicide | Triazoles | Sterol biosynthesis in membranes | 0.001 | 1 | Approved | Not approved | 31/12/2021 |
| Fluroxypyr | Herbicide | Pyridyloxy-carboxylates | Auxin Mimics | 0.010 | 1 | Approved | Approved | - |
| Glyphosate | Herbicide | Glycine | Inhibition of Enolpyruvyl Shikimate Phosphate Synthase | 0.025 | 3 | Approved | Approved | - |
| Imazalil | Fungicide | Imidazoles | Sterol biosynthesis in membranes | 0.001 | 1 | Approved | Approved | - |
| Imidacloprid | Insecticide | Neonicotinoide | Nicotinic acetylcholine receptor (nAChR) competitive modulators | 0.001 | 1 | Approved | Not approved | 01/12/2020 |
| Indoxacarb | Insecticide | Oxadiazine | Voltage-dependent sodium channel blocker | 0.001 | 1 | Approved | Not approved | 19/12/2021 |
| Isoproturon | Herbicide | Ureas | Inhibition of Photosynthesis at PSll - Serine 264 Binders | 0.001 | 1 | Not approved | Not approved | 30/06/2016 |
| Isoxaben | Herbicide | Benzamides | Inhibition of Cellulose Synthesis | 0.001 | 1 | Approved | Approved | - |
| Lenacil | Herbicide | Uracils | Inhibition of Photosynthesis at PSll - Serine 264 Binders | 0.005 | 1 | Approved | Approved | - |
| Linuron | Herbicide | Ureas | Inhibition of Photosynthesis at PSll - Serine 264 Binders | 0.001 | 1 | Not approved | Not approved | 03/03/2017 |
| MCPA | Herbicide | Phenoxy-carboxylates | Auxin Mimics | 0.005 | 1 | Approved | Approved | - |
| Metalaxyl | Fungicide | Acylalanines | Nucleic acids metabolism | 0.001 | 1 | Approved | Approved | - |
| Metamitron | Herbicide | Triazinones | Inhibition of Photosynthesis at PSll - Serine 264 Binders | 0.005 | 1 | Approved | Approved | - |
| Metconazole | Fungicide | Triazoles | Sterol biosynthesis in membranes | 0.005 | 1 | Approved | Approved | - |
| Metolachlorb^[[2]](#footnote-2)^ | Herbicide | α-Chloroacetamides | Inhibition of Very Long-Chain Fatty Acid Synthesis | 0.001 | 1 | Not approved | Not approved | NA |
| Metrafenone | Fungicide | Benzophenone | Cytoskeleton and motor protein | 0.001 | 1 | Approved | Approved | - |
| Myclobutanil | Fungicide | Triazoles | Sterol biosynthesis in membranes | 0.001 | 1 | Approved | Not approved | 31/05/2021 |
| Penconazole | Fungicide | Triazoles | Sterol biosynthesis in membranes | 0.001 | 1 | Approved | Approved | - |
| Pendimethalin | Herbicide | Dinitroanilines | Inhibition of Microtubule Assembly | 0.001 | 1 | Approved | Approved | - |
| Penflufen | Fungicide | Pyrazole-4-carboxamides | Respiration | 0.001 | 1 | Approved | Not approved | 31/01/2024 |
| Prochloraz | Fungicide | Imidazoles | Sterol biosynthesis in membranes | 0.001 | 1 | Approved | Not approved | 31/12/2021 |
| Prometryn | Herbicide | Triazines | Inhibition of Photosynthesis at PSll - Serine 264 Binders | 0.001 | 1 | Not approved | Not approved | 28/04/2004 (835/2004 EC) |
| Propiconazole | Fungicide | Triazoles | Sterol biosynthesis in membranes | 0.001 | 1 | Approved | Not approved | 19/12/2018 |
| Prosulfocarb | Herbicide | Thiocarbamates | Inhibition of Very Long-Chain Fatty Acid Synthesis | 0.001 | 1 | Approved | Approved | - |
| Phthalimide (PTI) | Fungicide | Dicarboximides | Signal transduction | 0.010 | 1 | Approved | Approved | - |
| Pyraclostrobin | Fungicide | Methoxy-carbamates | Respiration | 0.001 | 1 | Approved | Approved | - |
| Pyriofenone | Fungicide | Benzoylpyridine | Cytoskeleton and motor protein | 0.001 | 1 | Approved | Approved | - |
| Quinoxyfen | Fungicide | Aryloxyquinoline | Signal transduction | 0.001 | 1 | Approved | Not approved | 27/12/2018 |
| Simazine | Herbicide | Triazines | Inhibition of Photosynthesis at PSll - Serine 264 Binders | 0.001 | 1 | Not approved | Not approved | 2004 (2004/247/EC) |
| Tebuconazole | Fungicide | Triazoles | Sterol biosynthesis in membranes | 0.001 | 1 | Approved | Approved | - |
| Terbuthylazine | Herbicide | Triazines | Inhibition of Photosynthesis at PSll - Serine 264 Binders | 0.001 | 1 | Approved | Approved | - |
| Terbutryn | Herbicide | Triazines | Inhibition of Photosynthesis at PSll - Serine 264 Binders | 0.001 | 1 | Not approved | Not approved | 25/06/2003 for agricultural use; 30/06/2007 for other uses |
| Thiamethoxam | Insecticide | Neonicotinoide | Nicotinic acetylcholine receptor (nAChR) competitive modulators | 0.001 | 1 | Approved | Not approved | 30/04/2019 |
| Triadimenol | Fungicide | Triazoles | Sterol biosynthesis in membranes | 0.005 | 1 | Approved | Not approved | 31/08/2019 |

**Supplementary Results S1.**

**Specific pesticide concentration effects**

Varying results were also found for soil biodiversity assessed with metabarcoding (richness, diversity and functional group relative abundance) between glyphosate and AMPA. Although AMPA is a metabolite of glyphosate, it exhibited contrasting patterns with soil biodiversity (Extended Data Fig. S5). This discrepancy could be attributed to their differential occurrence across soil samples, which were not fully correlated (Pearson’s r = 0.68, Spearman’s ρ = 0.61; p-value < 0.05). The higher persistence of AMPA in soils (detected at 203 sites) compared to glyphosate (detected at 59 sites) may explain this difference.

The diversity of all functional gene groups was significantly influenced by pesticide concentrations, with each functional gene group affected by 2 to 11 pesticides (Fig. 3). Notably, 11 out of 28 pesticides had a particularly broad impact, influencing 10 to 25 gene groups (Fig. 3). In particular, fluopyram, clothianidin, tebuconazole, carbendazim, and AMPA impacted 20 to 25 gene groups. Certain pesticides, such as imidacloprid and bixafen were predominantly negatively related to gene diversity in croplands, while others, like fluopyram, clothianidin, tebuconazole and pendimethalin, were mostly positively related. These trends remained consistent when grassland samples were included (Supplementary Fig. S2 of Supplementary Data File S3).

**Supplementary Results S2.**

**Model evaluation**

Model performance was assessed based on the squared correlation between predicted and observed values (*r²*). For models based on metrics derived from metabarcoding data (i.e., community richness and diversity, multidiversity index, functional group relative abundance), performance ranged from 10% to 12% for nematode and arthropod diversity, to 46% to 48% for bacterial richness and diversity, and from 6% for protistan animal parasites to 60% for bacterial chemoheterotrophs (Supplementary Tables S16 and S17 of Supplementary Data File S4). Consistent findings were observed in croplands only (Supplementary Tables S12 and S13 of Supplementary Data File S2). These results illustrate a limitation in available information for less-studied organisms and functional groups, as well as potential predictors not accounted for in our models.

Bacterial models consistently outperformed other groups in predicting processes related to C, N and P cycling across all ecosystem types (Supplementary Tables S18-S20 of Supplementary Data File S4) and when focusing on croplands only (Supplementary Tables S14-S16 of Supplementary Data File S2). Specifically, fungal and bacterial models consistently performed the best in explaining most C-related processes in metagenomics data, with bacteria leading in many cases. In contrast, archaea showed limited effectiveness across these processes, and faunal models generally underperformed. For N-related processes, bacterial models demonstrated the highest performance, particularly in N-fixation (49%), denitrification (45%), and dissimilatory nitrate reduction (DNRA; 46%). Archaeal models exhibited moderate performance in DNRA (31%) and organic N degradation (30%) but were less effective in other processes. Fungal and faunal models generally had limited predictive power, though fungi performed slightly better in nitrate assimilation (35%). In P-related processes, bacteria outperformed other groups in organic P degradation (64%), while archaea outperformed in mineral P import (55%). However, bacterial models for phosphonate degradation performed poorly (10%).

Residual patterns in the QQ plots (see DHARMa files in the output folders together with the R scripts from the *Code availability* section) suggest the presence of additional environmental factors not captured by the current set of predictors. Nevertheless, since the objective was to test the relationships of pesticide concentrations across various biodiversity metrics, a consistent set of predictors was used across different taxonomic and functional groups and genes, enabling valid comparisons of variable importance.

Multicollinearity was detected in two functional group models (AMF and protist plant parasites). Although pairwise correlations did not reveal highly correlated (|r| > 0.5) pairs of predictors (including the ones with a high VIF value), principal component analyses (PCA) showed clustering among several predictors. For AMF, the concentrations of fenpropidin, chloridazon, difenoconazole, diflufenican and carbendazim were positively correlated. For protist plant parasites, pH correlated with several environmental predictors (e.g., potassium, temperature and precipitation in sample month). As a result, variable importance values in these models cannot be directly interpreted due to multicollinearity, which prevents clear attribution of importance to correlated predictors. Similarly, multicollinearity across driver types (e.g., soil properties and climate) complicates partitioning variance uniquely attributed to each driver type. Despite these issues, the conclusion that pesticide concentrations are relevant predictors remains valid.

Variation partitioning further indicated high shared variances between driver types (e.g., Fig. 4), underscoring the challenge of attributing specific effects to unique drivers. This emphasizes the need for further studies on pesticide persistence under varying soil and climatic conditions. Certain models showed some negative shared variances, a mathematical artifact of how variation is partitioned among sets of predictors. According to Legendre and Legendre (2012)^100^, such values indicate that groups of predictors collectively explain the dependent variable better than the sum of the individual effects of these predictors. In general, it is recommended to interpret these negative values as zero. In our models, variance inflation factors (VIFs) confirmed that collinearity was not problematic, supporting the validity of the results despite the presence of negative shared variances.

**Supplementary Results S3.**

**Potential persistence of individual pesticides**

We examined how environmental properties could relate to the persistence of pesticides, specifically focusing on residues that influenced soil communities and functional groups (identified in Fig. 3). Across all ecosystem types, pesticides which highly contributed to soil biodiversity responses were more likely detected in sites with less leaching (e.g., in sites with lower monthly precipitation and precipitation seasonality; Supplementary Data File S8), in more compacted soils (i.e., higher bulk density, lower coarse fragments) and in sites with higher nutrient retention capacity (i.e., lower sand content, higher C: N ratio, potassium and phosphorus contents). Pesticides influencing the diversity of functional gene groups involved in the C, N or P cycles were also found in the sites exhibiting lower temperature variations (i.e., lower mean diurnal range). These findings point to lower pesticide degradation, potentially connected to lower microbial activities in colder areas^101^. Further experimental validation would be necessary to confirm all observed associations.

In particular, tebuconazole, imidacloprid, and clothianidin were detected in sites with higher pH, while AMPA and carbendazim were detected in sites with lower pH (Supplementary Tables S10 and S11 of Supplementary Data File S4). In addition, glyphosate was observed to be accumulating in sites with a higher C:N ratio and/or a lower monthly precipitation, while boscalid was observed in sites with a lower C:N ratio (Supplementary Data File S8). When looking at croplands only, we found pesticides to be more present in sites with lower clay content (Supplementary Tables S10 and S11 of Supplementary Data File S2, Supplementary Data File S7). There, pesticides related to changes in C, N, P functional gene groups’ diversity were more generally found in sites with a lower pH (Supplementary Data File S7). For more details, see Supplementary Tables S10 and S11 of Supplementary Data File S2, Supplementary Tables S11 and S12 of Supplementary Data File S4, Supplementary Data File S7, Supplementary Data File S8.

**Supplementary Results S4.**

**Pesticide occurrence and ecotoxicological risk assessment (NOEC)**

The fungicide epoxiconazole and the insecticides imidacloprid and chlorpyrifos posed the highest ecotoxicological risk (based on the risk quotient, calculated as the ratio of pesticide concentration to the minimum no observed effect concentration – NOEC_min_ value; see Materials and Methods), particularly in croplands, despite being detected at low concentrations across sites (Supplementary Fig. S1). The cumulative risk associated with pesticide residues was highest in annual croplands, and increased with an increasing number of residues detected per site across all ecosystem types (R² = 0.44, p-value < 0.01; Supplementary Fig. S2). However, cumulative risk and cumulative concentration were not correlated (R² = 0.07, p-value < 0.01).

Here, we further used the cumulative pesticide risk per site, along with equivalent risk metrics calculated separately for insecticides, herbicides, and fungicides, as indicators of ecotoxicological risk. These were examined in relation to soil biodiversity, with no assumption of direct causality, and compared to descriptive metrics of pesticide occurrence (Supplementary Fig. S3). This exploratory approach provides a simplified overview of potential associations between aggregated metrics for pesticide risk, occurrence, and soil taxa and their functional roles (ignoring confounding factors like land use or environment). It highlights indicative trends that may warrant further investigation.

Overall, soil taxonomic and functional biodiversity showed weak correlations (Spearman’s |ρ| < 0.3) with aggregated metrics for pesticide occurrence and risk. Among relevant correlations (|ρ| ≥ 0.3; p-value < 0.05), AMF exhibited negative associations with both fungicide occurrence (ρ=-0.40) and risk (ρ=-0.39; Supplementary Fig. S3; Supplementary Fig. S5). At the functional gene level, the diversity of bacterial gene groups involved in nitrogen turnover was positively correlated with increasing pesticide risk. Notably, across all ecosystems, bacterial ammonia oxidation was positively associated with cumulative pesticide risk (ρ=0.41). In croplands, bacterial nitrification was related to higher herbicide risk (ρ=0.43), while bacterial denitrification was associated with increased fungicide risk (ρ=0.40).

The metrics for pesticide risk did not emerge as better descriptors than the simplest metrics for pesticide occurrence, such as the total number of pesticides detected (i.e., correlation coefficients were in the same range, despite not fully correlating with these metrics; Supplementary Fig. S3 and S4). Additionally, aggregated pesticide metrics failed to capture key patterns: arthropod communities showed no correlation with the number of insecticides detected (Supplementary Fig. S3), yet imidacloprid concentration was a predictor of arthropod richness and diversity (e.g., Fig. 3; Supplementary Fig. S5). Similarly, while the presence of more fungicides related positively to bacterial communities, higher concentrations of bixafen reduced their diversity. These contrasting effects underscore the limitations of grouping metrics and the need to assess the impacts of specific pesticide concentrations to better understand soil biodiversity responses.

Crucially, the risk quotient used in this study is based on toxicity data (NOEC) obtained for standard model organisms: annelids (*Eisenia fetida*, *E. andrei*, *Aporrectodea caliginosa*, *A.longa*, *A.icterica*, *Lumbricus* *rubellus*, *L. terrestris*, *Perionyx excavatus*, *Enchytraeus albidus*), collembolans (*Folsomia candida*, *F. fimetaria, Heteromurus nitidus*) and mites (*Hypoaspis aculeifer*)^102^. However, no reference values are available for the wide range of soil organisms considered in our analyses, such as archaea, bacteria, fungi or protists. As a result, this risk-based indicator widely used in regulatory context fails to account for a large part of the soil biodiversity. In line with this, our findings highlight a critical gap: ecotoxicological risk assessments based on NOECs and aggregated metrics may overlook key community-level responses. Because NOEC values are derived from a limited set of species under controlled conditions, they do not reflect the ecological complexity of soil communities or the functional roles of (microbial) groups. Consequently, building GLMs or other predictive models based on such toxicity profiles is inadequate, as these profiles exclude microbial taxa and any indirect effect occurring at the community or functional level.

Here, we propose some avenues for refining the regulatory frameworks based on risk assessment. These could include:

- define protection goals at the level of taxonomic diversity (e.g. community richness, Shannon diversity, using metabarcoding) and functional potential (e.g. diversity of various functional gene groups involved in major environmental pathways, using metagenomics).
- to develop acceptable exposure thresholds based on in-field community-level and functional endpoints, moving beyond the existing paradigm of relying on survival or reproduction toxicity data from few species. Controlled studies using in-field soil communities could help define concentration ranges or dose-response curves where a significant shift in community diversity (loss of taxa) or drop in functional potential occurs.
- to conduct laboratory experiments using in-field communities sampled across environmental gradients (e.g. different ecosystem types, soil types, climatic contexts). This approach can help refine and validate findings from controlled studies by incorporating real-world confounding factors such as land use, historical pesticide exposure (including mixtures), and broader environmental variability.


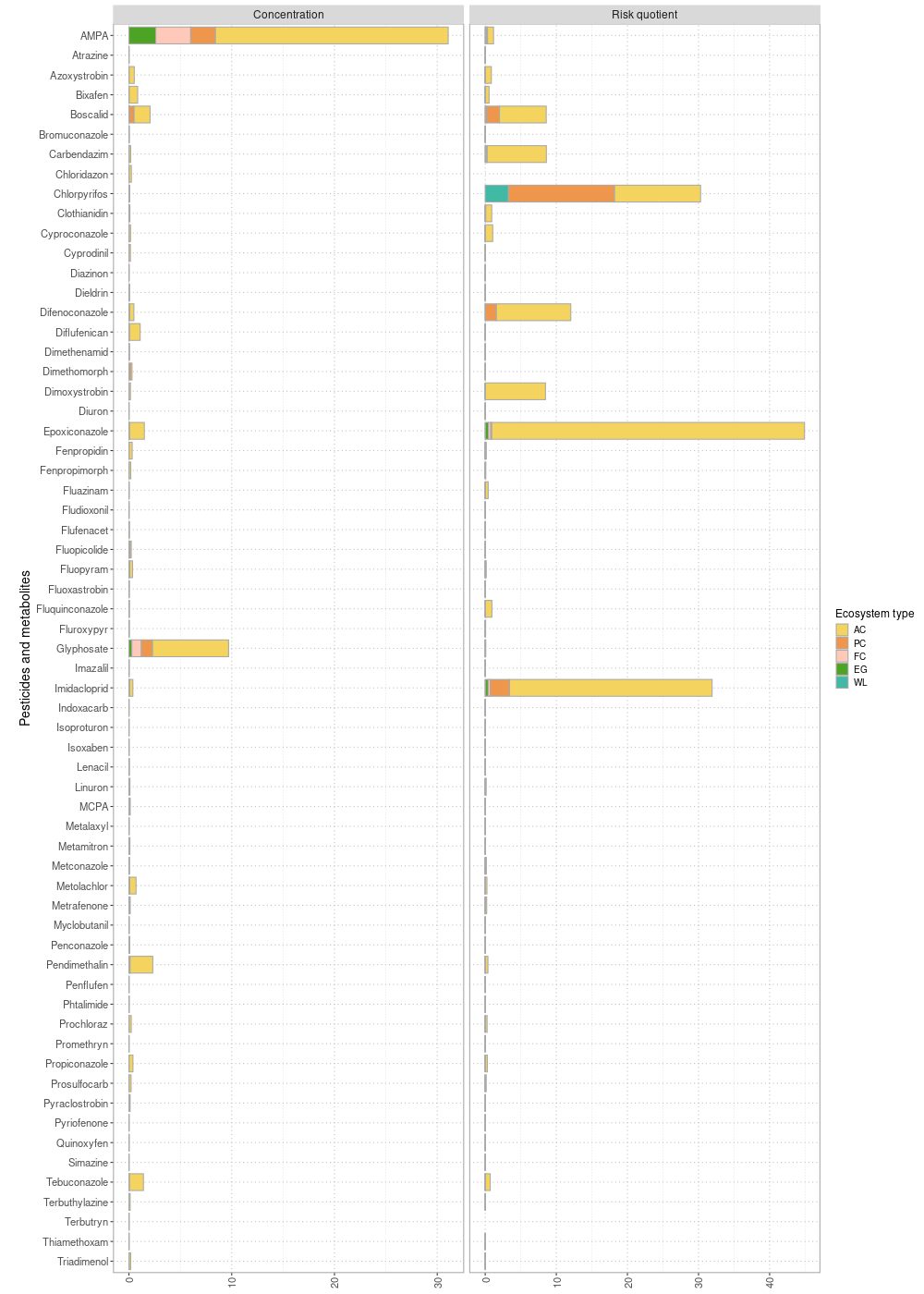


**Supplementary Fig. S1. Distribution of pesticide concentration and risk quotient across ecosystem types (n=373 sites).** Pesticide concentration (mg/kg) and risk quotient (pesticide concentration/NOECmin) for individual pesticides detected (summed values per pesticide) per ecosystem type (annual croplands (AC), permanent croplands (PC), former croplands recently converted to grasslands (FC), extensive grasslands (EG) and woodlands (WL)). The risk quotient is established for 61 products for which a NOEC_min_ value is reported (chloridazon and terbutryn are not included).


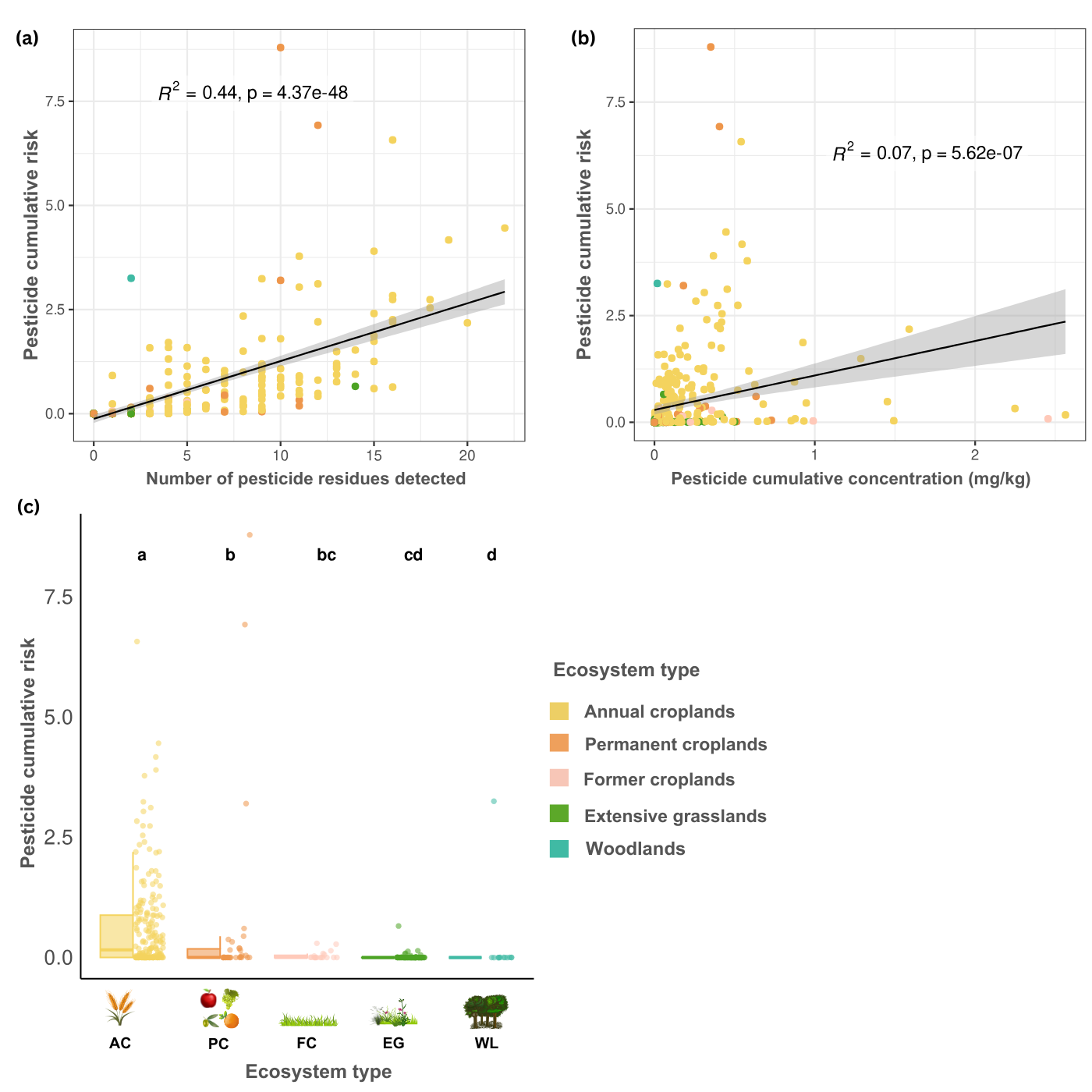


**Supplementary Fig. S2.** **Regression models between pesticide metrics (a,b) and distribution of pesticide cumulative risk per site across ecosystems (c)** (for n=373 sites). Regression models between **(a)** the pesticide cumulative risk and the number of pesticides per site, and **(b)** the pesticide cumulative risk and the pesticide cumulative concentration per site. R-squared (R²) and p-value (p) are derived from a linear model. **(c)** Boxplots of pesticide cumulative risk across ecosystem types (sum of risk quotients for a site, where a risk quotient is the ratio of a pesticide concentration and its NOEC_min_). Different letters indicate significant differences (two-sided pairwise Wilcoxon multiple comparison test (with a Benjamini & Hochberg’s correction)) between ecosystem types (for p-values, AC-PC=0.00354, AC-FC=0.00081, AC-EG<2e-16, AC-WL=0.00014, PC-FC=0.37318, PC-EG=0.00225, PC-WL=0.01909, FC-EG=0.05459, FC-WL=0.04932, EG-WL=0.10221). The cumulative risk is established on 61 products for which a NOEC_min_ value is reported: chloridazon and terbutryn were not included in the calculations.


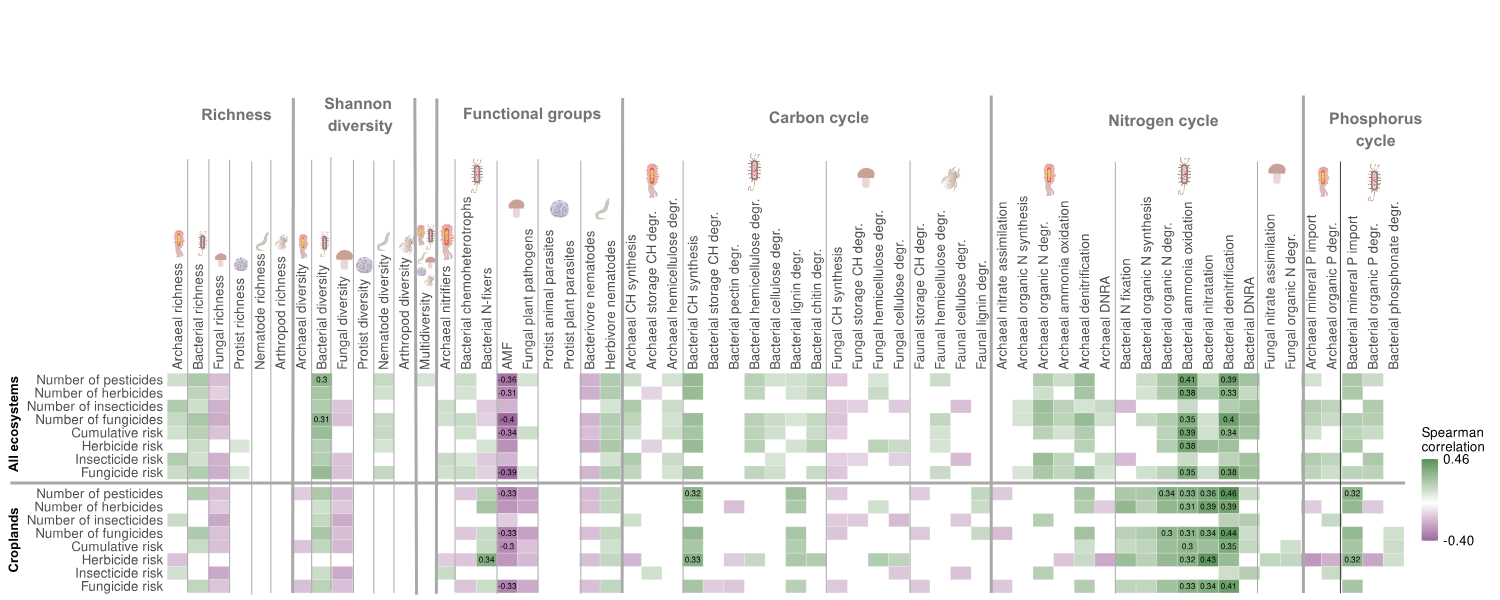


**Supplementary Fig. S3.** **Relationships (Spearman correlation) between soil biodiversity and pesticide** **metrics** across all ecosystems and croplands only for 63 detected pesticides. Pesticide metrics include the total number of pesticides detected, number of insecticides, of fungicides, of herbicides, cumulative risk of all pesticides, risk calculated for insecticides, for fungicides and for herbicides. Soil biodiversity was assessed by metabarcoding: richness, Shannon diversity, multidiversity index, functional group relative abundance (%) and metagenomics: functional gene group diversity (pmOGs), for all ecosystem types (n=373 sites for metabarcoding analyses, n=349 for metagenomics analyses) and croplands only (n=244 sites for metabarcoding analyses and n=234 for metagenomics analyses). Only significant correlations (|ρ| ≥ 0.3 and p-value < 0.05) are shown. Numbers on the scale and in the matrix show the co-variance between variables and hence, the strength of the relationship (green being positively related and purple being negatively related). Columns with similar colours indicate the same direction of correlation between pesticide metrics and soil biodiversity, reflecting underlying linear relationships among the pesticide metrics (see Supplementary Fig. S4). For metagenomics analyses, only the functional gene groups presenting at least one significant correlation with a pesticide metric are shown.


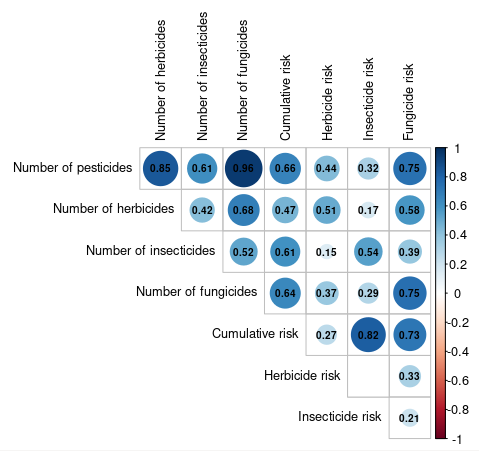


**Supplementary Fig. S4**. **Linear correlations (Pearson) between metrics for pesticide occurrence and risk** for all ecosystem types (n= 373 sites) and the 63 pesticides initially detected. Darker blue circles correspond to pairs of variables with high positive significant correlation values. Only significant correlations are shown.


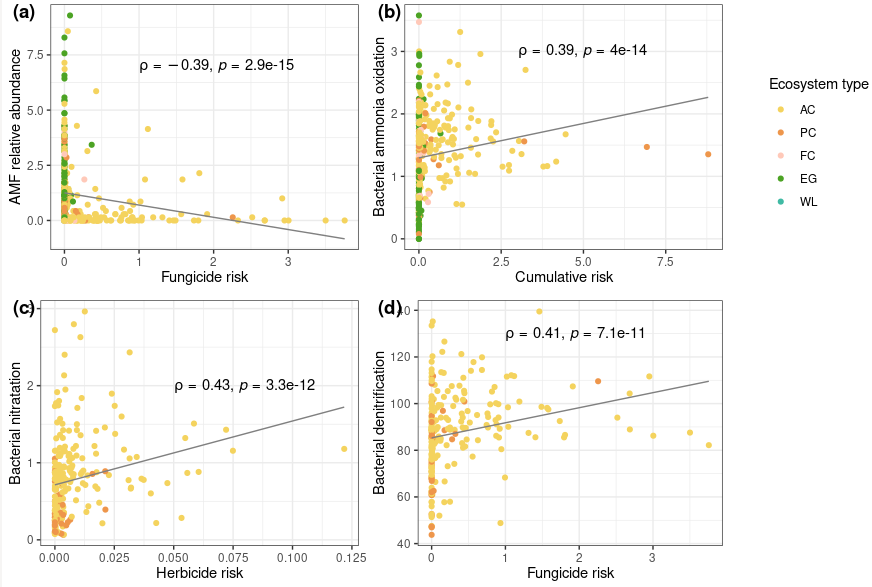


**Supplementary Fig. S5.** **Relationships (Spearman correlations) between soil biodiversity and pesticide risk** for **(a)** arbuscular mycorrhizal fungi (AMF) relative abundance and fungicide risk across all ecosystems (n=373 sites), **(b)** bacterial ammonia oxidation and cumulative risk in croplands and grasslands (n=349 sites), **(c)** bacterial nitratation and herbicide risk in croplands (n=234 sites) and **(d)** bacterial denitrification and fungicide risk in croplands (n=234 sites). Spearman correlation coefficient (ρ) and p-values (p) are given in each plot. Points are coloured according to ecosystem type (annual croplands (AC), permanent croplands (PC), former croplands recently converted to grasslands (FC), extensive grasslands (EG) and woodlands (WL)). The direction of the relationship is shown with a regression line.

**Supplementary Discussion**

Future research on the effects of pesticides on soil biodiversity requires increasing the number of sampling sites and conducting regular monitoring. This would facilitate more advanced analyses (e.g. counterfactual approaches). Also, this information is crucial to enhance future studies’ reliability, especially since pesticide responses vary across years^103^. Systematically sampling soils near cropland areas could help validate the cross-ecosystem patterns observed in this study by increasing the number of non-cropland sites with potential pesticide residues and reducing the current sampling bias toward cropland soils, which hosted most of the detected pesticides. Certain products, such as nematicides, often remain understudied and underrepresented in regulations^104,105^. Our study included two nematicides that we classified as fungicide or herbicide according to their listing^94,95^, highlighting a gap that needs to be addressed to ensure more comprehensive environmental risk assessments. Moreover, although a risk quotient below 1 indicates no harm to soil taxa^106^, we found negative associations between herbicide concentrations (e.g., glyphosate) and several soil taxa, in line with previous findings^107,108^, while other taxa showed positive trends. This highlights another limitation in current ecological risk assessments, which often fail to account for indirect effects^109^.

In addition, more information on land management practices are needed. Previous research found connections between lower pesticide use in crop rotations^110^ compared to greenhouse cultivation^111,112^—and their intertwined effects on soil processes and biota. For instance, excessive mineral fertilisation may lead to phosphorus loss when combined with an organophosphorus pesticide^113^, while antibiotic contamination can worsen with concurrent pesticide application^114,115^. Additionally, conventional tillage can increase pesticide leaching^116^, while conservation tillage practices —often involving pesticide-treated seeds—may shift fungal communities^117^. Additional factors such as pesticide bioavailability, degradation kinetics, and interactions with soil organic matter were not included in this study, but could provide important insights into actual pesticide exposure for soil biota. Moreover, pesticide persistence alone is not a sufficient indicator of ecological risk: highly persistent compounds may have low bioavailability, while rapidly degrading ones can still exert significant effects on soil communities.

Advancing soil biodiversity assessment methods and establishing robust baseline data on indigenous soil communities prior to pesticide application are crucial for drawing more reliable risk assessments of pesticide effects on soil biodiversity^118^. Improved methods (e.g., by considering larger volumes of soil) could capture a wider spectrum of taxonomic groups, including larger and more mobile organisms like annelids^119^. Also, deeper knowledge is required at the family, genus or species levels to infer functions based on taxonomy, particularly for protists and fauna^120^. Enhanced taxonomic and functional databases are needed^121^ to gain functional representativeness of soil communities by annotating more ASVs (or OTUs), at finer taxonomic levels (e.g., species). Finally, recent metabarcoding studies have reported a higher diversity of protists^122^, bacteria and fungi^123^, and overall eukaryotes^124^ in intensively managed ecosystems. However, DNA from deceased organisms (necromass DNA) may accumulate in soils, potentially obscuring the active community profile^125^. To disentangle this "DNA memory effect" from the current community composition, methods targeting intracellular DNA^126^ or RNA could offer valuable insights into community dynamics.

**Other Supplementary Information**

**Supplementary Data File S1:** Classification table for all pesticides and metabolites including information on their type and no observed effect concentration (NOEC).

**Supplementary Data File S2: For croplands only** – Supplementary tables S1 to S16 supporting the metabarcoding and metagenomics analyses for croplands only.

**Supplementary Table S1 – Data File S2. For croplands only - Variation partitioning between drivers explaining soil organism richness.** Percentages of explained variance of soil biota diversity by pesticide residue concentrations, soil properties, climate, ecosystem type together with the shared variance and residuals, for the organism observed ASV/OTU richness and the multidiversity index. This table shows the results when the data of cropland sites are analysed (n=244 sites) while the results in Supplementary Table S1 of Supplementary Data File S4 are based on all ecosystem types (n=373 sites).

**Supplementary Table S2 – Data File S2. For croplands only - Variation partitioning between drivers explaining soil organism Shannon diversity.** Percentages of explained variance of soil biota diversity by pesticide residue concentrations, soil properties, climate, ecosystem type together with the shared variance and residuals, for the organism Shannon diversity index. This table shows the results when the data of cropland sites are analysed (n=244 sites) while the results in Supplementary Table S2 of Supplementary Data File S4 are based on all ecosystem types (n=373 sites).

**Supplementary Table S3 – Data File S2.** **For croplands only - Variation partitioning between drivers explaining soil functional group relative abundance.** Percentages of explained variance of soil biota diversity by pesticide residue concentrations, soil properties, climate, ecosystem type together with the shared variance and residuals, for the functional group relative abundance, for associated generalised linear models. This table shows the results when the data of cropland sites are analysed (n=244 sites) while the results in Supplementary Table S3 of Supplementary Data File S4 are based on all ecosystem types (n=373 sites).

**Supplementary Table S4 – Data File S2. For croplands only - Variation partitioning between drivers explaining functional gene groups involved in C cycle.** Percentages of explained variance of soil biota diversity by pesticide residue concentrations, soil properties, climate, ecosystem type together with the shared variance and residuals, for the functional gene groups involved in C cycle. This table shows the results when the data of cropland sites are analysed (n=234 sites) while the results in Supplementary Table S4 of Supplementary Data File S4 are based on all ecosystem types (n=349 sites).

**Supplementary Table S5 – Data File S2. For croplands only - Variation partitioning between drivers explaining functional gene groups involved in N cycle.** Percentages of explained variance of soil biota diversity by pesticide residue concentrations, soil properties, climate, ecosystem type together with the shared variance and residuals, for the functional gene groups involved in N cycle. This table shows the results when the data of cropland sites are analysed (n=234 sites) while the results in Supplementary Table S5 of Supplementary Data File S4 are based on all ecosystem types (n=349 sites).

**Supplementary Table S6 – Data File S2. For croplands only - Variation partitioning between drivers explaining functional gene groups involved in P cycle.** Percentages of explained variance of soil biota diversity by pesticide residue concentrations, soil properties, climate, ecosystem type together with the shared variance and residuals, for the functional gene groups involved in P cycle. This table shows the results when the data of cropland sites are analysed (n=234 sites) while the results in Supplementary Table S6 of Supplementary Data File S4 are based on all ecosystem types (n=349 sites).

**Supplementary Table S7 – Data File S2. For croplands only - Selected environmental properties for metabarcoding.** Soil properties and climatic variables feature-selected next to pesticides and ecosystem type in the generalised linear model. This table shows the results when the data of cropland sites are analysed (n=244 sites) while the results in Supplementary Table S7 of Supplementary Data File S4 are based on all ecosystem types (n=373 sites).

**Supplementary Table S8 – Data File S2. For croplands only - Selected environmental properties for metagenomics.** Soil properties and climatic variables feature-selected next to pesticides and ecosystem type in the generalised linear model. This table shows the results when the data of cropland sites are analysed (n=234 sites) while the results in Supplementary Table S8 of Supplementary Data File S4 are based on all ecosystem types (n=349 sites).

**Supplementary Table S9 – Data File S2. For croplands only - Highest Pearson (linear) correlations (|r| > 0.5, p-value < 0.05) between initial set of predictors considered in the generalised linear models.**

**Supplementary Table S10 – Data File S2. For croplands only - Significant environmental variables in sites where a pesticide residue significantly impacted various soil organism or functional groups (selected in GLM).** A p-value is reported from a Kruskal-Wallis test when found significant: * refers to p-value < 0.05; ** to p-value < 0.01; *** to p-value < 0.001. An empty cell refers to a p-value > 0.05. The sum on the last row refers to the number of times an environmental variable significantly differed in presence or absence of a pesticide. Complete p-values and mean values of environmental variables are available in Supplementary Data File S7. This table shows the results when the data of cropland sites are analysed (n=244 sites) while the results in Supplementary Table S10 of Supplementary Data File S4 are based on all ecosystem types (n=373 sites).

**Supplementary Table S11 – Data File S2**. For croplands only - Significant environmental variables in sites where a pesticide residue significantly impacted various functional gene groups involved in C, N, P cycles (selected in GLM). A p-value is reported from a Kruskal-Wallis test when found significant: * refers to p-value < 0.05; ** to p-value < 0.01; *** to p-value < 0.001. An empty cell refers to a p-value > 0.05. The sum on the last row refers to the number of times an environmental variable significantly differed in presence or absence of a pesticide. Complete p-values and mean values of environmental variables are available in Supplementary Data File S7. This table shows the results when the data of cropland sites are analysed (n=234 sites) while the results in Supplementary Table S11 of Supplementary Data File S4 are based on all ecosystem types (n=349 sites).

**Supplementary Table S12 – Data File S2. For croplands only – Model performance for the generalised linear models including pesticide concentrations and environmental variables in explaining soil organism richness, Shannon diversity and multidiversity.** This table shows the results when the data of cropland sites are analysed (n=244 sites) while the results in Supplementary Table S16 of Supplementary Data File S4 are based on all ecosystem types (n=373 sites).

**Supplementary Table S13 – Data File S2. For croplands only - Model performance for the generalised linear models including pesticide concentrations and environmental variables in explaining functional group relative abundance.** This table shows the results when the data of cropland sites are analysed (n=244 sites) while the results in Supplementary Table S17 of Supplementary Data File S4 are based on all ecosystem types (n=373 sites).

**Supplementary Table S14 – Data File S2. For croplands only - Model performance for the generalised linear models including pesticide concentrations and environmental variables in explaining functional gene groups involved in C cycle.** This table shows the results when the data of cropland sites are analysed (n=234 sites) while the results in Supplementary Table S18 of Supplementary Data File S4 are based on all ecosystem types (n=349 sites).

**Supplementary Table S15 – Data File S2. For croplands only - Model performance for the generalised linear models including pesticide concentrations and environmental variables in explaining functional gene groups involved in N cycle.** This table shows the results when the data of cropland sites are analysed (n=234 sites) while the results in Supplementary Table S19 of Supplementary Data File S4 are based on all ecosystem types (n=349 sites).

**Supplementary Table S16 – Data File S2. For croplands only - Model performance for the generalised linear models including pesticide concentrations and environmental variables in explaining functional gene groups involved in P cycle.** This table shows the results when the data of cropland sites are analysed (n=234 sites) while the results in Supplementary Table S20 of Supplementary Data File S4 are based on all ecosystem types (n=349 sites).

**Supplementary Data File S3: For all ecosystem types** – Supplementary figures supporting the metabarcoding and metagenomics analyses across all ecosystem types.

**Supplementary Fig. S1 – Data File S3. For all ecosystem types – Soil biodiversity (assessed by metabarcoding) responses to key pesticide concentrations** (herbicides H, metabolite of a herbicide MH, fungicides F or insecticides I). Positive or negative relationship of concentration of pesticides selected in the GLMs with **(a)** soil organism observed richness, **(b)** Shannon diversity, **(c)** functional group relative abundance and **(d)** multidiversity. Horizontal bars correspond to the variable importance (VIP) coloured in green (positive relationship) or purple (negative), according to the coefficient sign of each pesticide in the associated GLM. Variable importance was calculated based on the GLM including pesticide concentrations, soil properties, climate, and ecosystem type information.

**Supplementary Fig. S2 – Data File S3. For all ecosystem types – Soil C, N, P functional gene groups responses to key pesticide concentrations** (herbicides H, metabolite of a herbicide MH, fungicides F or insecticides I). Positive or negative relationship of concentration of pesticides retained in the GLMs with the diversity of each functional gene group involved in the C, N, and P cycles. Horizontal bars correspond to the variable importance (VIP) coloured in green (positive relationship) or purple (negative), according to the coefficient sign of each pesticide in the associated GLM. Variable importance was calculated based on the GLM including pesticide concentrations, soil properties, climate, and ecosystem type information.

**Supplementary Fig. S3 – Data File S3. For all ecosystem types – Linear (Pearson) correlations between the initial set of predictors (i.e., before feature-selection) used in the generalised linear models from analyses performed on all ecosystem types (n=373 sites)**, i.e., all environmental variables next to the most occurring pesticides across all ecosystem types (20 pesticides out of 63, all other pesticides with near-zero variance are not kept). The highest correlations are displayed in Supplementary Table S9 of Supplementary Data File S4.

**Supplementary Fig. S4 – Data File S3. For all ecosystem types – Explained variance (in %) of soil biodiversity by selected variables**: pesticide residue concentrations (red), soil properties (brown), climate (blue), ecosystem type (green) together with the shared variance (yellow) for the organism observed ASV/OTU richness, Shannon diversity, multidiversity index, each functional group relative abundance and each functional gene group diversity involved in the C, N, P cycles, using data of all ecosystem types (n=373 sites for metabarcoding analyses, n=349 sites including croplands and grasslands for metagenomics analyses).

**Supplementary Data File S4: For all ecosystem types –**Supplementary tables S1 to S20 supporting the metabarcoding and metagenomics analyses across all ecosystem types.

**Supplementary Table S1 – Data File S4. For all ecosystem types - Variation partitioning between drivers explaining soil organism richness and multidiversity.** Percentages of explained variance of soil biota diversity by pesticide residue concentrations (Pesticides), soil properties, climate, ecosystem type together with the shared variance and residuals, for the organism observed ASV/OTU richness and the soil multidiversity index, for associated generalised linear models. This table shows the results when the data of all ecosystem types are analysed (n=373 sites) while the results in Supplementary Table S1 of Supplementary Data File S2 are based on cropland sites (n=244 sites).

**Supplementary Table S2 – Data File S4. For all ecosystem types - Variation partitioning between drivers explaining soil organism Shannon diversity.** Percentages of explained variance of soil biota diversity by pesticide residue concentrations (Pesticides), soil properties, climate, ecosystem type together with the shared variance and residuals, for the organism Shannon diversity, for associated generalised linear models. This table shows the results when the data of all ecosystem types are analysed (n=373 sites) while the results in Supplementary Table S2 of Supplementary Data File S2 are based on cropland sites (n=244 sites).

**Supplementary Table S3 – Data File S4. For all ecosystem types - Variation partitioning between drivers explaining soil functional group relative abundance.** Percentages of explained variance of soil biota diversity by pesticide residue concentrations (Pesticides), soil properties, climate, ecosystem type together with the shared variance and residuals, for the functional group relative abundance, for associated generalised models. This table shows the results when the data of all ecosystem types are analysed (n=373 sites) while the results in Supplementary Table S3 of Supplementary Data File S2 are based on cropland sites (n=244 sites).

**Supplementary Table S4 – Data File S4. For all ecosystem types - Variation partitioning between drivers explaining functional gene groups involved in the C cycle.** Percentages of explained variance of soil biota diversity by pesticide residue concentrations (Pesticides), soil properties, climate, ecosystem type together with the shared variance and residuals, for the functional gene groups involved in the C cycle, for associated generalised linear models. This table shows the results when the data of all ecosystem types are analysed (n=349 sites) while the results in Supplementary Table S4 of Supplementary Data File S2 are based on cropland sites (n=234 sites).

**Supplementary Table S5 – Data File S4. For all ecosystem types - Variation partitioning between drivers explaining functional gene groups involved in the N cycle.** Percentages of explained variance of soil biota diversity by pesticide residue concentrations (Pesticides), soil properties, climate, ecosystem type together with the shared variance and residuals, for the functional gene groups involved in the N cycle, for associated generalised linear models. This table shows the results when the data of all ecosystem types are analysed (n=349 sites) while the results in Supplementary Table S5 of Supplementary Data File S2 are based on cropland sites (n=234 sites).

**Supplementary Table S6 – Data File S4. For all ecosystem types - Variation partitioning between drivers explaining functional gene groups involved in the P cycle.** Percentages of explained variance of soil biota diversity by pesticide residue concentrations (Pesticides), soil properties, climate, ecosystem type together with the shared variance and residuals, for the functional gene groups involved in the P cycle, for associated generalised linear models. This table shows the results when the data of all ecosystem types are analysed (n=349 sites) while the results in Supplementary Table S6 of Supplementary Data File S2 are based on cropland sites (n=234 sites).

**Supplementary Table S7 – Data File S4. For all ecosystem types - Environmental properties feature-selected in the generalised linear models for metabarcoding data.** Soil properties and climatic variables feature-selected next to pesticides and ecosystem type in the models (GLM) using data of all ecosystem types (n=373 sites). This table shows the results when the data of all ecosystem types are analysed while the results in Supplementary Table S7 of Supplementary Data File S2 are based on cropland sites (n=244 sites).

**Supplementary Table S8 – Data File S4. For all ecosystem types - Environmental properties feature-selected in the generalised linear models for the metagenomics data.** Soil properties and climatic variables feature-selected next to pesticides and ecosystem type in the models (GLM) using data of all ecosystem types (n=349 sites). This table shows the results when the data of all ecosystem types are analysed while the results in Supplementary Table S8 of Supplementary Data File S2 are based on cropland sites (n=234 sites).

**Supplementary Table S9 – Data File S4. For all ecosystem types - Highest Pearson (linear) correlations (|r| > 0.5, p-value < 0.05) between initial set of predictors considered in the generalised linear models.**

**Supplementary Table S10 – Data File S4.** **For all ecosystem types - Significant environmental variables in sites where a pesticide residue significantly impacted various soil organism or functional groups** (selected in previously conducted GLM) using data of all ecosystem types (n=373 sites). A p-value is reported from a Kruskal-Wallis test when found significant: * refers to p-value < 0.05; ** to p-value < 0.01; *** to p-value < 0.001. An empty cell refers to a p-value > 0.05. The sum on the last row refers to the number of times an environmental variable significantly differed in presence or absence of a pesticide. Complete p-values and mean values of environmental variables are available in Supplementary Data File S8. This table shows the results when the data of all ecosystem types are analysed while the results in Supplementary Table S10 of Supplementary Data File S2 are based on cropland sites (n=244 sites).

**Supplementary Table S11 – Data File S4. For all ecosystem types - Significant environmental variables in sites where a pesticide residue significantly impacted various functional genes involved in the C, N, P cycles** (selected in previously conducted GLM) using data of all ecosystem types (n=349 sites). A p-value is reported from a Kruskal-Wallis test when found significant: * refers to p-value < 0.05; ** to p-value < 0.01; *** to p-value < 0.001. An empty cell refers to a p-value > 0.05. The sum on the last row refers to the number of times an environmental variable significantly differed in presence or absence of a pesticide. Complete p-values and mean values of environmental variables are available in Supplementary Data File S8. This table shows the results when the data of all ecosystem types are analysed while the results in Supplementary Table S11 of Supplementary Data File S2 are based on cropland sites (n=234 sites).

**Supplementary Table S12 – Data File S4. Number of sites for which the relative abundance of a functional group is positive (>0) across ecosystem types.** Abbreviation AMF designates the arbuscular mycorrhizal fungi, N-fixers the Nitrogen-fixing bacteria. AC refers to annual croplands, PC to permanent croplands, FC to former croplands (recently converted to grasslands), EG to extensive grasslands and WL to woodlands.

**Supplementary Table S13 – Data File S4. List of genes involved in the C, N, P cycles.** These genes are grouped in functional gene groups used for further analyses.

**Supplementary Table S14 – Data File S4. Functional gene groups involved in the carbon (C), nitrogen (N) and phosphorus (P) cycles** (a: archaea, b: bacteria, f: fungi, fa: fauna).

**Supplementary Table S15 – Data File S4. Number of sites for which the diversity of a functional gene group is positive (>0) across ecosystem types.** AC refers to annual croplands, PC to permanent croplands, FC to former croplands (recently converted to grasslands) and EG to extensive grasslands.

**Supplementary Table S16 – Data File S4. For all ecosystem types - Model performance for the generalised linear models including pesticide concentrations and environmental variables in explaining soil organism richness, Shannon diversity and multidiversity**, using data of all ecosystem types (n=373 sites). This table shows the results when the data of all ecosystem types are analysed while the results in Supplementary Table S12 of Supplementary Data File S2 are based on cropland sites (n=244 sites).

**Supplementary Table S17 – Data File S4. For all ecosystem types - Model performance for the generalised linear models including pesticide concentrations and environmental variables in explaining functional group relative abundance**, using data of all ecosystem types (n=373 sites). This table shows the results when the data of all ecosystem types are analysed while the results in Supplementary Table S13 of Supplementary Data File S2 are based on cropland sites (n=244 sites).

**Supplementary Table S18 – Data File S4.** **For all ecosystem types - Model performance for the generalised linear models including pesticide concentrations and environmental variables in explaining functional gene group involved in the C cycle**, using data of all ecosystem types (n=349 sites). This table shows the results when the data of all ecosystem types are analysed while the results in Supplementary Table S14 of Supplementary Data File S2 are based on cropland sites (n=234 sites).

**Supplementary Table S19 – Data File S4. For all ecosystem types - Model performance for the generalised linear models including pesticide concentrations and environmental variables in explaining functional gene group involved in the N cycle**, using data of all ecosystem types (n=349 sites). This table shows the results when the data of all ecosystem types are analysed while the results in Supplementary Table S15 of Supplementary Data File S2 are based on cropland sites (n=234 sites).

**Supplementary Table S20 – Data File S4. For all ecosystem types - Model performance for the generalised linear models including pesticide concentrations and environmental variables in explaining functional gene group involved in the P cycle**, using data of all ecosystem types (n=349 sites). This table shows the results when the data of all ecosystem types are analysed while the results in Supplementary Table S16 of Supplementary Data File S2 are based on cropland sites (n=234 sites).

**Supplementary Data File S5: For croplands only**– Partial plots of the generalised linear models supporting the metabarcoding and metagenomics analyses for croplands only.

**Supplementary Data File S6: For all ecosystem types**– Partial plots of the generalised linear models supporting the metabarcoding and metagenomics analyses across all ecosystem types.

**Supplementary Data File S7:** **For croplands only –**Tables of the differences in environmental variables’ mean values between sites where a pesticide was detected or not, for each pesticide selected as a relevant predictor of soil biodiversity. Complete p-values from a Kruskal-Wallis test and mean values of environmental variables are reported.

**Supplementary Data File S8: For all ecosystem types –**Tables of the differences in environmental variables’ mean values between sites where a pesticide was detected or not, for each pesticide selected as a relevant predictor of soil biodiversity. Complete p-values from a Kruskal-Wallis test and mean values of environmental variables are reported.

**References**

93 Lewis, K. A., Tzilivakis, J., Warner, D. J. & Green, A. An international database for pesticide risk assessments and management. *Human and Ecological Risk Assessment: An International Journal* **22**, 1050-1064 (2016).

94 Forouzesh, A., Zand, E., Soufizadeh, S. & Samadi Foroushani, S. Classification of herbicides according to chemical family for weed resistance management strategies–an update. *Weed Research* **55**, 334-358 (2015).

95 Hermann, D. & Stenzel, K. FRAC mode‐of‐action classification and resistance risk of fungicides. *Modern crop protection compounds* **2**, 589-608 (2019).

96 Sparks, T. C. & Nauen, R. IRAC: Mode of action classification and insecticide resistance management. *Pesticide biochemistry and physiology* **121**, 122-128 (2015).

97 Vieira, D., Franco, A., De Medici, D., Martin Jimanez, J., Wojda P., Jones, A. Pesticides residues in European agricultural soils - Results from LUCAS 2018 soil module. (European Commission, 2023).

98 Directive 2009/128/EC of the European Parliament and of the Council of 21 October 2009 Establishing a Framework for Community Action to Achieve the Sustainable Use of Pesticides, OJL 309, 24.11.2009, p. 71–86.

99 European Commission. Database active substances. (<https://ec.europa.eu/food/plant/pesticides/eu-pesticides-database/start/screen/active-substances>, 2023).

100 Legendre, P., Legendre, L. in *Numerical Ecology,* (Elsevier, 2012)

101 Wirsching, J. *et al.* Temperature and soil moisture change microbial allocation of pesticide‐derived carbon. (2023).

102 Franco, A., Vieira, D., Clerbaux, L-A., Orgiazzi, A., Labouyrie M., Köninger, J., Silva, V., van Dam, R, Carnesecchi, E.,  Dorne J-J. CM, Vuaille, J., Vicente, J. L., Jones, A. Evaluation of the ecological risk of pesticides residues from the  European LUCAS soil monitoring 2018 survey. *Integrated Environmental Assessment and Management* (2024).

103 Wang, C.-N. *et al.* Effects of pesticide residues on bacterial community diversity and structure in typical greenhouse soils with increasing cultivation years in Northern China. *Science of the Total Environment* **710**, 136321 (2020).

104 Cabrera, L. C., Di Piazza, G., Dujardin, B., Marchese, E. & Pastor, P. M. The 2022 Europea Union report on pesticide residues in food. *Efsa Journal* **22** (2024).

105 Eurostat. Agri-environmental indicator - consumption of pesticides. (European Commission,, 2024).

106 Tincani, F. *et al.* Applying a tiered environmental risk assessment framework to estimate the risk of pesticides to soil organisms in Latin America. *Integrated Environmental Assessment and Management* **19**, 446-460 (2023).

107 Ruuskanen, S. *et al.* Ecosystem consequences of herbicides: the role of microbiome. *Trends in ecology & evolution* **38**, 35-43 (2023).

108 Hagner, M., Mikola, J., Saloniemi, I., Saikkonen, K. & Helander, M. Effects of a glyphosate-based herbicide on soil animal trophic groups and associated ecosystem functioning in a northern agricultural field. *Scientific reports* **9**, 8540 (2019).

109 EFSA Panel, C. *et al.* Scientific opinion addressing the state of the science on risk assessment of plant protection products for in‐soil organisms. *Efsa Journal* **15**, e04690 (2017).

110 Liao, H. et al. Herbicide selection promotes antibiotic resistance in soil microbiomes. 615 Molecular Biology and Evolution 38, 2337-2350 (2021).

111 Guinet, M. *et al.* Fostering temporal crop diversification to reduce pesticide use. *Nature Communications* **14**, 7416 (2023).

112 Angioni, A., Porcu, L. & Dedola, F. Determination of famoxadone, fenamidone, fenhexamid and iprodione residues in greenhouse tomatoes. *Pest Management Science* **68**, 543-547 (2012).

113 Bojacá, C. R., Arias, L. A., Ahumada, D. A., Casilimas, H. A. & Schrevens, E. Evaluation of pesticide residues in open field and greenhouse tomatoes from Colombia. *Food control* **30**, 400-403 (2013).

114 Liu, L., Zheng, X., Wei, X., Kai, Z. & Xu, Y. Excessive application of chemical fertilizer and organophosphorus pesticides induced total phosphorus loss from planting causing surface water eutrophication. *Scientific Reports* **11**, 23015 (2021).

115 Zhao, F. *et al.* Reducing risks of antibiotics to crop production requires land system intensification within thresholds. *Nature Communications* **14**, 6094 (2023).

116 Summerton, L., Greener, M., Patterson, D. & Brown, C. D. Effects of soil redistribution by tillage on subsequent transport of pesticide to subsurface drains. *Pest management science* **79**, 616-626 (2023).

117 Mackay, J. E., Bernhardt, L. T., Smith, R. G. & Ernakovich, J. G. Tillage and pesticide seed treatments have distinct effects on soil microbial diversity and function. *Soil Biology and Biochemistry* **176**, 108860 (2023).

118 Vischetti, C. *et al.* Sub-lethal effects of pesticides on the DNA of soil organisms as early ecotoxicological biomarkers. *Frontiers in Microbiology* **11**, 1892 (2020).

119 Lilja, M. A. *et al.* Comparing earthworm biodiversity estimated by DNA metabarcoding and morphology-based approaches. *Applied Soil Ecology* **185**, 104798 (2023).

120 Louca, S., Parfrey, L. W. & Doebeli, M. Decoupling function and taxonomy in the global ocean microbiome. *Science* **353**, 1272–1277 (2016).

121 Sansupa, C. et al. Can We Use Functional Annotation of Prokaryotic Taxa (FAPROTAX) to Assign the Ecological Functions of Soil Bacteria? *Appl. Sci.* **11**, 688 (2021).

122 Aslani, F., Geisen, S., Ning, D., Tedersoo, L. & Bahram, M. Towards revealing the global diversity and community assembly of soil eukaryotes. *Ecology letters* **25**, 65-76 (2022).

123 Labouyrie, M., Ballabio C., Romero F., Panagos P., Jones, A., Schmid, M.W., Mikryukov V., Dulya O., Tedersoo L., Bahram, M., Lugato, E., van der Heijden, M.G-A., Orgiazzi, A. Patterns in soil microbial diversity across Europe. *Nature Communications* (2023).

124 Köninger, J., Ballabio, C., Panagos, P., Jones, A., Schmid, M.W., Orgiazzi, A., Briones, M.J.I. Ecosystem type drives soil eukaryotic diversity and composition in Europe. *Global change biology*, 1-14 (2023).

125 Carini, P. *et al.* Relic DNA is abundant in soil and obscures estimates of soil microbial diversity. *Nature microbiology* **2**, 1-6 (2016).

126 Nagler, M., Podmirseg, S. M., Ascher‐Jenull, J., Sint, D. & Traugott, M. Why eDNA fractions need consideration in biomonitoring. *Molecular Ecology Resources* **22**, 2458-2470 (2022).

1. a While Dimethenamid was already banned on 23 May 2006, the isomer Dimethenamid-P is still approved on the market. Our analytical method does not allow to differentiate between Dimethenamid and Dimethenamid-P. [↑](#footnote-ref-1)
2. b While Metolachlor was already banned previously, the isomer S-Metolachlor was banned 22 January 2024 only. Our analytical method does not allow to differentiate between Metolachlor and S-Metolachlor. [↑](#footnote-ref-2)
