# Supplementary material for: Pesticide residues alter taxonomic and functional biodiversity in soils
Source: Nature. 2026 Jan 28;650(8101):367–73. doi: 10.1038/s41586-025-09991-z (PMC12965876; doi:10.1038/s41586-025-09991-z)
Supplement: Supplementary file 2 — Reporting Summary [file 41586_2025_9991_MOESM2_ESM.pdf]

Corresponding author(s): Marcel van der Heijden, Maria Briones, Alberto Orgiazzi

Last updated by author(s): Jan 23, 2026

Reporting Summary

Nature Portfolio wishes to improve the reproducibility of the work that we publish. This form provides structure for consistency and transparency in reporting. For further information on Nature Portfolio policies, see our Editorial Policies and the Editorial Policy Checklist.

Statistics

For all statistical analyses, confirm that the following items are present in the figure legend, table legend, main text, or Methods section.

n/a	Confirmed	
☐	☒	The exact sample size (*n*) for each experimental group/condition, given as a discrete number and unit of measurement
☐	☒	A statement on whether measurements were taken from distinct samples or whether the same sample was measured repeatedly
☐	☒	The statistical test(s) used AND whether they are one- or two-sided<br>*Only common tests should be described solely by name; describe more complex techniques in the Methods section.*
☐	☒	A description of all covariates tested
☐	☒	A description of any assumptions or corrections, such as tests of normality and adjustment for multiple comparisons
☐	☒	A full description of the statistical parameters including central tendency (e.g. means) or other basic estimates (e.g. regression coefficient) AND variation (e.g. standard deviation) or associated estimates of uncertainty (e.g. confidence intervals)
☐	☒	For null hypothesis testing, the test statistic (e.g. *F*, *t*, *r*) with confidence intervals, effect sizes, degrees of freedom and *P* value noted<br>*Give P values as exact values whenever suitable.*
☒	☐	For Bayesian analysis, information on the choice of priors and Markov chain Monte Carlo settings
☒	☐	For hierarchical and complex designs, identification of the appropriate level for tests and full reporting of outcomes
☒	☐	Estimates of effect sizes (e.g. Cohen's *d*, Pearson's *r*), indicating how they were calculated

Our web collection on statistics for biologists contains articles on many of the points above.

Software and code

Policy information about availability of computer code

Data collection

We used Rstudio (version 2021.09.0) and R (version 4.2.1).

Data analysis

We used Rstudio (version 2021.09.0) and R (version 4.2.1). All R scripts relating pesticide analysis to soil biodiversity are available from European Soil Data Centre - ESDAC (https://esdac.jrc.ec.europa.eu/content/pesticides-and-soil-biodiversity).

The R packages needed are the following:
#library(BiocManager) v1.30.18
#library(devtools) v2.4.3
library(SRS) v0.2.3
library(microeco) v0.15.0
library(car) v3.0.12
library(caret) v6.0.94
library(corrplot) v0.92
library(cowplot) v1.1.3
library(dplyr) v1.0.8
library(geoR) v1.9.2
library(ggord) v1.1.5 – instructions of installation here: https://fawda123.github.io/ggord/
library(ggordiplots) v0.4.1 – instructions of installation here: https://rdrr.io/github/jfq3/ggordiplots/
library(ggplot2) v3.3.5
library(ggpmisc) v0.5.5
library(ggpol) v0.0.7

```
library(ggpubr) v0.6.0
library(ggrepel) v0.9.5
library(magrittr) v2.0.3
library(multcompView) v0.1.8
library(patchwork) v1.2.0
library(plyr) v1.8.7
library(readxl) v1.4.0
library(rnaturalearth) v1.0.1
library(rnaturalearthdata) v1.0.0
library(sf) v1.0.7
library(stringr) v1.4.0
library(tibble) v3.2.1
library(tidyr) v1.2.0
library(vegan) v2.5.7
library(DHARMa) v0.4.7
library(Metrics) v0.1.4
library(ggraph) v2.2.1
library(visNetwork) v2.1.2
library(stats) v4.5.0
```

For manuscripts utilizing custom algorithms or software that are central to the research but not yet described in published literature, software must be made available to editors and reviewers. We strongly encourage code deposition in a community repository (e.g. GitHub). See the Nature Portfolio guidelines for submitting code & software for further information.

Data

Policy information about availability of data

All manuscripts must include a data availability statement. This statement should provide the following information, where applicable:

- Accession codes, unique identifiers, or web links for publicly available datasets
- A description of any restrictions on data availability
- For clinical datasets or third party data, please ensure that the statement adheres to our policy

Pesticide data supporting this study are available from European Soil Data Centre (ESDAC) (https://esdac.jrc.ec.europa.eu/content/pesticides-and-soil-biodiversity), subject to registration and a data sharing agreement, owing to the confidential nature of the measurements. The Pesticide Properties Database is accessible at https://sitem.herts.ac.uk/aeru/ppdb/. The database from the Herbicide Resistance Action Committee (HRAC) is accessible at https://hracglobal.com/files/2024-HRAC-GLOBAL-HERBICIDE-MOA-CLASSIFICATION-POSTERold.pdf, the database from the Fungicide Resistance Action Committee (FRAC) is accessible at https://www.frac.info/fungicide-resistance-management/by-frac-mode-of-action-group/#open-tour, and the one from Insecticide Resistance Action Committee (IRAC) is accessible at https://irac-online.org/mode-of-action/. The raw data (DNA sequences) generated in this study have been deposited in the Sequence Read Archive (SRA) database under BioProject ID PRJNA1118194 for archaeal 16S data, BioProject ID PRJNA952168 for bacterial 16S and fungal ITS data, BioProject ID PRJNA985135 for eukaryotic 18S data and BioProject ID PRJNA1032917 for metagenomic data. The Global database of soil nematodes is available at https://github.com/hooge104/2020_global_nematode_dataset/blob/master/data/nematode_full_dataset_wBiome.csv. The sampling site environmental metadata used in this study are available from ESDAC (https://esdac.jrc.ec.europa.eu/content/soil-biodiversity-dna-eukaryotes).

Human research participants

Policy information about studies involving human research participants and Sex and Gender in Research.

Reporting on sex and gender	N/A
Population characteristics	N/A
Recruitment	N/A
Ethics oversight	N/A

Note that full information on the approval of the study protocol must also be provided in the manuscript.

Field-specific reporting

Please select the one below that is the best fit for your research. If you are not sure, read the appropriate sections before making your selection.

☐ Life sciences ☐ Behavioural & social sciences ☒ Ecological, evolutionary & environmental sciences

For a reference copy of the document with all sections, see nature.com/documents/nr-reporting-summary-flat.pdf

Ecological, evolutionary & environmental sciences study design

All studies must disclose on these points even when the disclosure is negative.

Study description	We investigated the impact of pesticide concentrations on soil communities of archaea, bacteria, fungi, protists, nematodes and arthropods across Europe. Nine functional groups were also investigated: archaeal nitrifiers, bacterial chemoheterotrophs, bacterial Nitrogen-fixers (N-fixers), arbuscular mycorrhizal fungi (AMF), fungal plant pathogens, (animal and plant) parasitic protists, and bacterivore and herbivore nematodes, as well as the diversity of 48 functional gene groups involved in the carbon (C), nitrogen (N) and phosphorus (P) cycles, derived from archaeal, bacterial, fungal and faunal metagenomes. We explored the effect of pesticides on the richness and diversity of the organisms, their combined diversity (using a multidiversity index), the relative abundance of the functional groups, and the diversity of each of the functional gene groups, in five ecosystem types spanning annual croplands, permanent croplands, former croplands recently converted to grasslands, extensive grasslands and woodlands.
Research sample	We analysed pesticide concentrations and archaeal, bacterial, eukaryotic (fungi, protists, nematodes, arthropods) DNA sequences from 373 soil samples collected all over Europe as part of LUCAS (Land Use/Cover Area frame Survey), the largest European soil survey coordinated by the European Commission. We also analysed metagenomes of archaea, bacteria, fungi and fauna in 349 soil samples.
Sampling strategy	Sampling points were selected based on their overlapping between LUCAS Soil Pesticides and LUCAS Soil Biodiversity modules, both presented in Orgiazzi, A. et al. LUCAS Soil Biodiversity and LUCAS Soil Pesticides, new tools for research and policy development. European Journal of Soil Science 73, e13299 (2022).
Data collection	At each location, five subsamples covering a depth of 20 cm were collected and mixed together. One subsample was collected at the precise geographical location of the pre-selected point while four additional subsamples were collected at the four cardinal directions (North, East, South and West), at a distance of 2m from the first subsample location in each direction. Data collection was performed by trained surveyors. Surveyor instructions for data collection are presented in Fernández-Ugalde O., Orgiazzi A., Jones A., Lugato E., Panagos P., LUCAS 2018 – SOIL COMPONENT: Sampling Instructions for Surveyors, EUR 28501 EN, doi 10.2760/023673 https://esdac.jrc.ec.europa.eu/public_path/shared_folder/doc_pub/JRC105923_LUCAS2018_JRCTechnicalReport.pdf
Timing and spatial scale	Data collection took place across Europe from April to October 2018. The final 373 selected soil samples were part of 26 European countries.
Data exclusions	Certain eukaryotic groups (e.g. rotifers, tardigrades and annelids) were excluded due to the small soil volume collected that prevents robust analyses of macrofauna and the limitations of reference databases.
Reproducibility	To date, there is no repeat of the experiment, but a LUCAS biodiversity campaign has been performed in 2022 and will allow in the future to verify the patterns found for 2018. Analysis and results for the latest campaign will be available in 2025-2026.
Randomization	Samples were characterized by their meta-information (e.g., ecosystem type) but not allocated into further groups for the analyses.
Blinding	*Describe the extent of blinding used during data acquisition and analysis. If blinding was not possible, describe why OR explain why blinding was not relevant to your study.*

Did the study involve field work? ☒ Yes ☐ No

Field work, collection and transport

Field conditions	Soil samples were collected from April to October 2018. A full list of metadata (e.g., date, coordinates) were collected. Climatic conditions were accounted for into the models.
Location	Soils samples were collected across European Union and United Kingdom. Sampling locations extended from Sweden to Cyprus (latitudinal gradient) and from Portugal to Cyprus (longitudinal gradient).
Access & import/export	Samples were collected following national rules. In some cases (private land) owners were duly informed before collection. No permission was sought for land in public ownership.
Disturbance	No disturbance were implied.

Reporting for specific materials, systems and methods

We require information from authors about some types of materials, experimental systems and methods used in many studies. Here, indicate whether each material, system or method listed is relevant to your study. If you are not sure if a list item applies to your research, read the appropriate section before selecting a response.

Materials & experimental systems

n/a	Involved in the study
☒	☐ Antibodies
☒	☐ Eukaryotic cell lines
☒	☐ Palaeontology and archaeology
☒	☐ Animals and other organisms
☒	☐ Clinical data
☒	☐ Dual use research of concern

Methods

n/a	Involved in the study
☒	☐ ChIP-seq
☒	☐ Flow cytometry
☒	☐ MRI-based neuroimaging
