# Supplementary material for: Pesticide residues alter taxonomic and functional biodiversity in soils
Source: Nature. 2026 Jan 28;650(8101):367–73. doi: 10.1038/s41586-025-09991-z (PMC12965876; doi:10.1038/s41586-025-09991-z)
Supplement: Supplementary file 5 — Figures for all ecosystem types. See main Supplementary Information file for further description. [file 41586_2025_9991_MOESM5_ESM.pdf]

ALL ECOSYSTEM TYPES

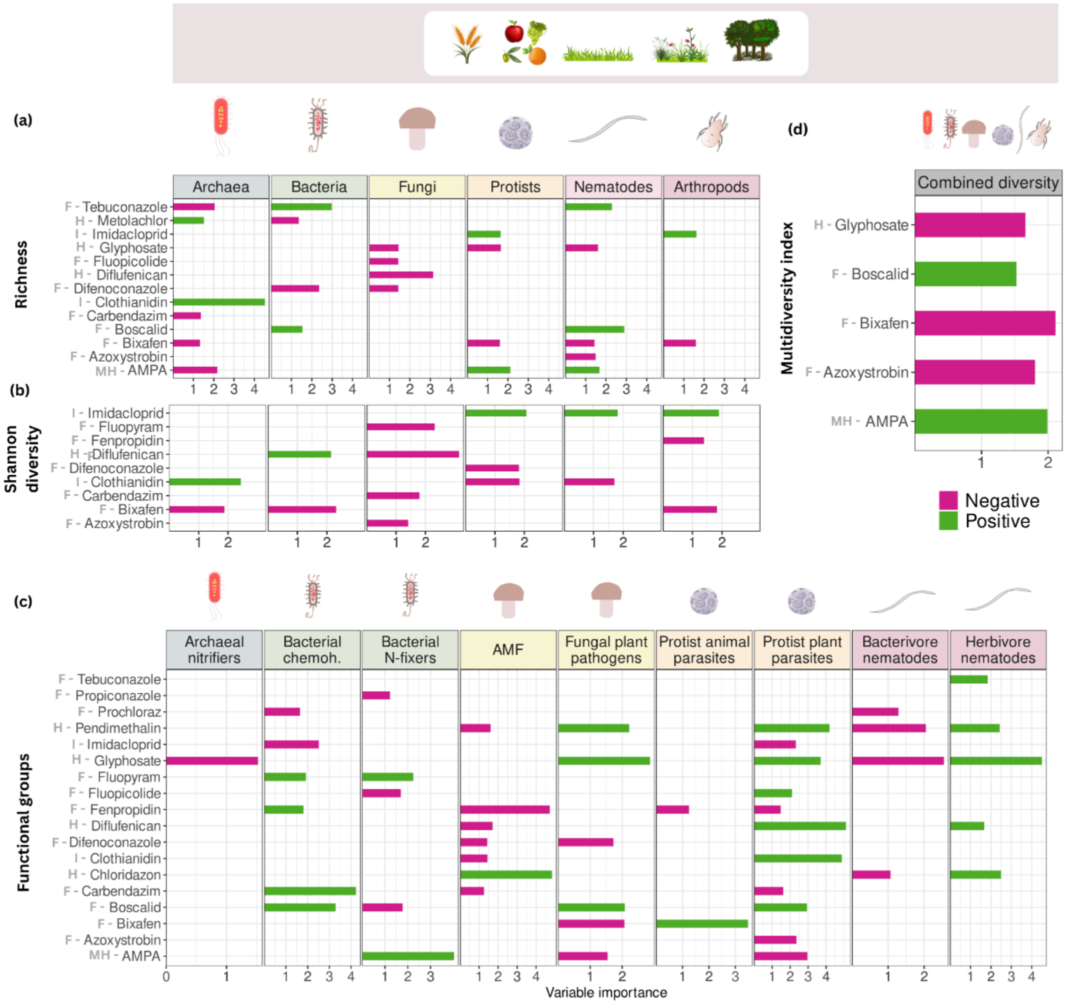

Supplementary Fig. S1. For all ecosystem types – Soil biodiversity (assessed by metabarcoding) responses to key pesticide concentrations (herbicides H, metabolite of a herbicide MH, fungicides F or insecticides I). Positive or negative relationship of concentration of pesticides selected in the GLMs with **(a)** soil organism observed richness, **(b)** Shannon diversity, **(c)** functional group relative abundance and **(d)** multidiversity. Horizontal bars correspond to the variable importance (VIP) coloured in green (positive relationship) or purple (negative), according to the coefficient sign of each pesticide in the associated GLM. Variable importance was calculated based on the GLM including pesticide concentrations, soil properties, climate, and ecosystem type information. The correlation matrix for all environmental and pesticide predictors is available in Supplementary Fig. S3 below. These analyses focus on all ecosystem types together (n=373 sites), while analyses for croplands only (n=244 sites) are presented in Extended Data Fig. S5.

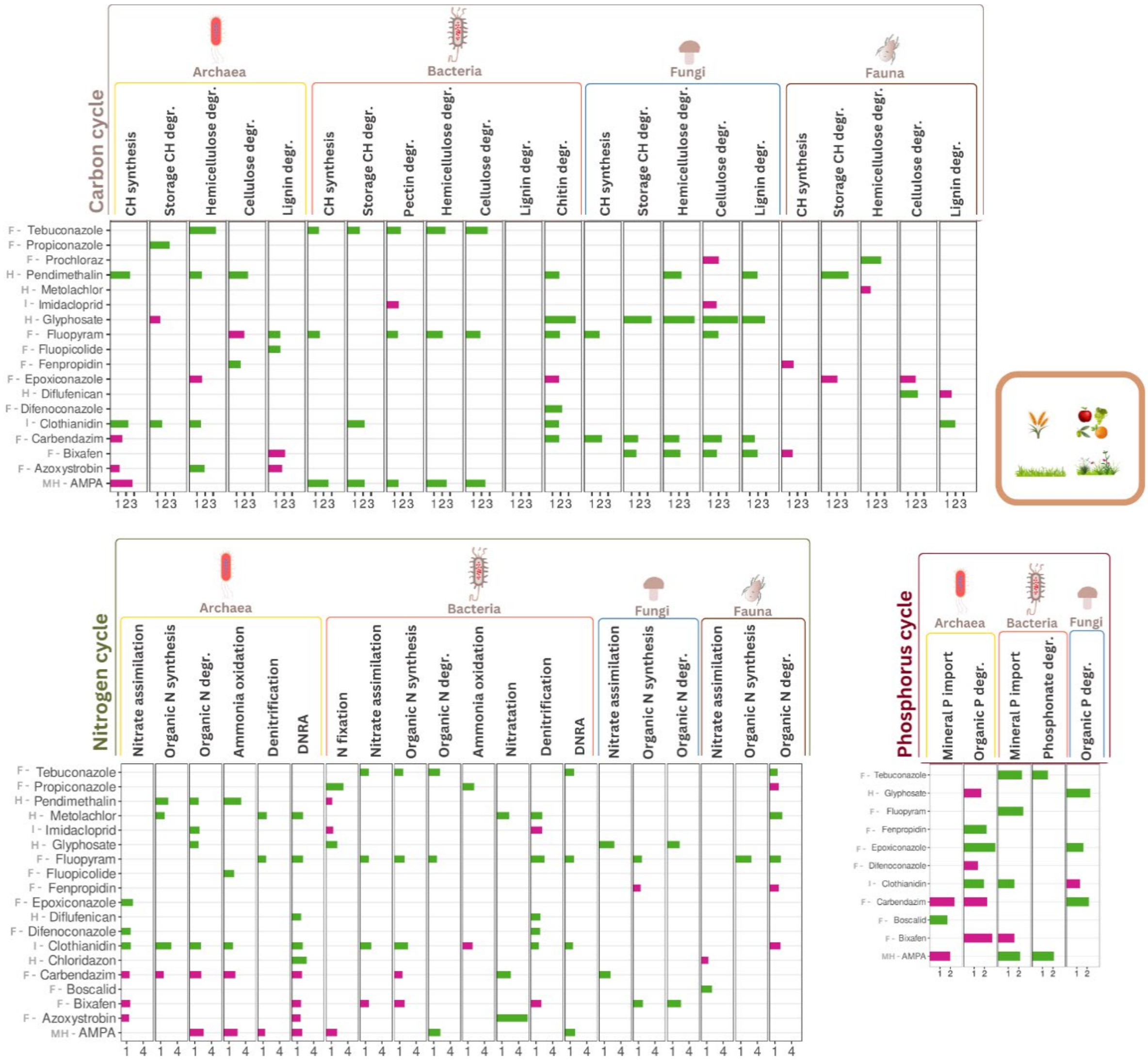

Supplementary Fig. S2. For all ecosystem types – Soil C, N, P functional gene groups responses to key pesticide concentrations (herbicides H, metabolite of a herbicide MH, fungicides F or insecticides I). Positive or negative relationship of concentration of pesticides retained in the GLMs with the diversity of each functional gene group involved in the C, N, and P cycles. Horizontal bars correspond to the variable importance (VIP) coloured in green (positive relationship) or purple (negative), according to the coefficient sign of each pesticide in the associated GLM. Variable importance was calculated based on the GLM including pesticide concentrations, soil properties, climate, and ecosystem type information. These analyses focus on all ecosystem types together (croplands together with grasslands, n=349 sites), while analyses for croplands only (n=234 sites) are presented in Extended Data Fig. S6.

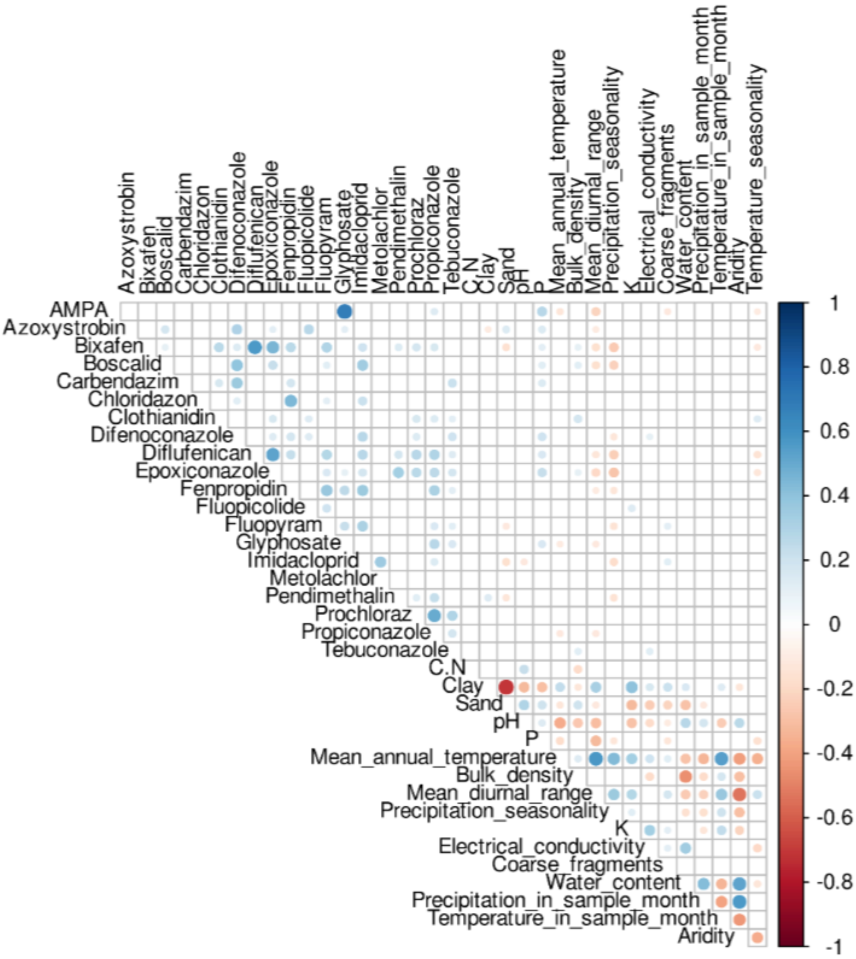

Supplementary Fig. S3. For all ecosystem types – Linear (Pearson) correlations between the initial set of predictors (i.e., before feature-selection) used in the generalised linear models from analyses performed on all ecosystem types (n=373 sites), i.e., all environmental variables next to the most occurring pesticides across all ecosystem types (20 pesticides out of 63, all other pesticides with near-zero variance are not kept). The highest correlations are displayed in Supplementary Table S9 of Supplementary Data File S4. A similar correlation matrix for croplands only (n=244 sites) is displayed in Extended Data Fig. S7 and highest correlations displayed in Supplementary Table S9 of Supplementary Data File S2.

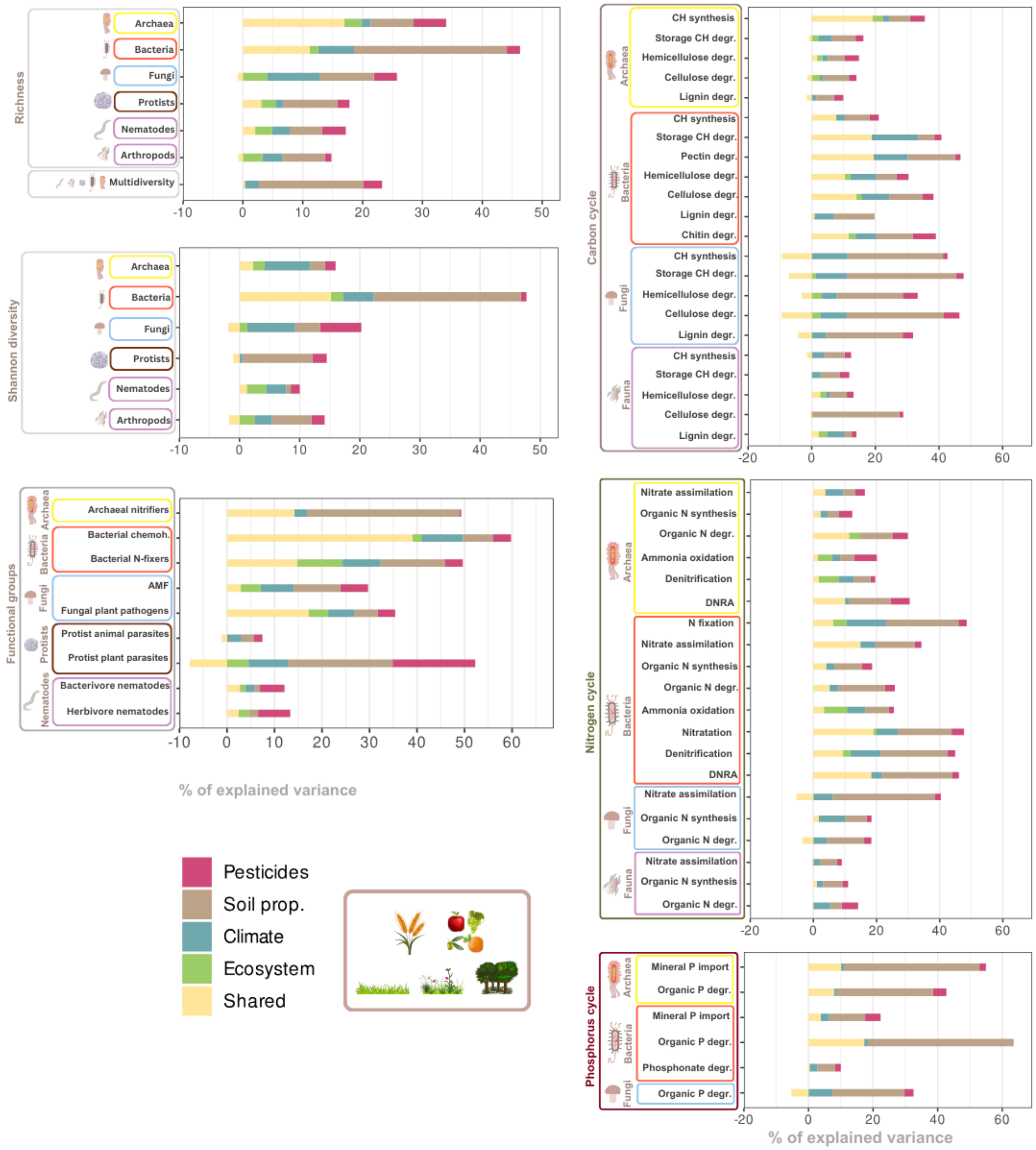

Supplementary Fig. S4. For all ecosystem types – Explained variance (in %) of soil biodiversity by selected variables: pesticide residue concentrations (red), soil properties (brown), climate (blue), ecosystem type (green) together with the shared variance (yellow) for the organism observed ASV/OTU richness, Shannon diversity, multidiversity index, each functional group relative abundance and each functional gene group diversity involved in the C, N, P cycles, using data of all ecosystem types (n=373 sites for metabarcoding analyses, n=349 sites including croplands and grasslands for metagenomics analyses). See Supplementary Fig. S1 and S2 above for the detailed pesticide concentrations and Supplementary Tables S7-S8 of Supplementary Data File S4 for the selected soil properties and climatic variables retained per GLM. This figure shows the results when the data of all ecosystem types are analysed while the results in Fig. 4 and Extended Data Fig. S8 are based on cropland sites (n=244 sites for metabarcoding analyses, n=234 for metagenomics analyses).
