# Supplementary material for: Pesticide residues alter taxonomic and functional biodiversity in soils
Source: Nature. 2026 Jan 28;650(8101):367–73. doi: 10.1038/s41586-025-09991-z (PMC12965876; doi:10.1038/s41586-025-09991-z)

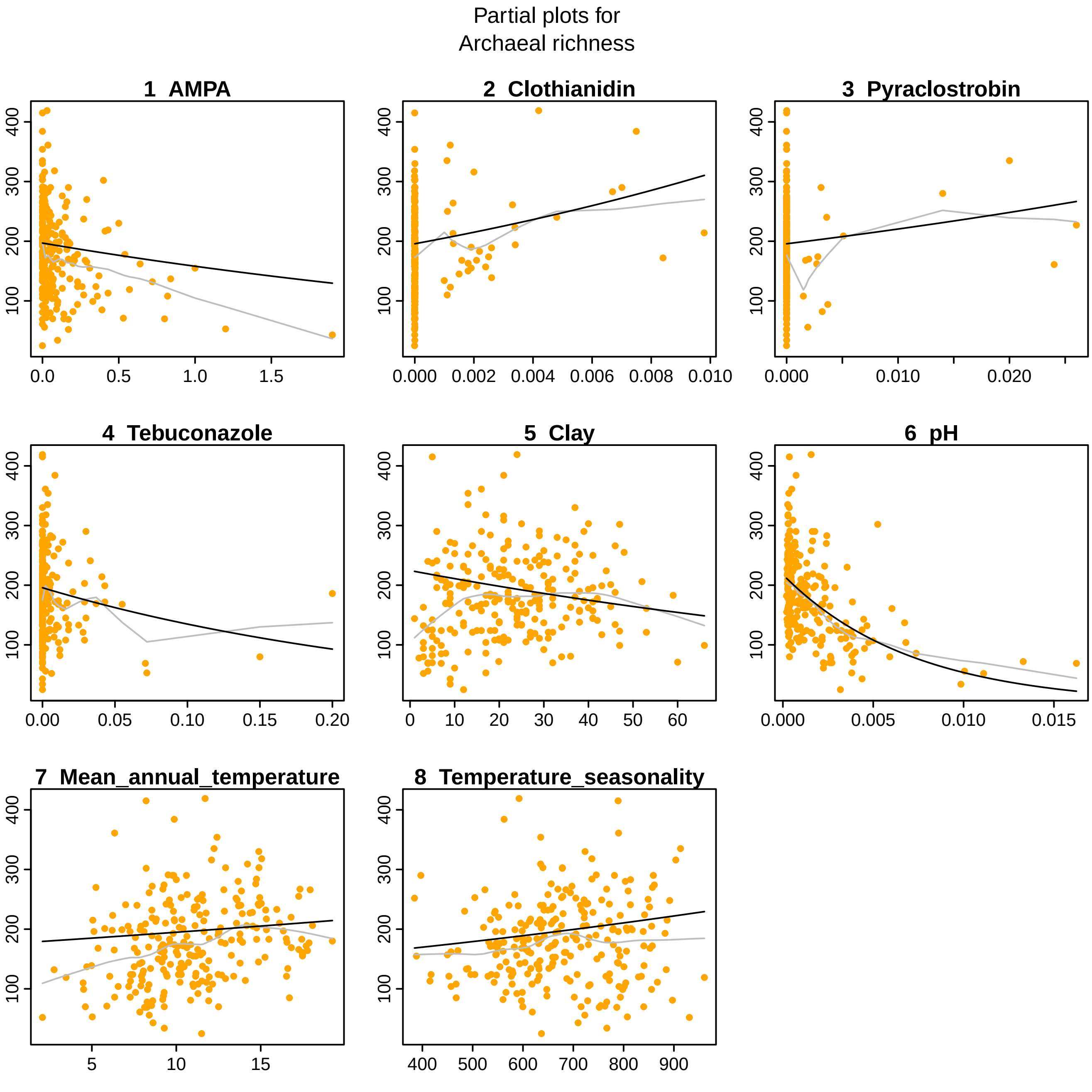
Partial plots for
Archaeal richness
1 AMPA
2 Clothianidin
3 Pyraclostrobin
4 Tebuconazole
5 Clay
6 pH
7 Mean_annual_temperature
8 Temperature_seasonality

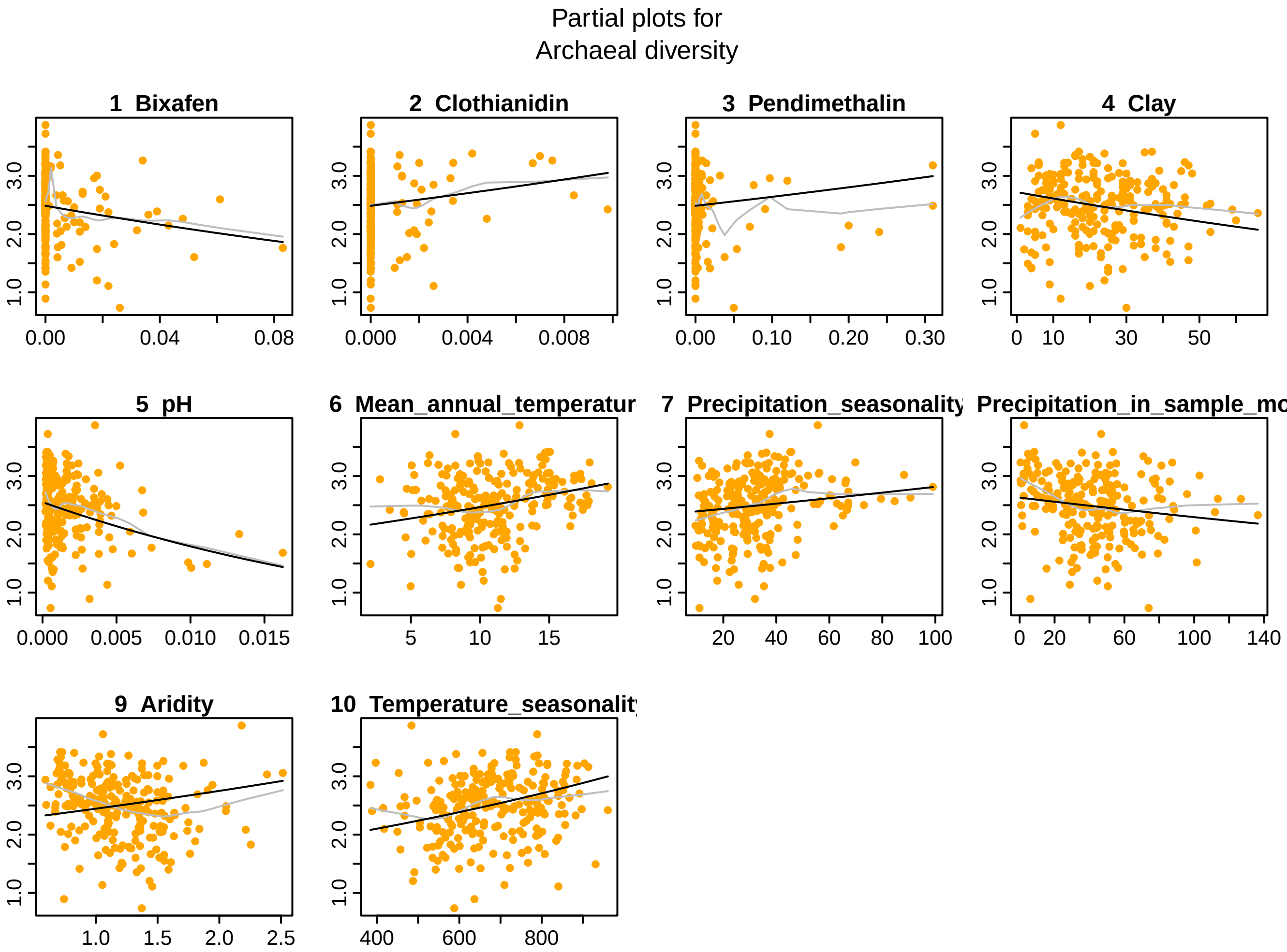
Partial plots for
Archaeal diversity
1 Bixafen
2 Clothianidin
3 Pendimethalin
4 Clay
5 pH
6 Mean_annual_temperatur
7 Precipitation_seasonality
Precipitation_in_sample_mo
9 Aridity
10 Temperature_seasonalit
1.0
2.0
3.0
0.00
0.04
0.08
0.000
0.004
0.008
0.10
0.20
0.30
0
10
30
50
0.005
0.010
0.015
5
15
20
40
60
80
100
140
1.5
2.5
400
600
800

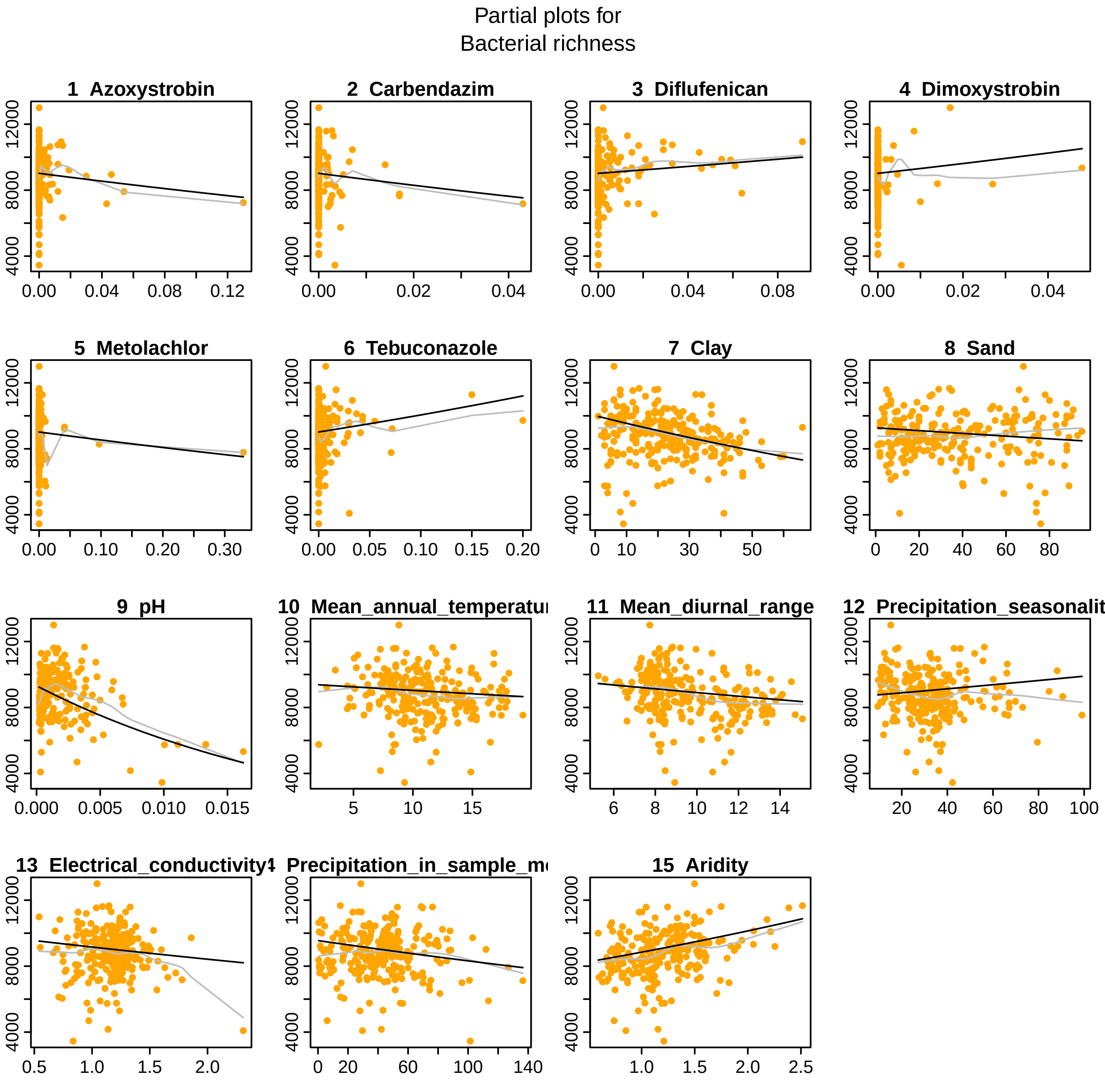
Partial plots for
Bacterial richness
1 Azoxystrobin
2 Carbendazim
3 Diflufenican
4 Dimoxystrobin
5 Metolachlor
6 Tebuconazole
7 Clay
8 Sand
9 pH
10 Mean_annual_temperatu
11 Mean_diurnal_range
12 Precipitation_seasonalit
13 Electrical_conductivity
14 Precipitation_in_sample_m
15 Aridity

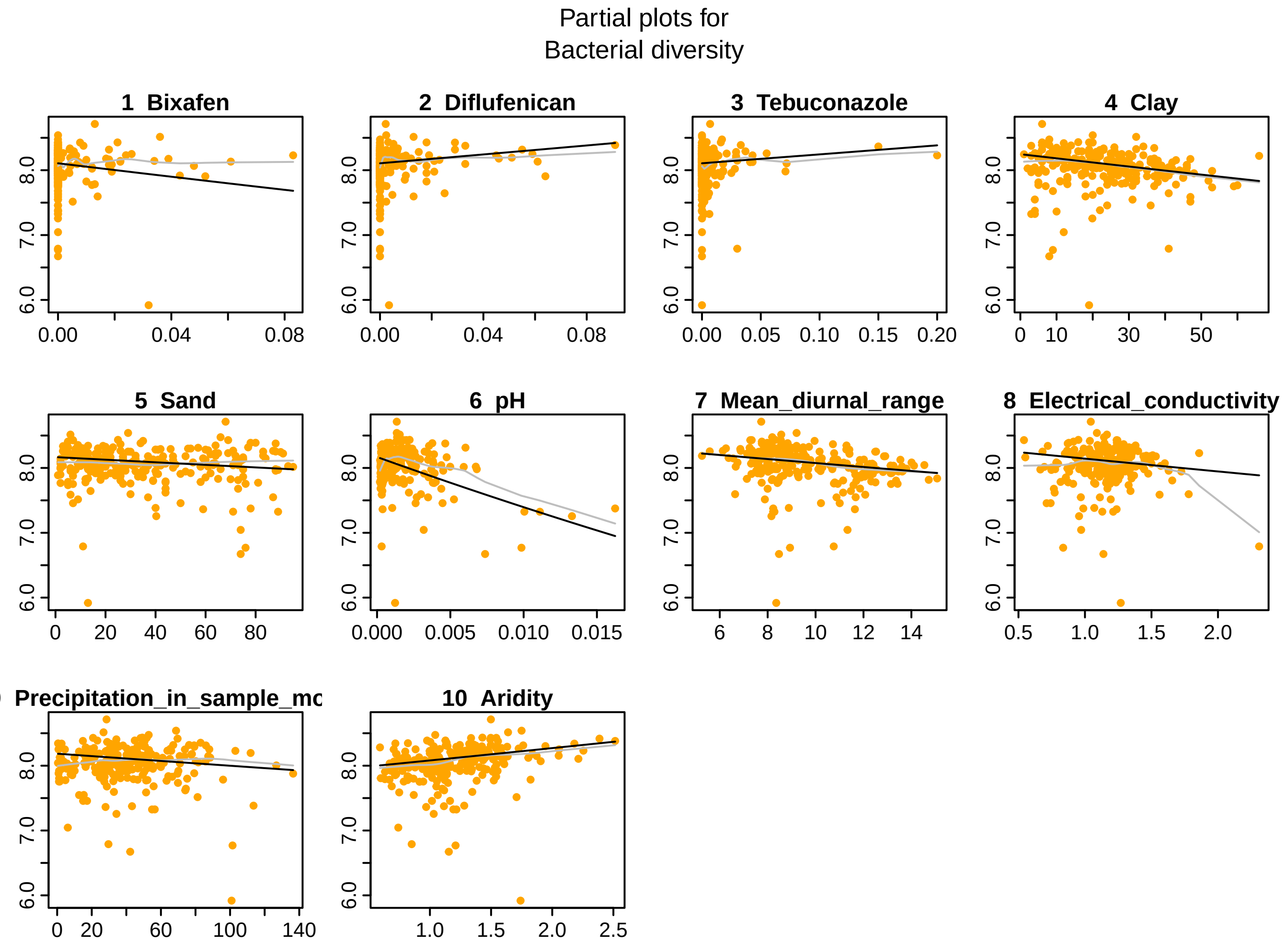

Partial plots for
Bacterial diversity
1 Bixafen
2 Diflufenican
3 Tebuconazole
4 Clay
5 Sand
6 pH
7 Mean_diurnal_range
8 Electrical_conductivity
Precipitation_in_sample_mo
10 Aridity

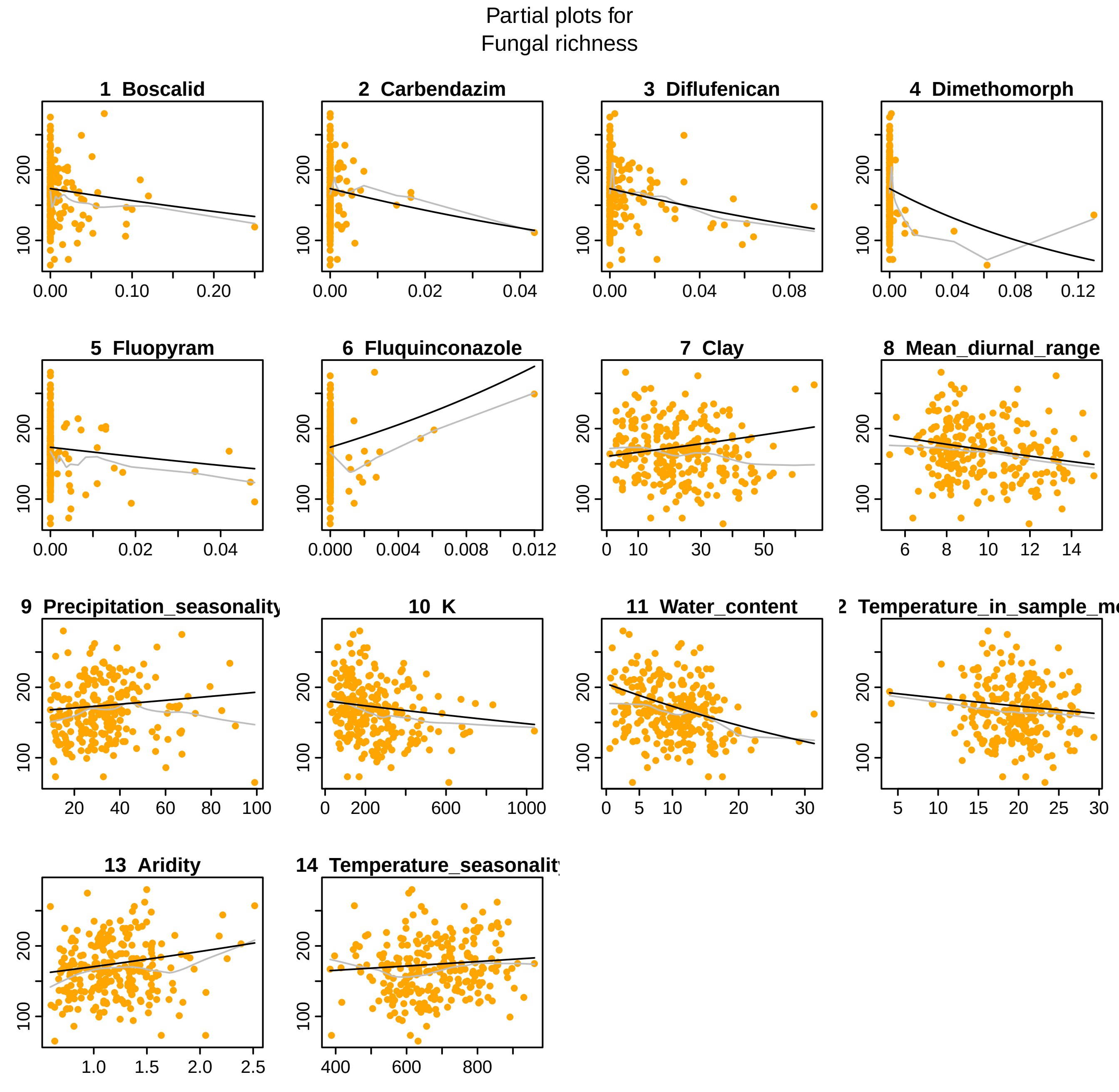

Partial plots for
Fungal richness
1 Boscalid
2 Carbendazim
3 Diflufenican
4 Dimethomorph
5 Fluopyram
6 Fluquinconazole
7 Clay
8 Mean_diurnal_range
9 Precipitation_seasonality
10 K
11 Water_content
Temperature_in_sample_m
13 Aridity
14 Temperature_seasonalit

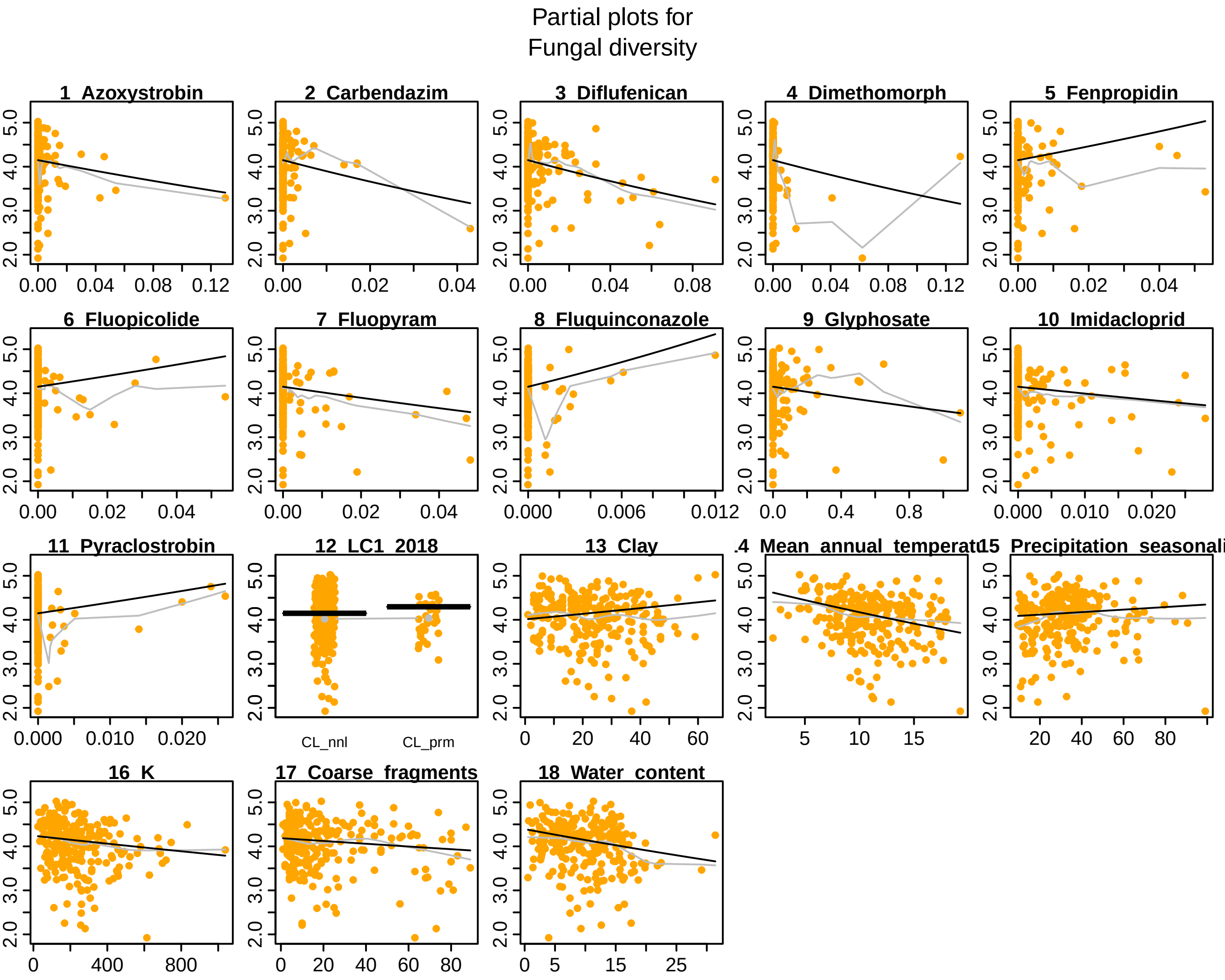

Partial plots for
Fungal diversity
1 Azoxystrobin
2 Carbendazim
3 Diflufenican
4 Dimethomorph
5 Fenpropidin
6 Fluopicolide
7 Fluopyram
8 Fluquinconazole
9 Glyphosate
10 Imidacloprid
11 Pyraclostrobin
12 LC1 2018
13 Clay
14 Mean annual temperatu
15 Precipitation seasonali
16 K
17 Coarse fragments
18 Water content
CL_nnl
CL_prm

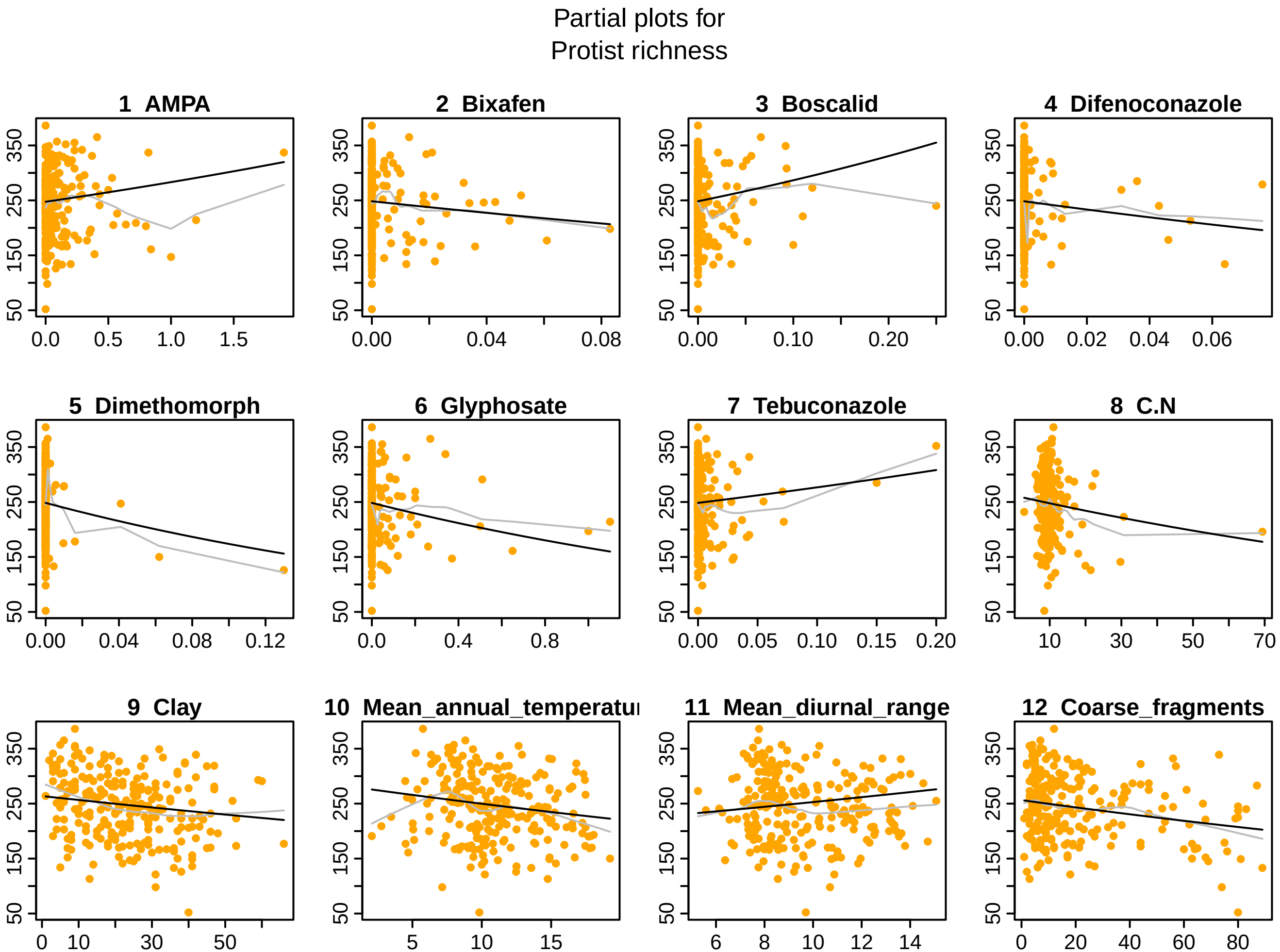
Partial plots for
Protist richness
1 AMPA
2 Bixafen
3 Boscalid
4 Difenoconazole
5 Dimethomorph
6 Glyphosate
7 Tebuconazole
8 C.N
9 Clay
10 Mean_annual_temperatu
11 Mean_diurnal_range
12 Coarse_fragments

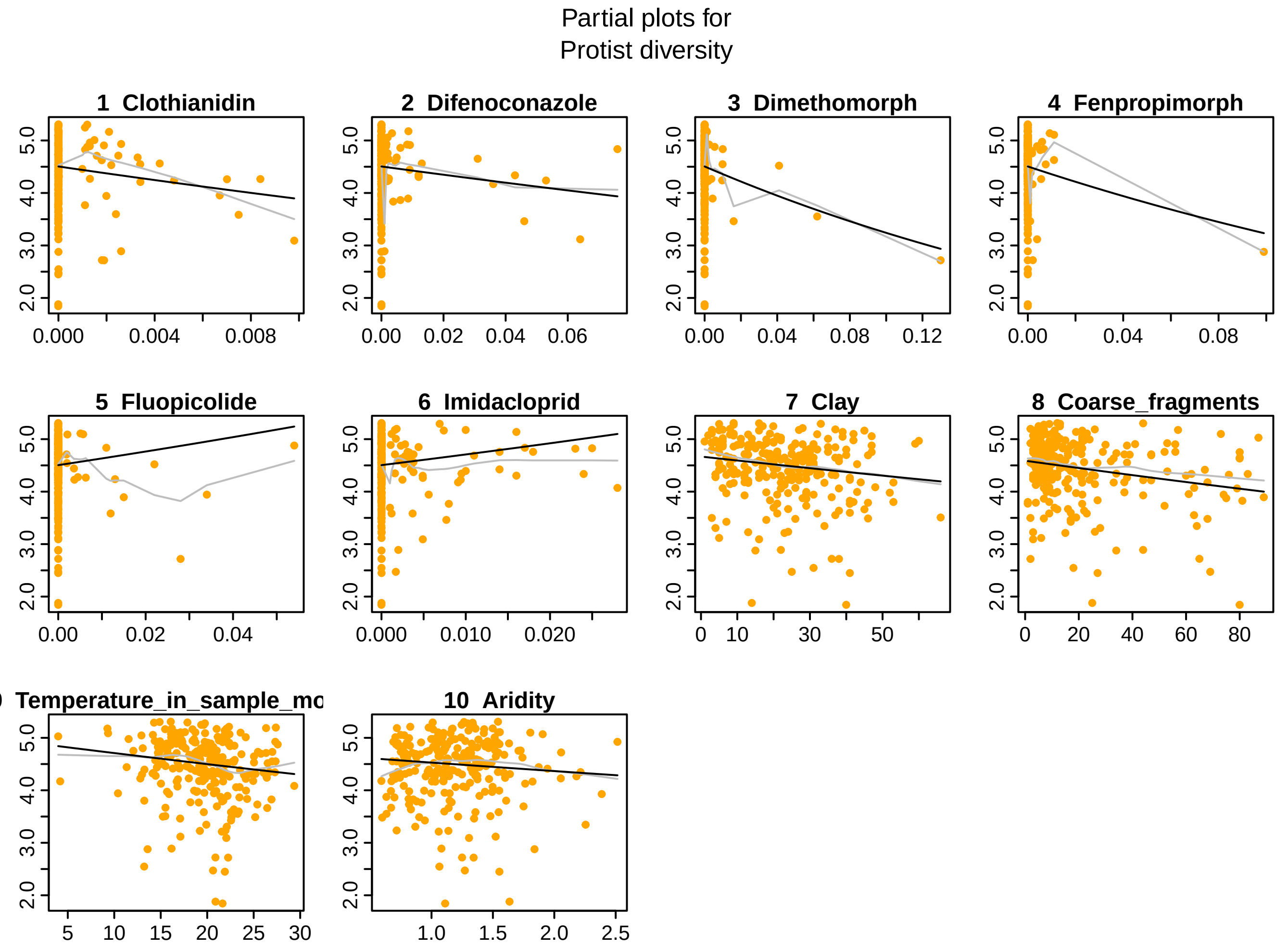
Partial plots for
Protist diversity
1 Clothianidin
2 Difenoconazole
3 Dimethomorph
4 Fenpropimorph
5 Fluopicolide
6 Imidacloprid
7 Clay
8 Coarse_fragments
Temperature_in_sample_mo
10 Aridity

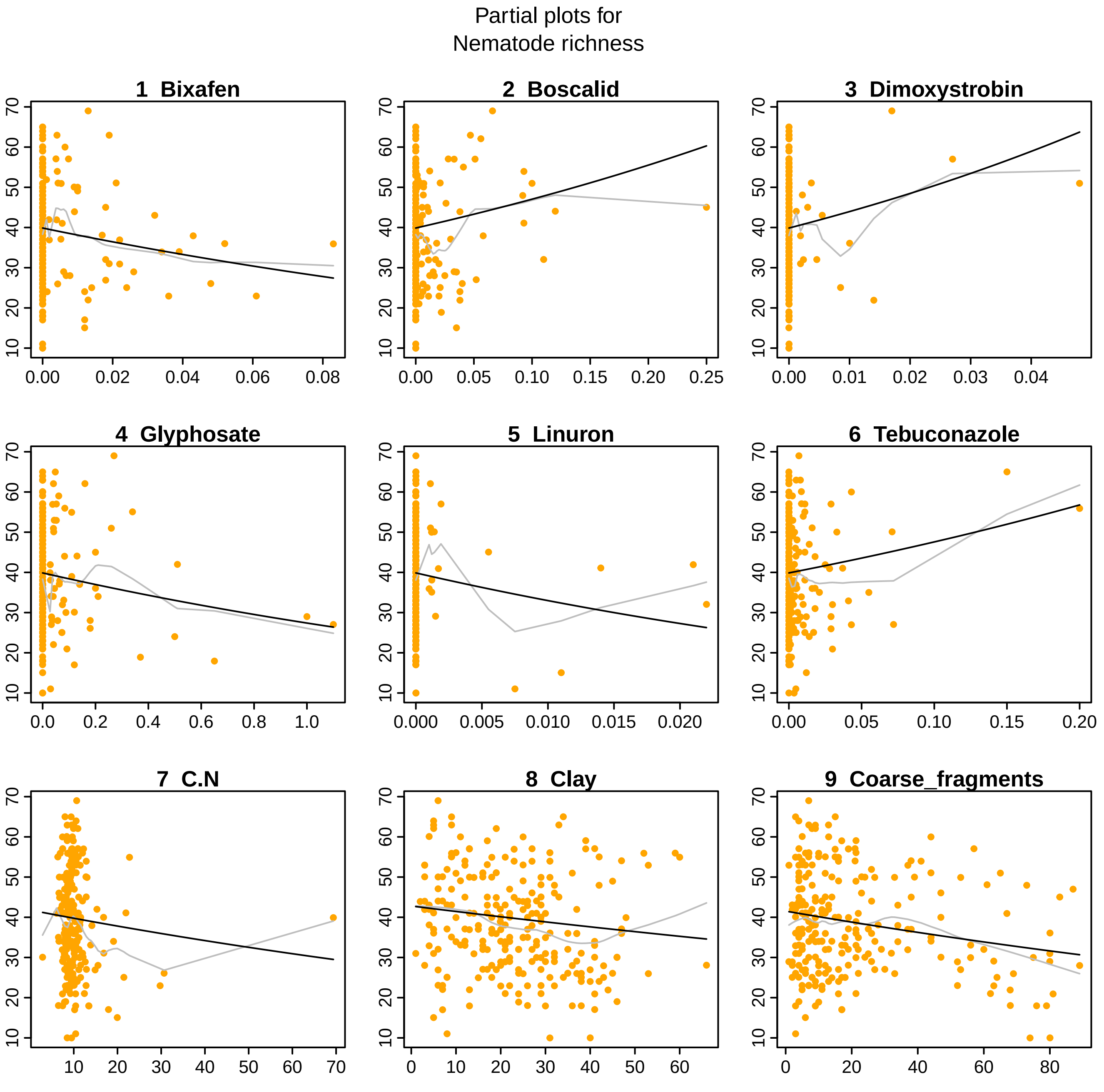
Partial plots for
Nematode richness
1 Bixafen
2 Boscalid
3 Dimoxystrobin
4 Glyphosate
5 Linuron
6 Tebuconazole
7 C.N
8 Clay
9 Coarse_fragments

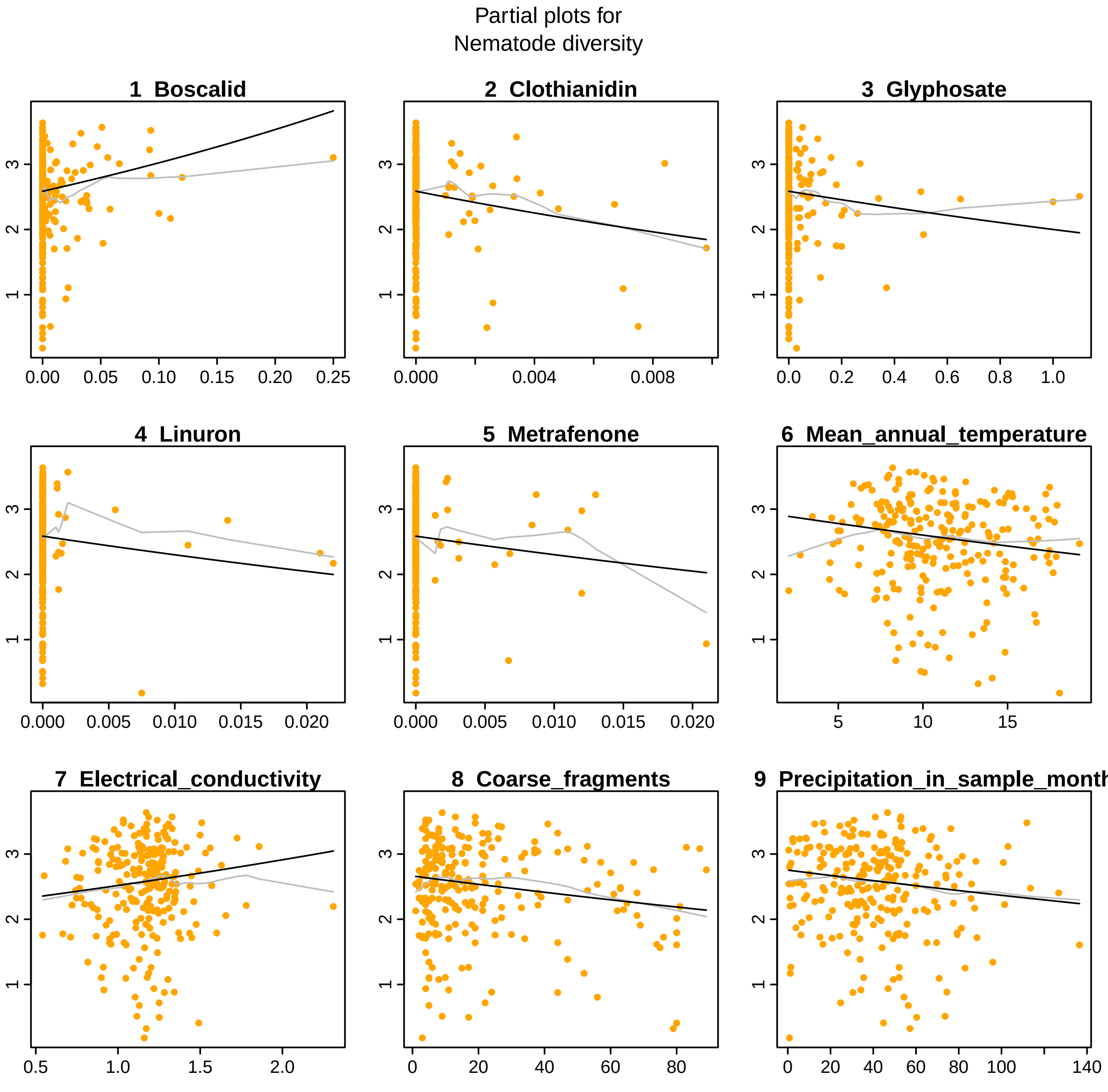

Partial plots for
Nematode diversity
1 Boscalid
2 Clothianidin
3 Glyphosate
4 Linuron
5 Metrafenone
6 Mean_annual_temperature
7 Electrical_conductivity
8 Coarse_fragments
9 Precipitation_in_sample_month

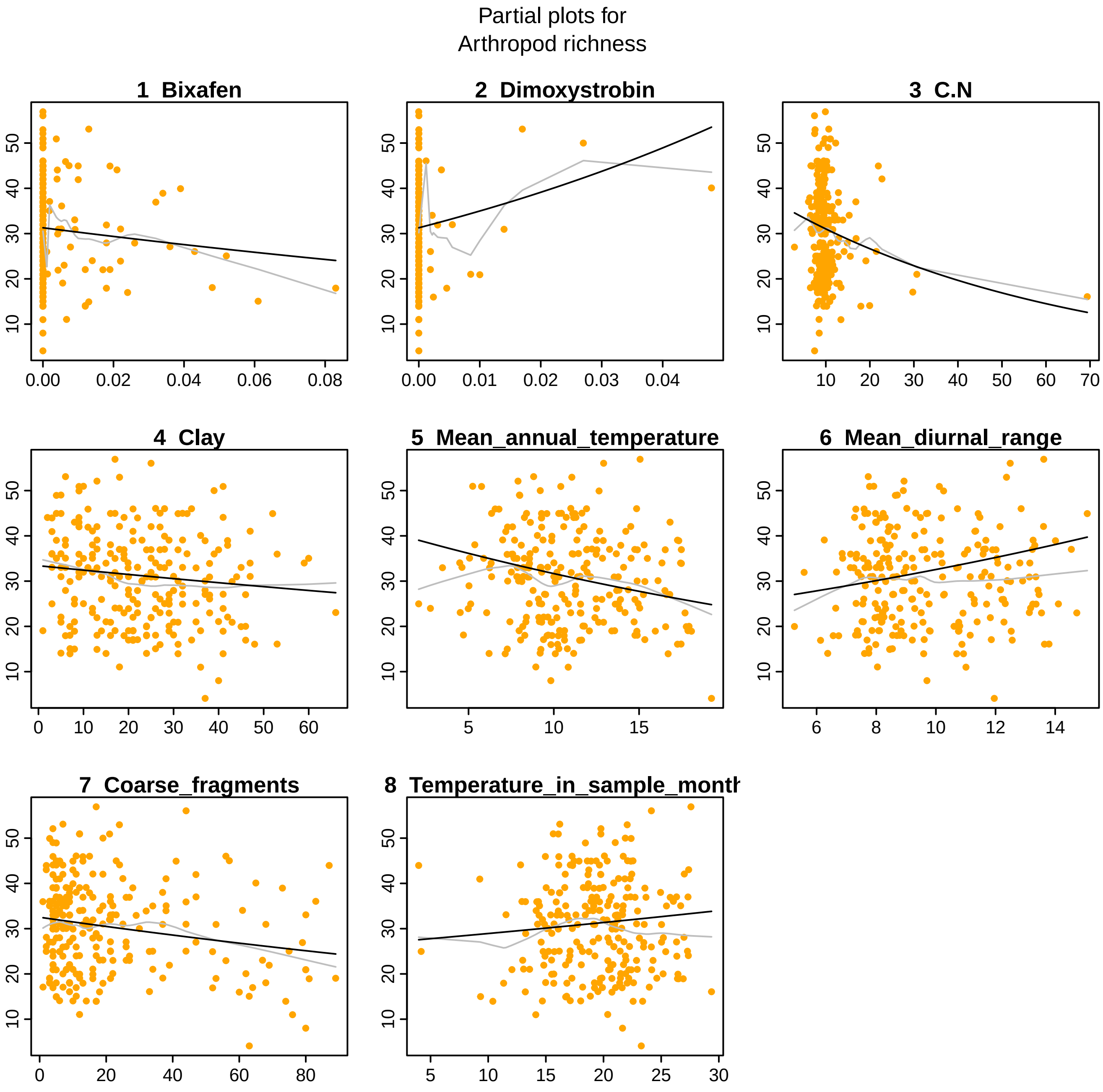

Partial plots for
Arthropod richness
1 Bixafen
2 Dimoxystrobin
3 C.N
4 Clay
5 Mean_annual_temperature
6 Mean_diurnal_range
7 Coarse_fragments
8 Temperature_in_sample_montl

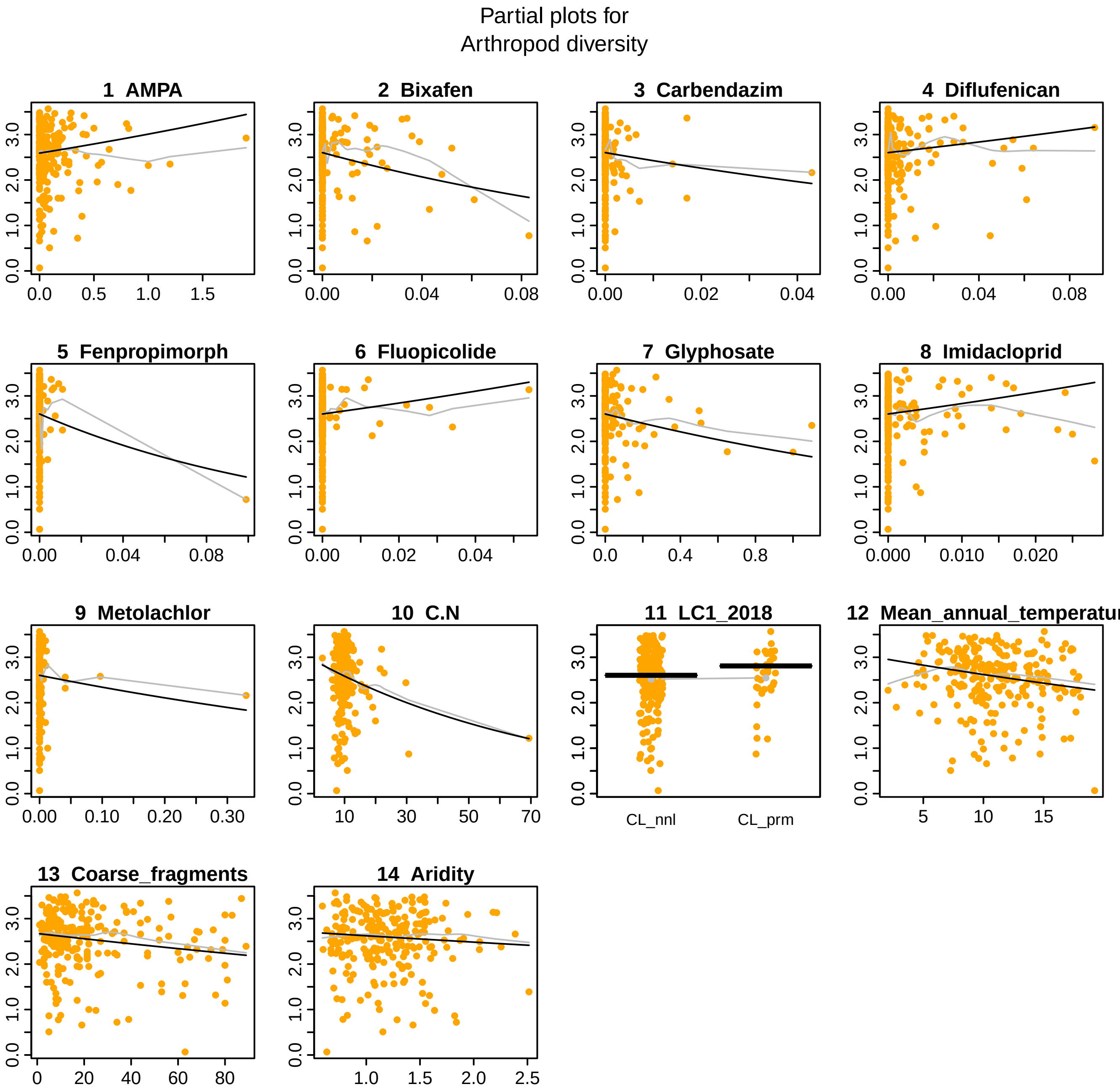
Partial plots for
Arthropod diversity
1 AMPA
2 Bixafen
3 Carbendazim
4 Diflufenican
5 Fenpropimorph
6 Fluopicolide
7 Glyphosate
8 Imidacloprid
9 Metolachlor
10 C.N
11 LC1_2018
12 Mean_annual_temperatu
13 Coarse_fragments
14 Aridity
CL_nnl
CL_prm

Partial plots for
Multidiversity

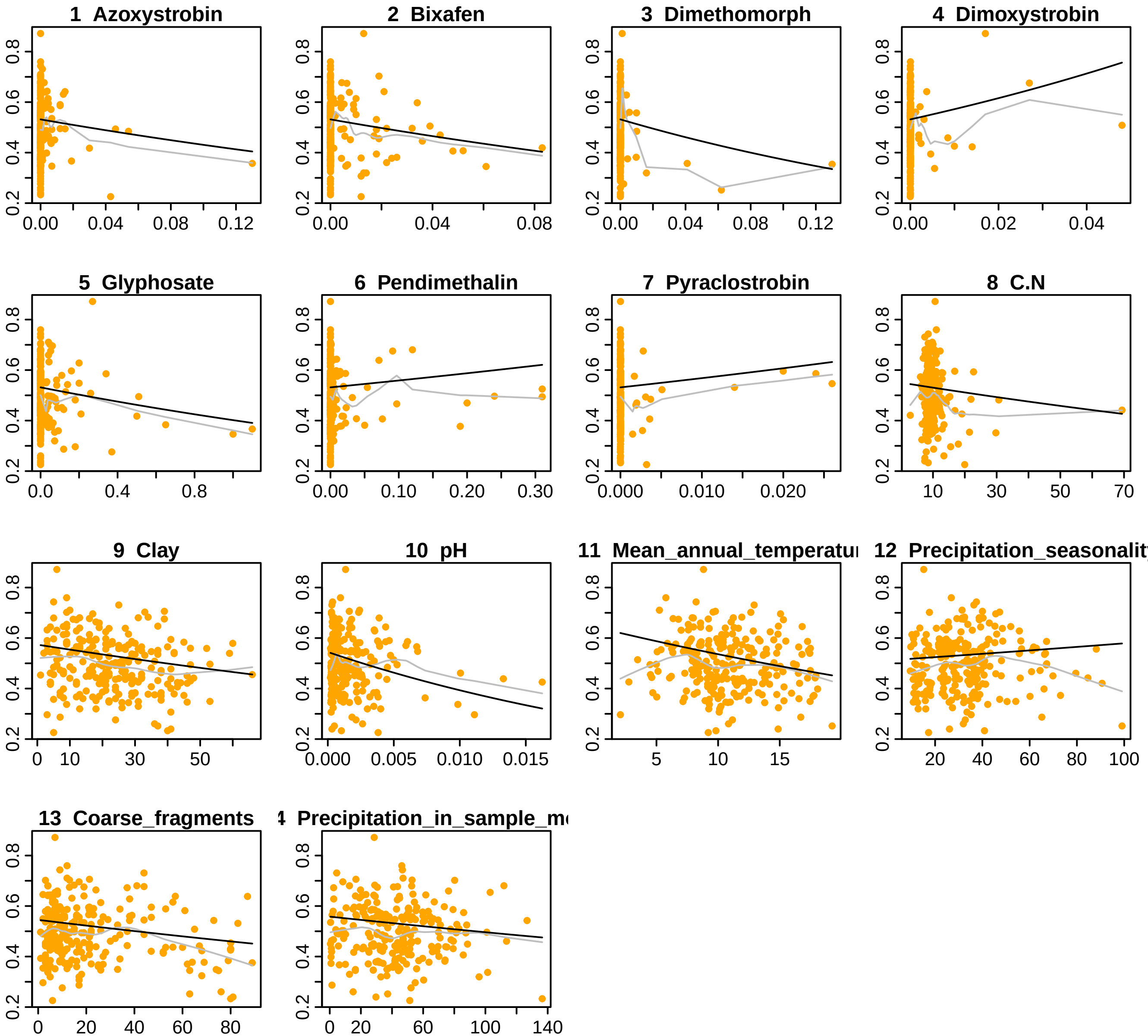

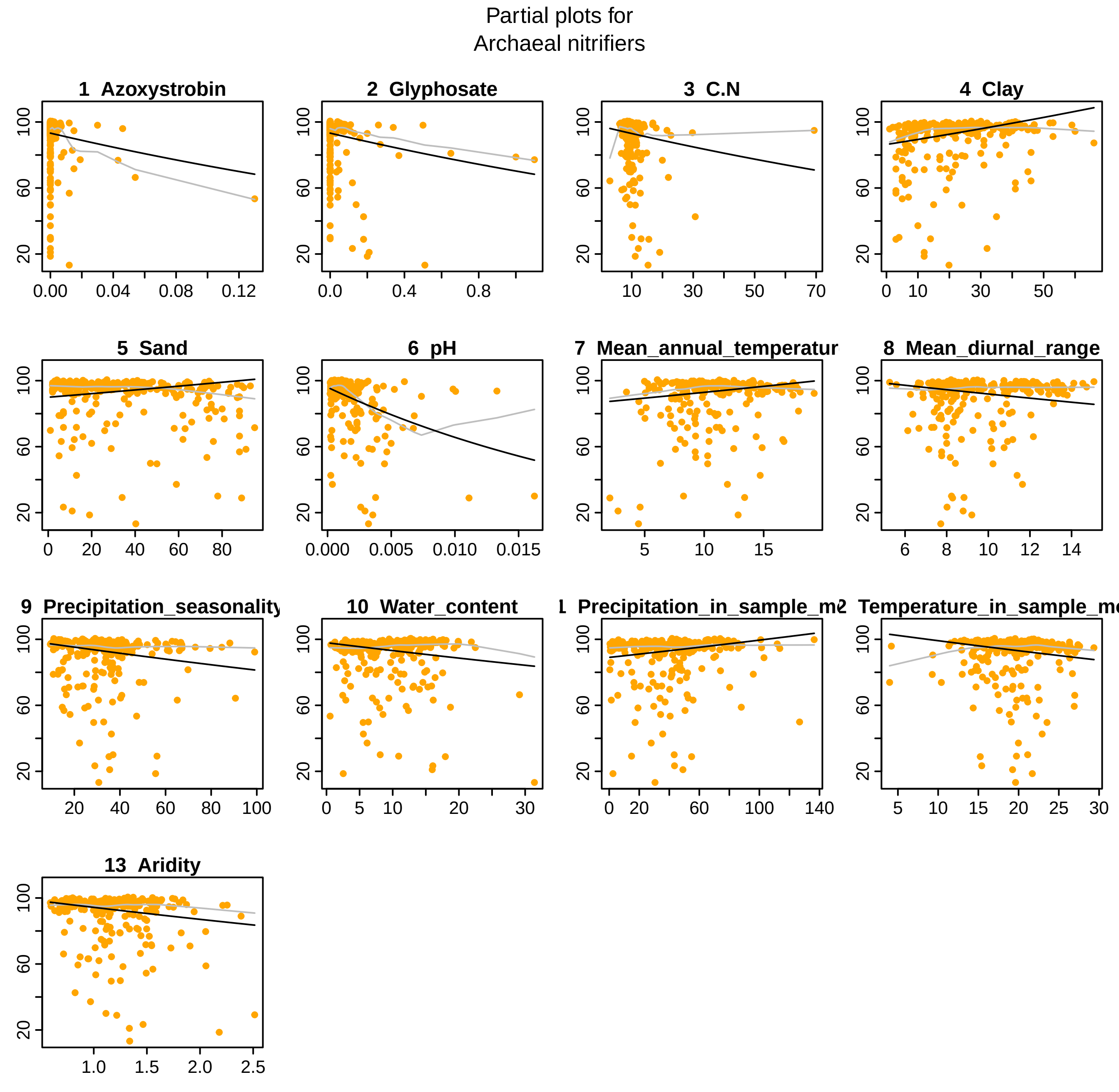
Partial plots for
Archaeal nitrifiers
1 Azoxystrobin
2 Glyphosate
3 C.N
4 Clay
5 Sand
6 pH
7 Mean_annual_temperatur
8 Mean_diurnal_range
9 Precipitation_seasonality
10 Water_content
Precipitation_in_sample_m
Temperature_in_sample_m
13 Aridity

Partial plots for
Bacterial chemoheterotrophs

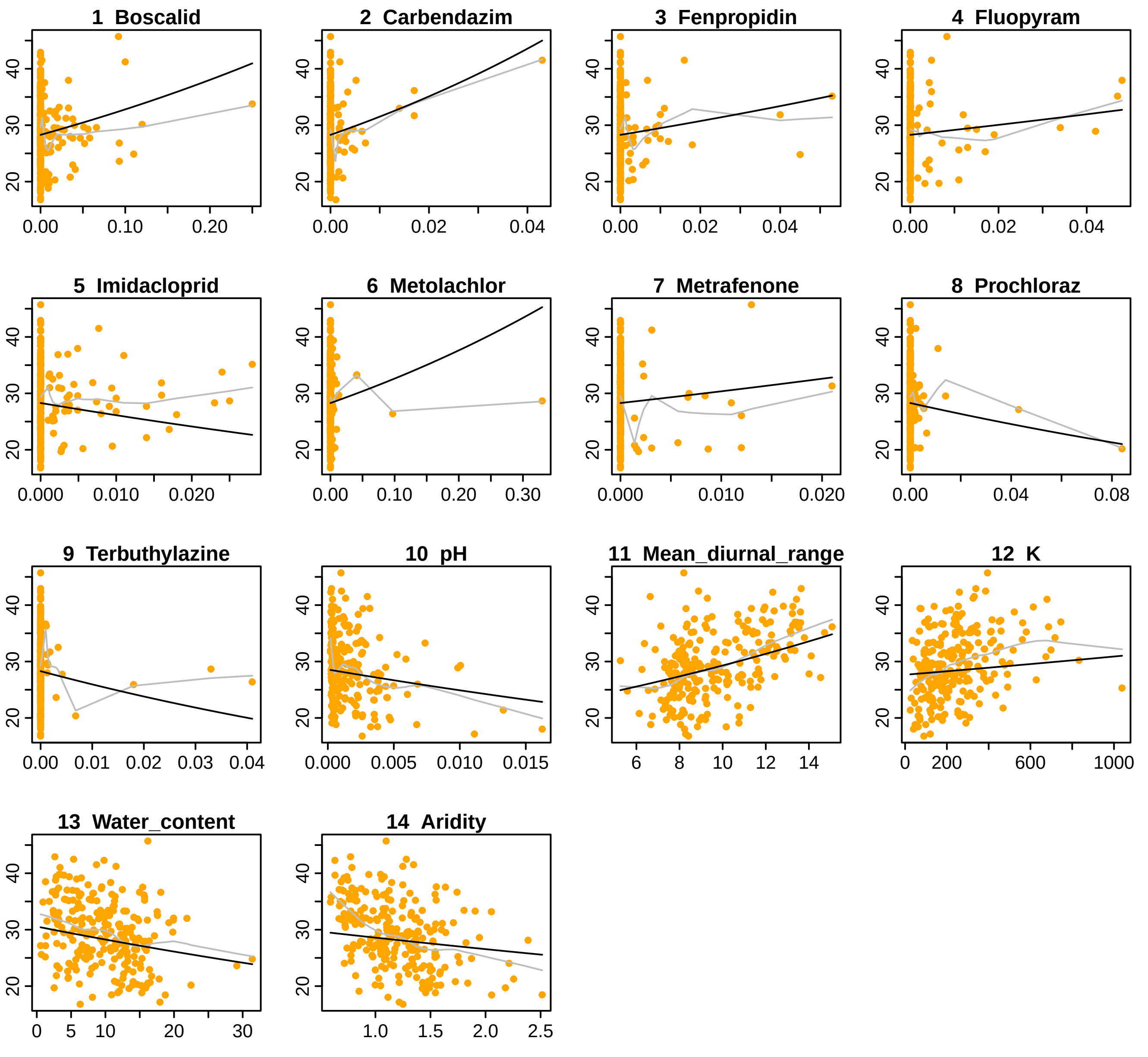

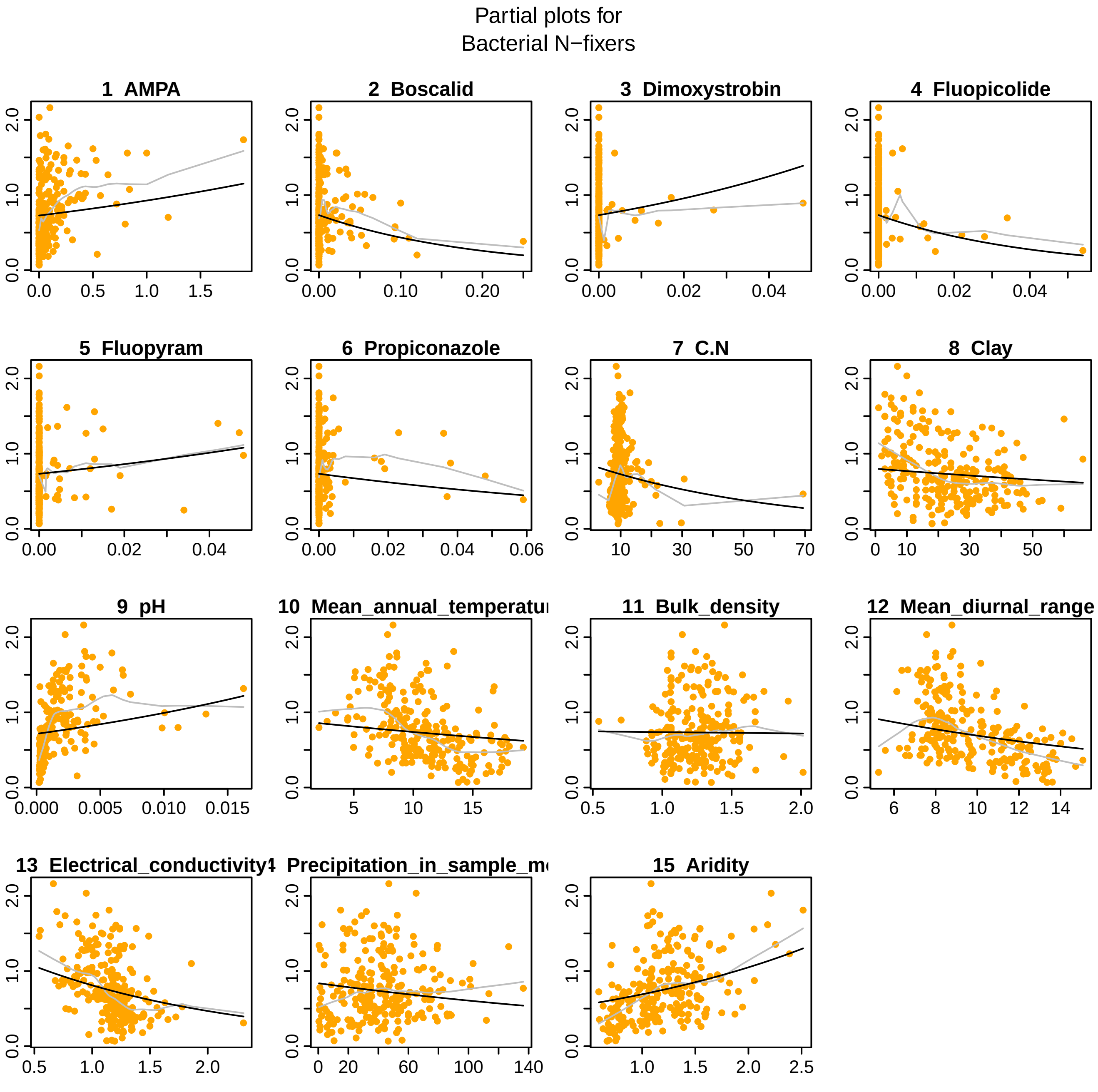

Partial plots for
Bacterial N−fixers
1 AMPA
2 Boscalid
3 Dimoxystrobin
4 Fluopicolide
5 Fluopyram
6 Propiconazole
7 C.N
8 Clay
9 pH
10 Mean_annual_temperatu
11 Bulk_density
12 Mean_diurnal_range
13 Electrical_conductivity
Precipitation_in_sample_m
15 Aridity

Partial plots for
AMF

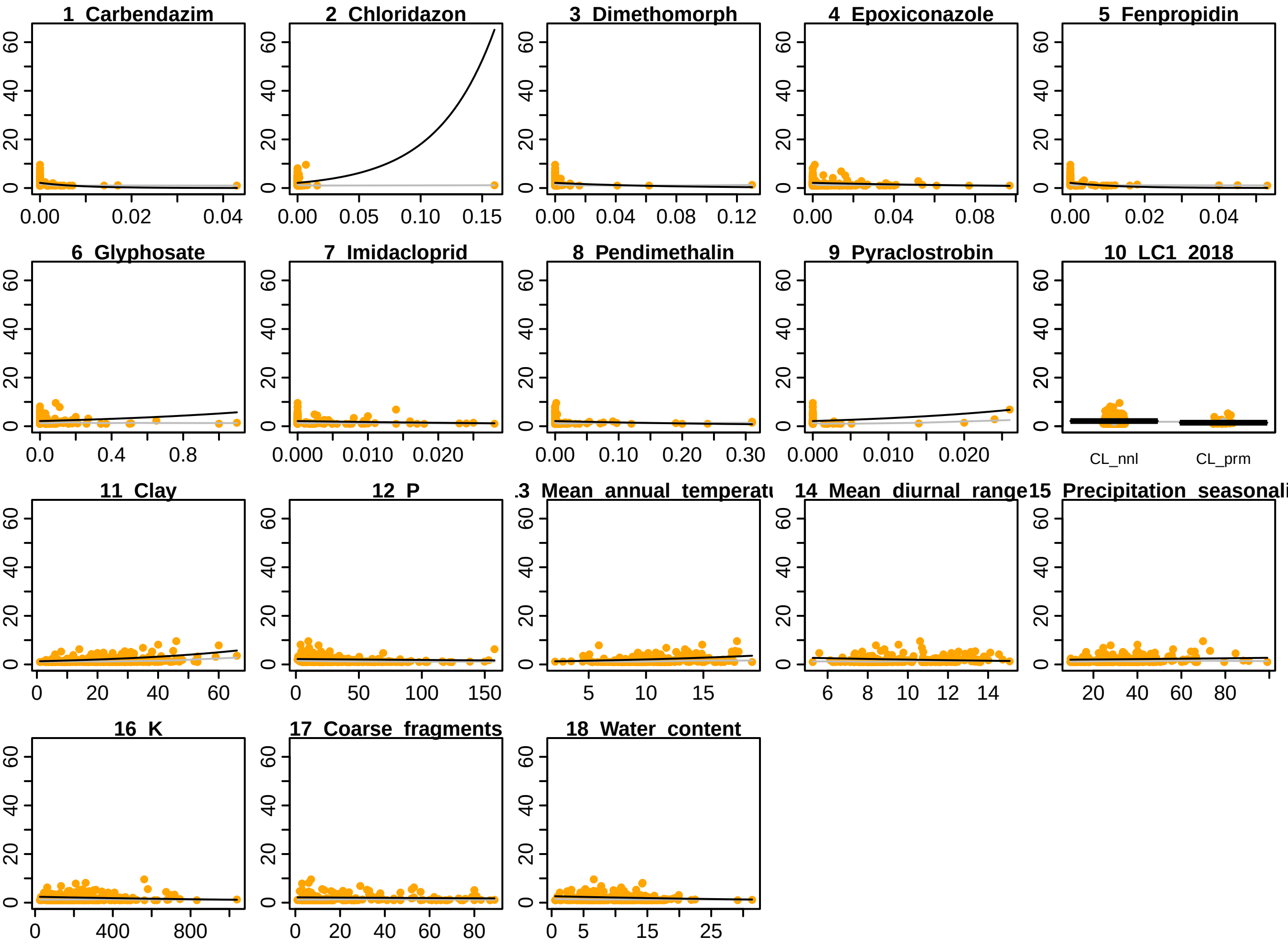

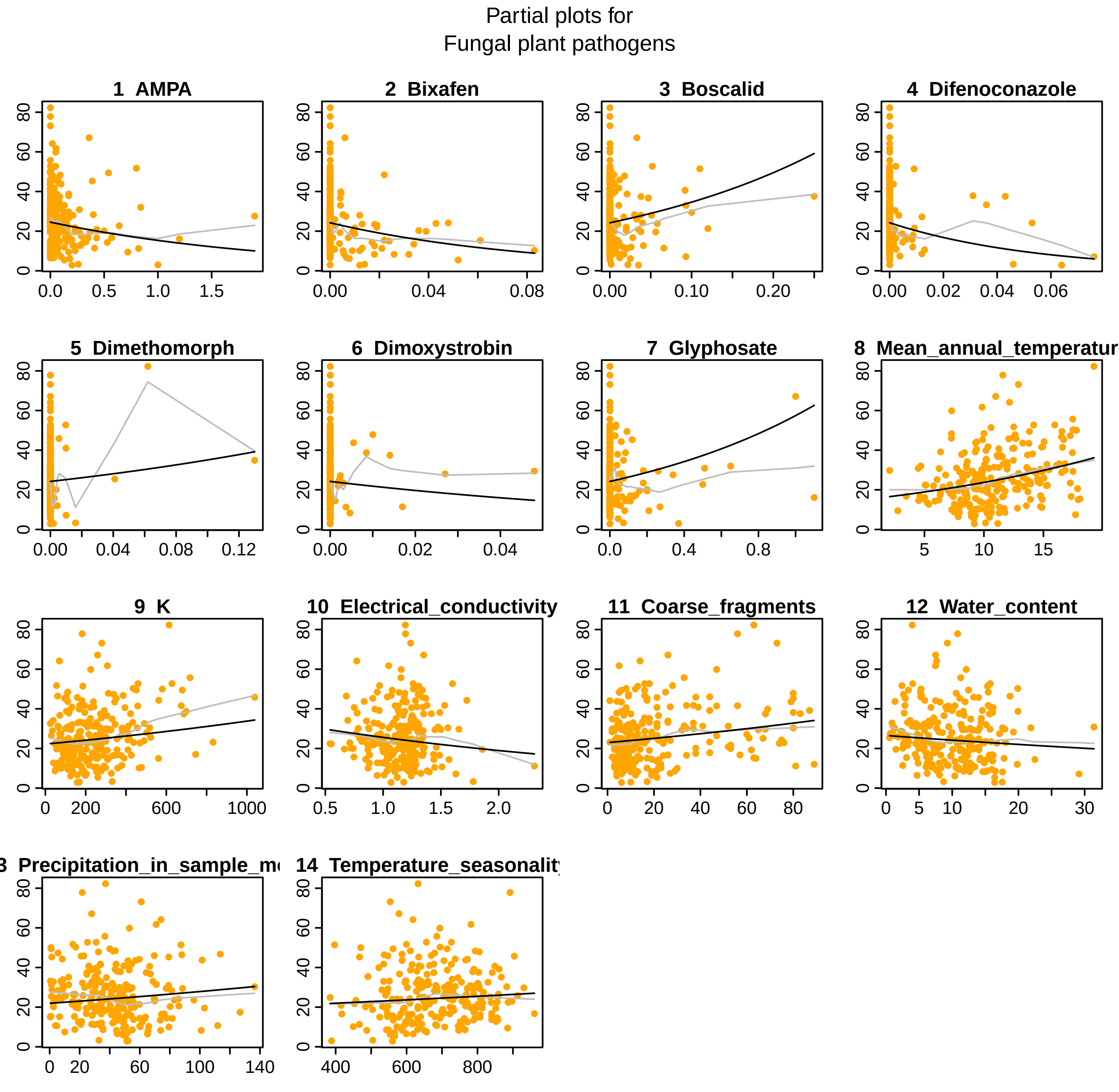

Partial plots for
Fungal plant pathogens
1 AMPA
2 Bixafen
3 Boscalid
4 Difenoconazole
5 Dimethomorph
6 Dimoxystrobin
7 Glyphosate
8 Mean_annual_temperatur
9 K
10 Electrical_conductivity
11 Coarse_fragments
12 Water_content
3 Precipitation_in_sample_m
14 Temperature_seasonalit

Partial plots for
Protist animal parasites

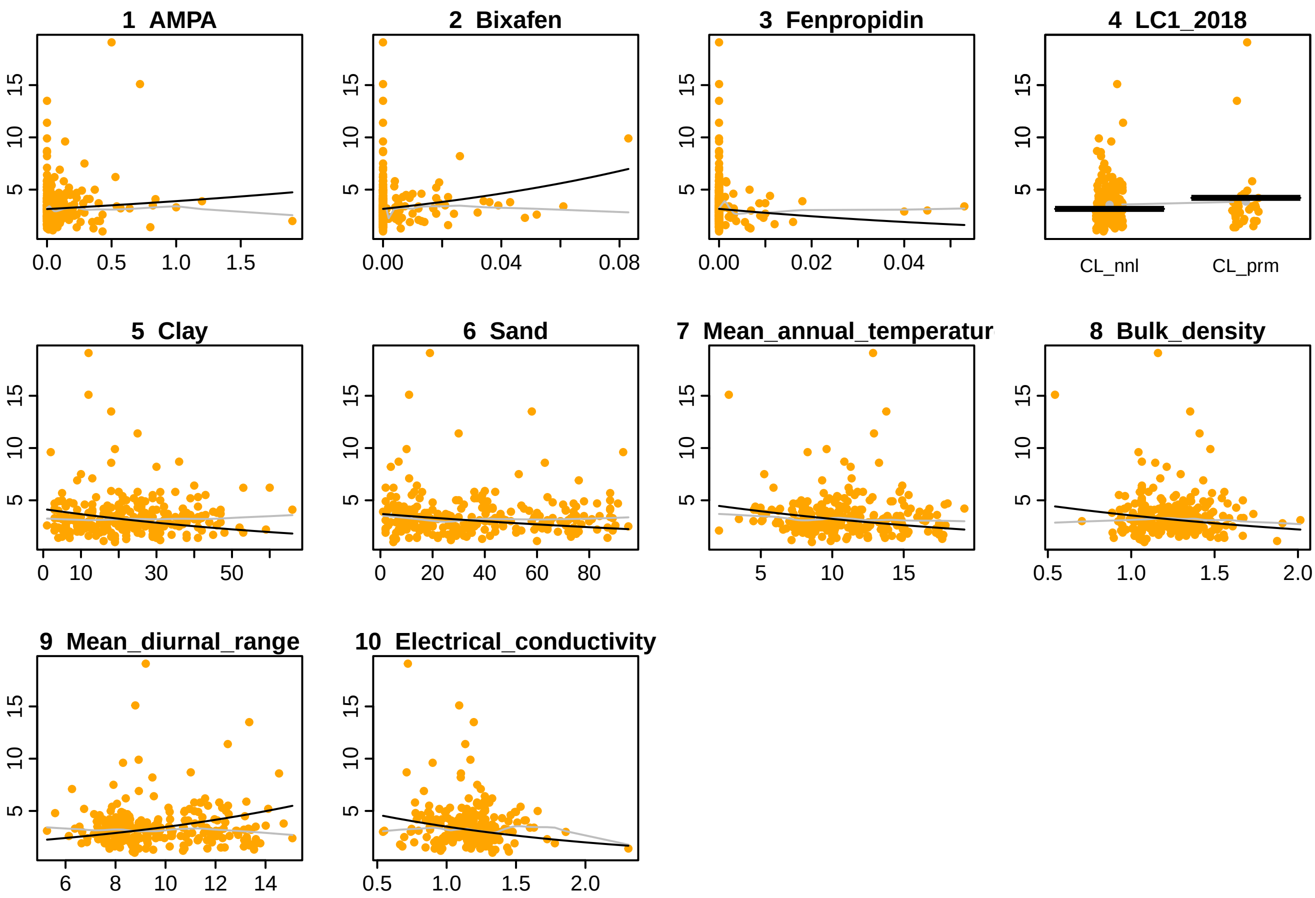

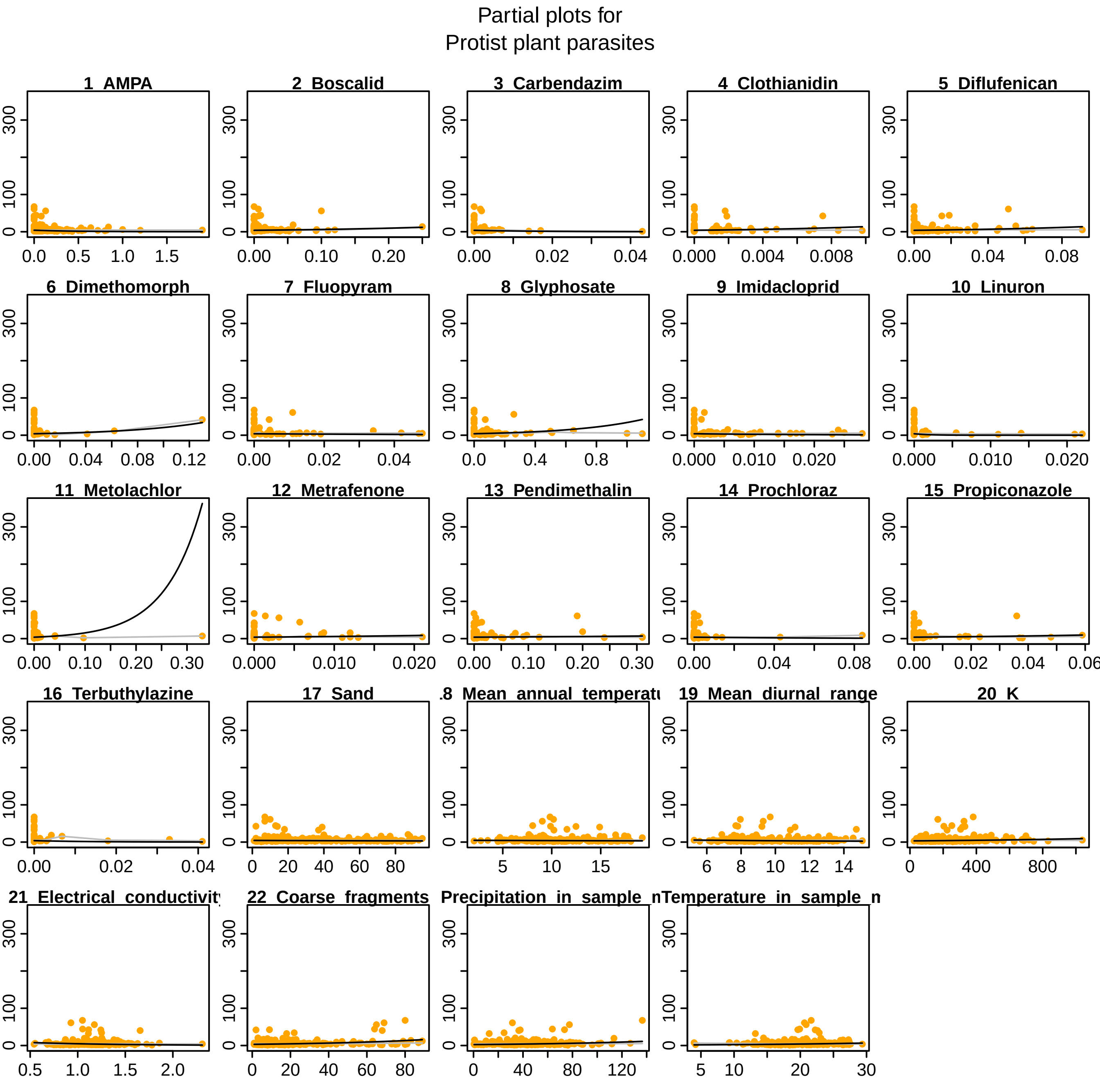

Partial plots for
Protist plant parasites
1 AMPA
2 Boscalid
3 Carbendazim
4 Clothianidin
5 Diflufenican
6 Dimethomorph
7 Fluopyram
8 Glyphosate
9 Imidacloprid
10 Linuron
11 Metolachlor
12 Metrafenone
13 Pendimethalin
14 Prochloraz
15 Propiconazole
16 Terbuthylazine
17 Sand
.8 Mean annual temperatu
19 Mean diurnal range
20 K
21 Electrical conductivity
22 Coarse fragments
Precipitation in sample n
Temperature in sample n
0
100
300

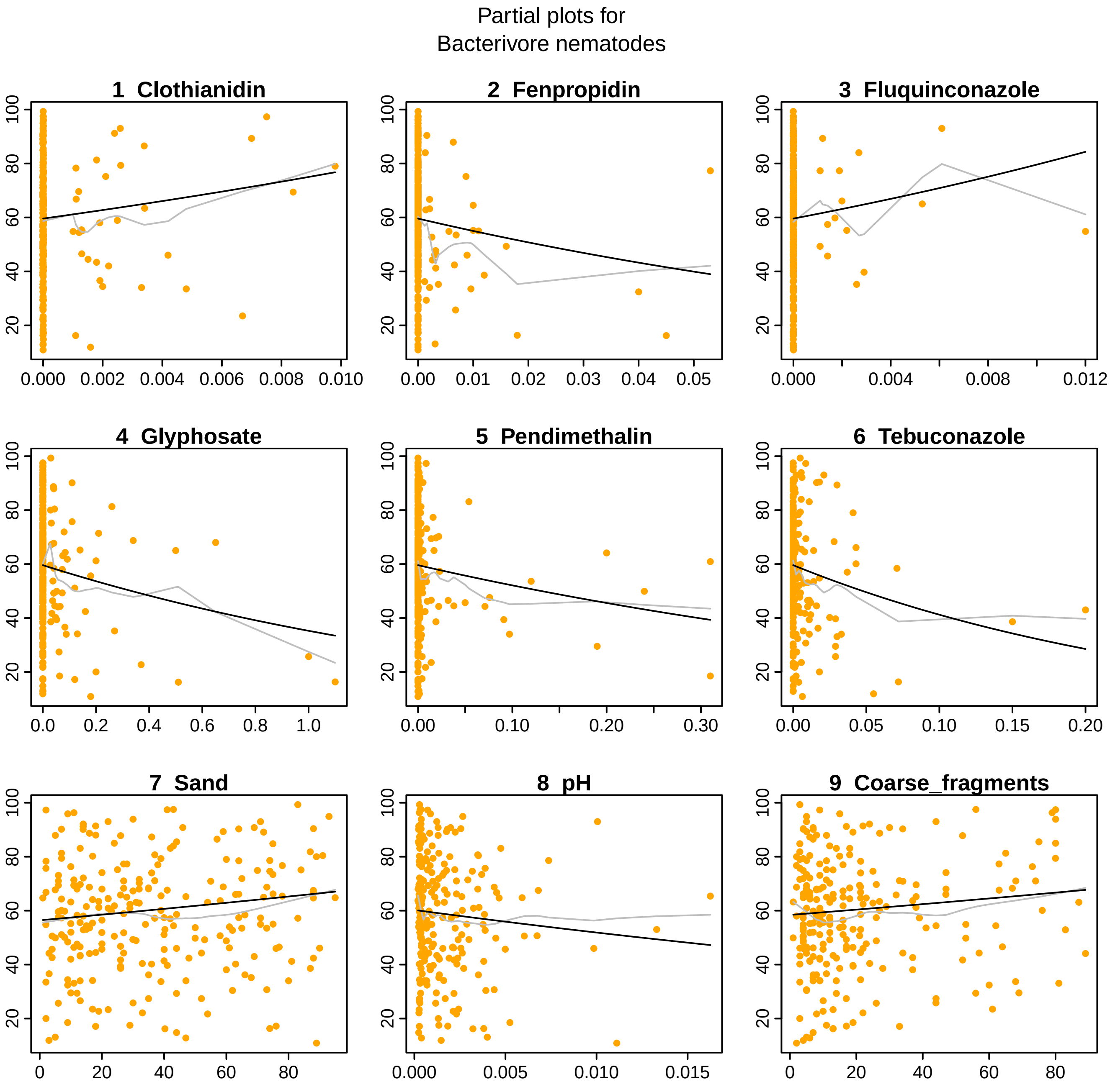

Partial plots for
Bacterivore nematodes
1 Clothianidin
2 Fenpropidin
3 Fluquinconazole
4 Glyphosate
5 Pendimethalin
6 Tebuconazole
7 Sand
8 pH
9 Coarse_fragments

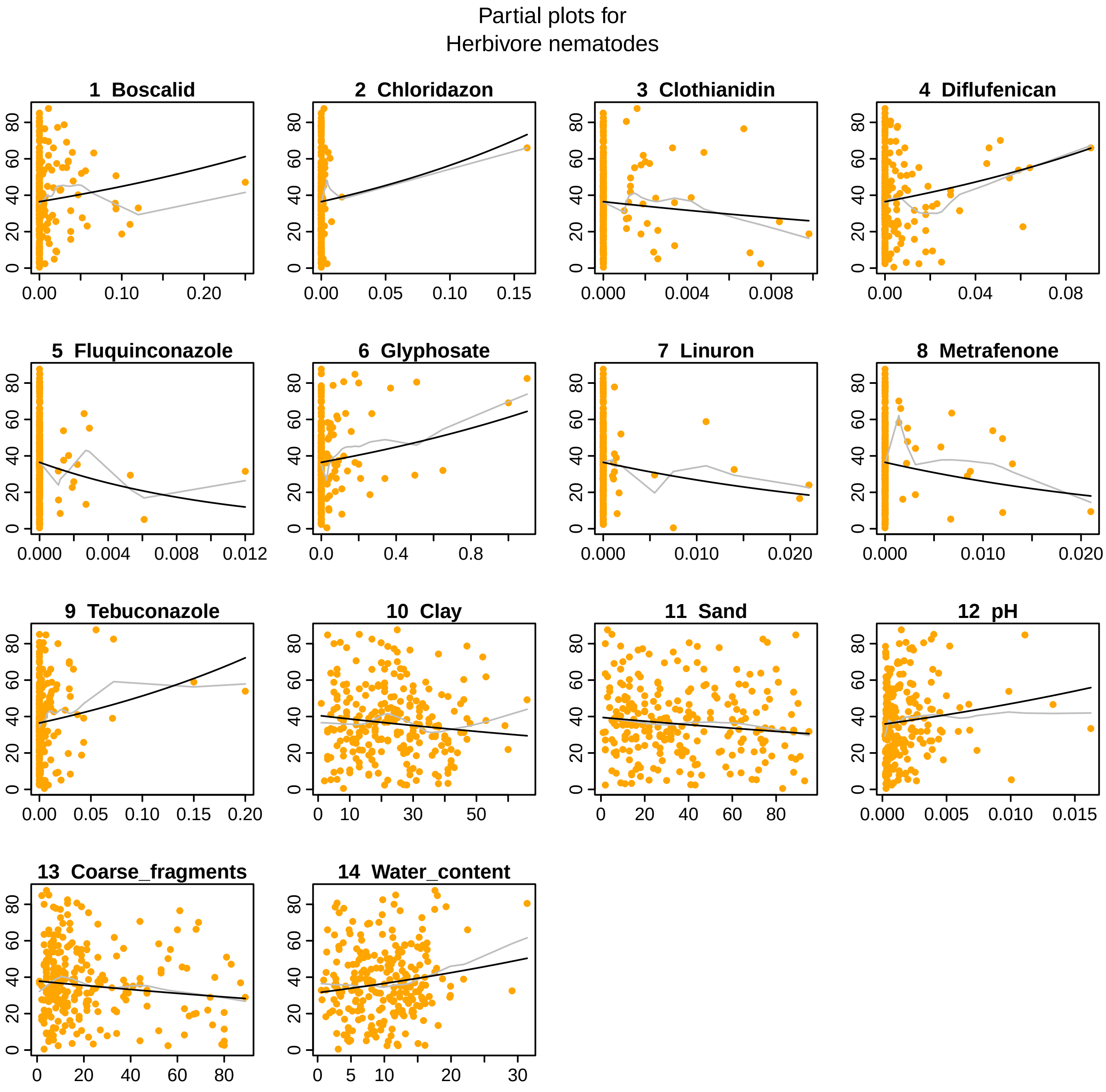
Partial plots for
Herbivore nematodes
1 Boscalid
2 Chloridazon
3 Clothianidin
4 Diflufenican
5 Fluquinconazole
6 Glyphosate
7 Linuron
8 Metrafenone
9 Tebuconazole
10 Clay
11 Sand
12 pH
13 Coarse_fragments
14 Water_content

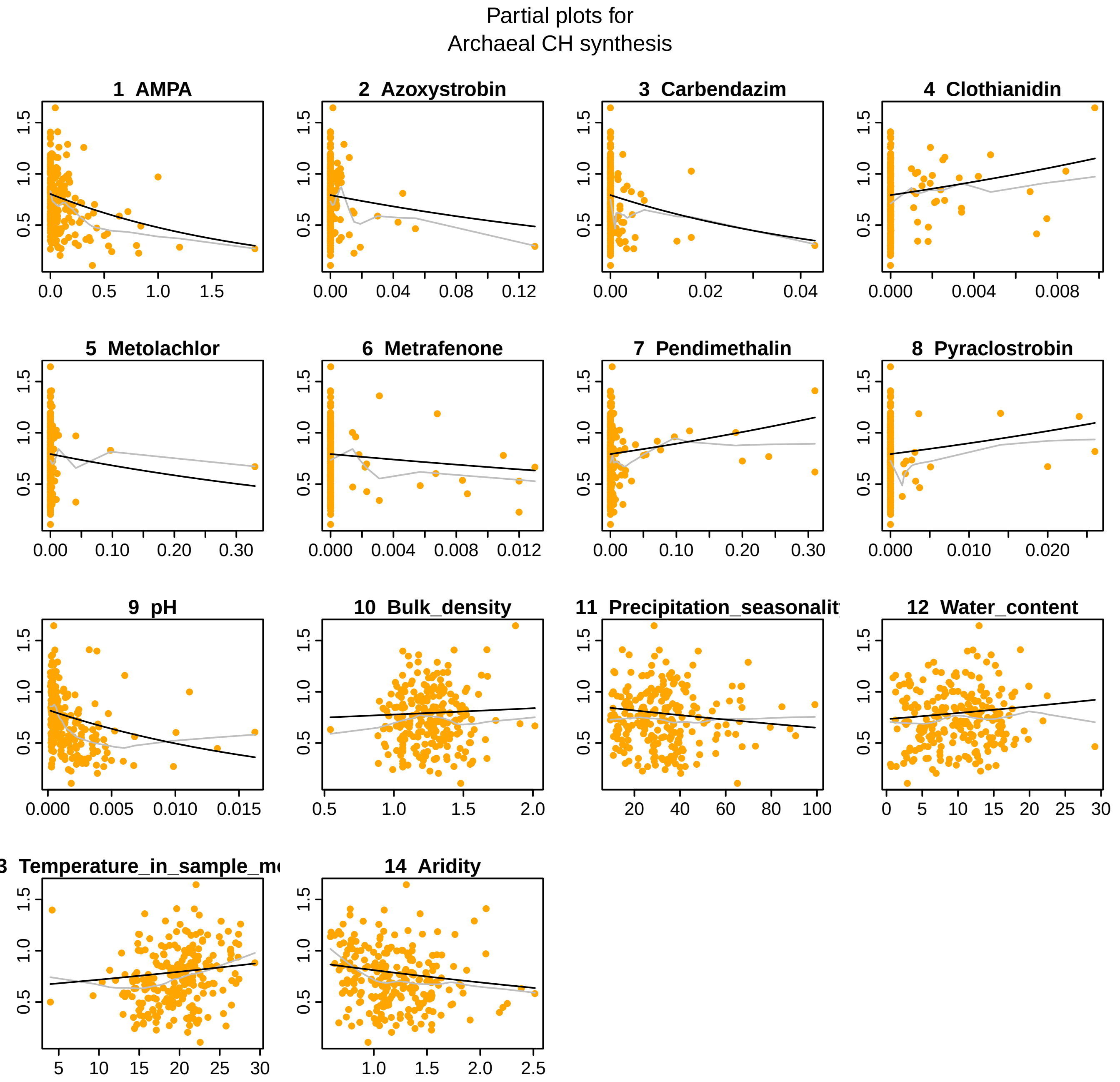

Partial plots for
Archaeal CH synthesis
1 AMPA
2 Azoxystrobin
3 Carbendazim
4 Clothianidin
5 Metolachlor
6 Metrafenone
7 Pendimethalin
8 Pyraclostrobin
9 pH
10 Bulk_density
11 Precipitation_seasonalit
12 Water_content
3 Temperature_in_sample_mo
14 Aridity

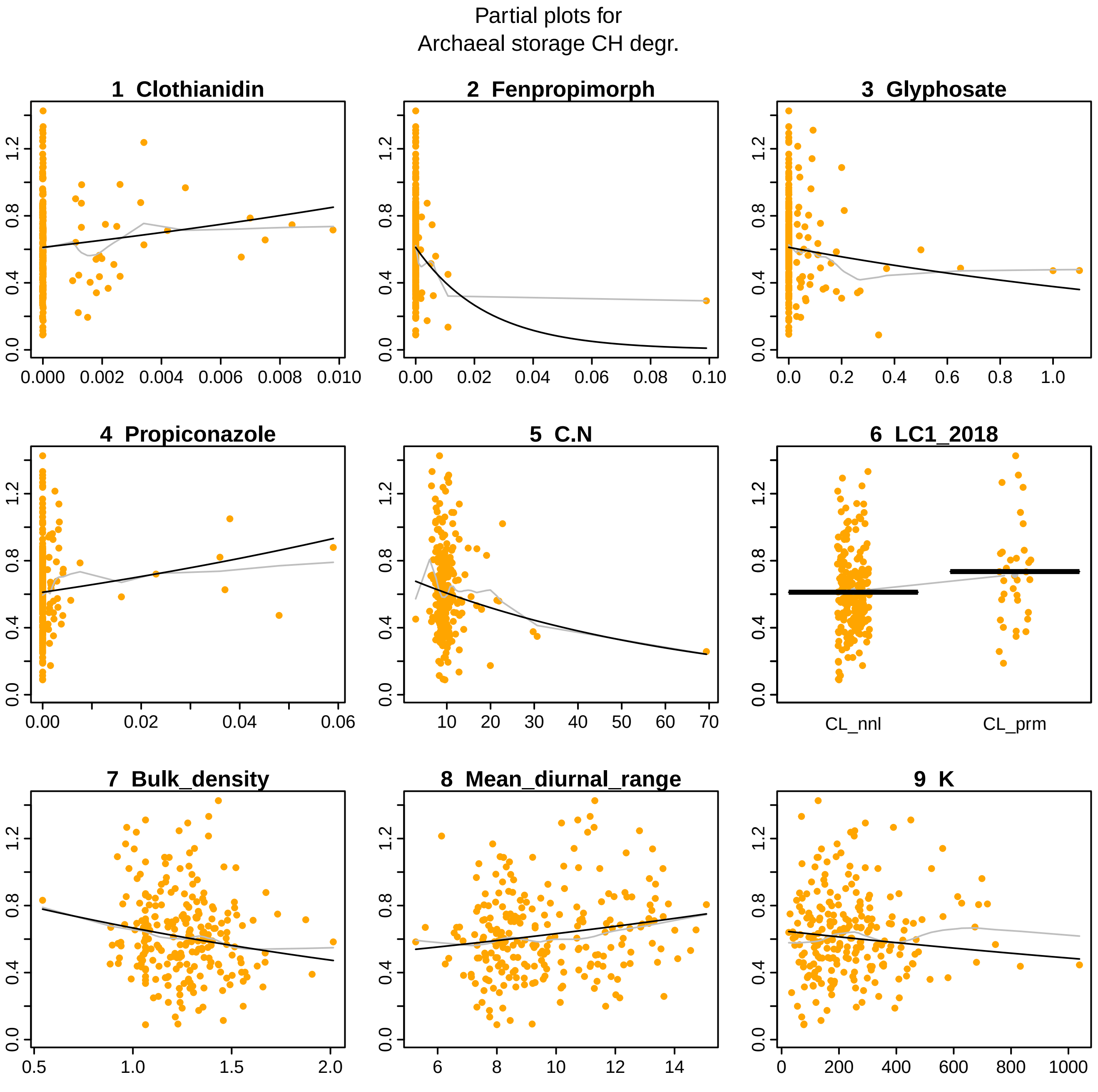

Partial plots for
Archaeal storage CH degr.
1 Clothianidin
2 Fenpropimorph
3 Glyphosate
4 Propiconazole
5 C.N
6 LC1_2018
7 Bulk_density
8 Mean_diurnal_range
9 K
0.0
0.4
0.8
1.2
0.000 0.002 0.004 0.006 0.008 0.010
0.00 0.02 0.04 0.06 0.08 0.10
0.0 0.2 0.4 0.6 0.8 1.0
0.00 0.02 0.04 0.06
10 20 30 40 50 60 70
CL_nnl
CL_prm
0.5 1.0 1.5 2.0
6 8 10 12 14
0 200 400 600 800 1000

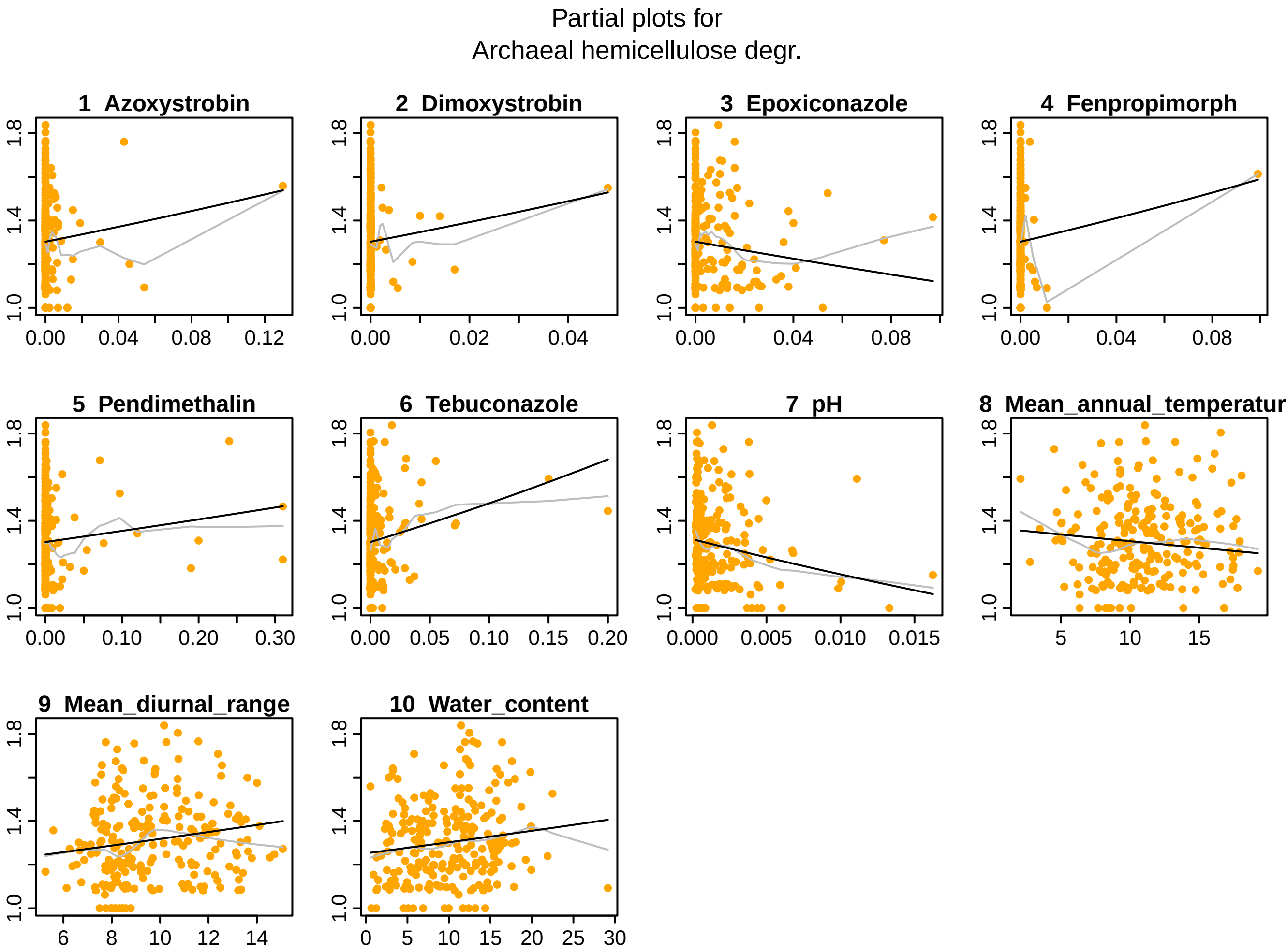

Partial plots for
Archaeal hemicellulose degr.
1 Azoxystrobin
2 Dimoxystrobin
3 Epoxiconazole
4 Fenpropimorph
5 Pendimethalin
6 Tebuconazole
7 pH
8 Mean_annual_temperatur
9 Mean_diurnal_range
10 Water_content

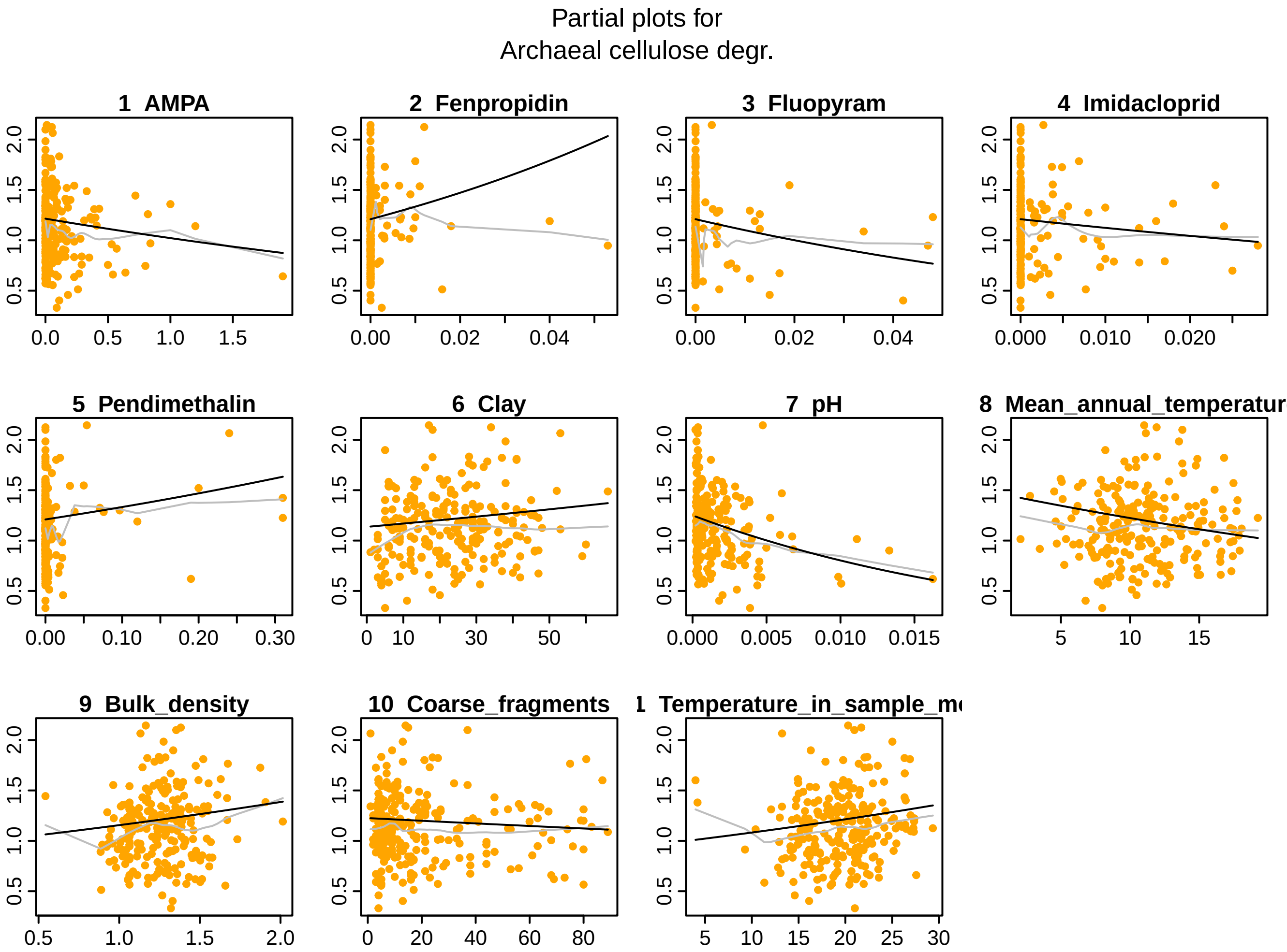
Partial plots for
Archaeal cellulose degr.
1 AMPA
2 Fenpropidin
3 Fluopyram
4 Imidacloprid
5 Pendimethalin
6 Clay
7 pH
8 Mean_annual_temperatur
9 Bulk_density
10 Coarse_fragments
1 Temperature_in_sample_m

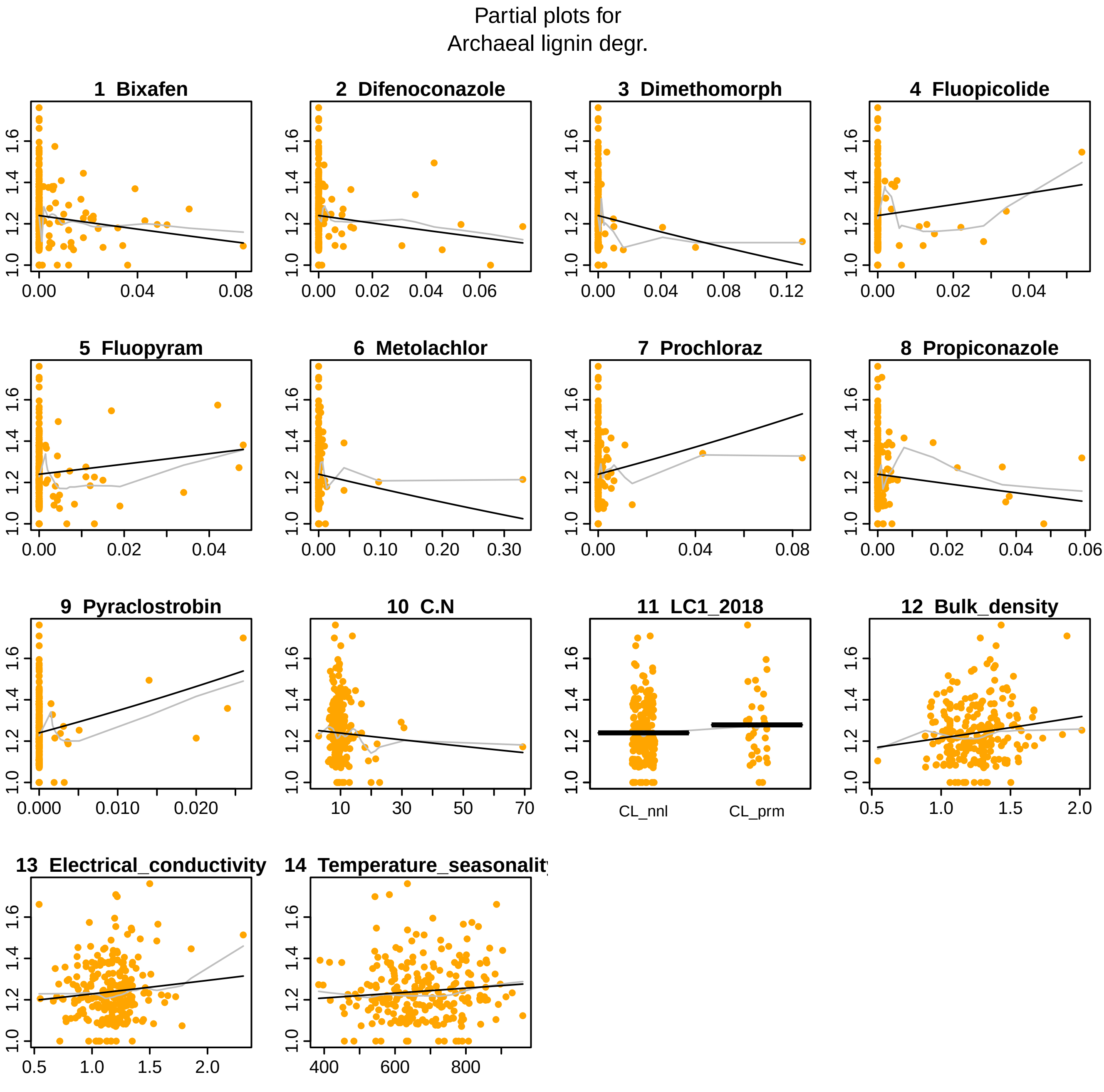
Partial plots for
Archaeal lignin degr.
1 Bixafen
2 Difenoconazole
3 Dimethomorph
4 Fluopicolide
5 Fluopyram
6 Metolachlor
7 Prochloraz
8 Propiconazole
9 Pyraclostrobin
10 C.N
11 LC1_2018
CL_nnl
CL_prm
12 Bulk_density
13 Electrical_conductivity
14 Temperature_seasonalit

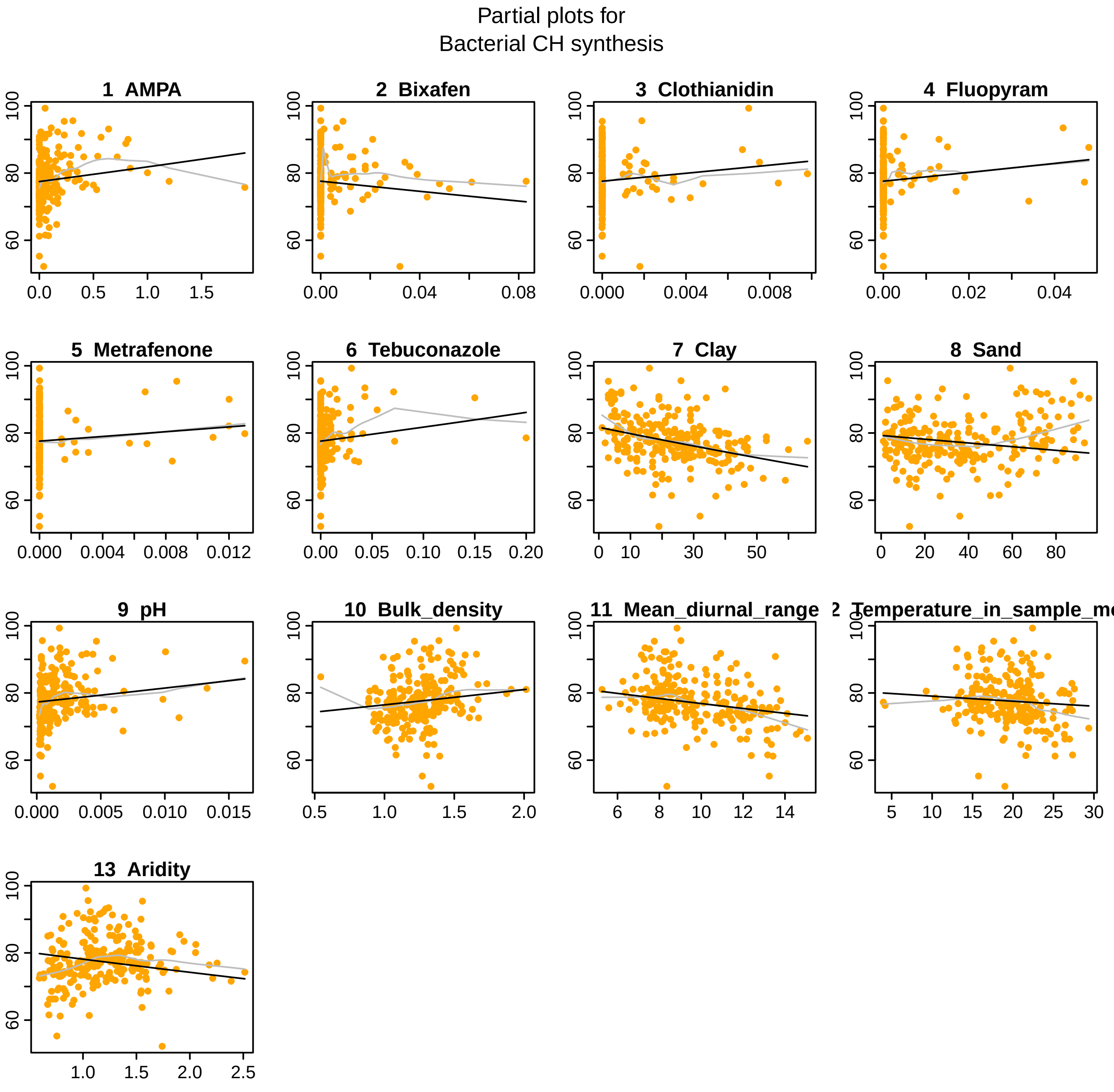
Partial plots for
Bacterial CH synthesis
1 AMPA
2 Bixafen
3 Clothianidin
4 Fluopyram
5 Metrafenone
6 Tebuconazole
7 Clay
8 Sand
9 pH
10 Bulk_density
11 Mean_diurnal_range
Temperature_in_sample_mo
13 Aridity

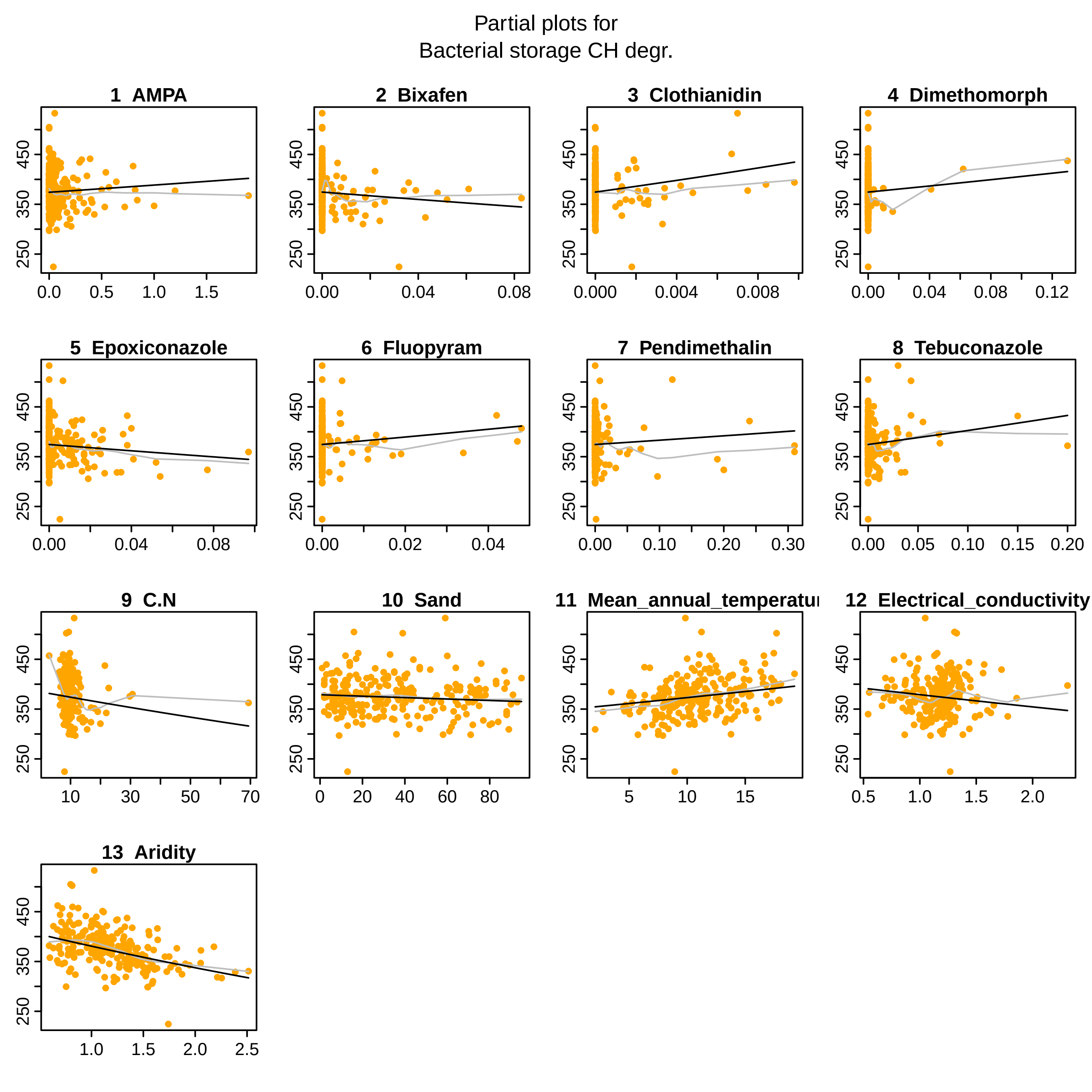

Partial plots for
Bacterial storage CH degr.
1 AMPA
2 Bixafen
3 Clothianidin
4 Dimethomorph
5 Epoxiconazole
6 Fluopyram
7 Pendimethalin
8 Tebuconazole
9 C.N
10 Sand
11 Mean_annual_temperatu
12 Electrical_conductivity
13 Aridity

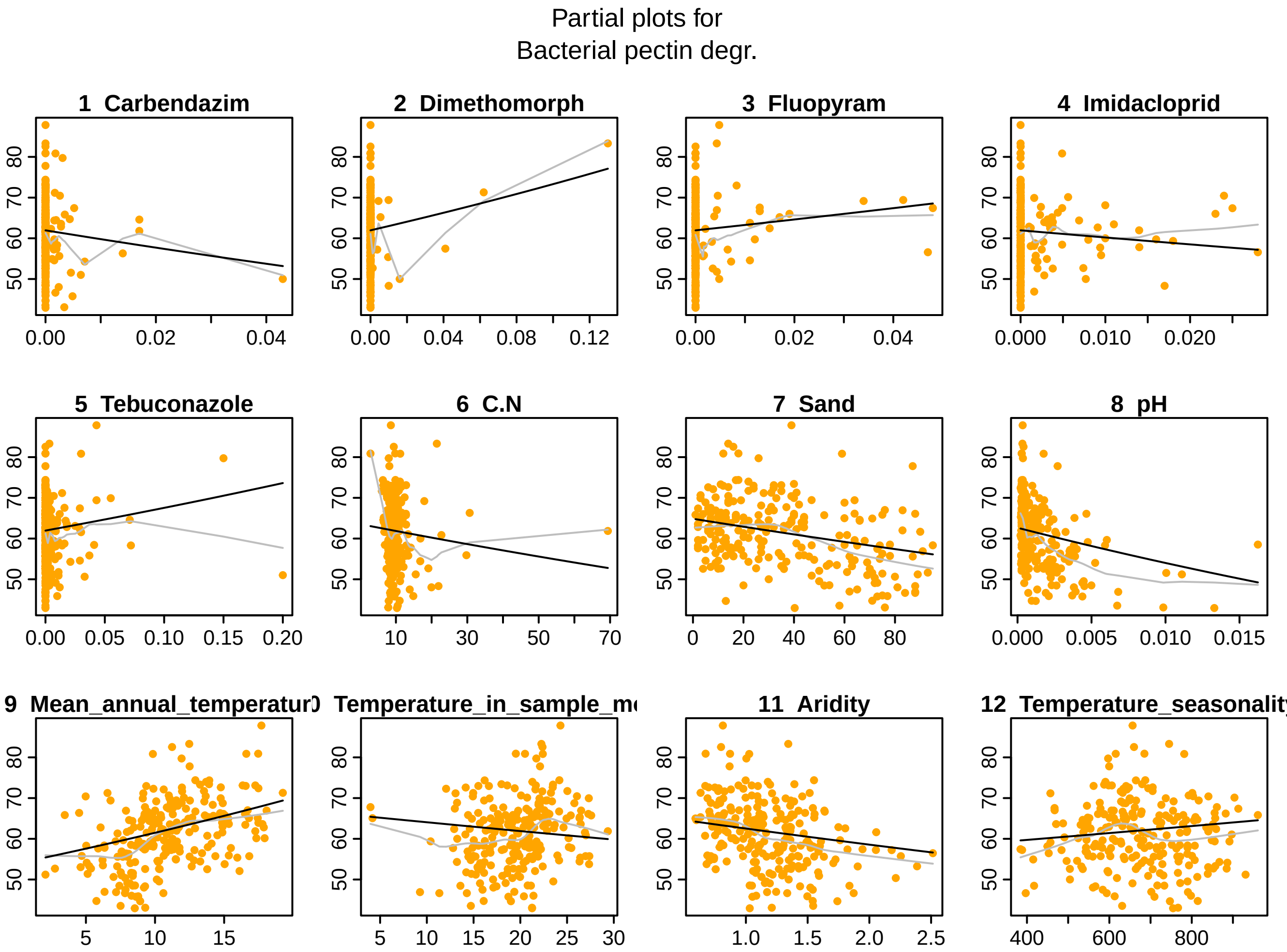
Partial plots for
Bacterial pectin degr.
1 Carbendazim
2 Dimethomorph
3 Fluopyram
4 Imidacloprid
5 Tebuconazole
6 C.N
7 Sand
8 pH
9 Mean_annual_temperatur
Temperature_in_sample_m
11 Aridity
12 Temperature_seasonality

Partial plots for
Bacterial hemicellulose degr.

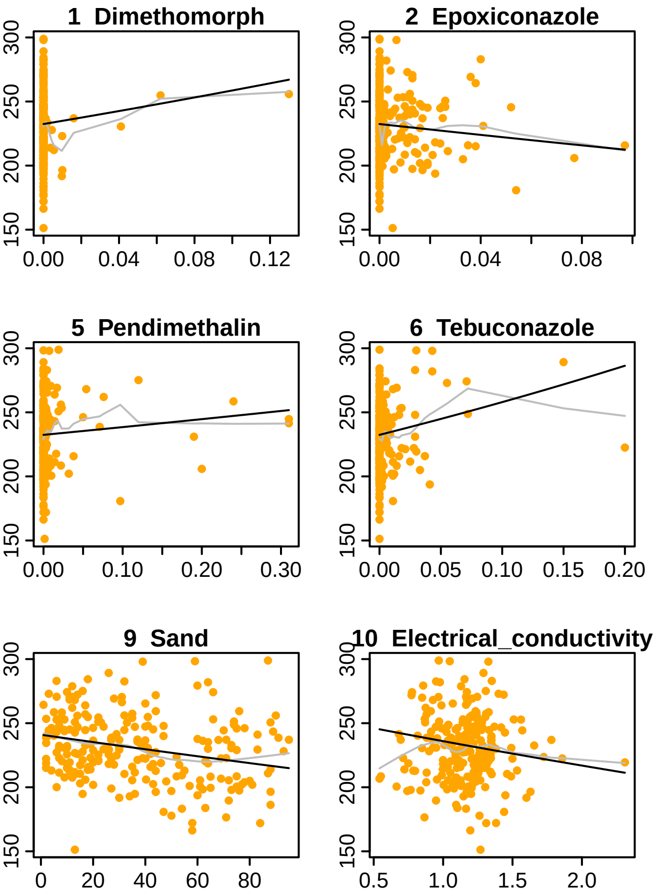

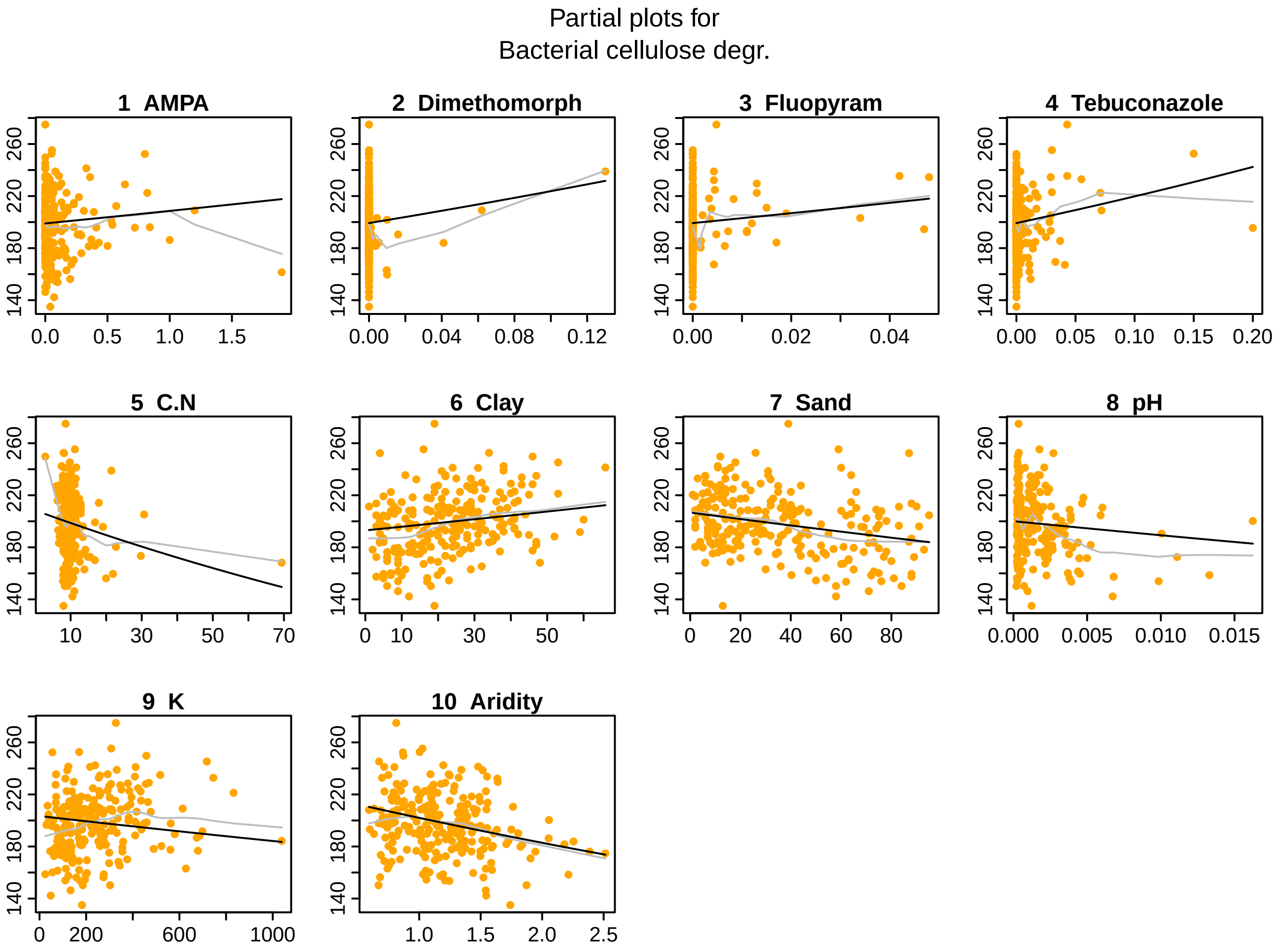

Partial plots for
Bacterial cellulose degr.
1 AMPA
2 Dimethomorph
3 Fluopyram
4 Tebuconazole
5 C.N
6 Clay
7 Sand
8 pH
9 K
10 Aridity

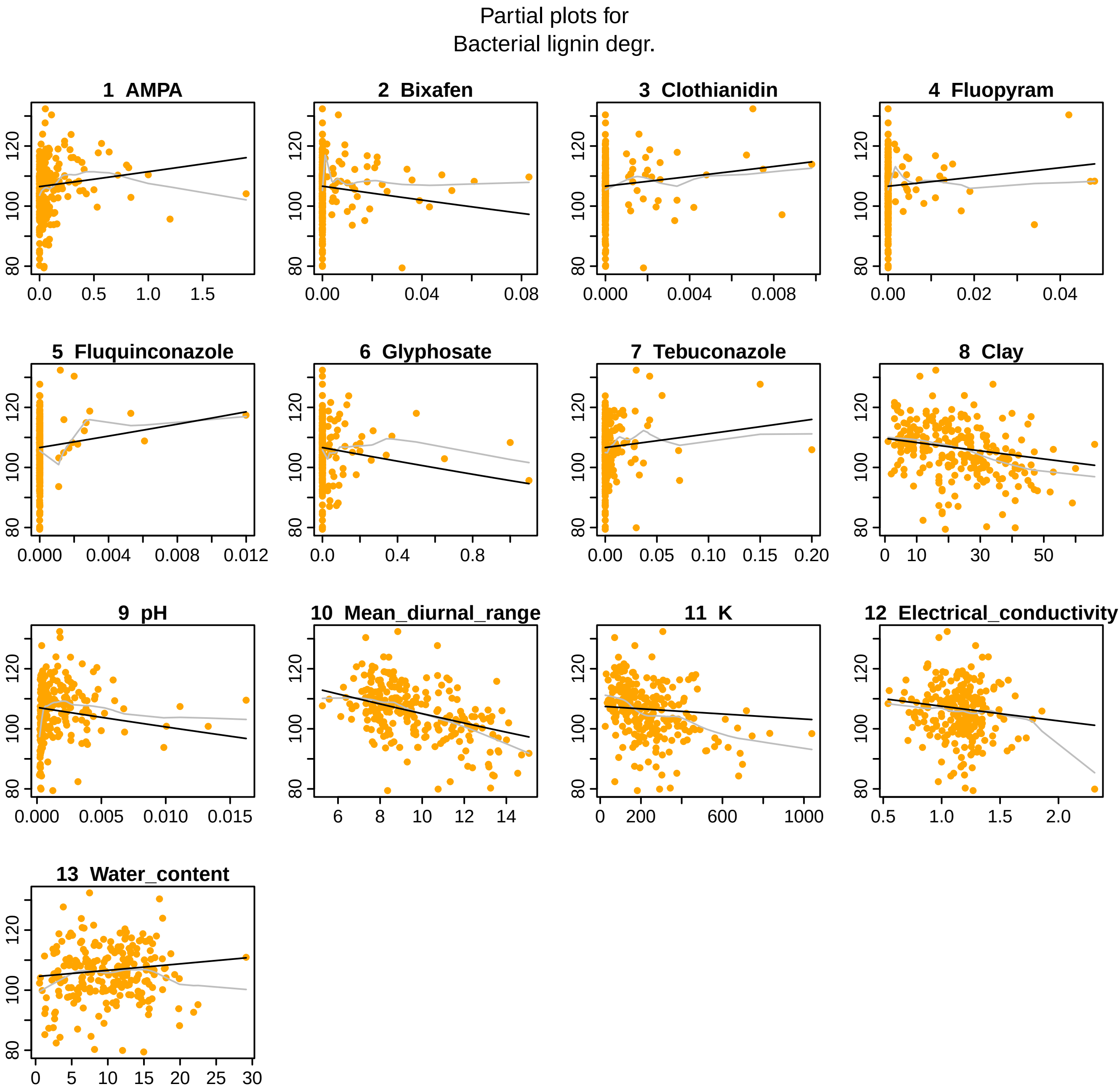

Partial plots for
Bacterial lignin degr.
1 AMPA
2 Bixafen
3 Clothianidin
4 Fluopyram
5 Fluquinconazole
6 Glyphosate
7 Tebuconazole
8 Clay
9 pH
10 Mean_diurnal_range
11 K
12 Electrical_conductivity
13 Water_content

Partial plots for
Bacterial chitin degr.

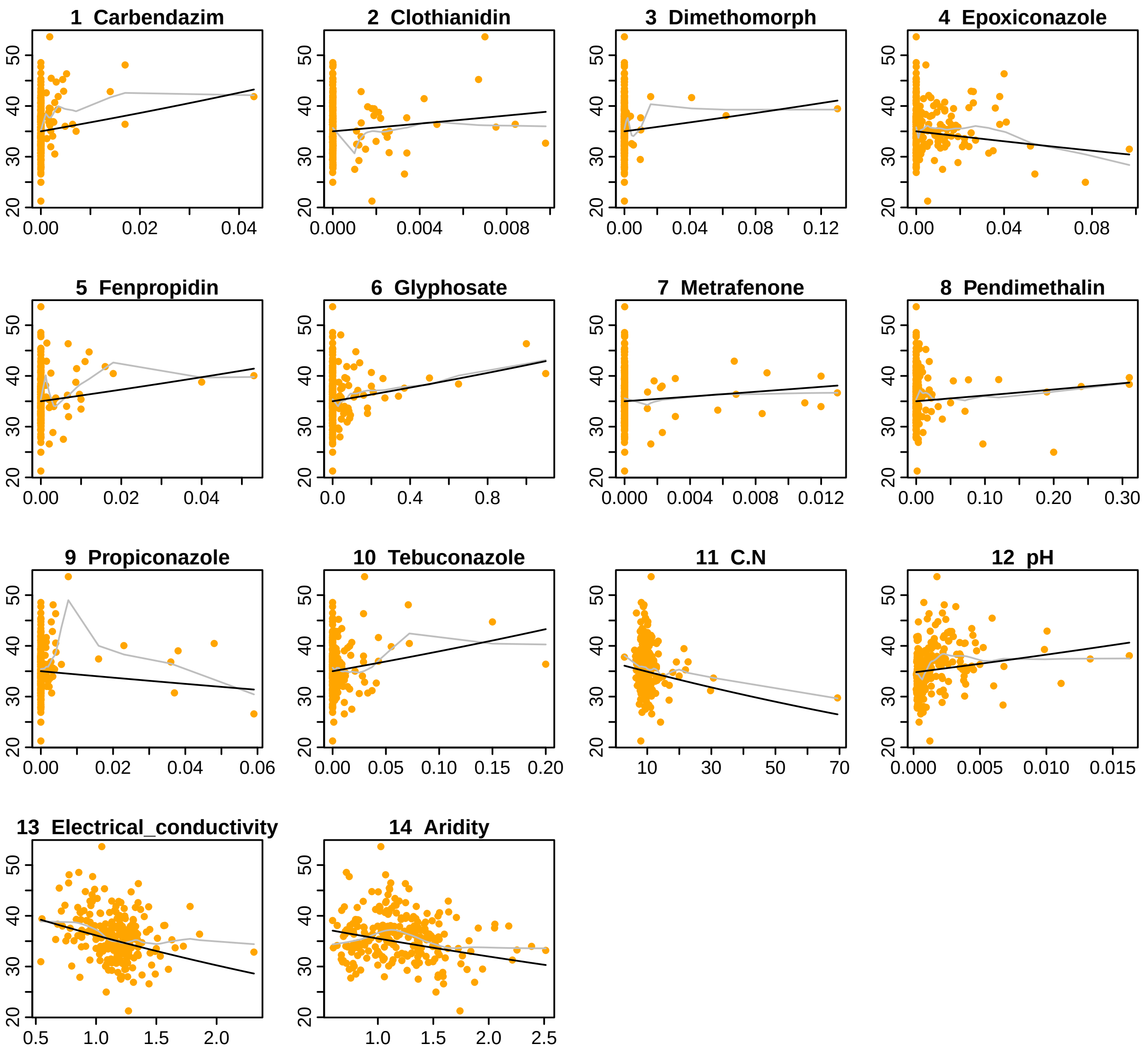

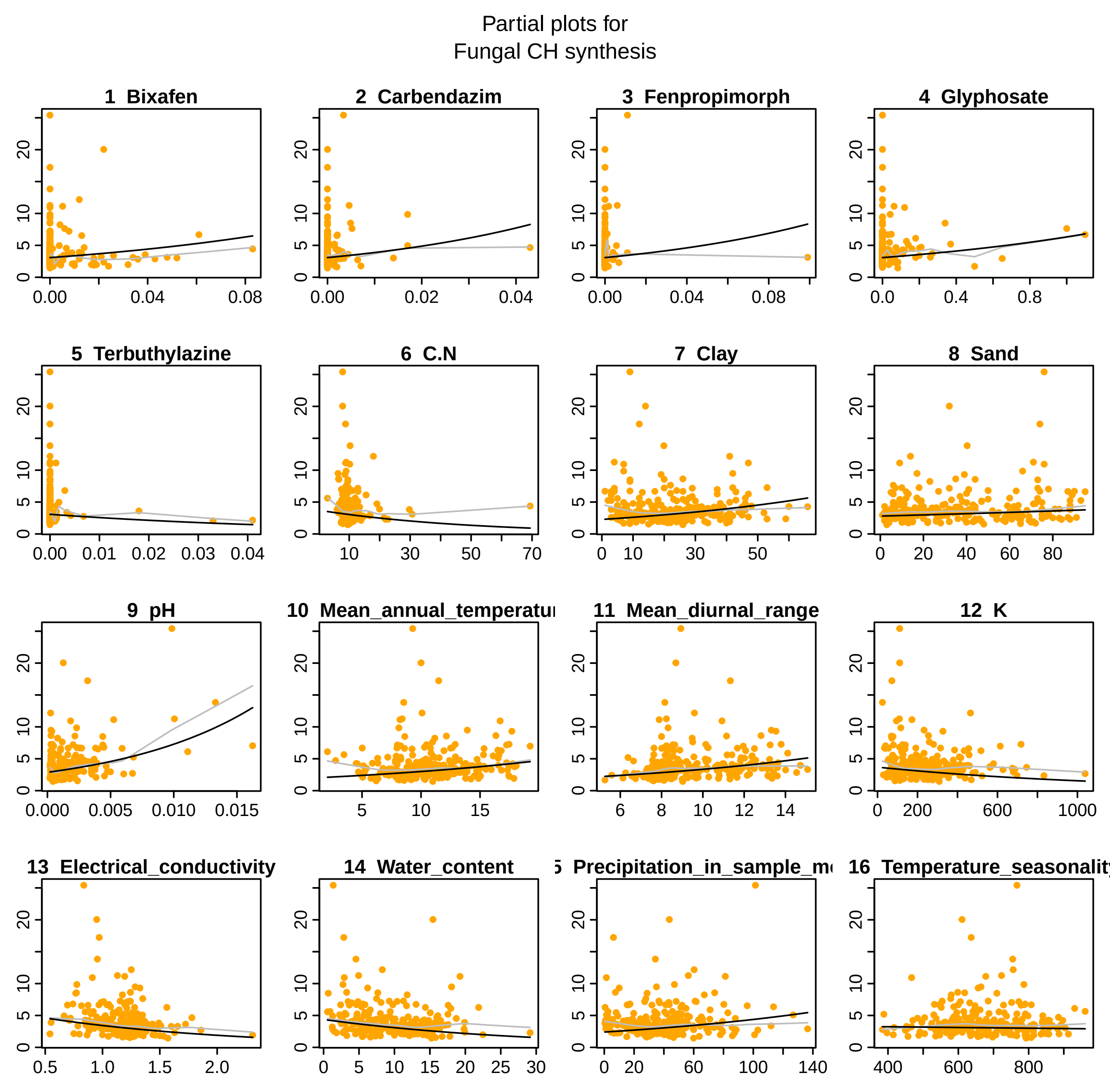
Partial plots for
Fungal CH synthesis
1 Bixafen
2 Carbendazim
3 Fenpropimorph
4 Glyphosate
5 Terbuthylazine
6 C.N
7 Clay
8 Sand
9 pH
10 Mean_annual_temperatu
11 Mean_diurnal_range
12 K
13 Electrical_conductivity
14 Water_content
5 Precipitation_in_sample_m
16 Temperature_seasonality
0
5
10
20
0.00
0.04
0.08
0.02
0.0
0.4
0.8
0.01
0.03
10
30
50
70
0
20
40
60
80
0.000
0.005
0.010
0.015
5
15
6
8
12
14
200
600
1000
0.5
1.0
1.5
2.0
25
100
140
400
800

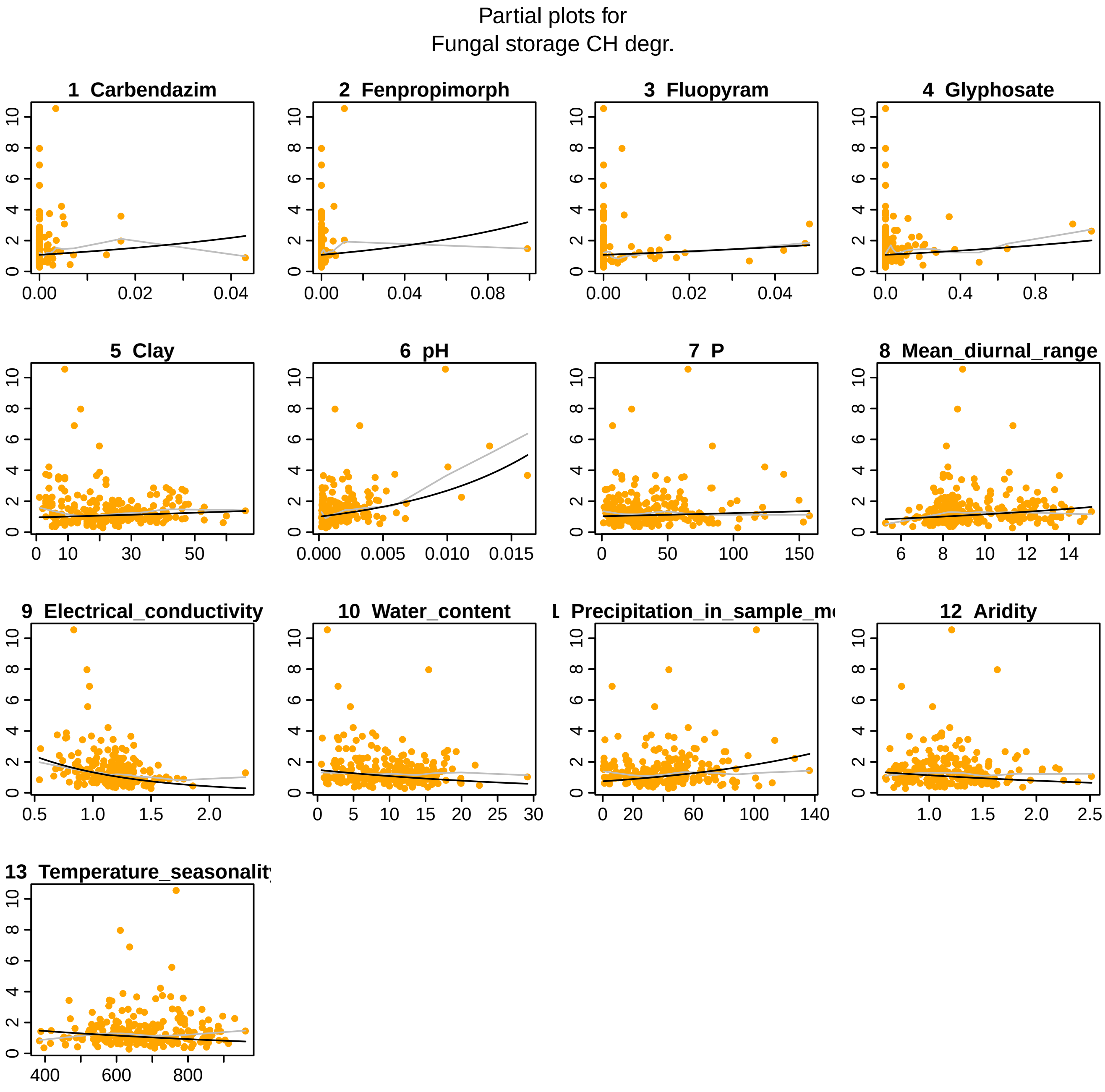
Partial plots for
Fungal storage CH degr.
1 Carbendazim
2 Fenpropimorph
3 Fluopyram
4 Glyphosate
5 Clay
6 pH
7 P
8 Mean_diurnal_range
9 Electrical_conductivity
10 Water_content
Precipitation_in_sample_m
12 Aridity
13 Temperature_seasonalit
0 2 4 6 8 10
0.00 0.02 0.04
0.00 0.04 0.08
0.00 0.02 0.04
0.0 0.4 0.8
0 10 30 50
0.000 0.005 0.010 0.015
0 50 100 150
6 8 10 12 14
0.5 1.0 1.5 2.0
0 5 10 15 20 25 30
0 20 60 100 140
1.0 1.5 2.0 2.5
400 600 800

Partial plots for
Fungal hemicellulose degr.

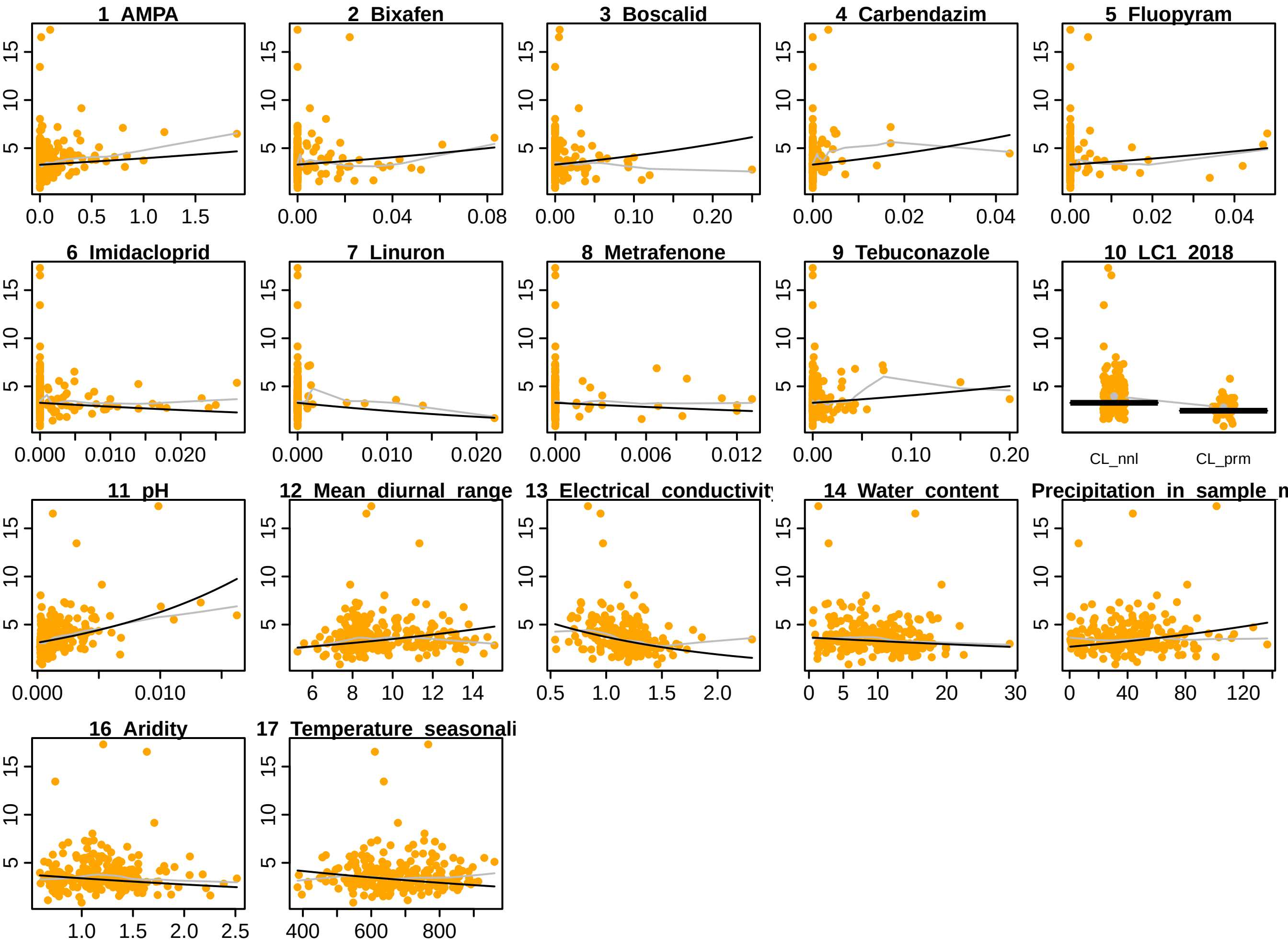

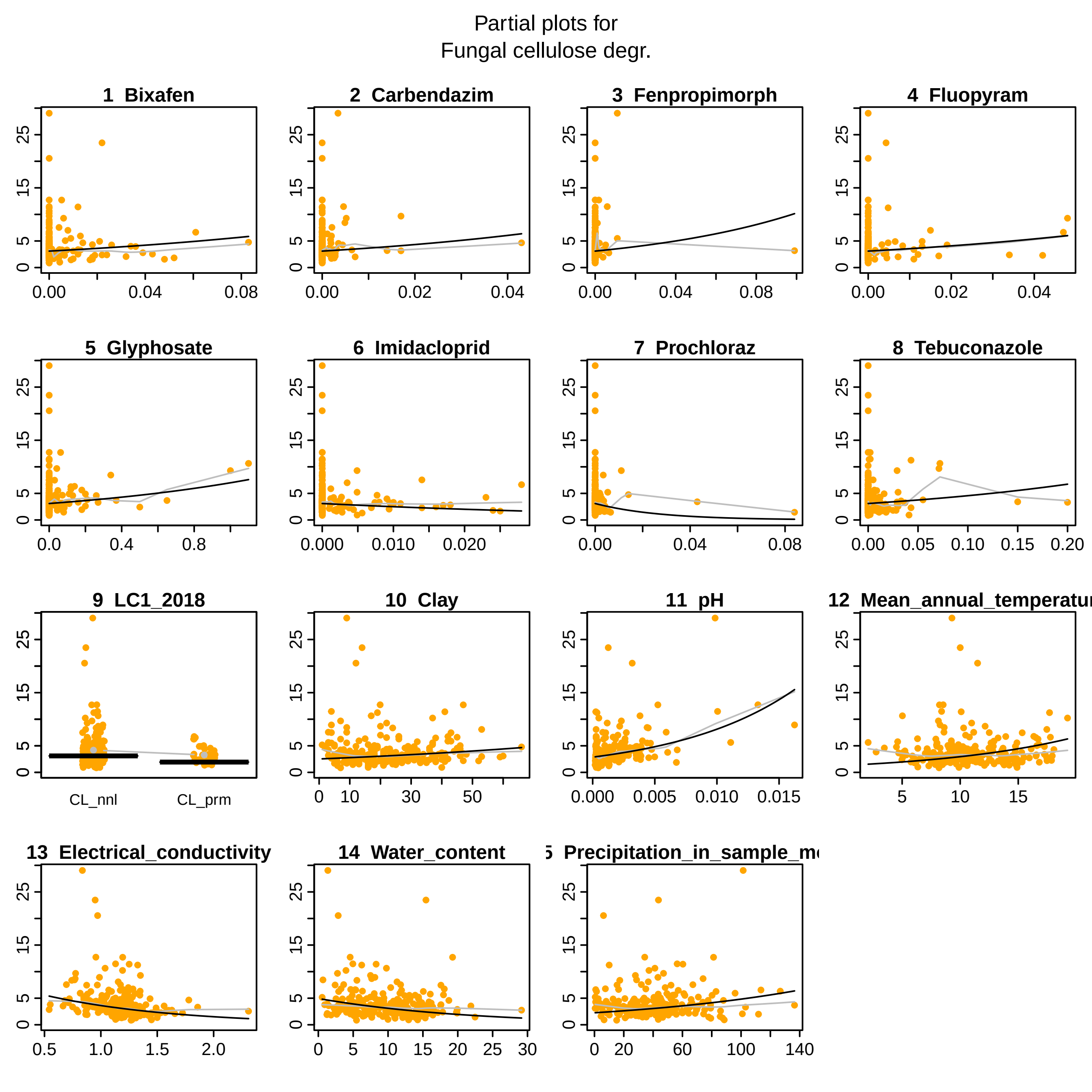

Partial plots for
Fungal cellulose degr.
1 Bixafen
2 Carbendazim
3 Fenpropimorph
4 Fluopyram
5 Glyphosate
6 Imidacloprid
7 Prochloraz
8 Tebuconazole
9 LC1_2018
CL_nnl
CL_prm
10 Clay
11 pH
12 Mean_annual_temperatur
13 Electrical_conductivity
14 Water_content
5 Precipitation_in_sample_m

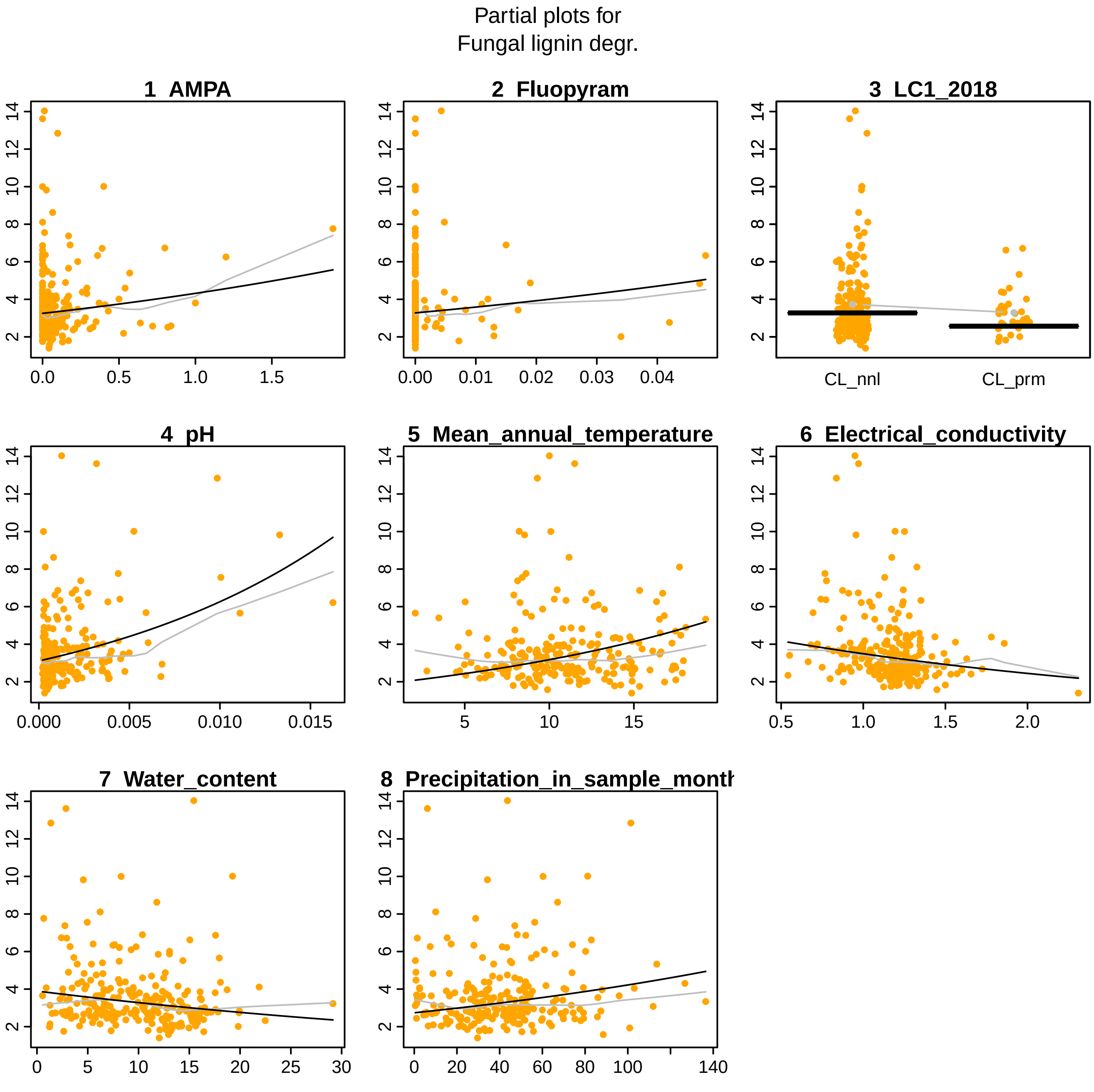
Partial plots for
Fungal lignin degr.
1 AMPA
2 Fluopyram
3 LC1_2018
CL_nnl
CL_prm
4 pH
5 Mean_annual_temperature
6 Electrical_conductivity
7 Water_content
8 Precipitation_in_sample_montl

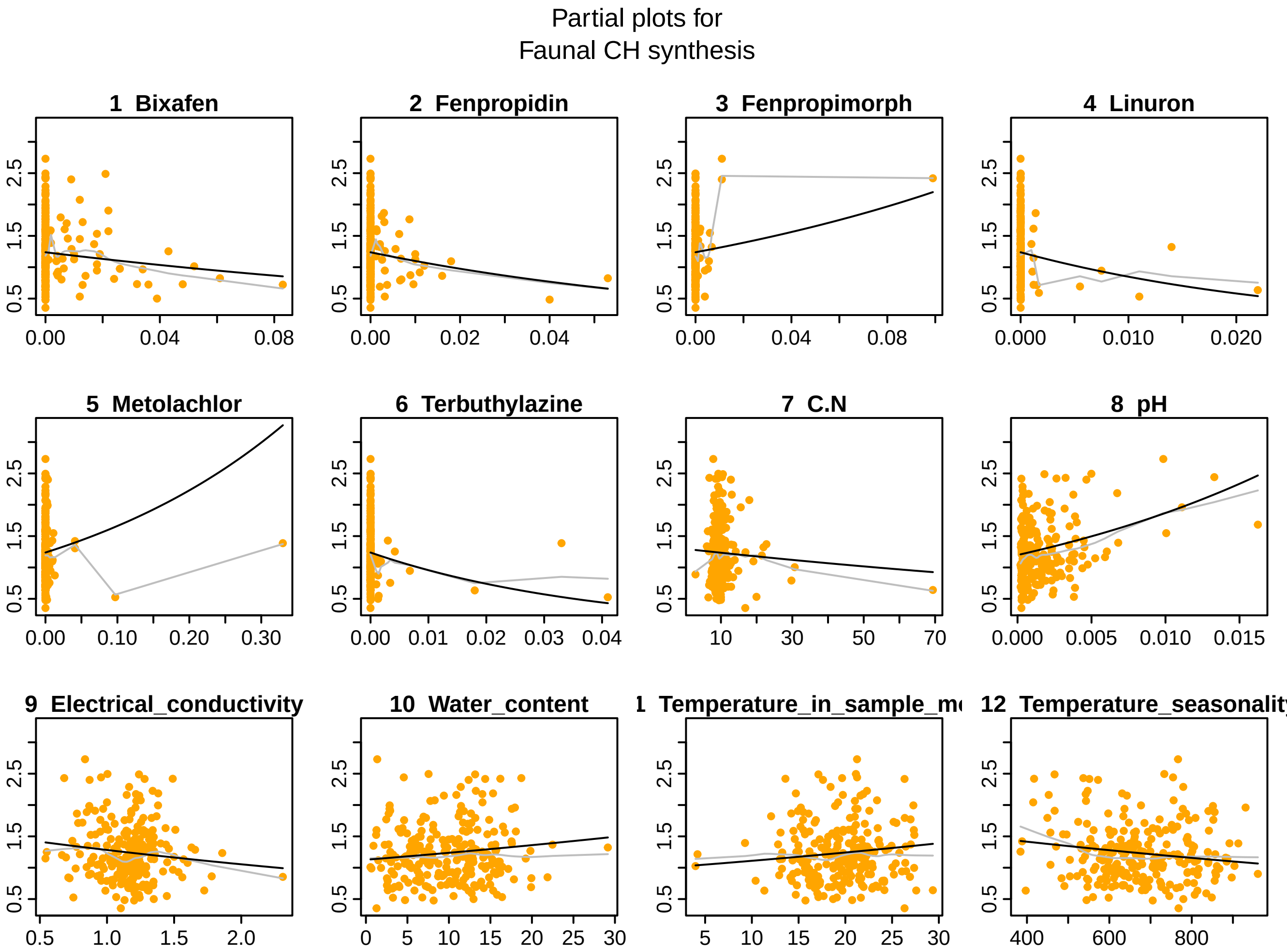

Partial plots for
Faunal CH synthesis
1 Bixafen
2 Fenpropidin
3 Fenpropimorph
4 Linuron
5 Metolachlor
6 Terbuthylazine
7 C.N
8 pH
9 Electrical_conductivity
10 Water_content
1 Temperature_in_sample_m
12 Temperature_seasonalit

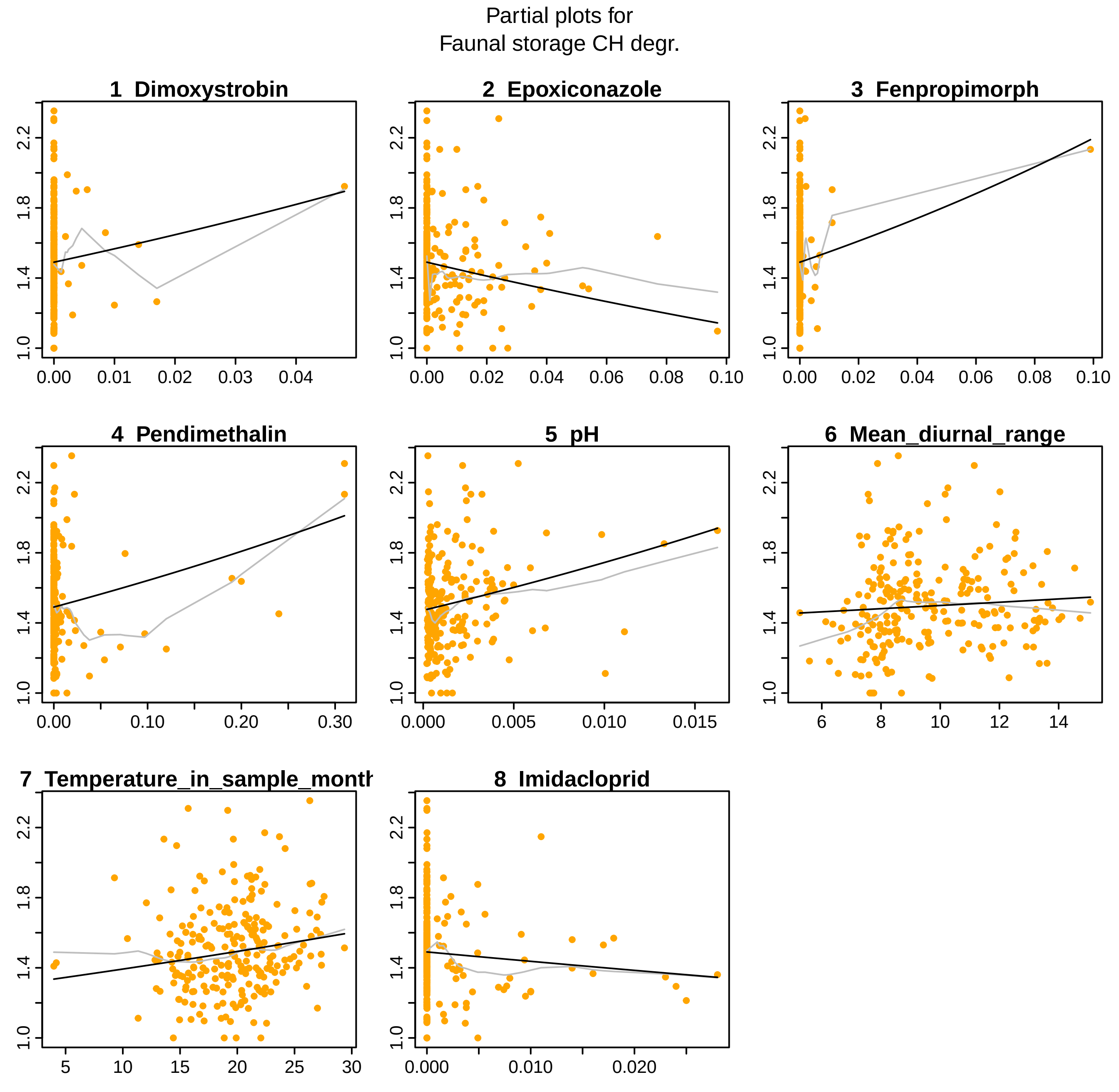

Partial plots for
Faunal storage CH degr.
1 Dimoxystrobin
2 Epoxiconazole
3 Fenpropimorph
4 Pendimethalin
5 pH
6 Mean_diurnal_range
7 Temperature_in_sample_month
8 Imidacloprid

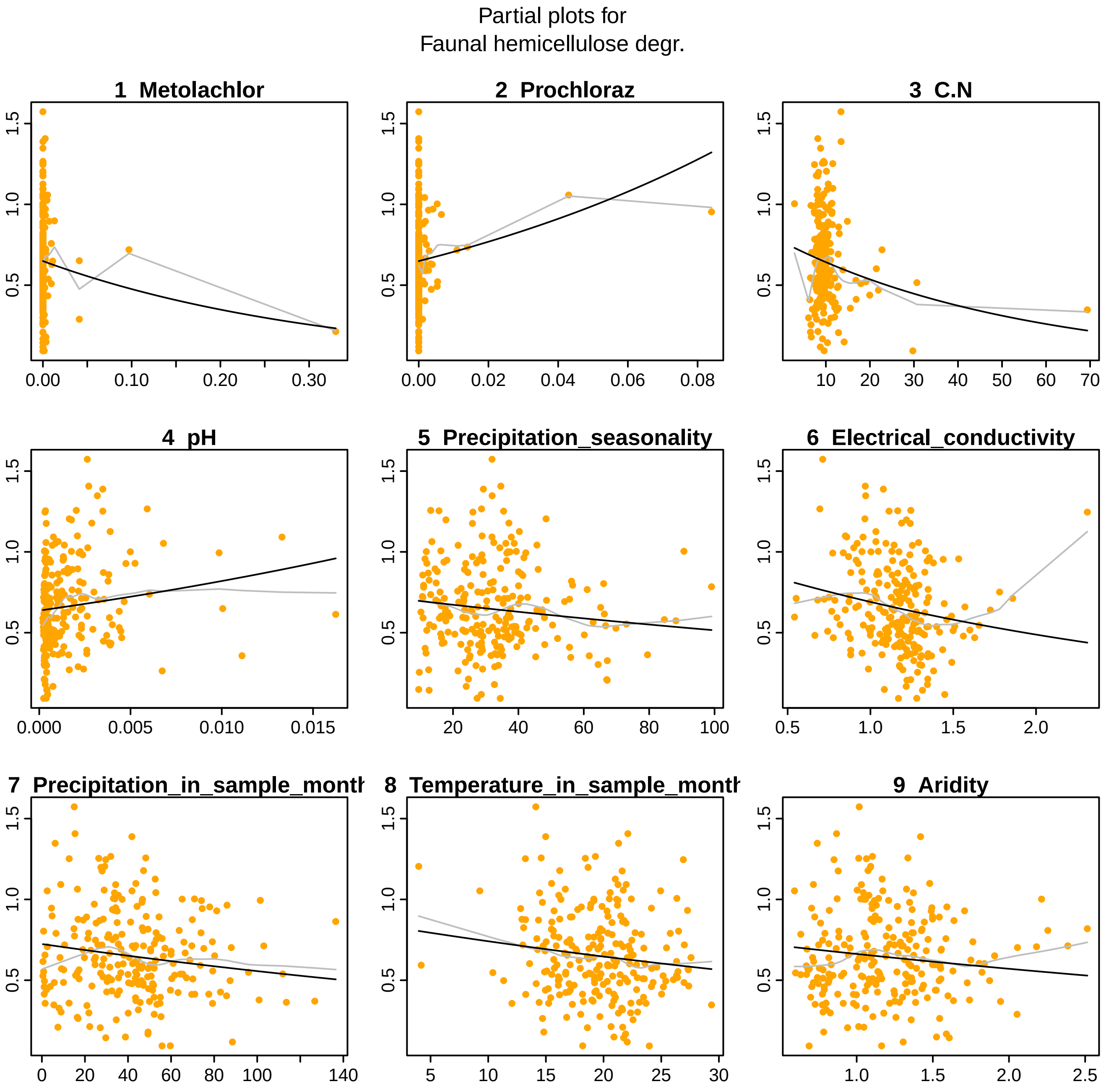
Partial plots for
Faunal hemicellulose degr.
1 Metolachlor
2 Prochloraz
3 C.N
4 pH
5 Precipitation_seasonality
6 Electrical_conductivity
7 Precipitation_in_sample_montl
8 Temperature_in_sample_montl
9 Aridity

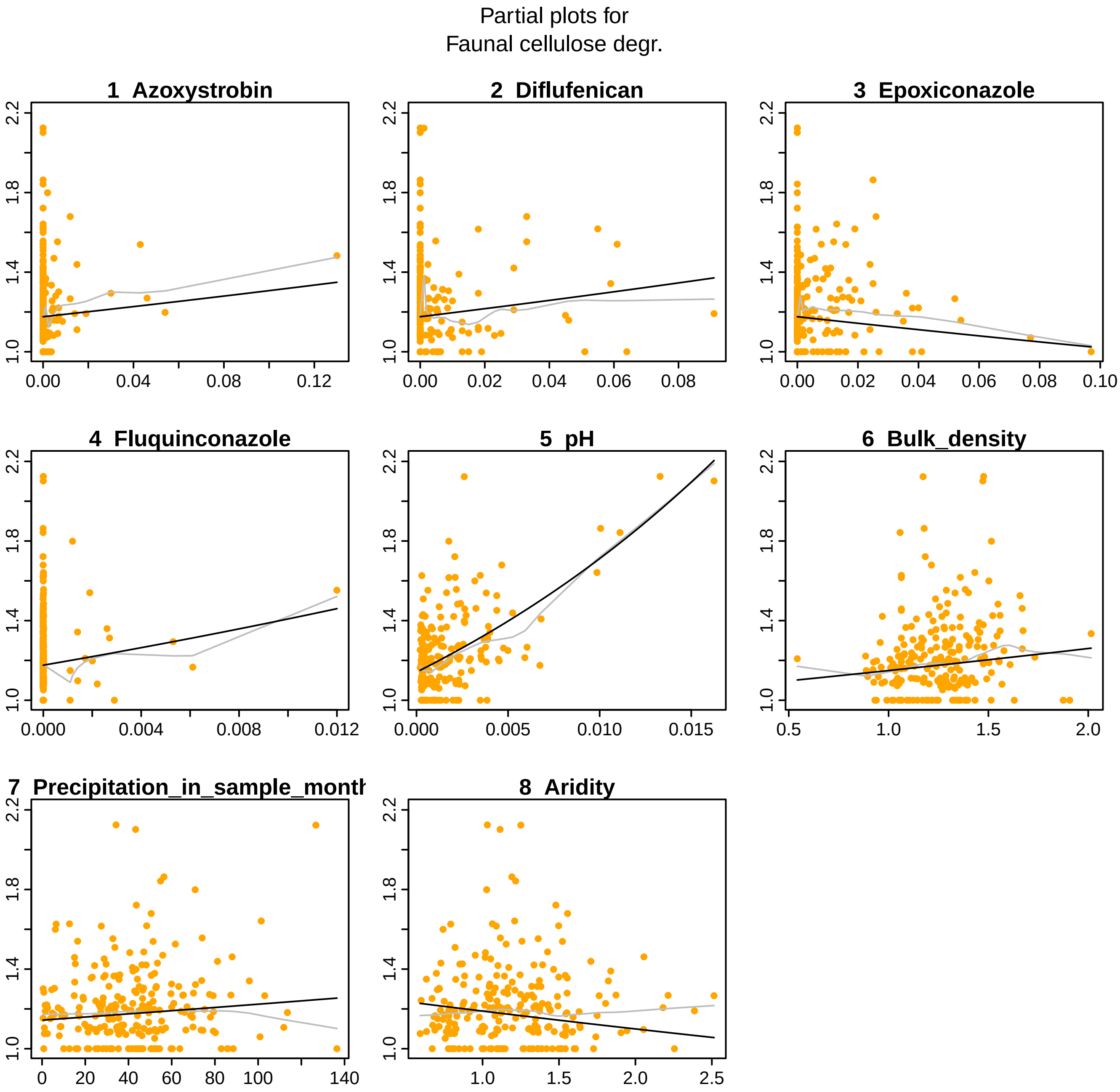

Partial plots for
Faunal cellulose degr.
1 Azoxystrobin
2 Diflufenican
3 Epoxiconazole
4 Fluquinconazole
5 pH
6 Bulk_density
7 Precipitation_in_sample_montl
8 Aridity
1.0
1.4
1.8
2.2
0.00
0.04
0.08
0.12
0.02
0.06
0.10
0.000
0.004
0.008
0.012
0.005
0.010
0.015
0.5
1.0
1.5
2.0
2.5
0
20
40
60
80
100
140

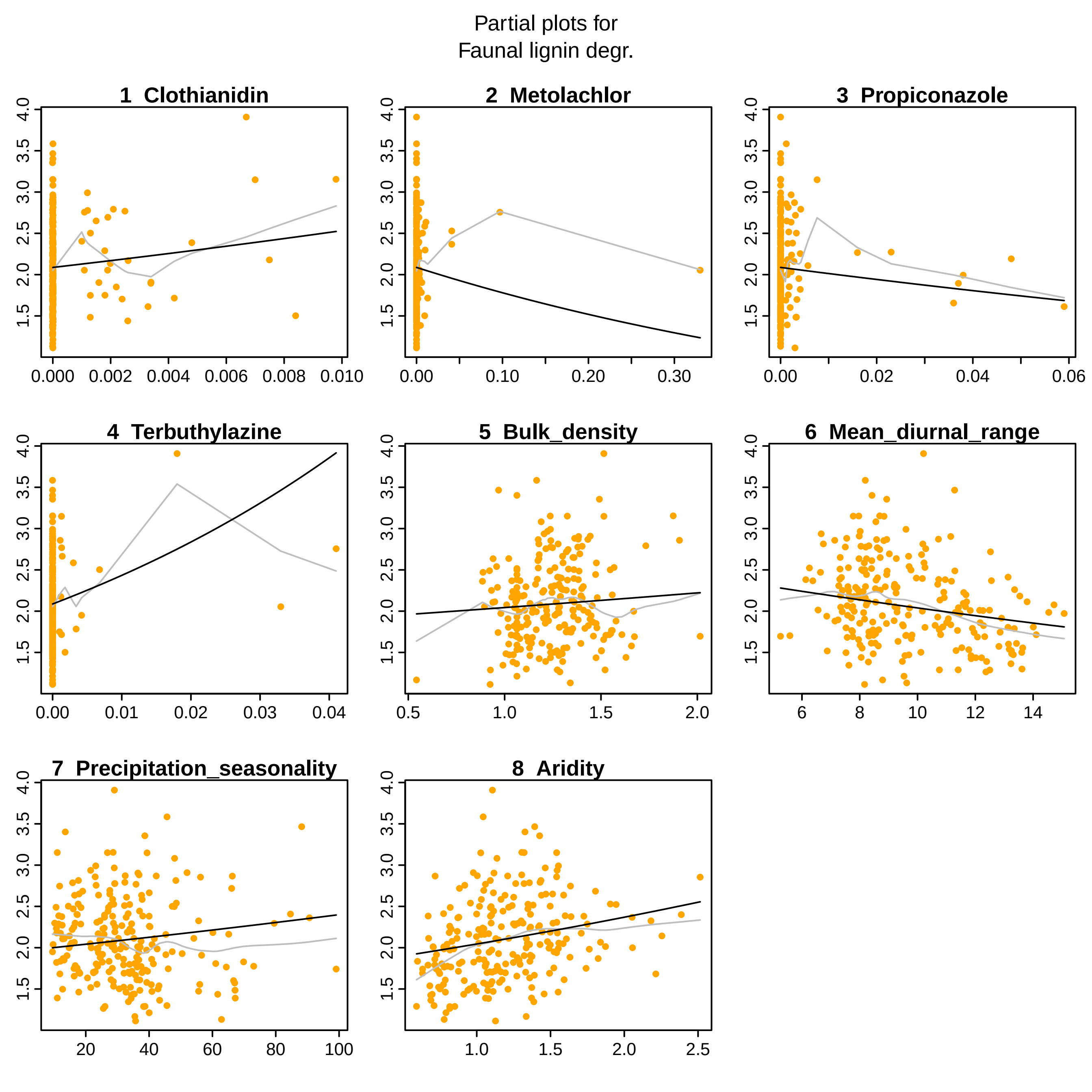

Partial plots for
Faunal lignin degr.
1 Clothianidin
2 Metolachlor
3 Propiconazole
4 Terbuthylazine
5 Bulk_density
6 Mean_diurnal_range
7 Precipitation_seasonality
8 Aridity

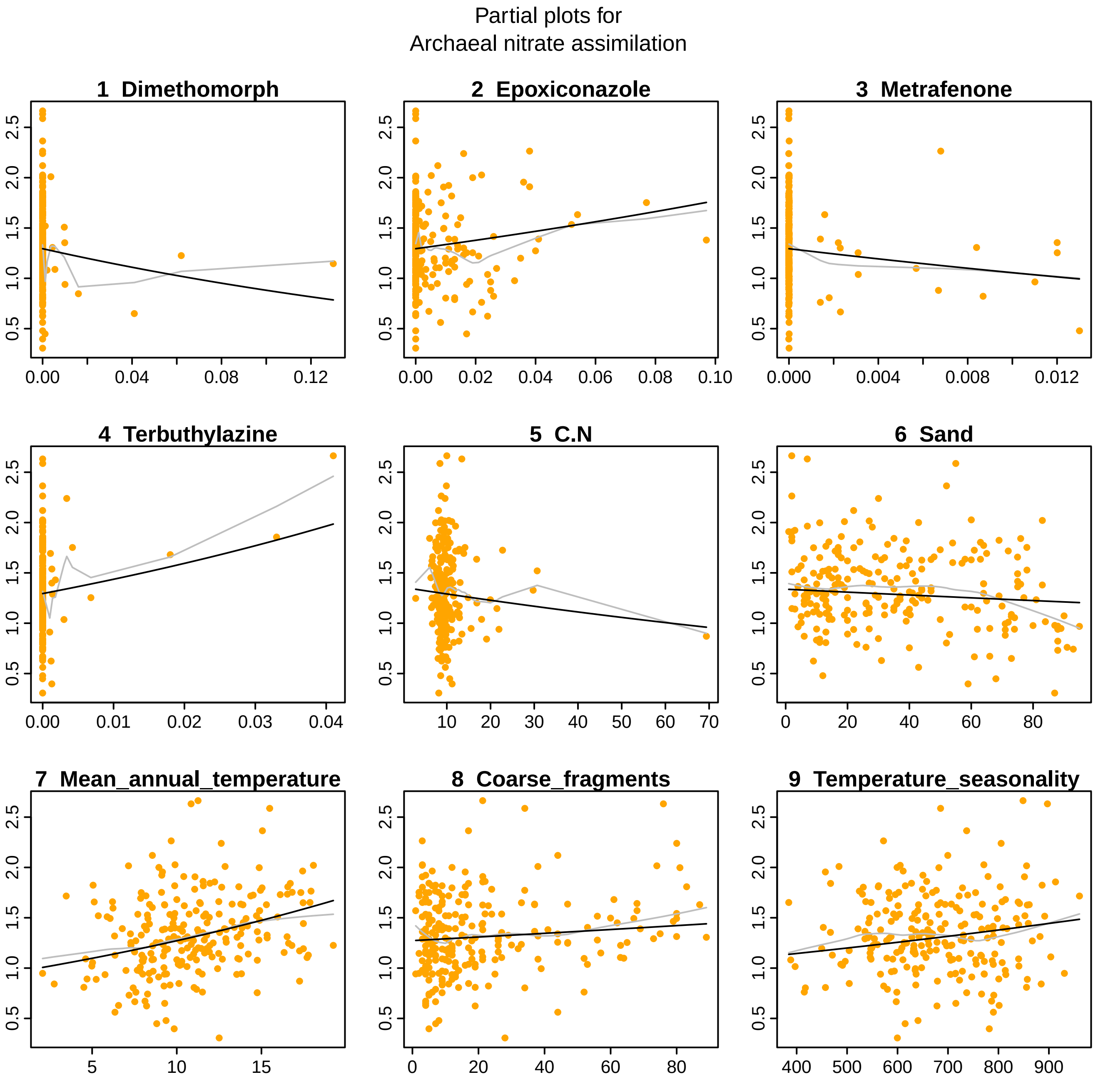
Partial plots for
Archaeal nitrate assimilation
1 Dimethomorph
2 Epoxiconazole
3 Metrafenone
4 Terbuthylazine
5 C.N
6 Sand
7 Mean_annual_temperature
8 Coarse_fragments
9 Temperature_seasonality

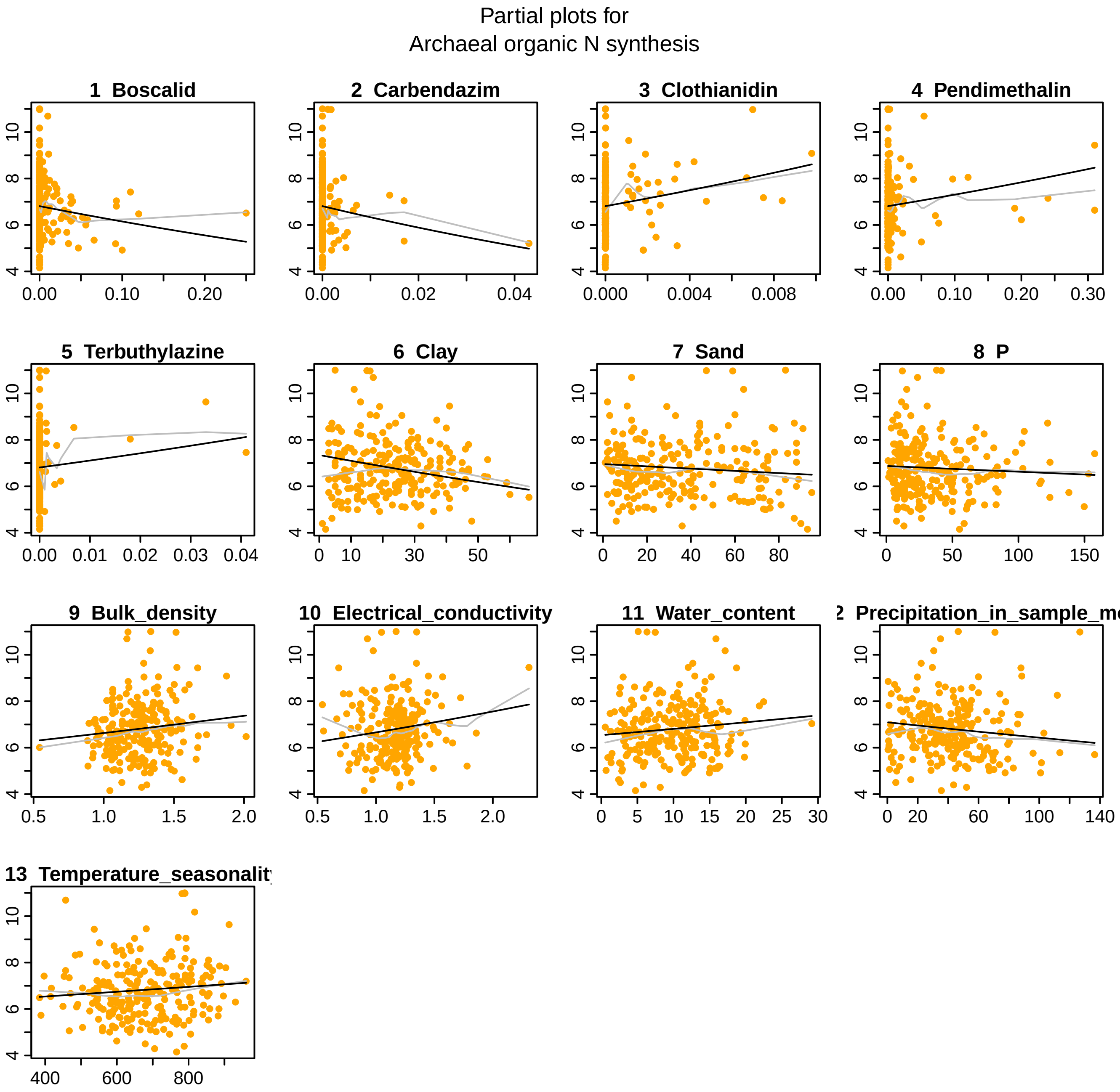
Partial plots for
Archaeal organic N synthesis
1 Boscalid
2 Carbendazim
3 Clothianidin
4 Pendimethalin
5 Terbuthylazine
6 Clay
7 Sand
8 P
9 Bulk_density
10 Electrical_conductivity
11 Water_content
2 Precipitation_in_sample_m
13 Temperature_seasonalit
0.00
0.10
0.20
0.02
0.04
0.000
0.004
0.008
0.30
0.01
0.03
10
30
50
20
40
60
80
100
150
0.5
1.0
1.5
2.0
5
15
25
140
400
600
800
4
6
8

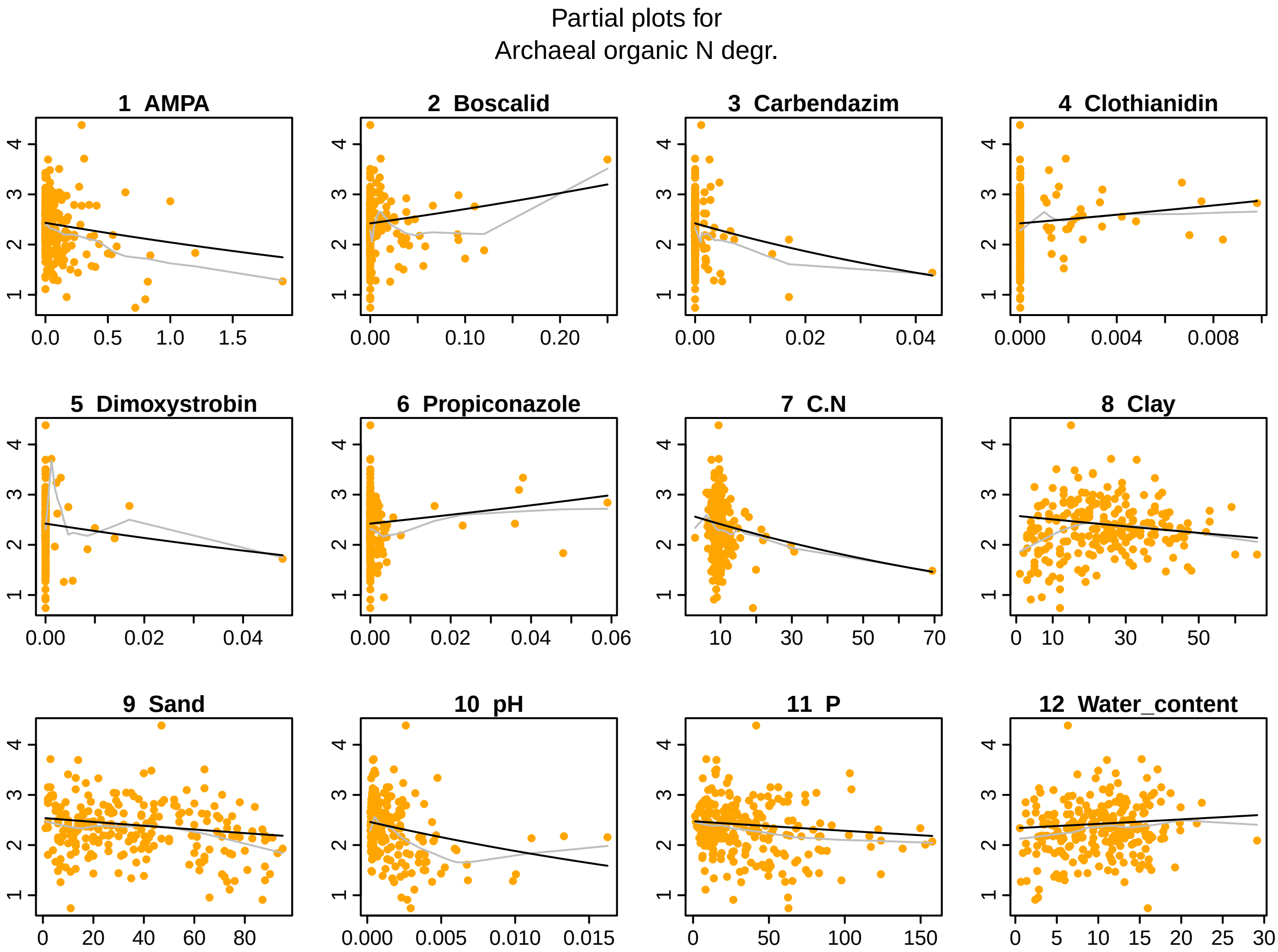
Partial plots for
Archaeal organic N degr.
1 AMPA
2 Boscalid
3 Carbendazim
4 Clothianidin
5 Dimoxystrobin
6 Propiconazole
7 C.N
8 Clay
9 Sand
10 pH
11 P
12 Water_content

Partial plots for
Archaeal ammonia oxidation

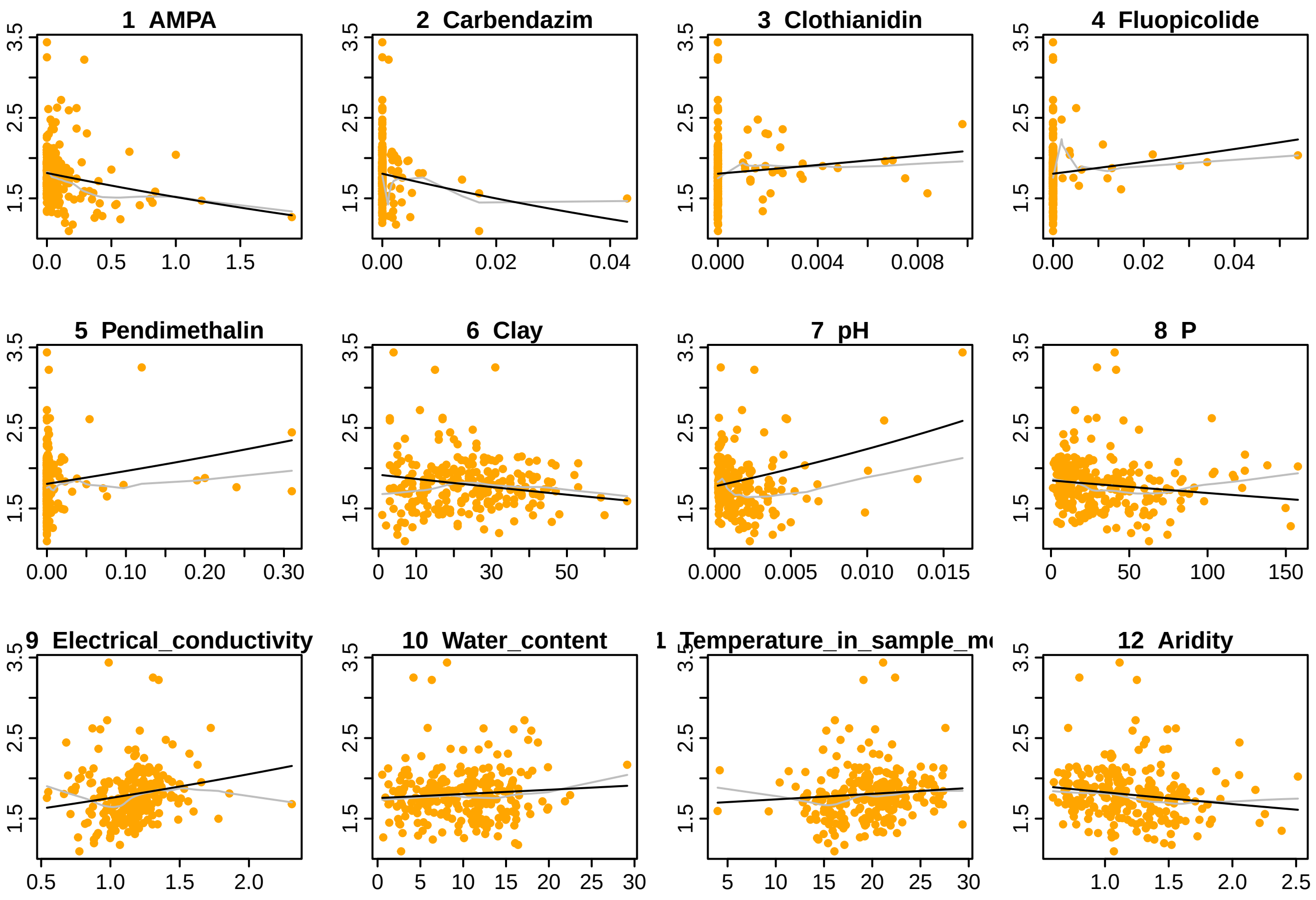

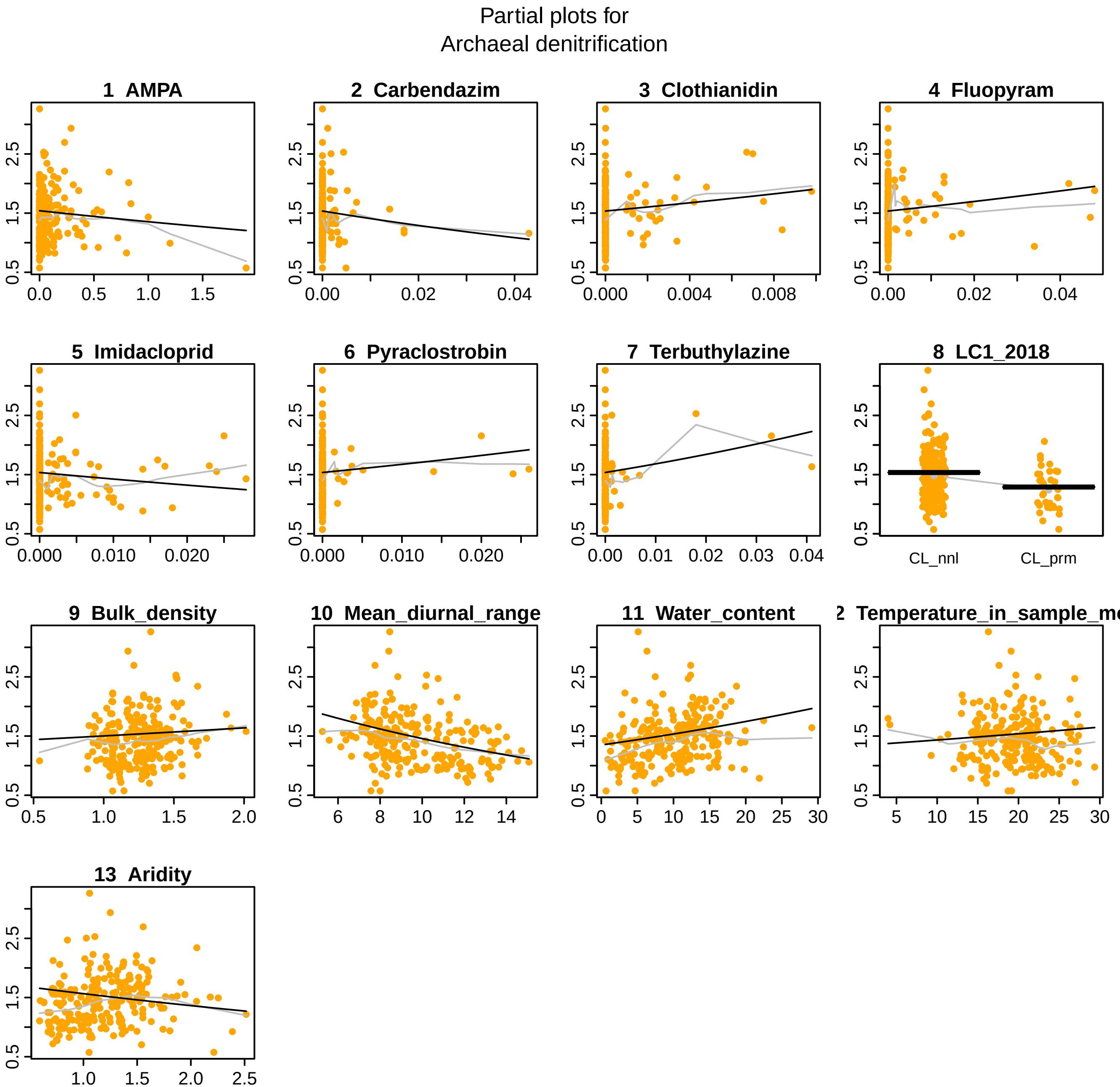
Partial plots for
Archaeal denitrification
1 AMPA
2 Carbendazim
3 Clothianidin
4 Fluopyram
5 Imidacloprid
6 Pyraclostrobin
7 Terbuthylazine
8 LC1_2018
CL_nnl
CL_prm
9 Bulk_density
10 Mean_diurnal_range
11 Water_content
2 Temperature_in_sample_mo
13 Aridity

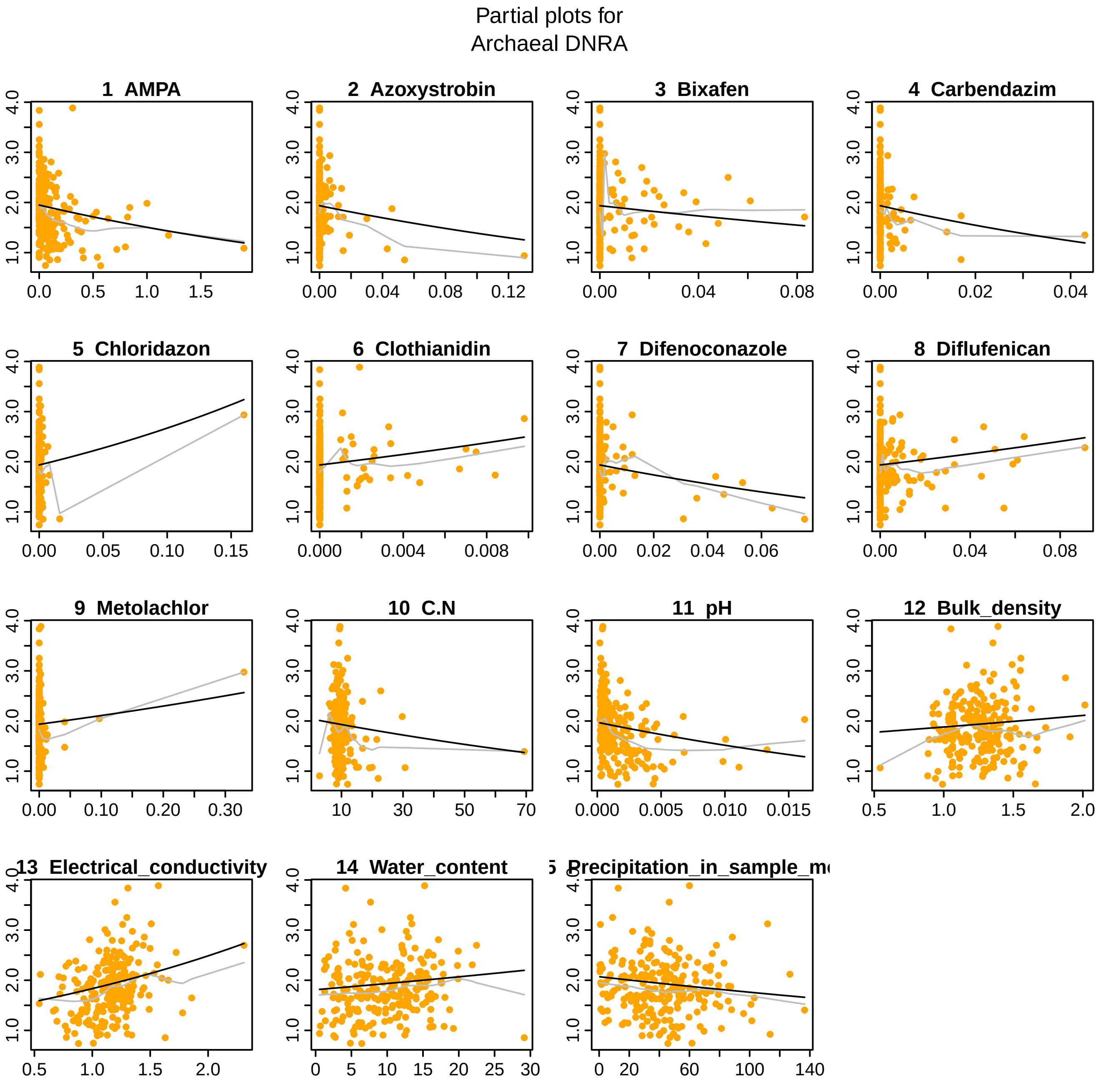

Partial plots for
Archaeal DNRA
1 AMPA
2 Azoxystrobin
3 Bixafen
4 Carbendazim
5 Chloridazon
6 Clothianidin
7 Difenoconazole
8 Diflufenican
9 Metolachlor
10 C.N
11 pH
12 Bulk_density
13 Electrical_conductivity
14 Water_content
Precipitation_in_sample_m

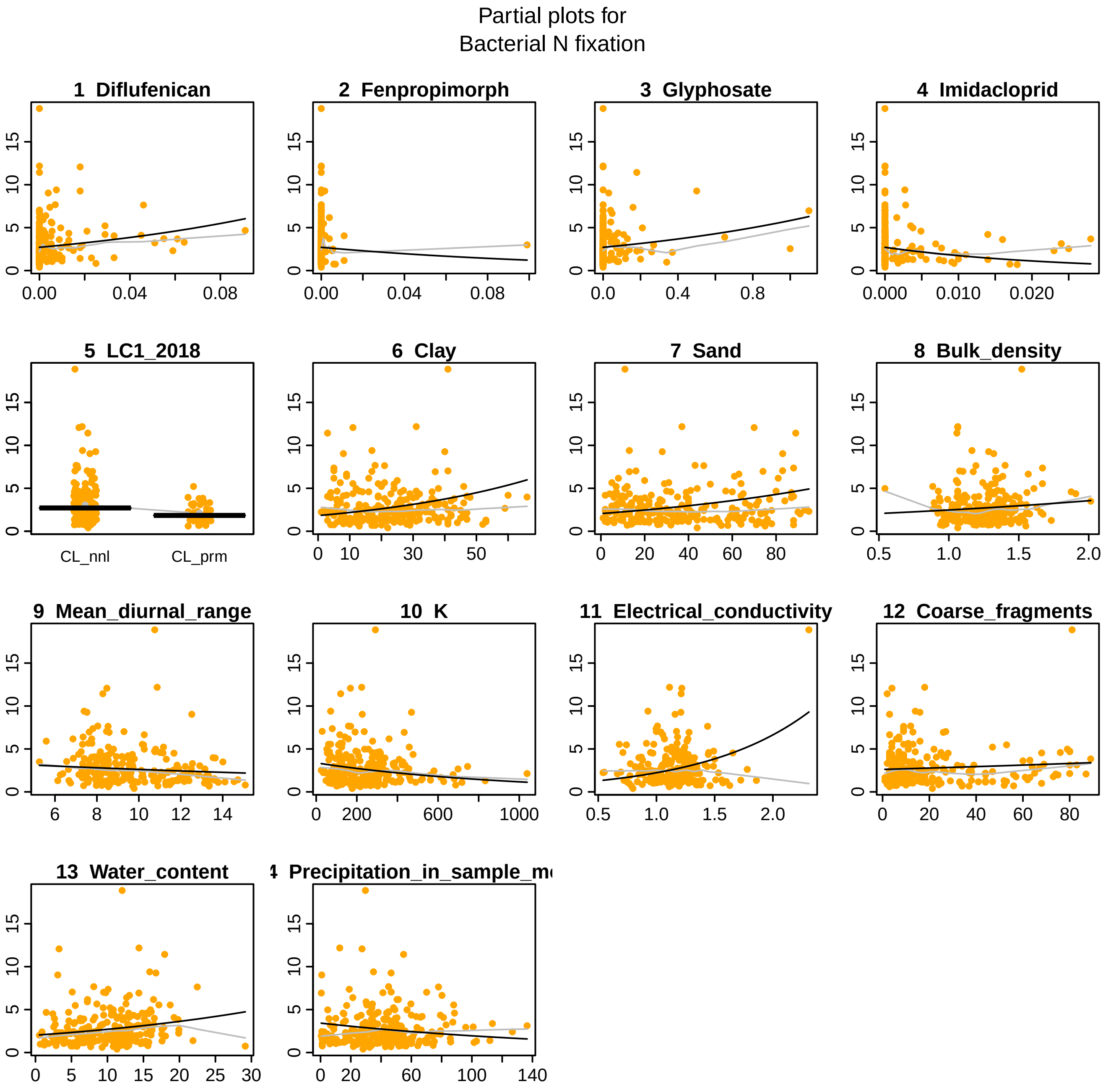
Partial plots for
Bacterial N fixation
1 Diflufenican
2 Fenpropimorph
3 Glyphosate
4 Imidacloprid
5 LC1_2018
6 Clay
7 Sand
8 Bulk_density
9 Mean_diurnal_range
10 K
11 Electrical_conductivity
12 Coarse_fragments
13 Water_content
4 Precipitation_in_sample_m
CL_nnl
CL_prm

Partial plots for
Bacterial nitrate assimilation

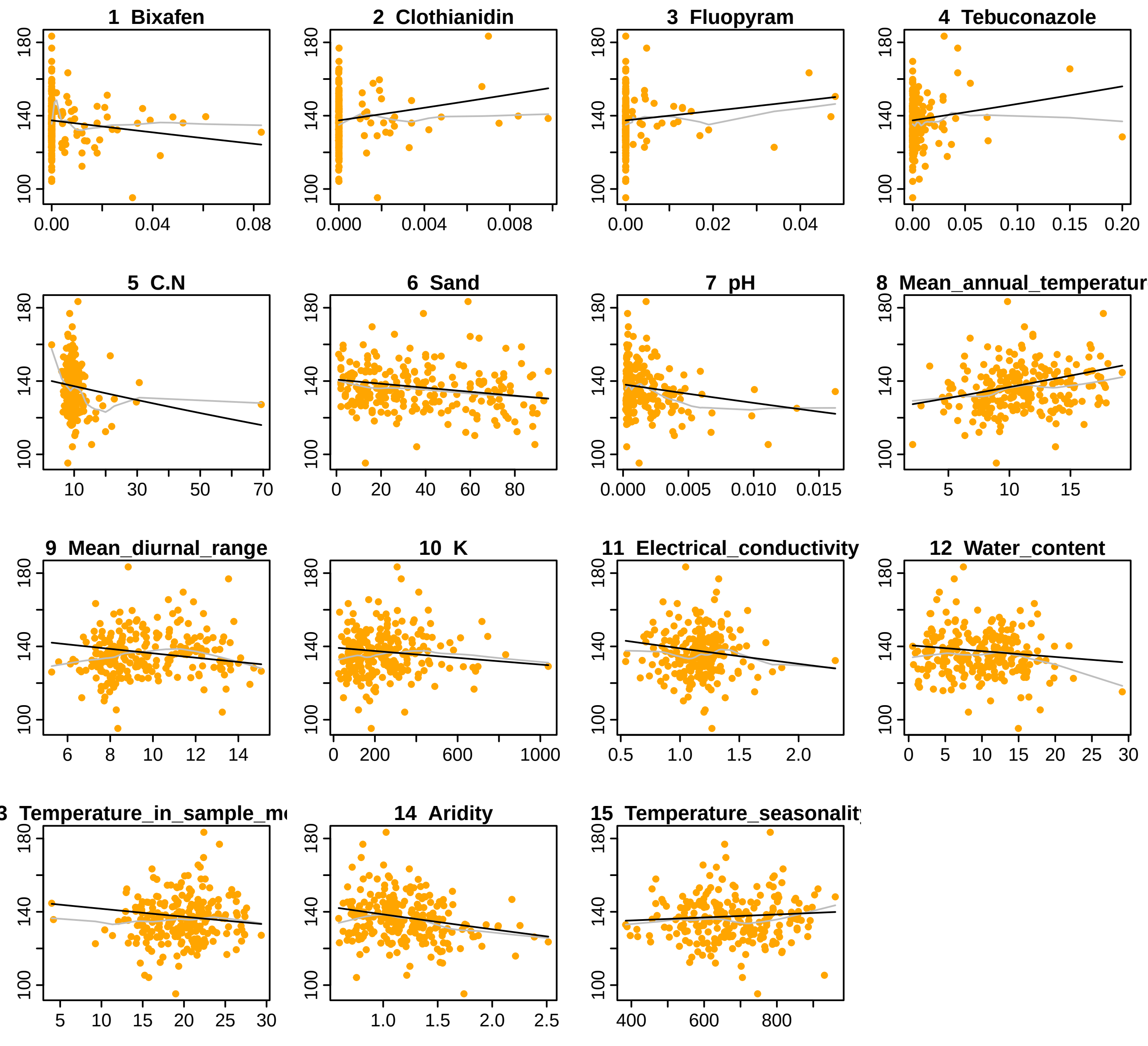

Partial plots for
Bacterial organic N synthesis

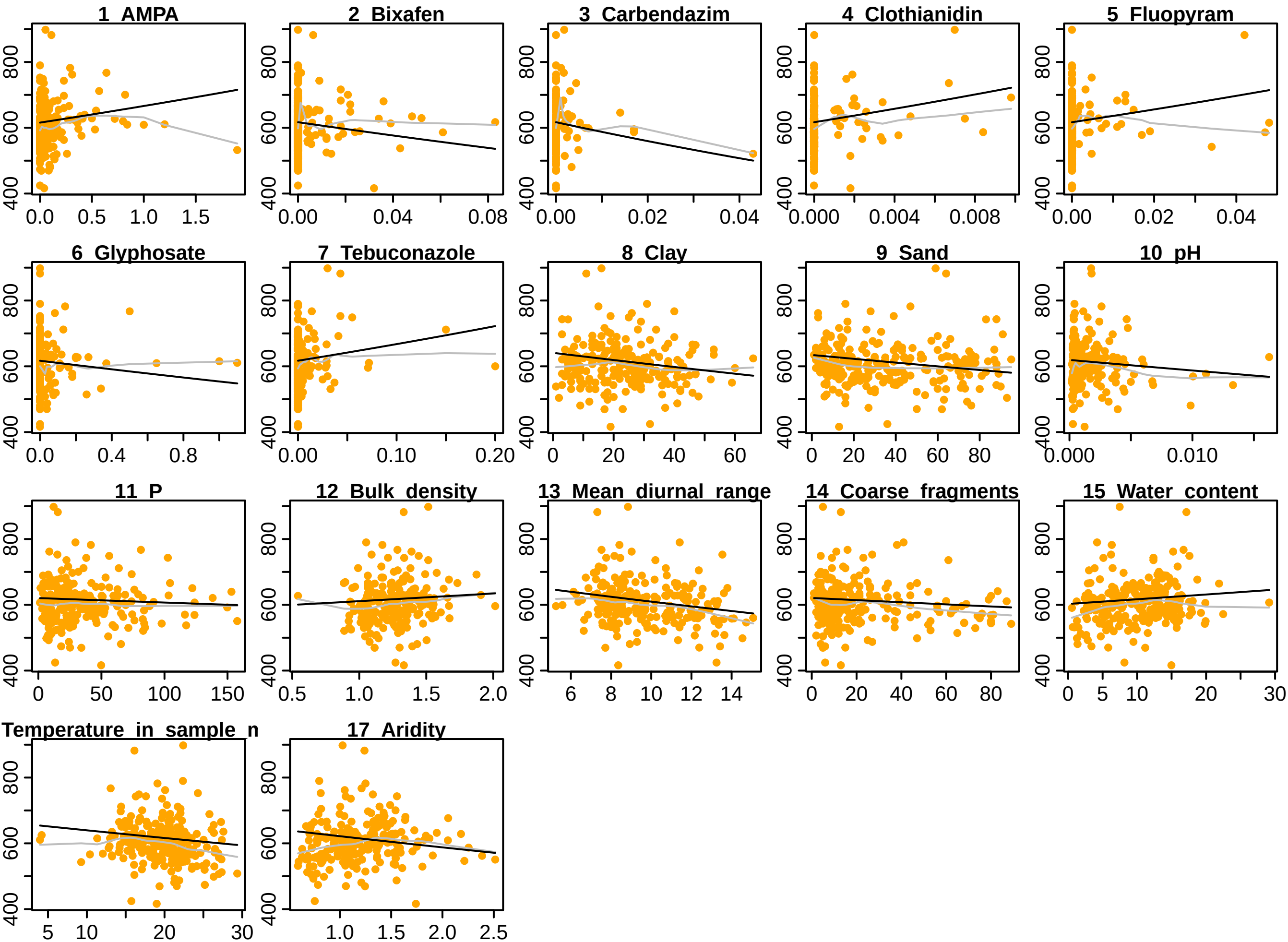

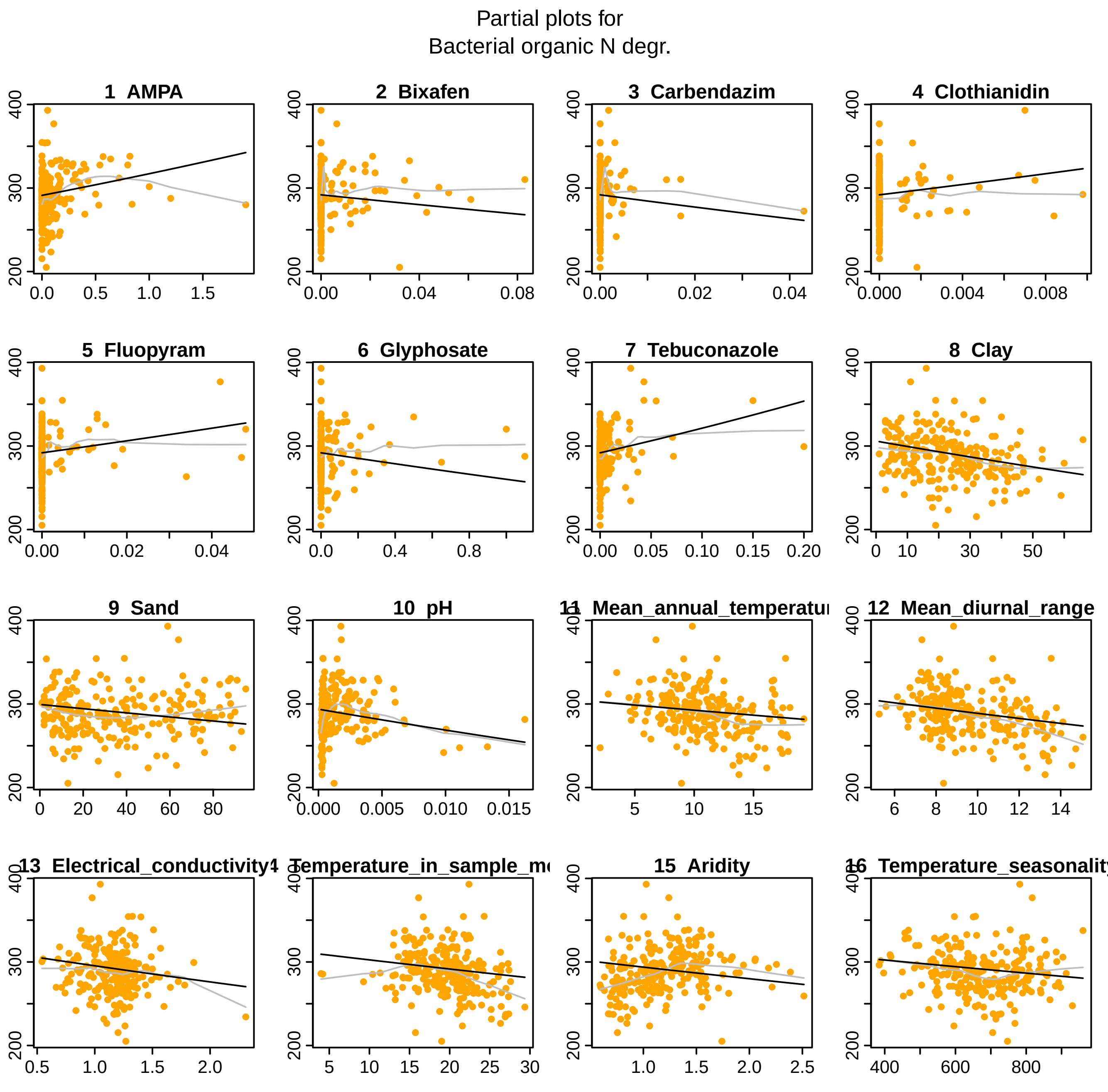
Partial plots for
Bacterial organic N degr.
1 AMPA
2 Bixafen
3 Carbendazim
4 Clothianidin
5 Fluopyram
6 Glyphosate
7 Tebuconazole
8 Clay
9 Sand
10 pH
11 Mean_annual_temperatu
12 Mean_diurnal_range
13 Electrical_conductivity
Temperature_in_sample_m
15 Aridity
16 Temperature_seasonalit

Partial plots for
Bacterial ammonia oxidation

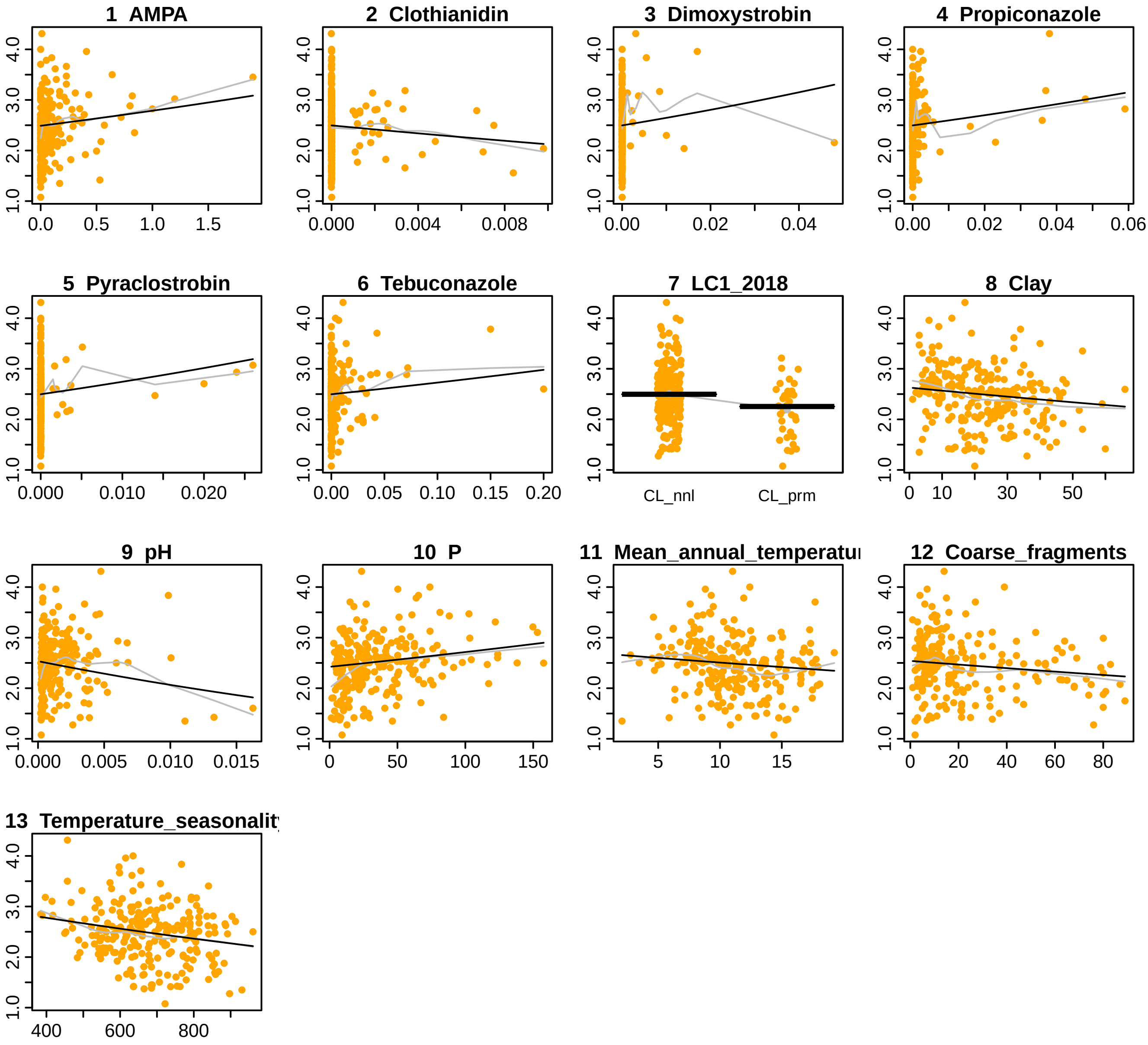

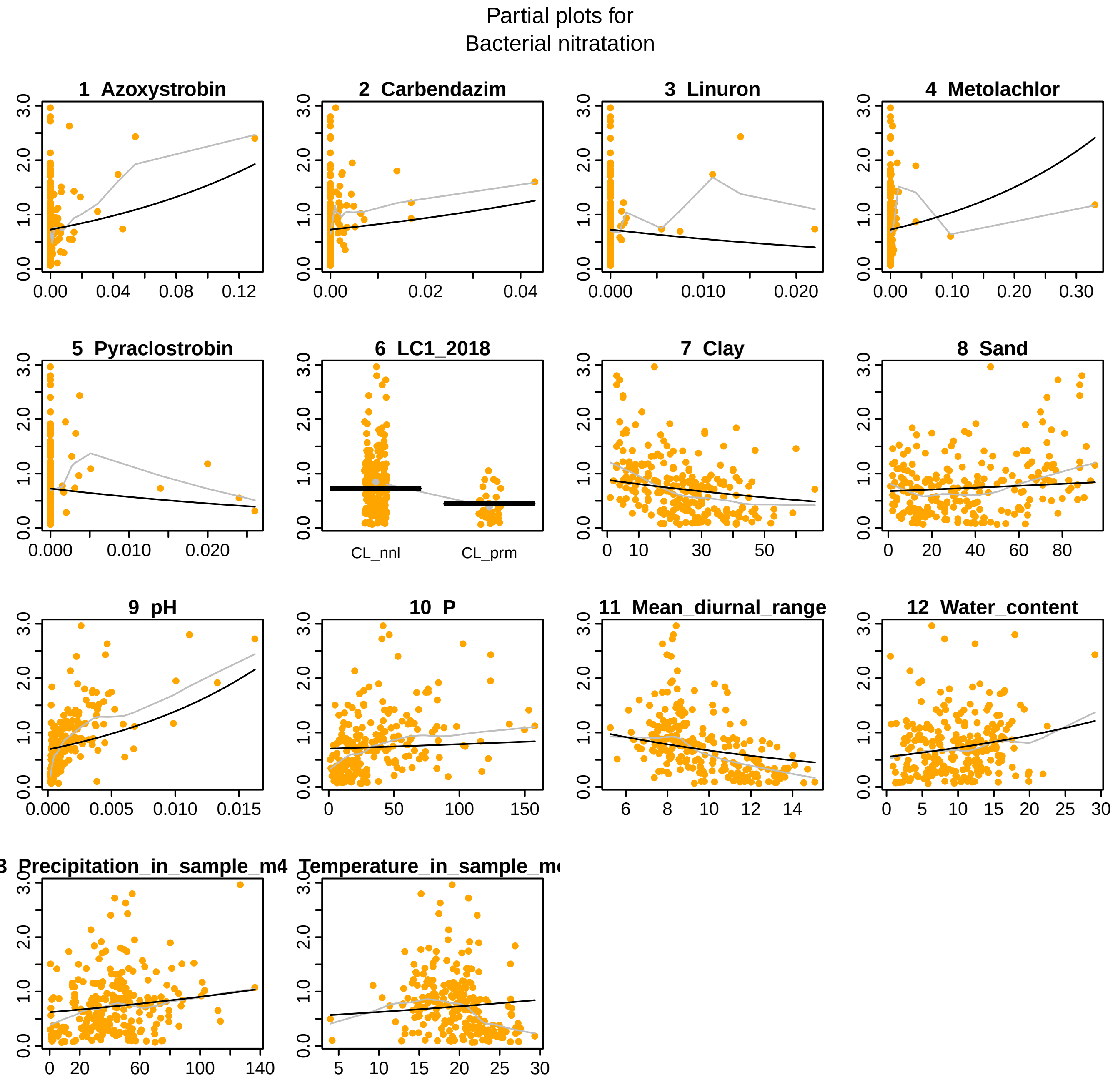
Partial plots for
Bacterial nitratation
1 Azoxystrobin
2 Carbendazim
3 Linuron
4 Metolachlor
5 Pyraclostrobin
6 LC1_2018
CL_nnl
CL_prm
7 Clay
8 Sand
9 pH
10 P
11 Mean_diurnal_range
12 Water_content
3 Precipitation_in_sample_m4
Temperature_in_sample_m
0.0
1.0
2.0
3.0
0.00
0.04
0.08
0.12
0.02
0.000
0.010
0.020
0.10
0.20
0.30
0.005
0.015
0
10
20
30
40
50
60
80
100
140
150
5
15
25
6
8
12
14

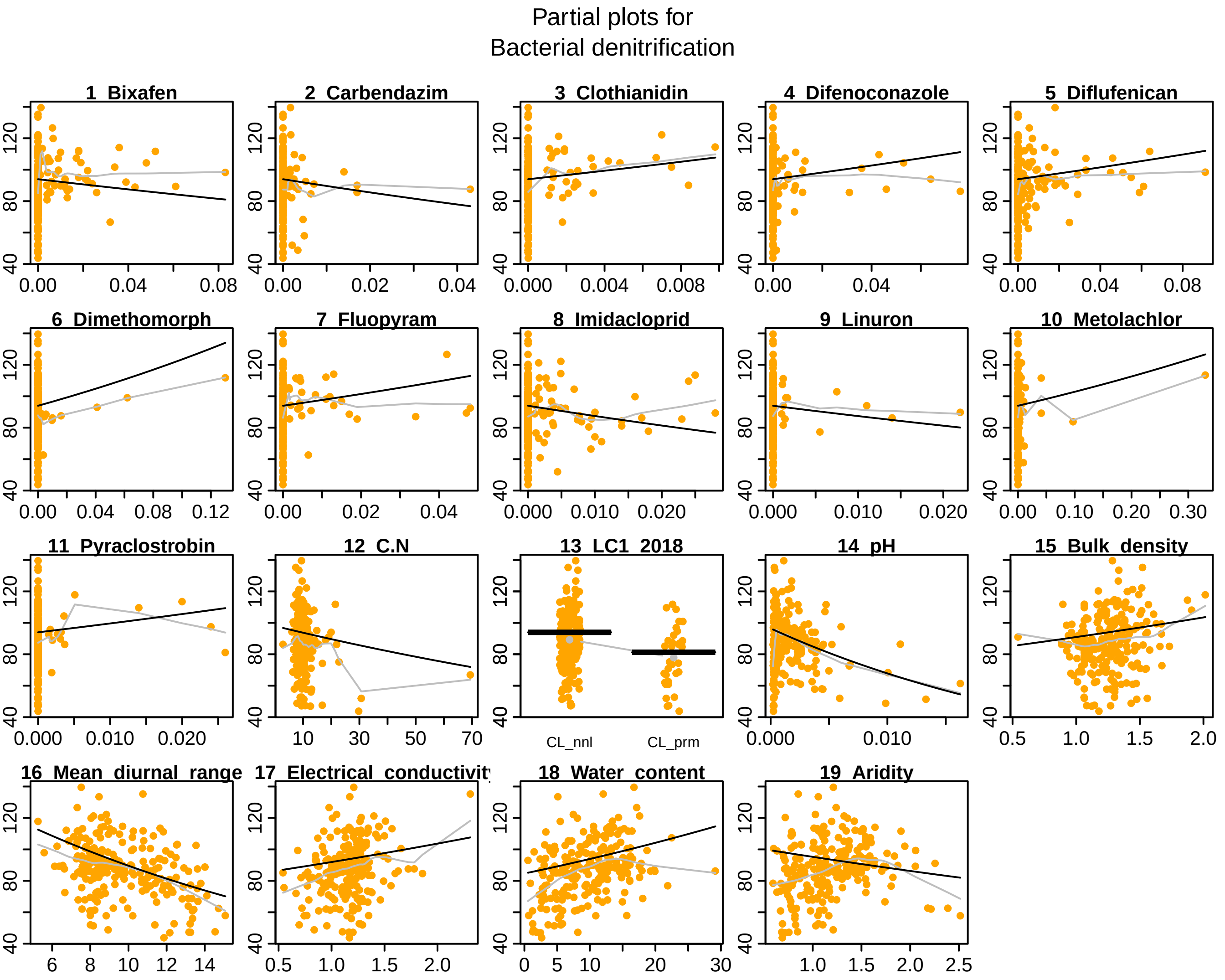
Partial plots for
Bacterial denitrification
1 Bixafen
2 Carbendazim
3 Clothianidin
4 Difenoconazole
5 Diflufenican
6 Dimethomorph
7 Fluopyram
8 Imidacloprid
9 Linuron
10 Metolachlor
11 Pyraclostrobin
12 C.N
13 LC1 2018
14 pH
15 Bulk density
16 Mean diurnal range
17 Electrical conductivity
18 Water content
19 Aridity
CL_nnl
CL_prm

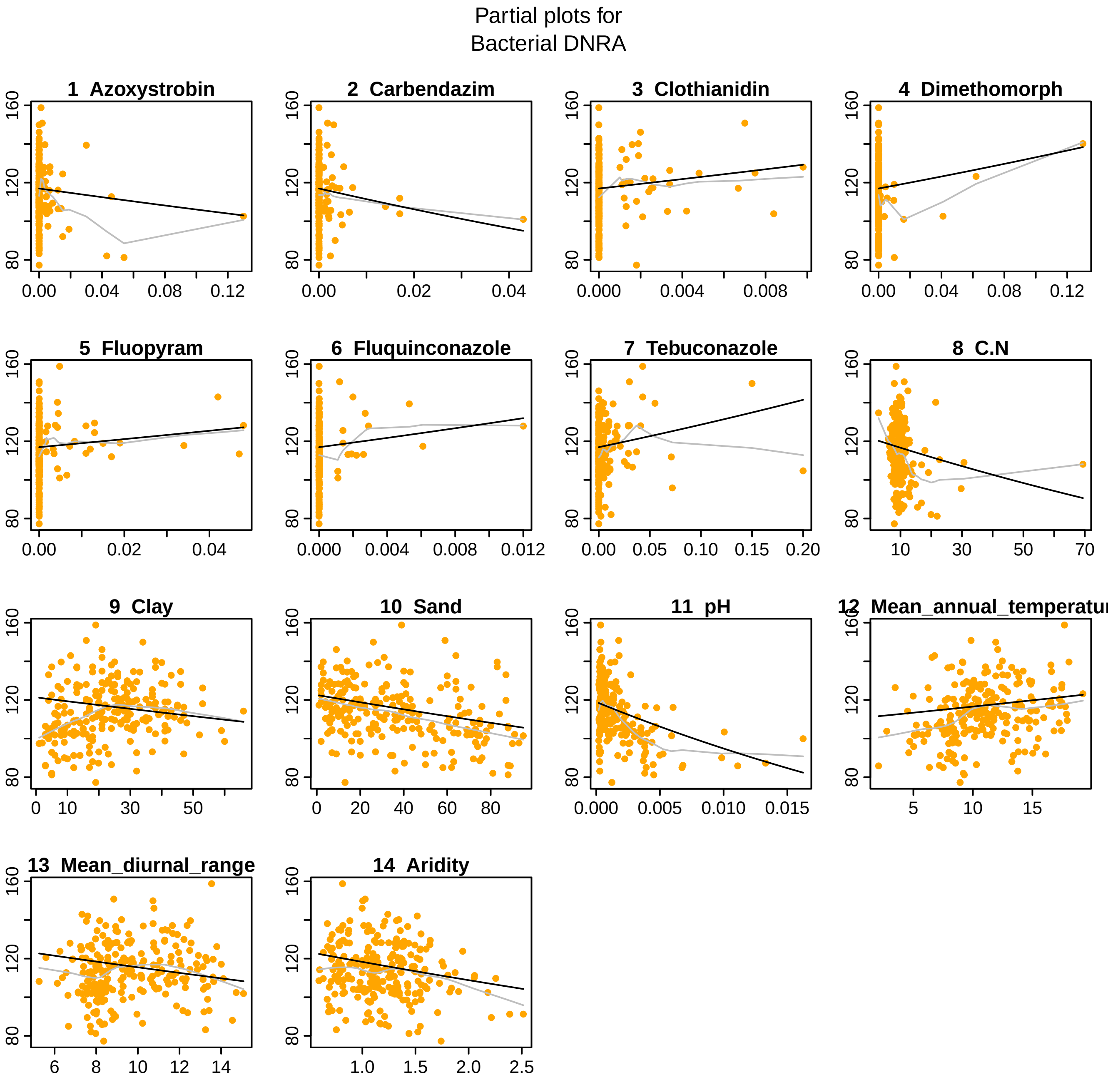
Partial plots for
Bacterial DNRA
1 Azoxystrobin
2 Carbendazim
3 Clothianidin
4 Dimethomorph
5 Fluopyram
6 Fluquinconazole
7 Tebuconazole
8 C.N
9 Clay
10 Sand
11 pH
12 Mean_annual_temperatur
13 Mean_diurnal_range
14 Aridity
80
120
160
0.00
0.04
0.08
0.12
0.02
0.000
0.004
0.008
0.012
0.05
0.10
0.15
0.20
10
30
50
70
0
20
40
60
80
0.005
0.010
0.015
5
15
6
8
12
14
1.0
1.5
2.0
2.5

Partial plots for
Fungal nitrate assimilation

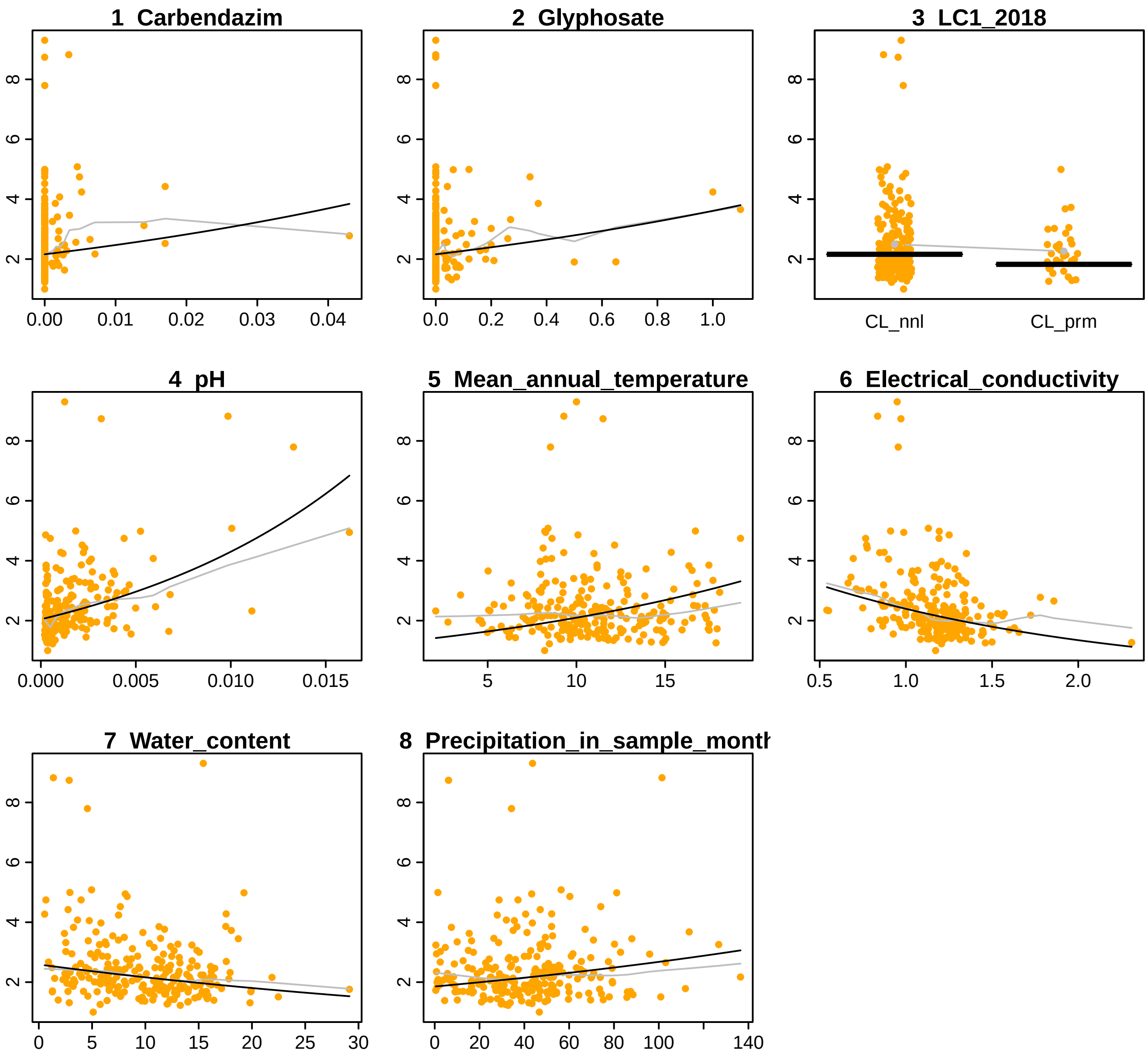

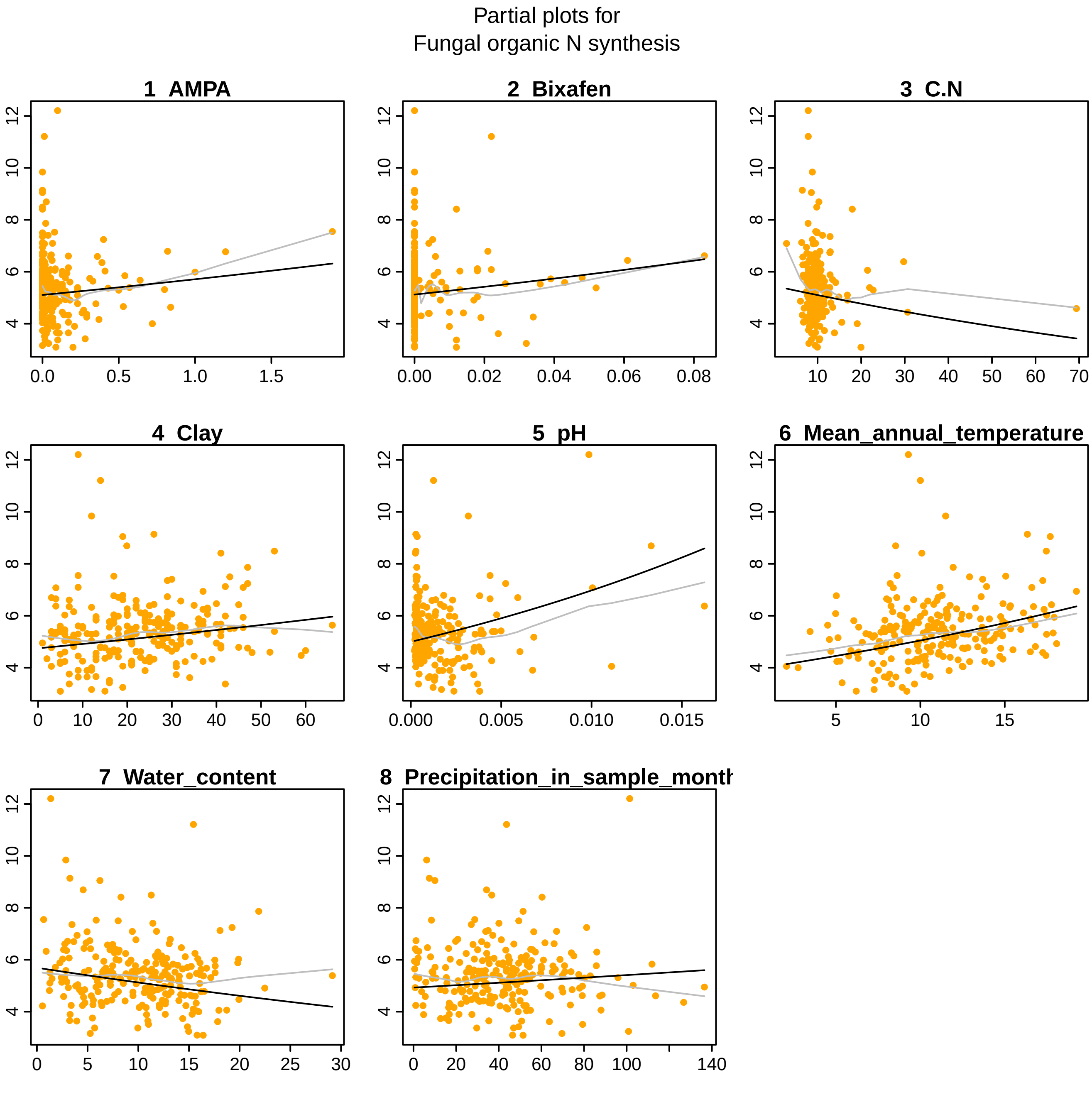
Partial plots for
Fungal organic N synthesis
1 AMPA
2 Bixafen
3 C.N
4 Clay
5 pH
6 Mean_annual_temperature
7 Water_content
8 Precipitation_in_sample_montl

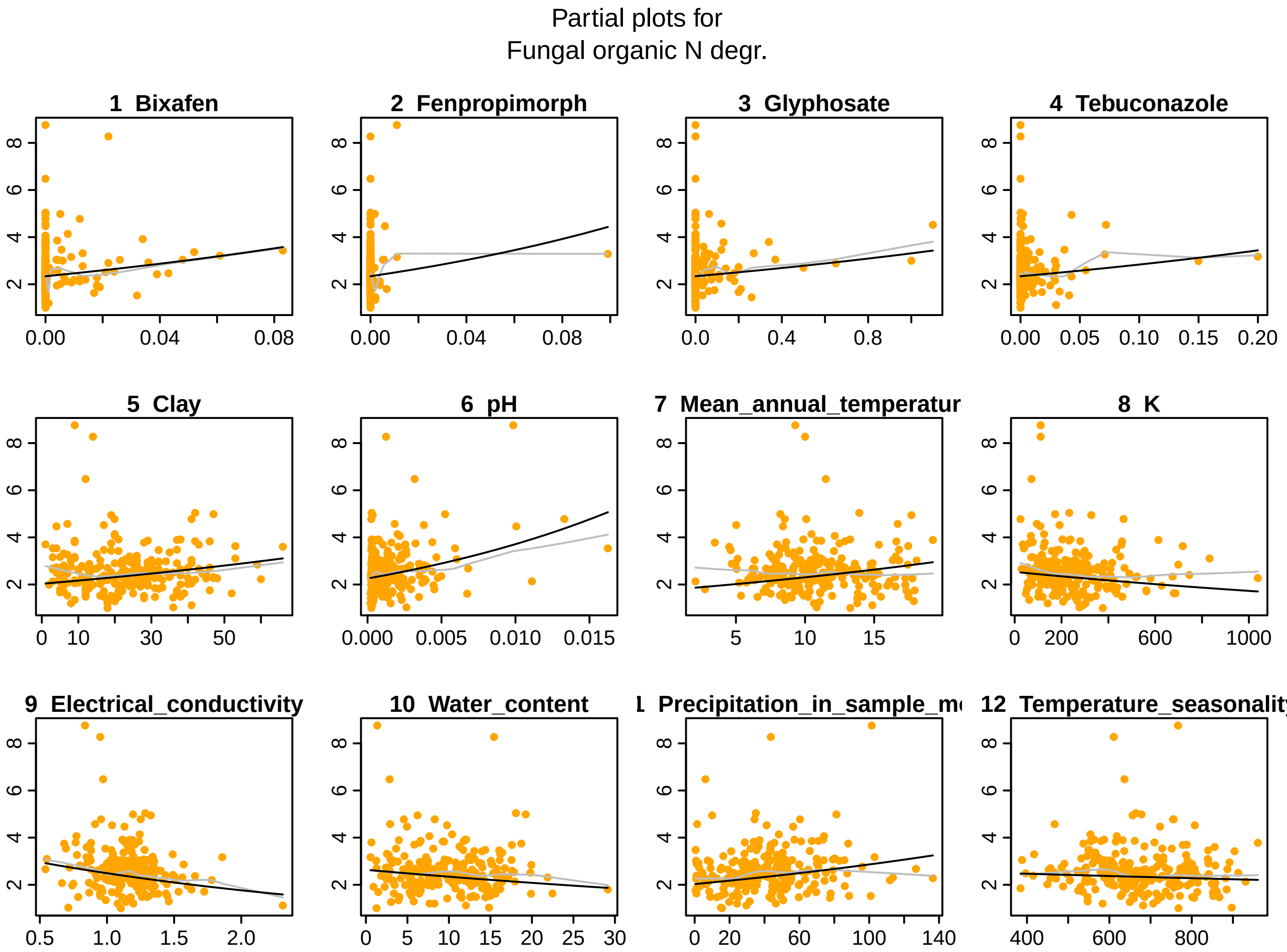
Partial plots for
Fungal organic N degr.
1 Bixafen
2 Fenpropimorph
3 Glyphosate
4 Tebuconazole
5 Clay
6 pH
7 Mean_annual_temperatur
8 K
9 Electrical_conductivity
10 Water_content
Precipitation_in_sample_m
12 Temperature_seasonalit

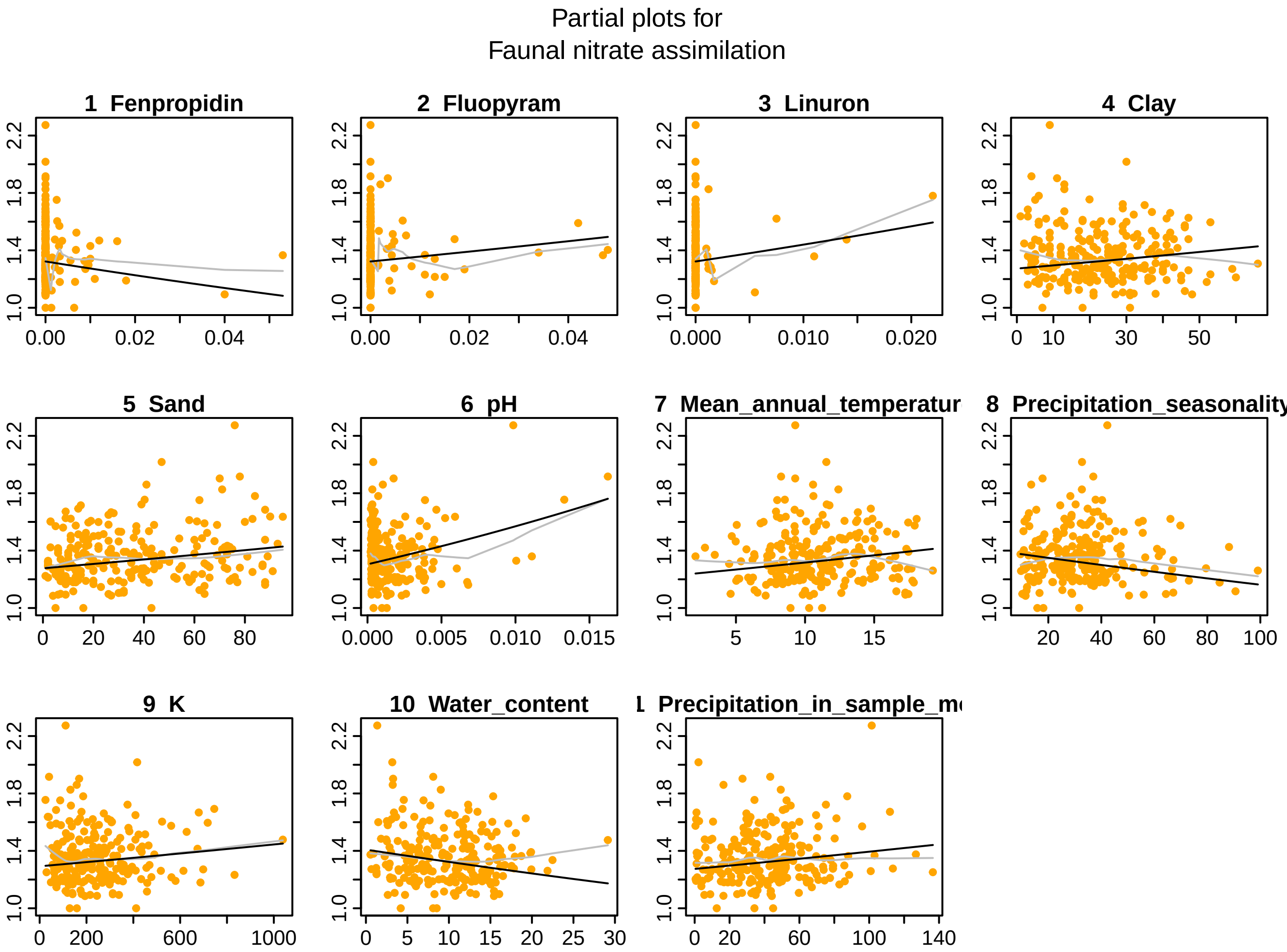
Partial plots for
Faunal nitrate assimilation
1 Fenpropidin
2 Fluopyram
3 Linuron
4 Clay
5 Sand
6 pH
7 Mean_annual_temperatur
8 Precipitation_seasonality
9 K
10 Water_content
Precipitation_in_sample_m
1.0
1.4
1.8
2.2
0.00
0.02
0.04
0.000
0.010
0.020
0
10
30
50
20
40
60
80
0.005
0.015
5
15
100
200
600
1000
25
140

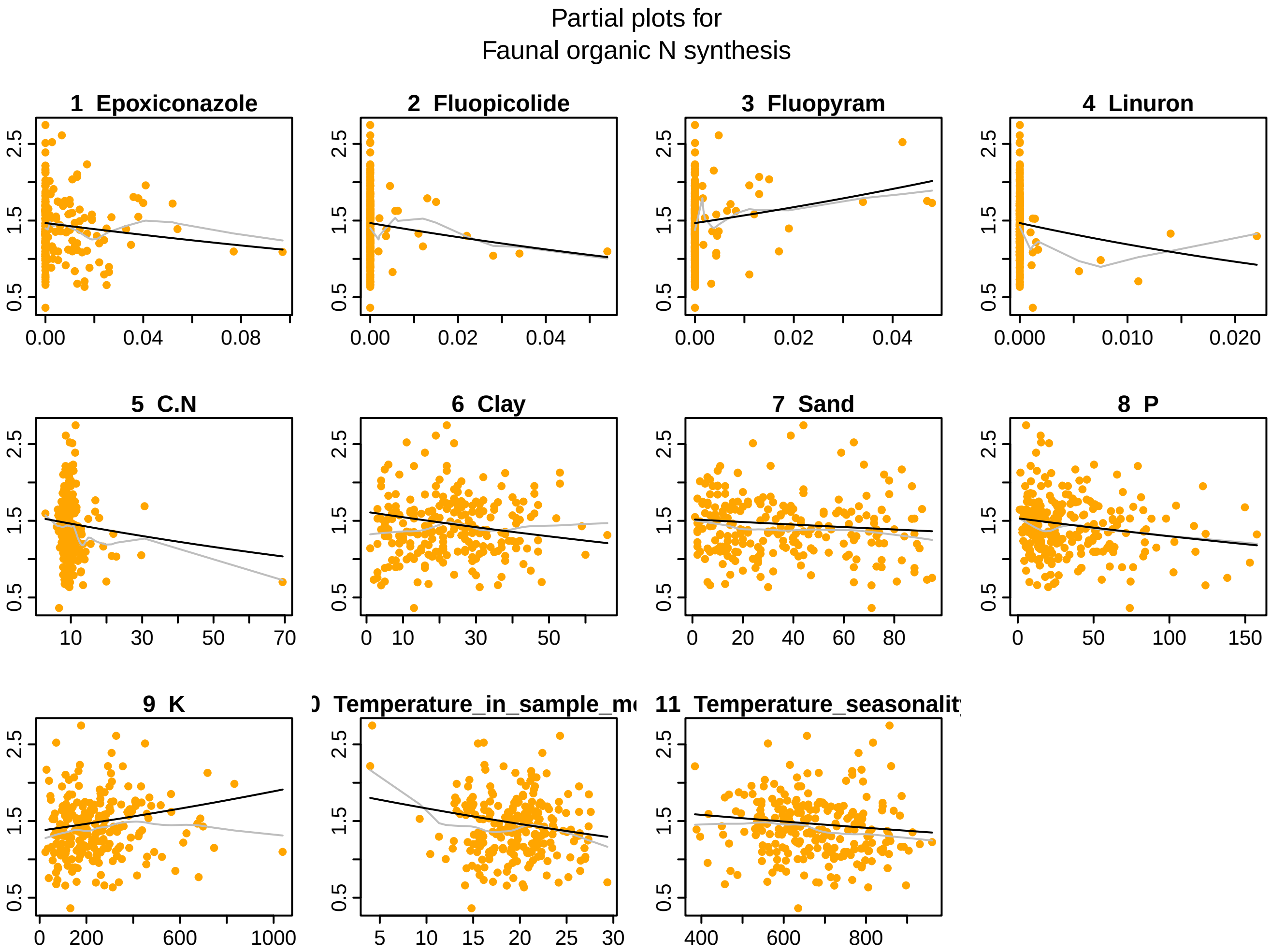
Partial plots for
Faunal organic N synthesis
1 Epoxiconazole
2 Fluopicolide
3 Fluopyram
4 Linuron
5 C.N
6 Clay
7 Sand
8 P
9 K
0 Temperature_in_sample_m
11 Temperature_seasonality

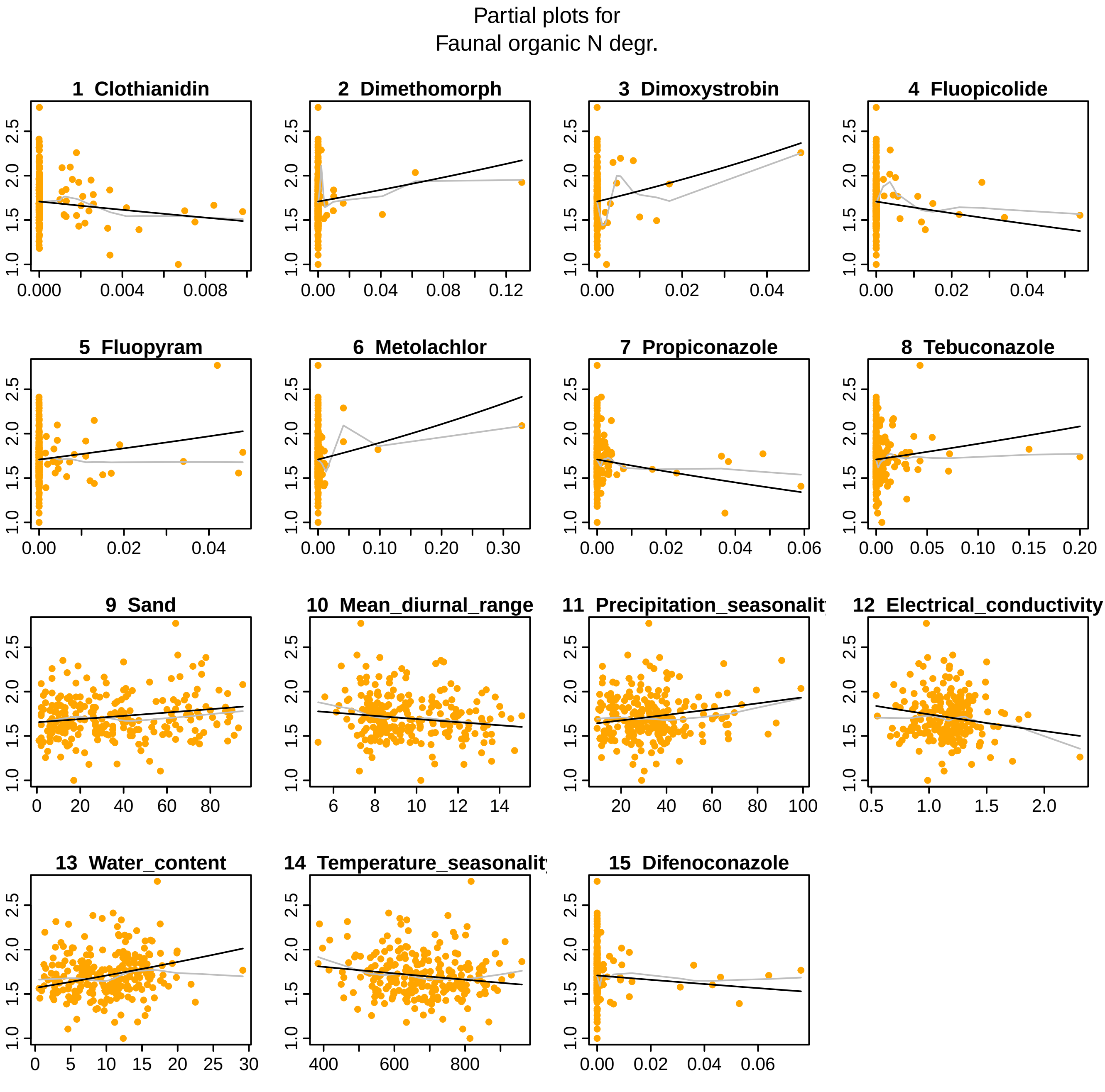
Partial plots for
Faunal organic N degr.
1 Clothianidin
2 Dimethomorph
3 Dimoxystrobin
4 Fluopicolide
5 Fluopyram
6 Metolachlor
7 Propiconazole
8 Tebuconazole
9 Sand
10 Mean_diurnal_range
11 Precipitation_seasonalit
12 Electrical_conductivity
13 Water_content
14 Temperature_seasonalit
15 Difenoconazole

Partial plots for
Archaeal mineral P import

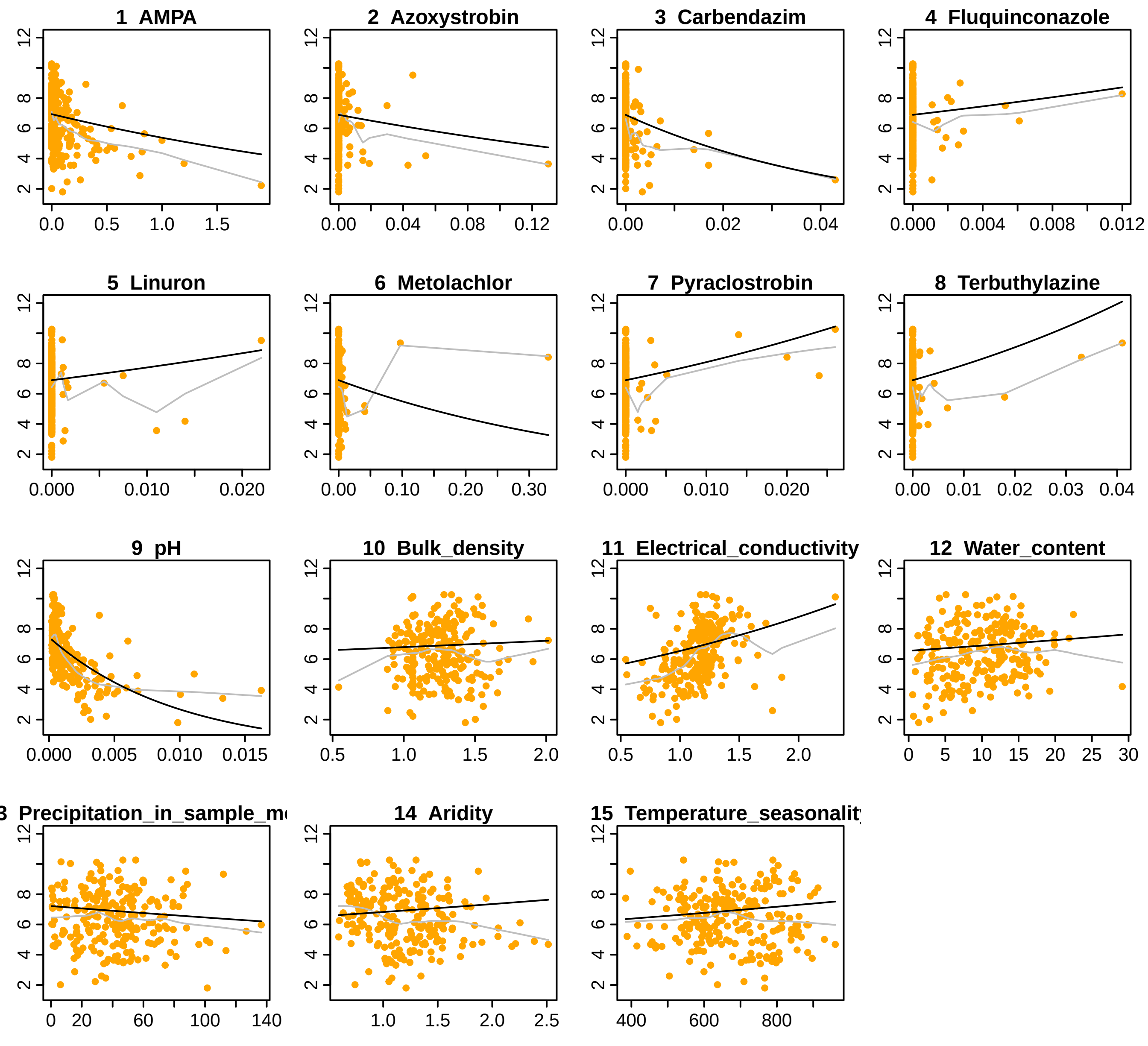

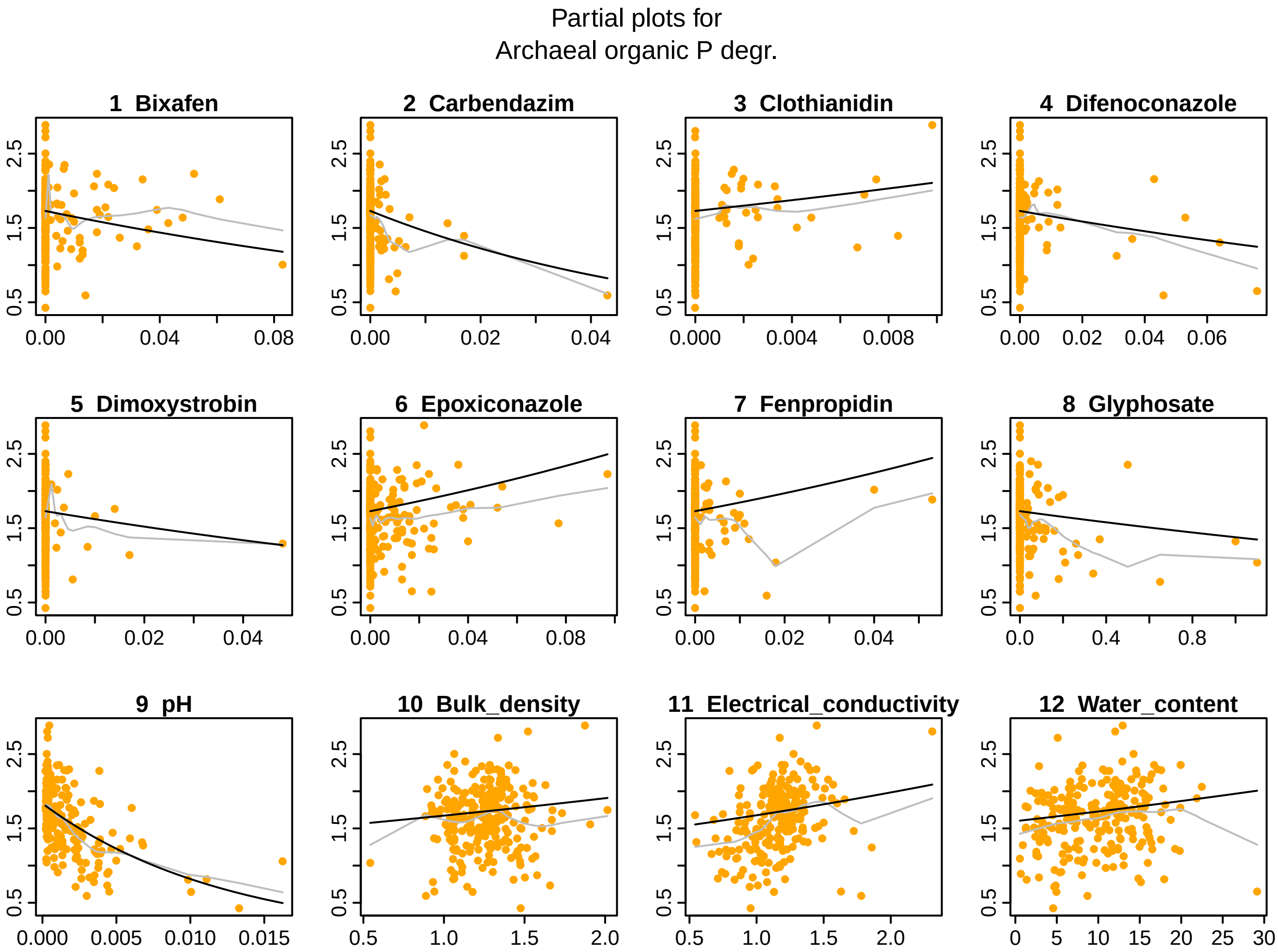
Partial plots for
Archaeal organic P degr.
1 Bixafen
2 Carbendazim
3 Clothianidin
4 Difenoconazole
5 Dimoxystrobin
6 Epoxiconazole
7 Fenpropidin
8 Glyphosate
9 pH
10 Bulk_density
11 Electrical_conductivity
12 Water_content

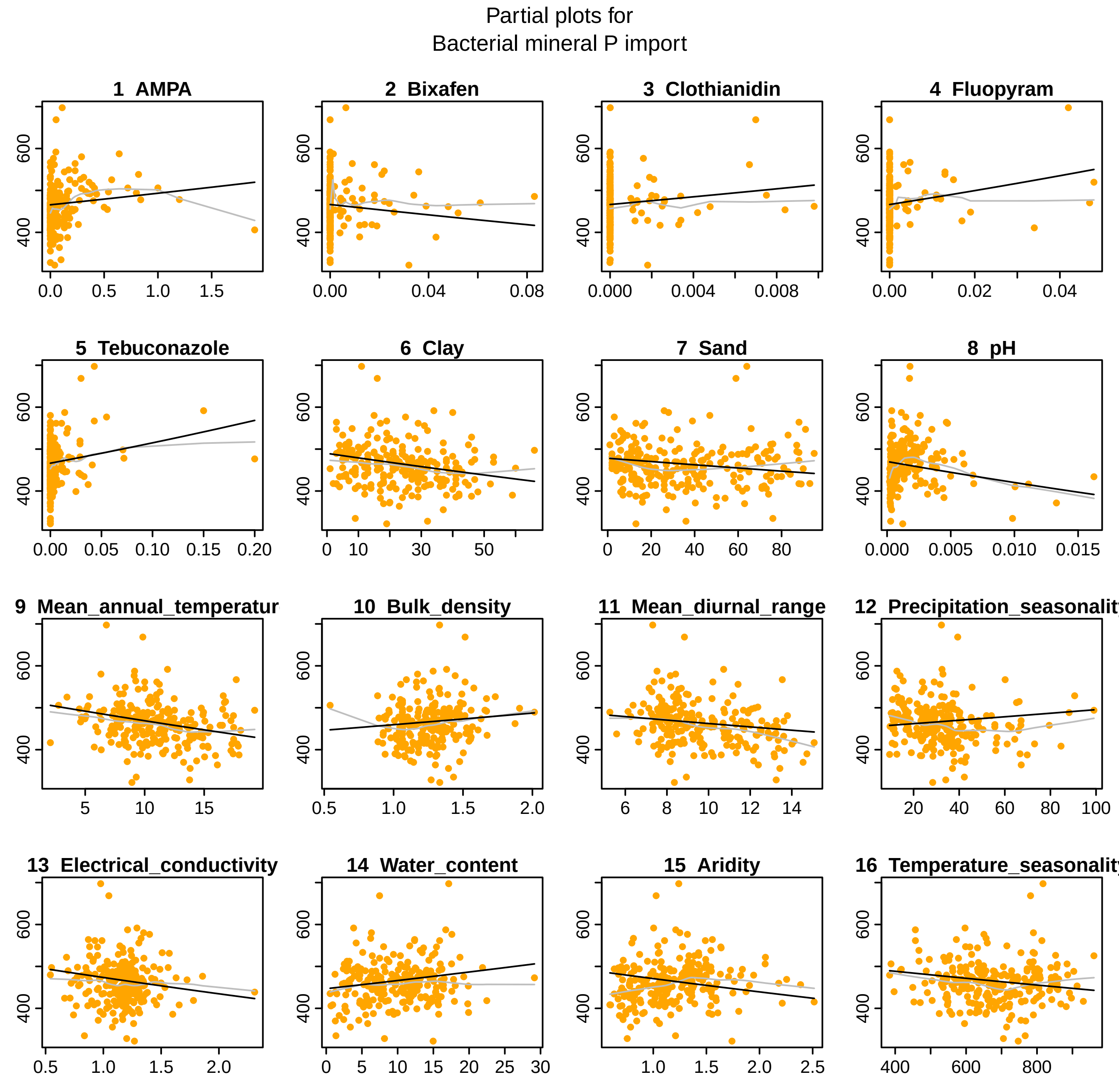
Partial plots for
Bacterial mineral P import
1 AMPA
2 Bixafen
3 Clothianidin
4 Fluopyram
5 Tebuconazole
6 Clay
7 Sand
8 pH
9 Mean_annual_temperatur
10 Bulk_density
11 Mean_diurnal_range
12 Precipitation_seasonality
13 Electrical_conductivity
14 Water_content
15 Aridity
16 Temperature_seasonality
400
600

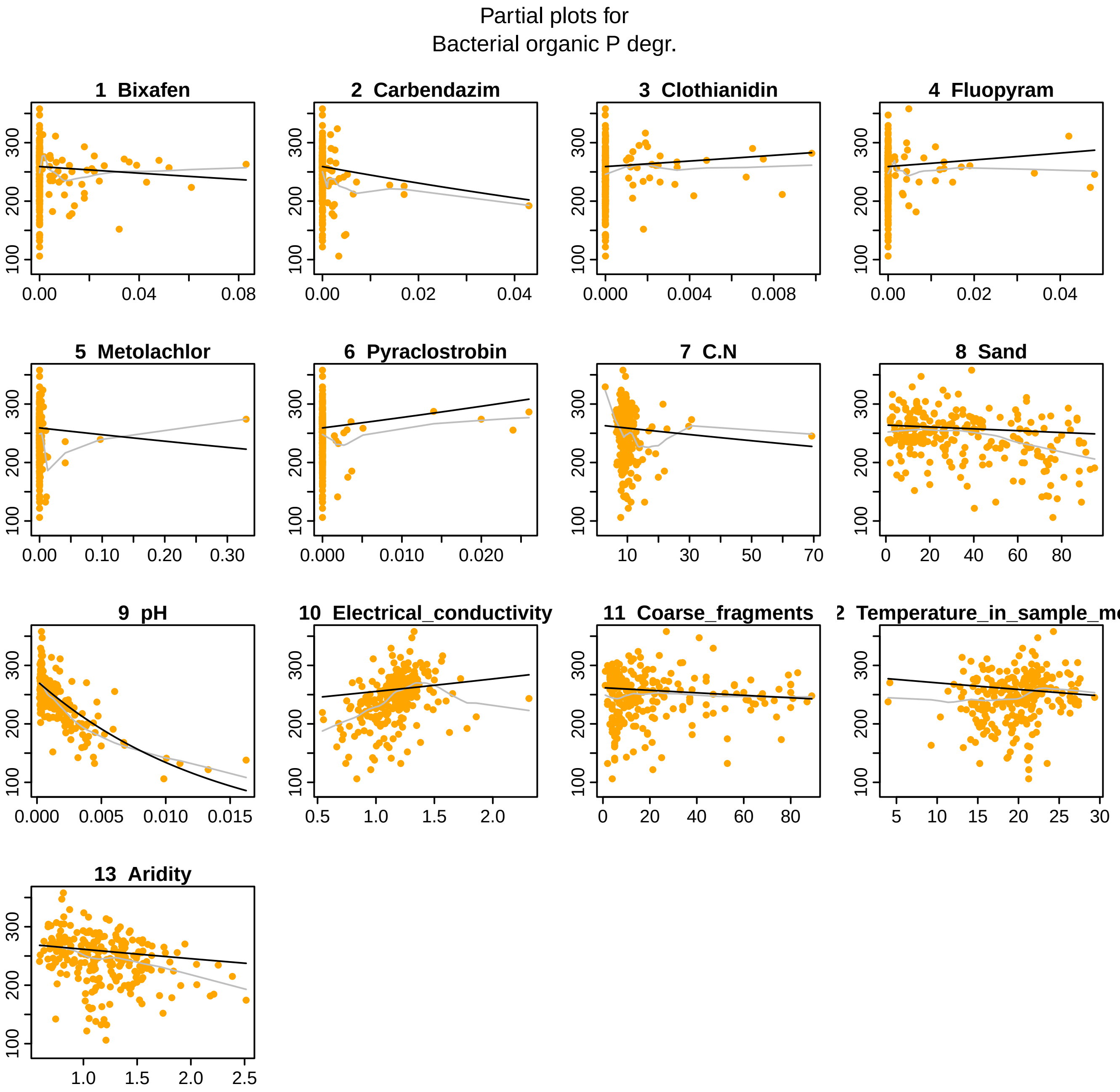

Partial plots for
Bacterial organic P degr.
1 Bixafen
2 Carbendazim
3 Clothianidin
4 Fluopyram
5 Metolachlor
6 Pyraclostrobin
7 C.N
8 Sand
9 pH
10 Electrical_conductivity
11 Coarse_fragments
2 Temperature_in_sample_m
13 Aridity

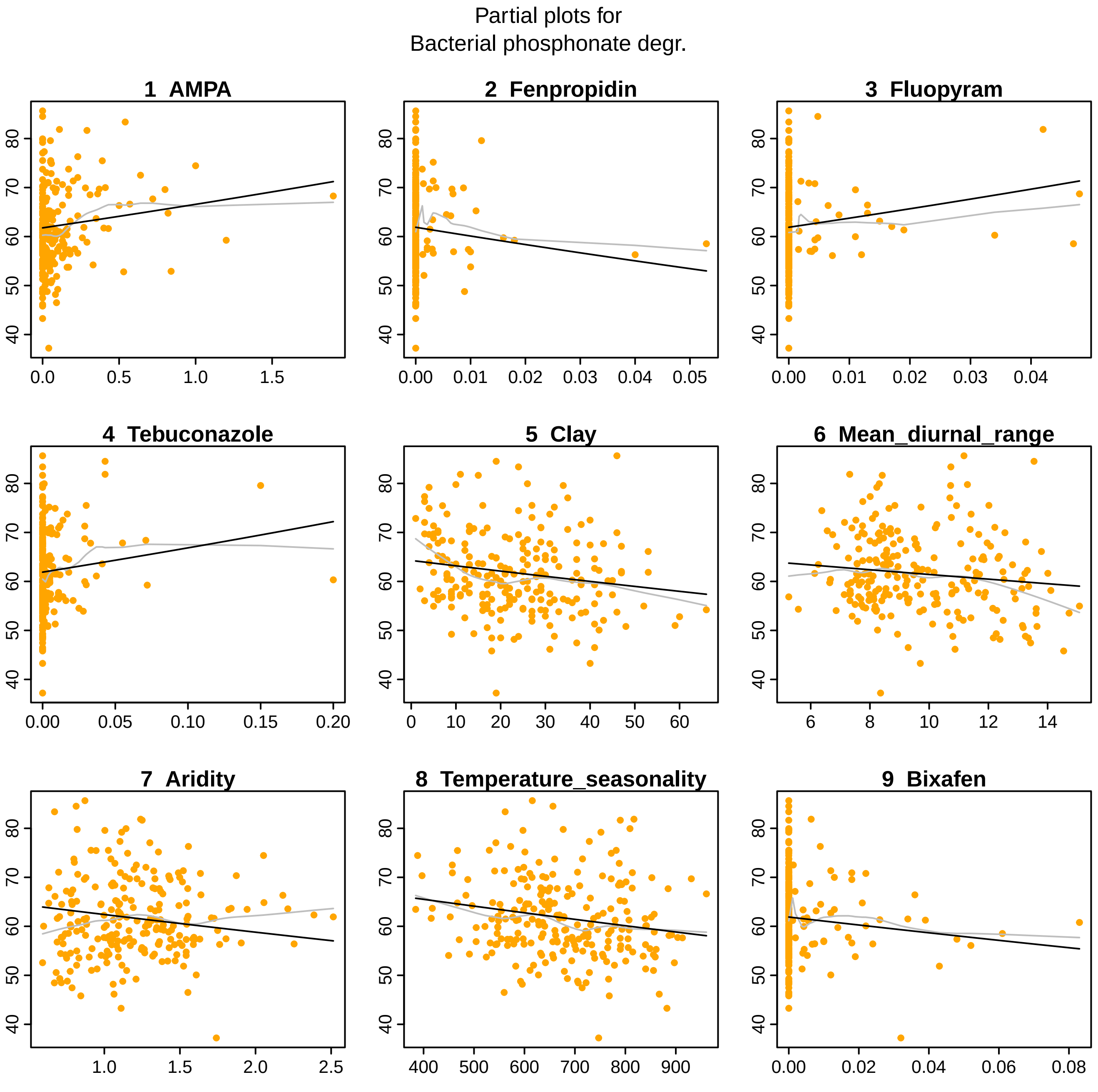

Partial plots for
Bacterial phosphonate degr.
1 AMPA
2 Fenpropidin
3 Fluopyram
4 Tebuconazole
5 Clay
6 Mean_diurnal_range
7 Aridity
8 Temperature_seasonality
9 Bixafen

Partial plots for
Fungal organic P degr.

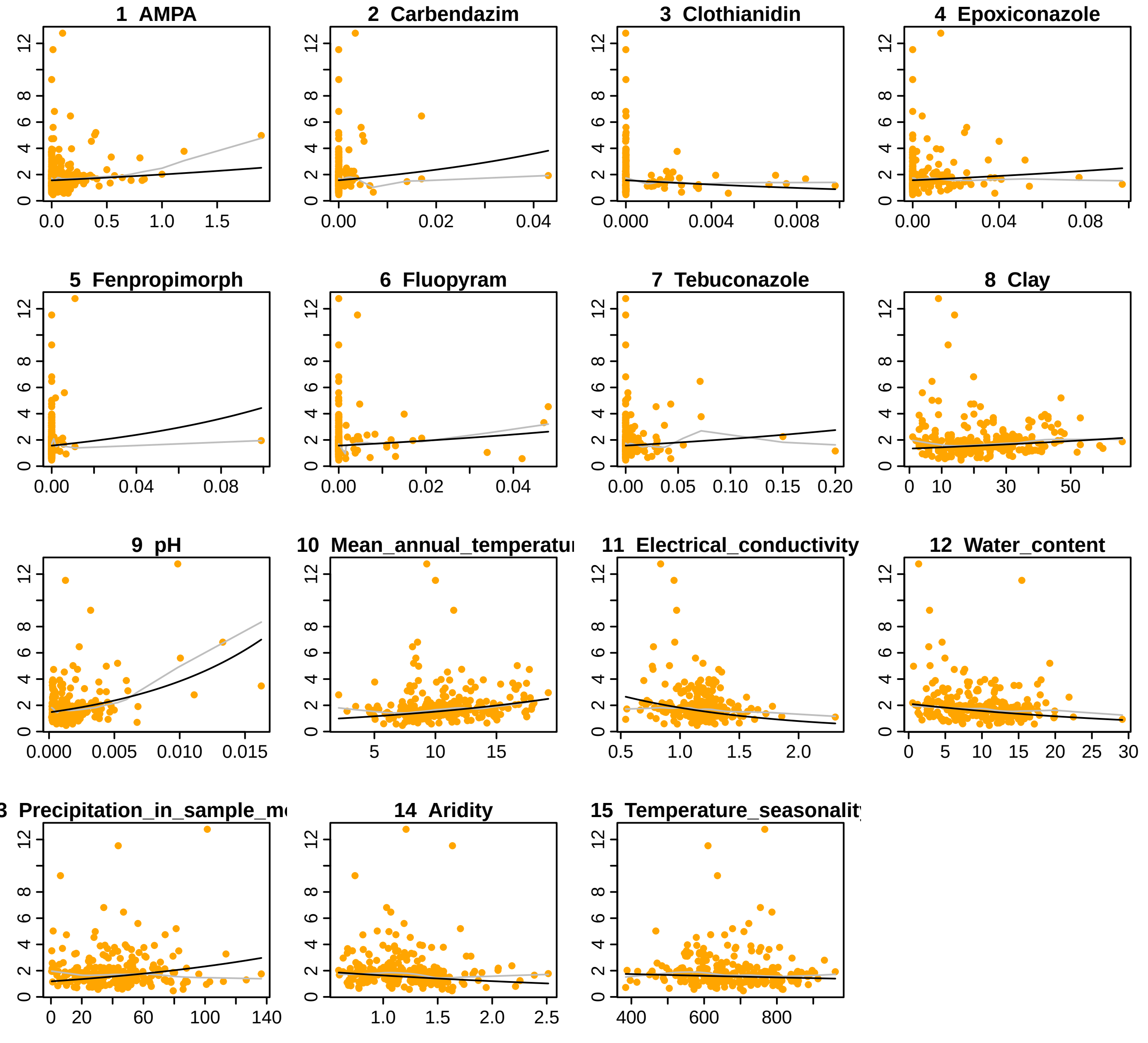

Supplement: Supplementary file 7 — Partial plots of the GLMs for croplands only. See main Supplementary Information file for further description. [file 41586_2025_9991_MOESM7_ESM.pdf]
