# Supplementary material for: Pesticide residues alter taxonomic and functional biodiversity in soils
Source: Nature. 2026 Jan 28;650(8101):367–73. doi: 10.1038/s41586-025-09991-z (PMC12965876; doi:10.1038/s41586-025-09991-z)
Supplement: Supplementary file 8 — Partial plots of the GLM for all ecosystem types. See main Supplementary Information file for further description. [file 41586_2025_9991_MOESM8_ESM.pdf]

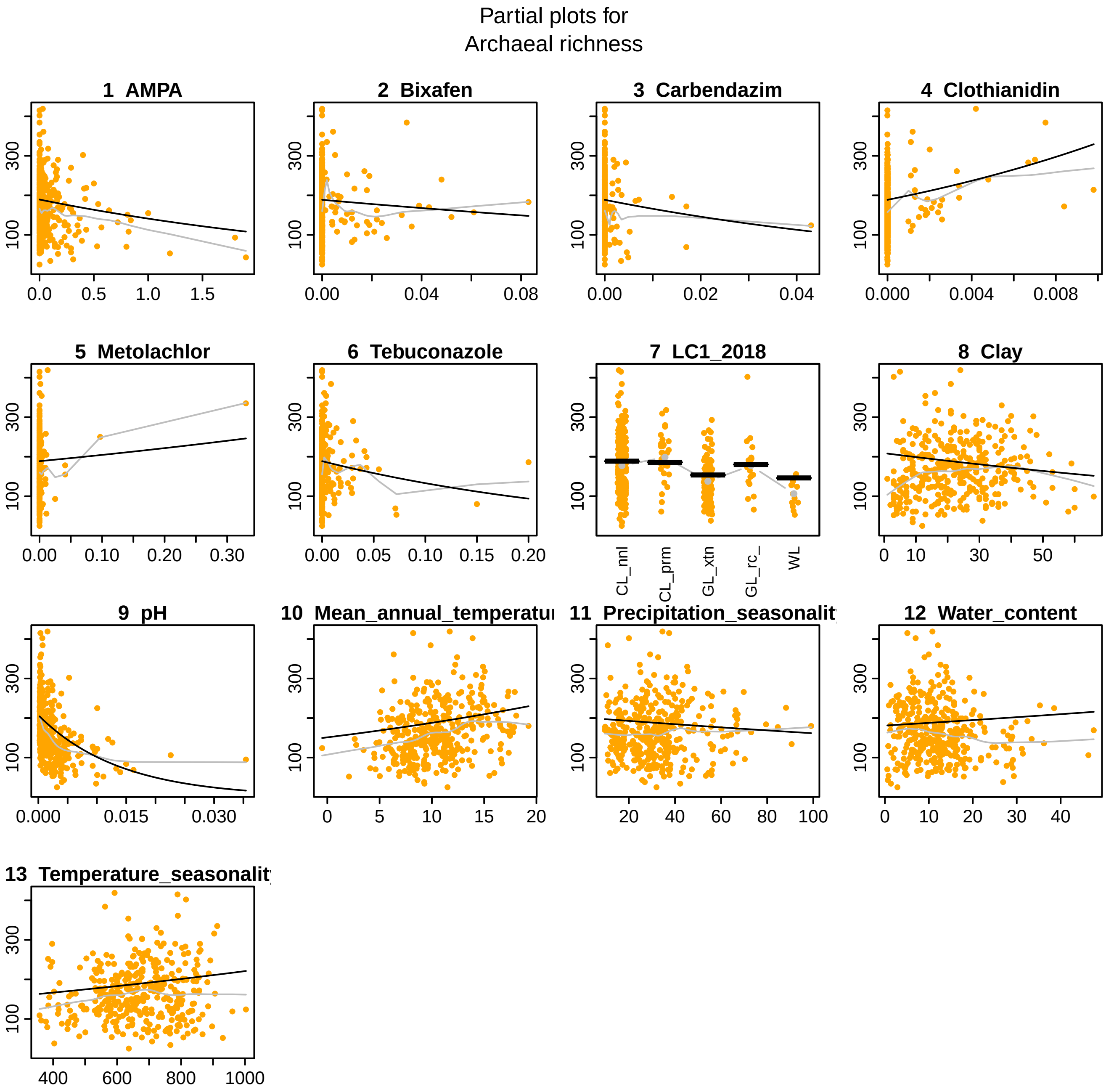
Partial plots for
Archaeal richness
1 AMPA
2 Bixafen
3 Carbendazim
4 Clothianidin
5 Metolachlor
6 Tebuconazole
7 LC1_2018
8 Clay
9 pH
10 Mean_annual_temperatu
11 Precipitation_seasonalit
12 Water_content
13 Temperature_seasonalit
100
300
CL_nnl
CL_prm
GL_xtn
GL_rc_
WL

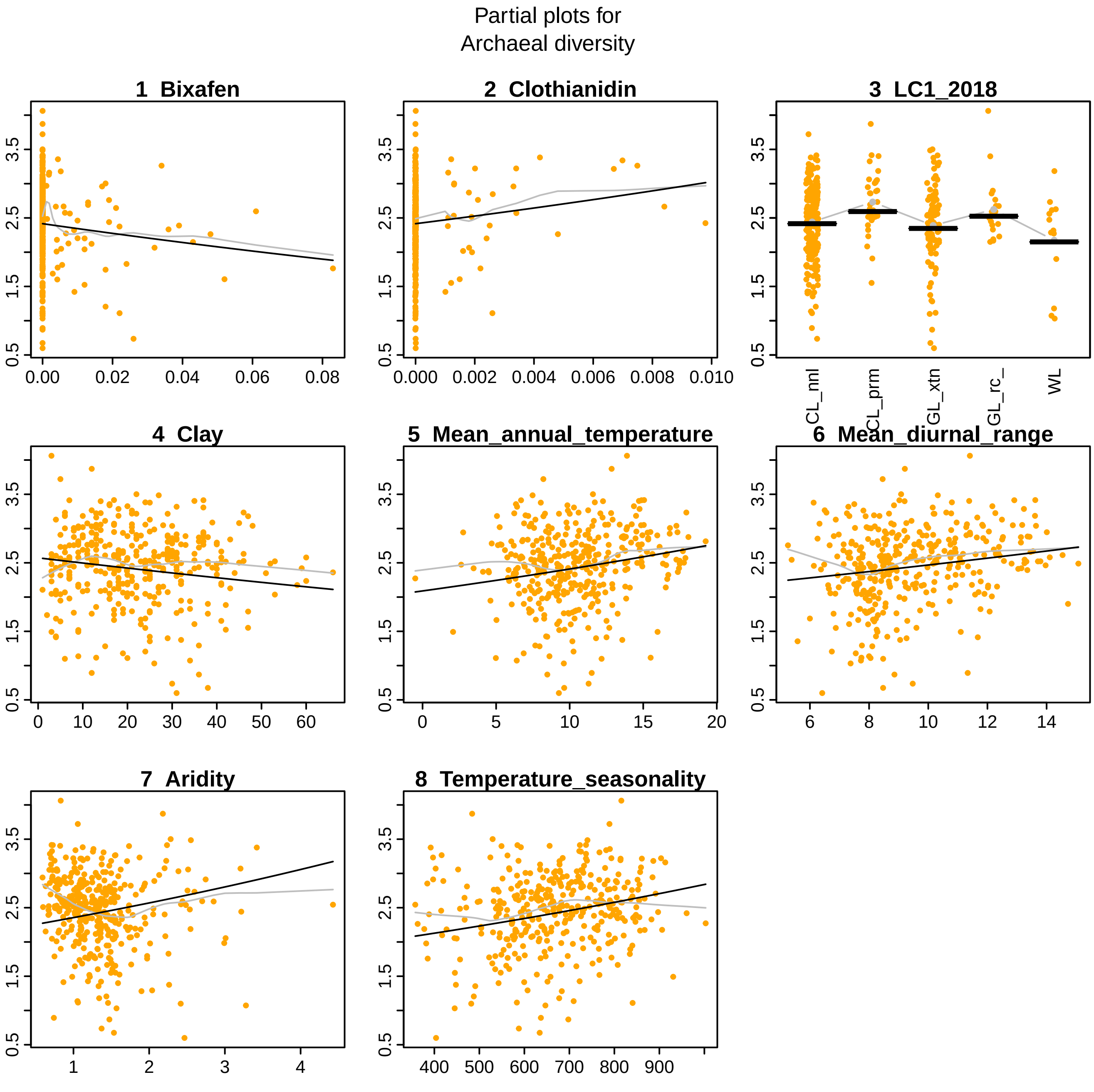
Partial plots for
Archaeal diversity
1 Bixafen
2 Clothianidin
3 LC1_2018
CL_nnl
CL_prm
GL_xtn
GL_rc_
WL
4 Clay
5 Mean_annual_temperature
6 Mean_diurnal_range
7 Aridity
8 Temperature_seasonality

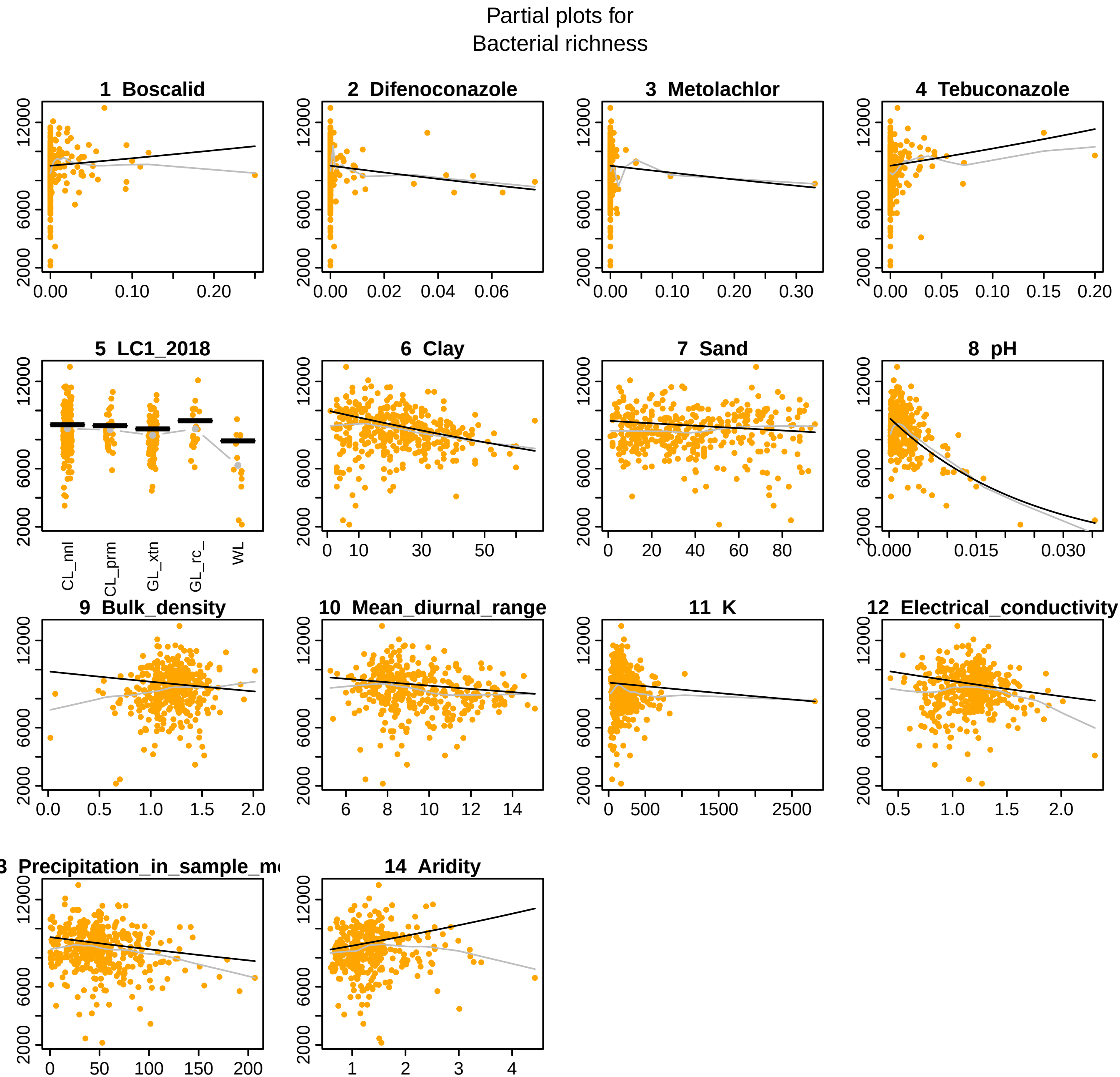

Partial plots for
Bacterial richness
1 Boscalid
2 Difenoconazole
3 Metolachlor
4 Tebuconazole
5 LC1_2018
CL_nnl
CL_prm
GL_xtn
GL_rc_
WL
6 Clay
7 Sand
8 pH
9 Bulk_density
10 Mean_diurnal_range
11 K
12 Electrical_conductivity
3 Precipitation_in_sample_mo
14 Aridity

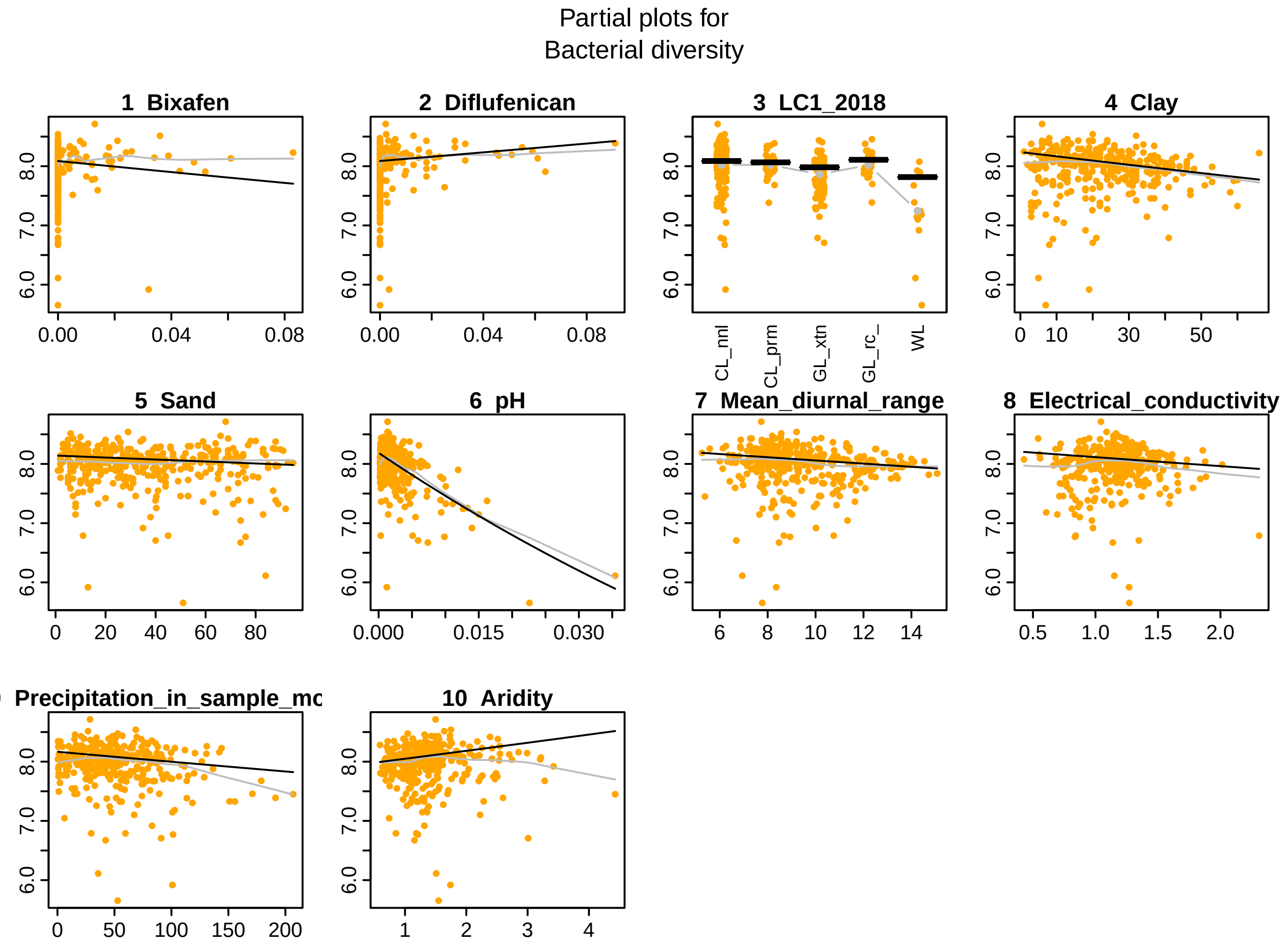

Partial plots for
Bacterial diversity
1 Bixafen
2 Diflufenican
3 LC1_2018
4 Clay
5 Sand
6 pH
7 Mean_diurnal_range
8 Electrical_conductivity
Precipitation_in_sample_mo
10 Aridity
CL_nnl
CL_prm
GL_xtn
GL_rc_
WL

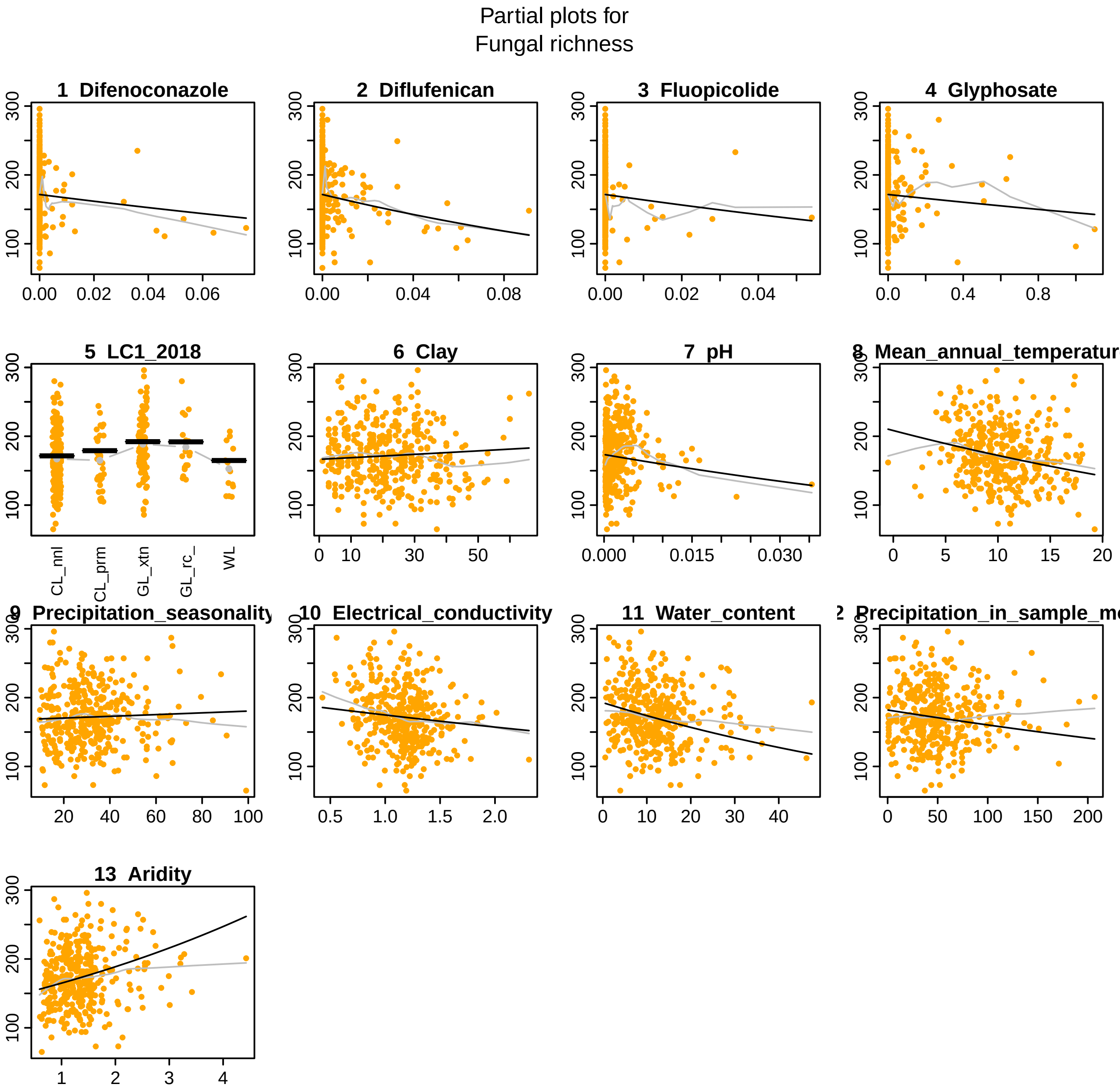

Partial plots for
Fungal richness
1 Difenoconazole
2 Diflufenican
3 Fluopicolide
4 Glyphosate
5 LC1_2018
6 Clay
7 pH
8 Mean_annual_temperatur
9 Precipitation_seasonality
10 Electrical_conductivity
11 Water_content
Precipitation_in_sample_mo
13 Aridity
CL_nnl
CL_prm
GL_xtn
GL_rc_
WL

Partial plots for
Fungal diversity

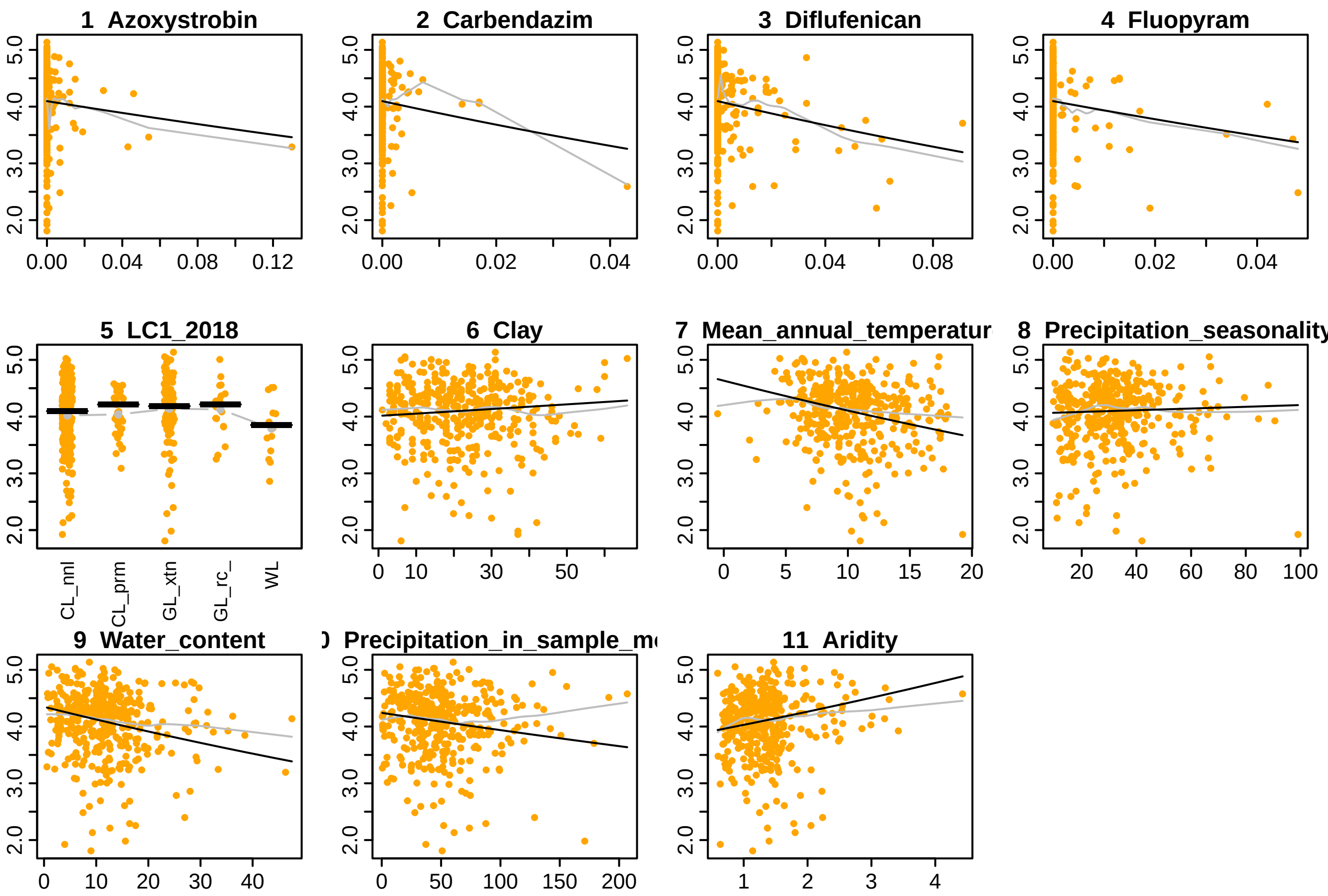

Partial plots for
Protist richness

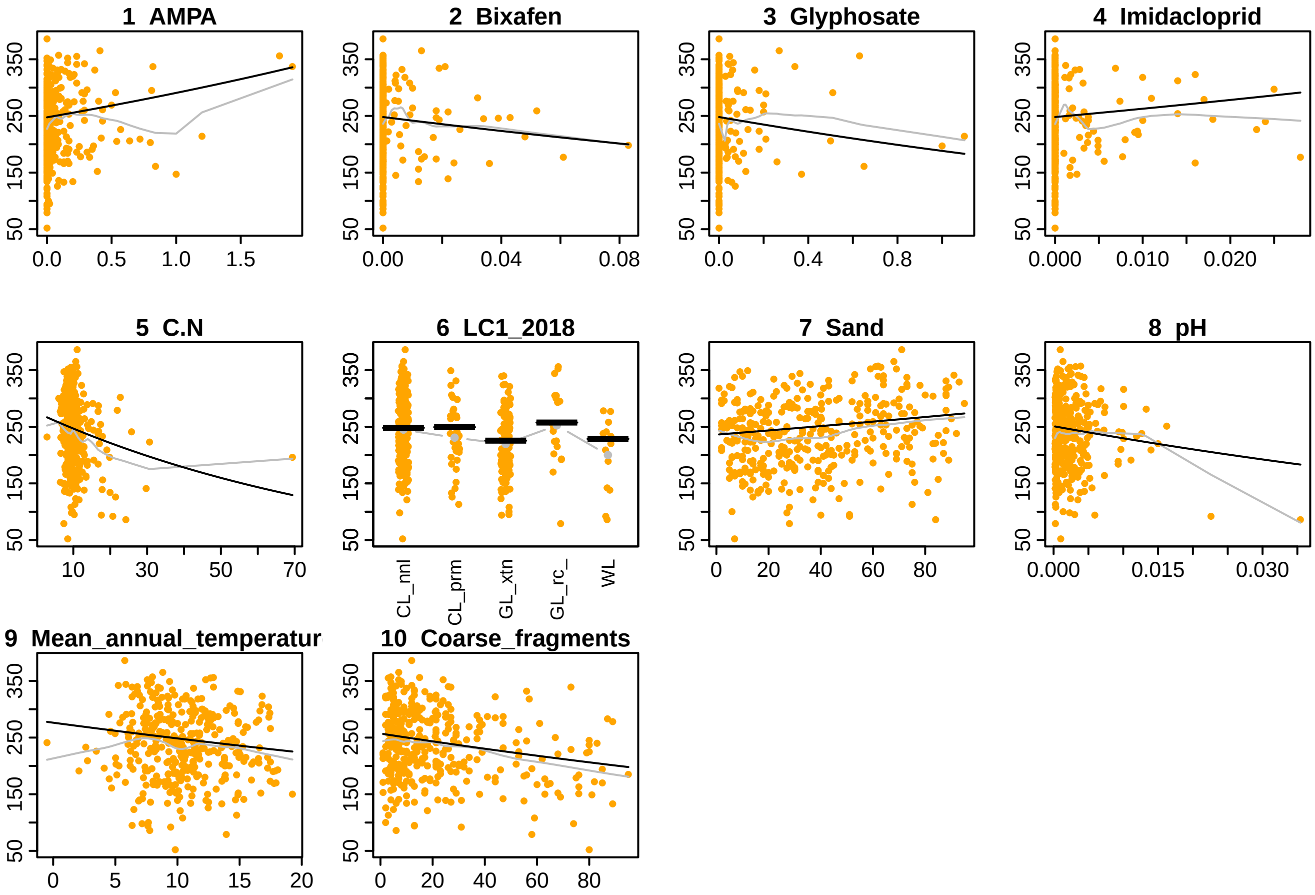

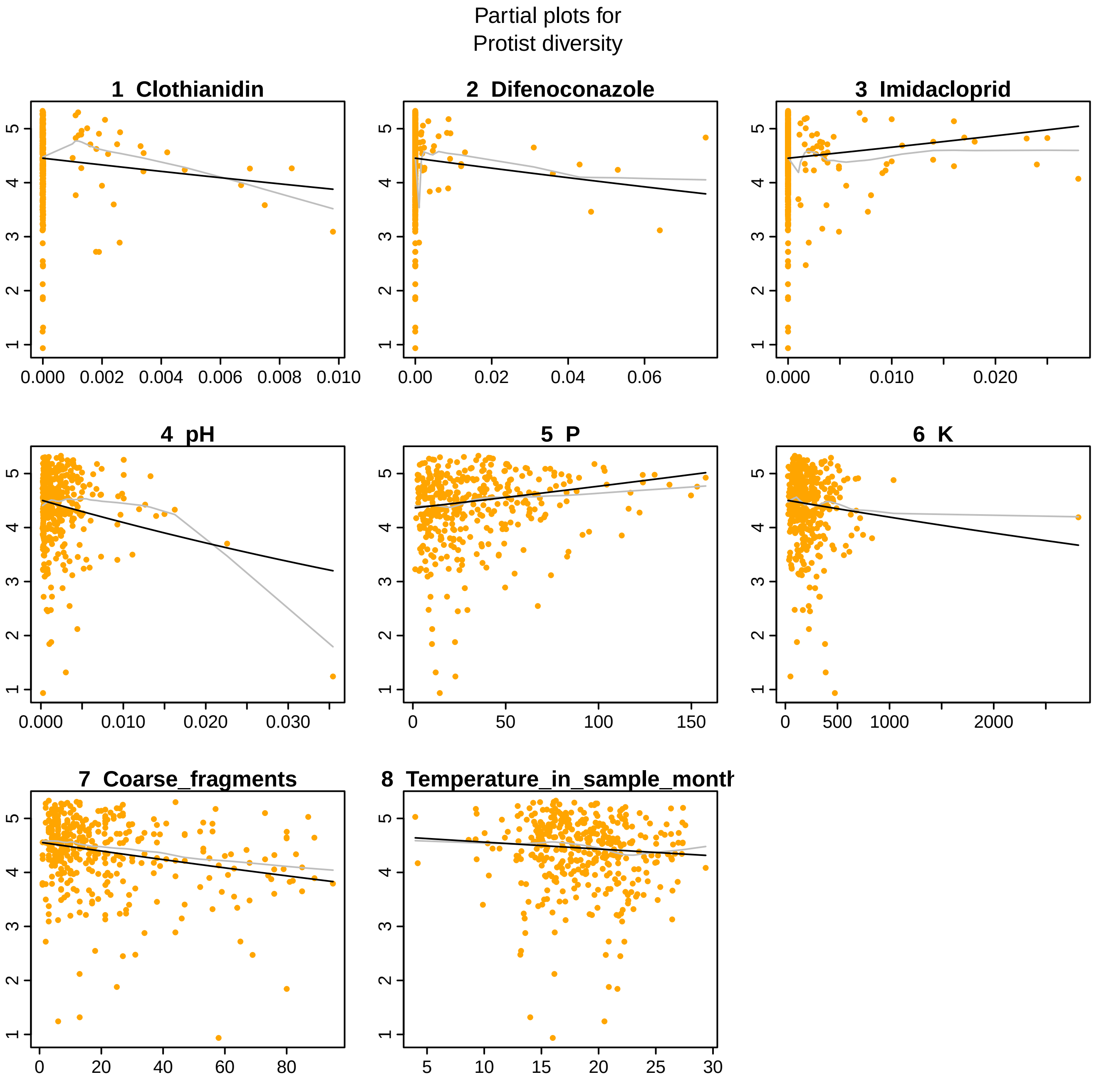
Partial plots for
Protist diversity
1 Clothianidin
2 Difenoconazole
3 Imidacloprid
4 pH
5 P
6 K
7 Coarse_fragments
8 Temperature_in_sample_montl

Partial plots for
Nematode richness

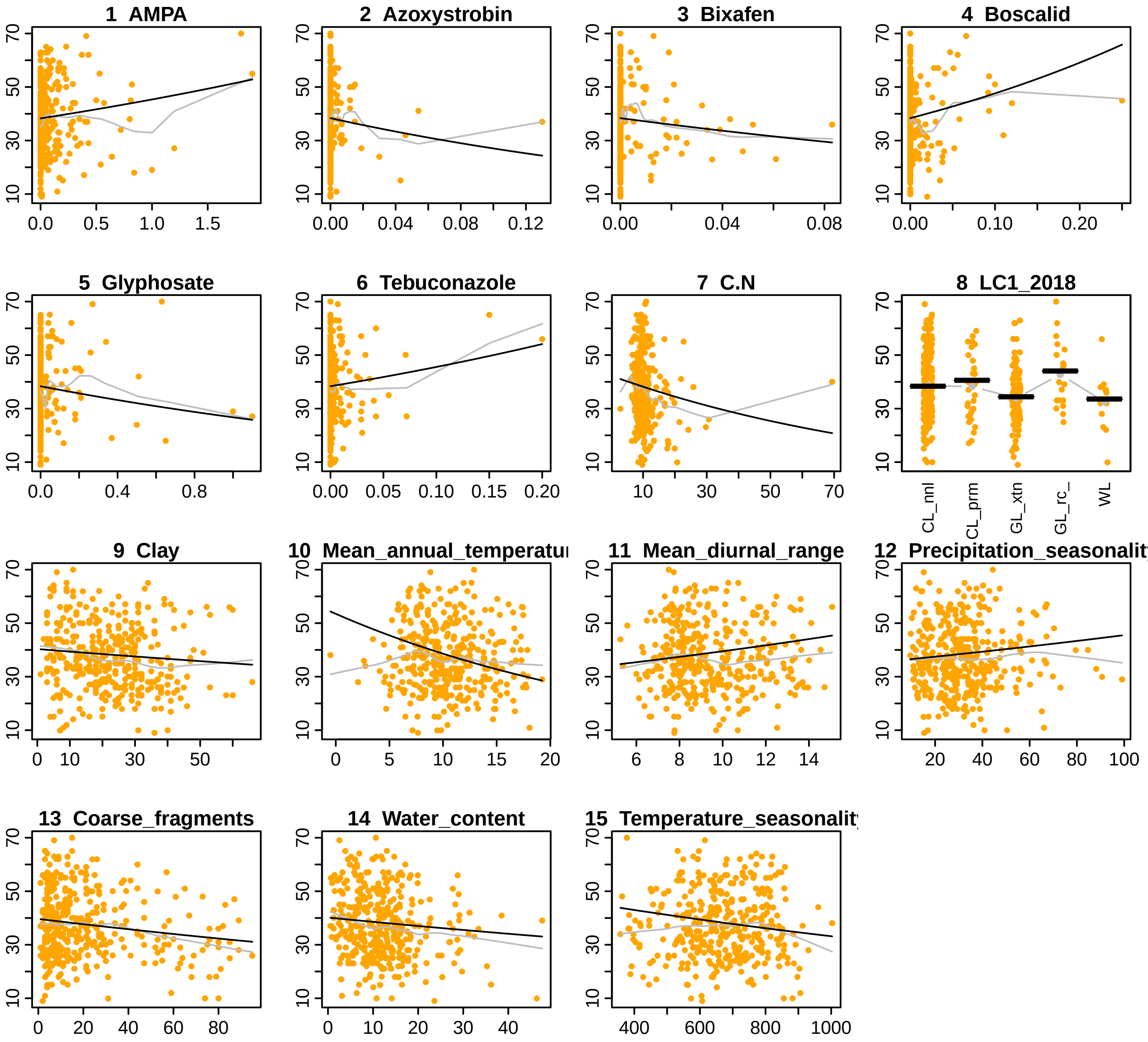

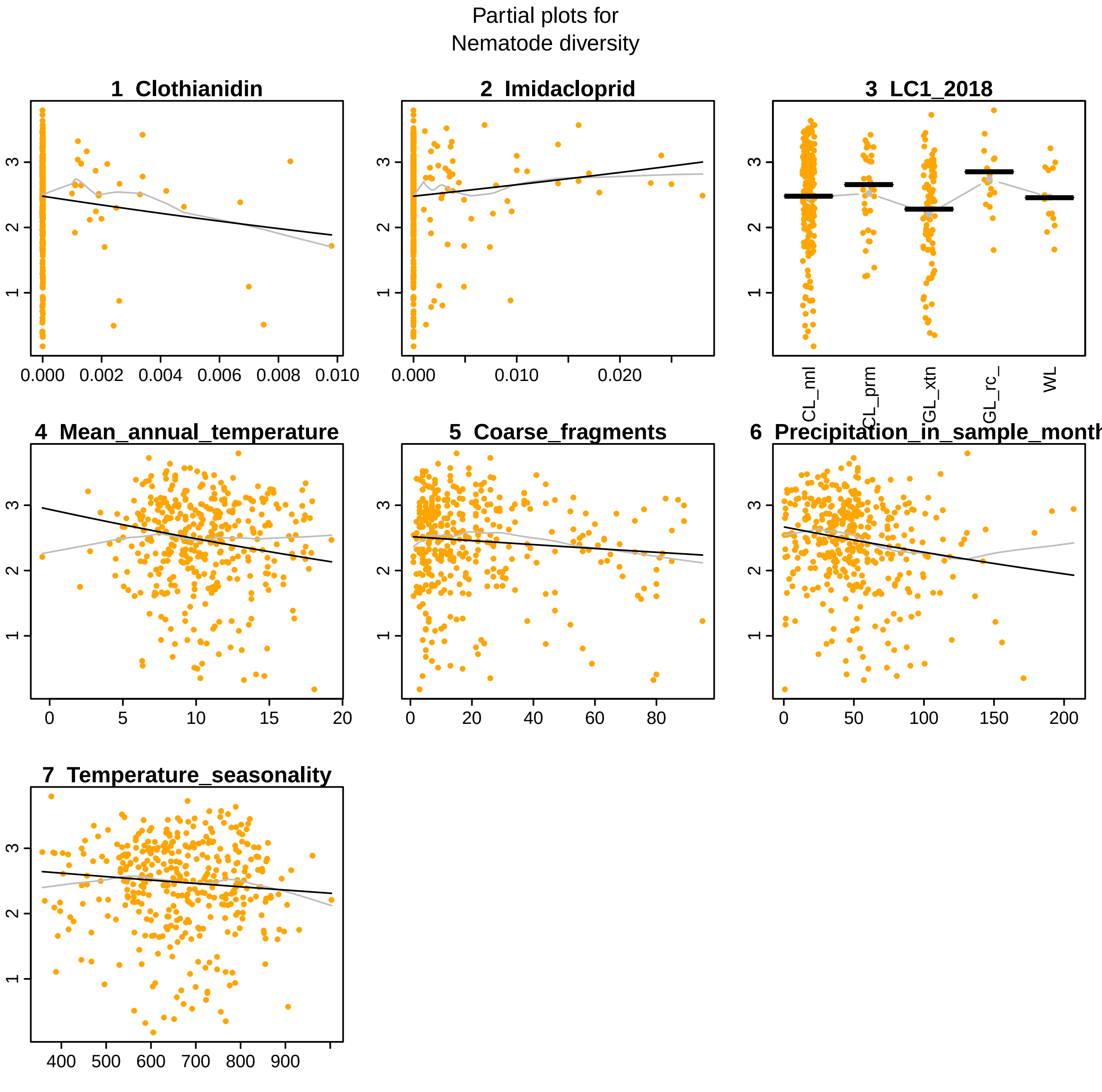
Partial plots for
Nematode diversity
1 Clothianidin
0.000
0.002
0.004
0.006
0.008
0.010
2 Imidacloprid
0.000
0.010
0.020
3 LC1_2018
CL_nnl
CL_prm
GL_xtn
GL_rc_
WL
4 Mean_annual_temperature
0
5
10
15
20
5 Coarse_fragments
0
20
40
60
80
6 Precipitation_in_sample_month
0
50
100
150
200
7 Temperature_seasonality
400
500
600
700
800
900
1
2
3

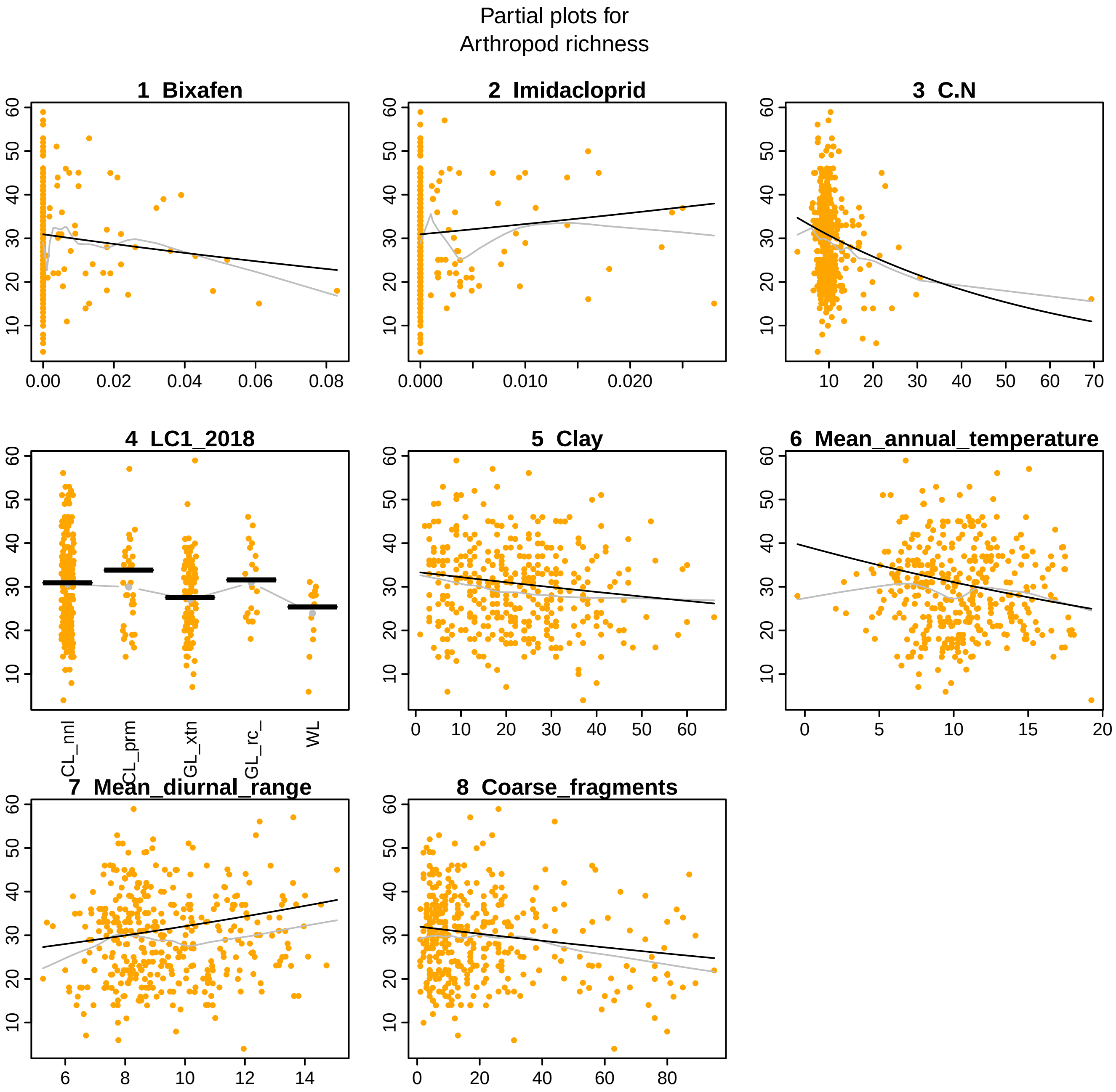
Partial plots for
Arthropod richness
1 Bixafen
2 Imidacloprid
3 C.N
4 LC1_2018
5 Clay
6 Mean_annual_temperature
7 Mean_diurnal_range
8 Coarse_fragments
CL_nnl
CL_prm
GL_xtn
GL_rc_
WL

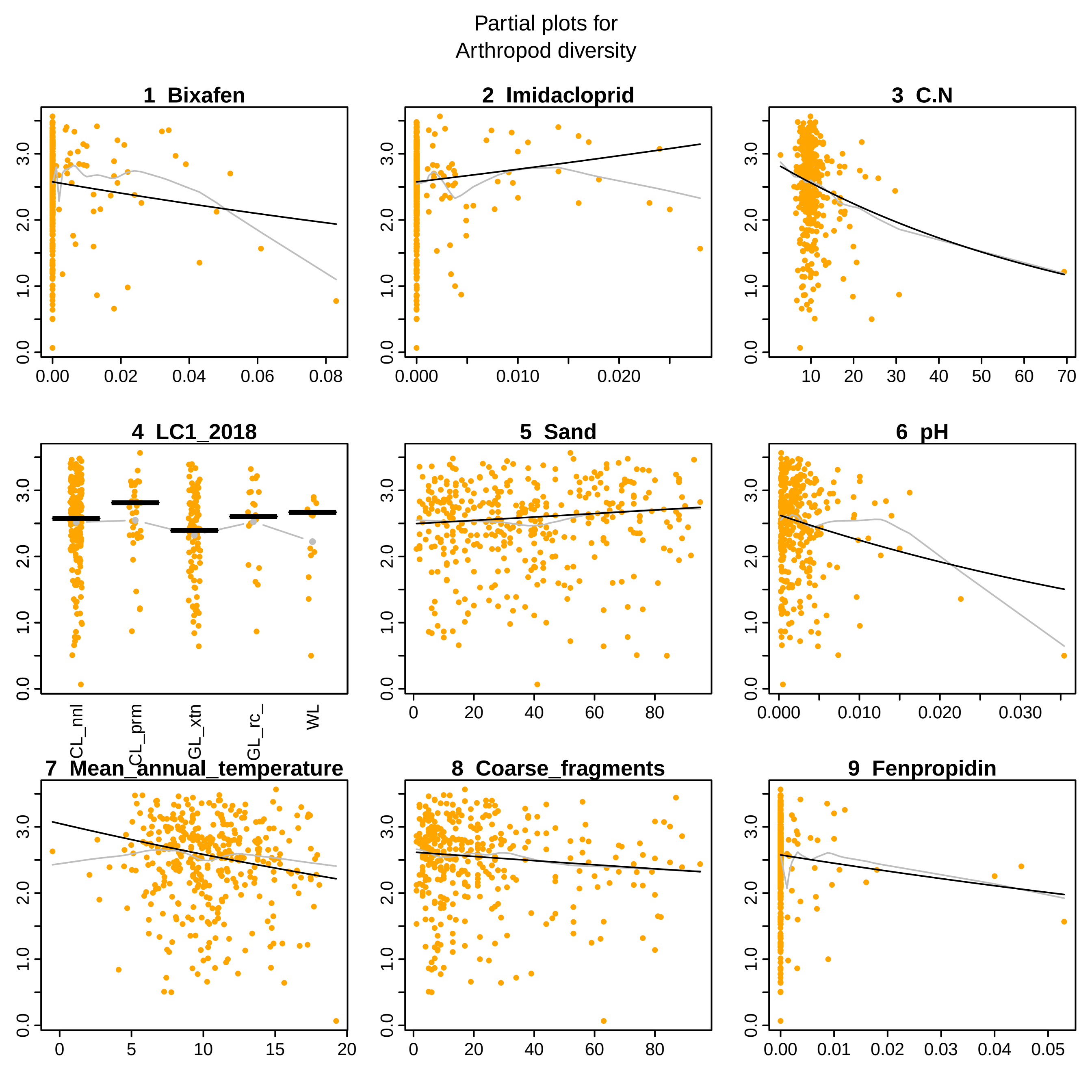

Partial plots for
Arthropod diversity
1 Bixafen
2 Imidacloprid
3 C.N
4 LC1_2018
5 Sand
6 pH
7 Mean_annual_temperature
8 Coarse_fragments
9 Fenpropidin
CL_nnl
CL_prm
GL_xtn
GL_rc_
WL
0.0
1.0
2.0
3.0
0.00
0.02
0.04
0.06
0.08
0.000
0.010
0.020
0.030
10
20
30
40
50
60
70
80
0
5
15
0.01
0.03
0.05

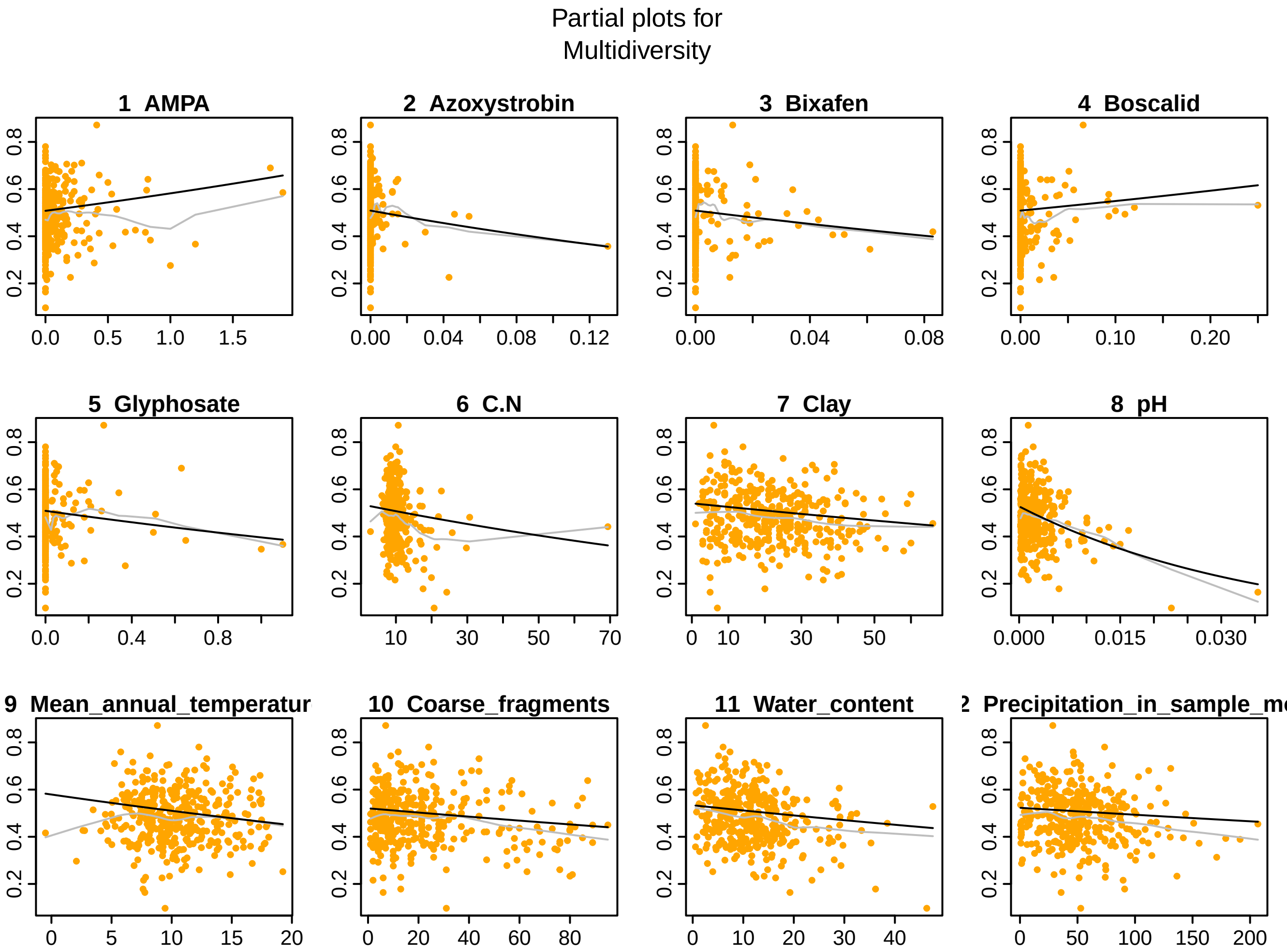

Partial plots for
Multidiversity
1 AMPA
2 Azoxystrobin
3 Bixafen
4 Boscalid
5 Glyphosate
6 C.N
7 Clay
8 pH
9 Mean_annual_temperatur
10 Coarse_fragments
11 Water_content
2 Precipitation_in_sample_m

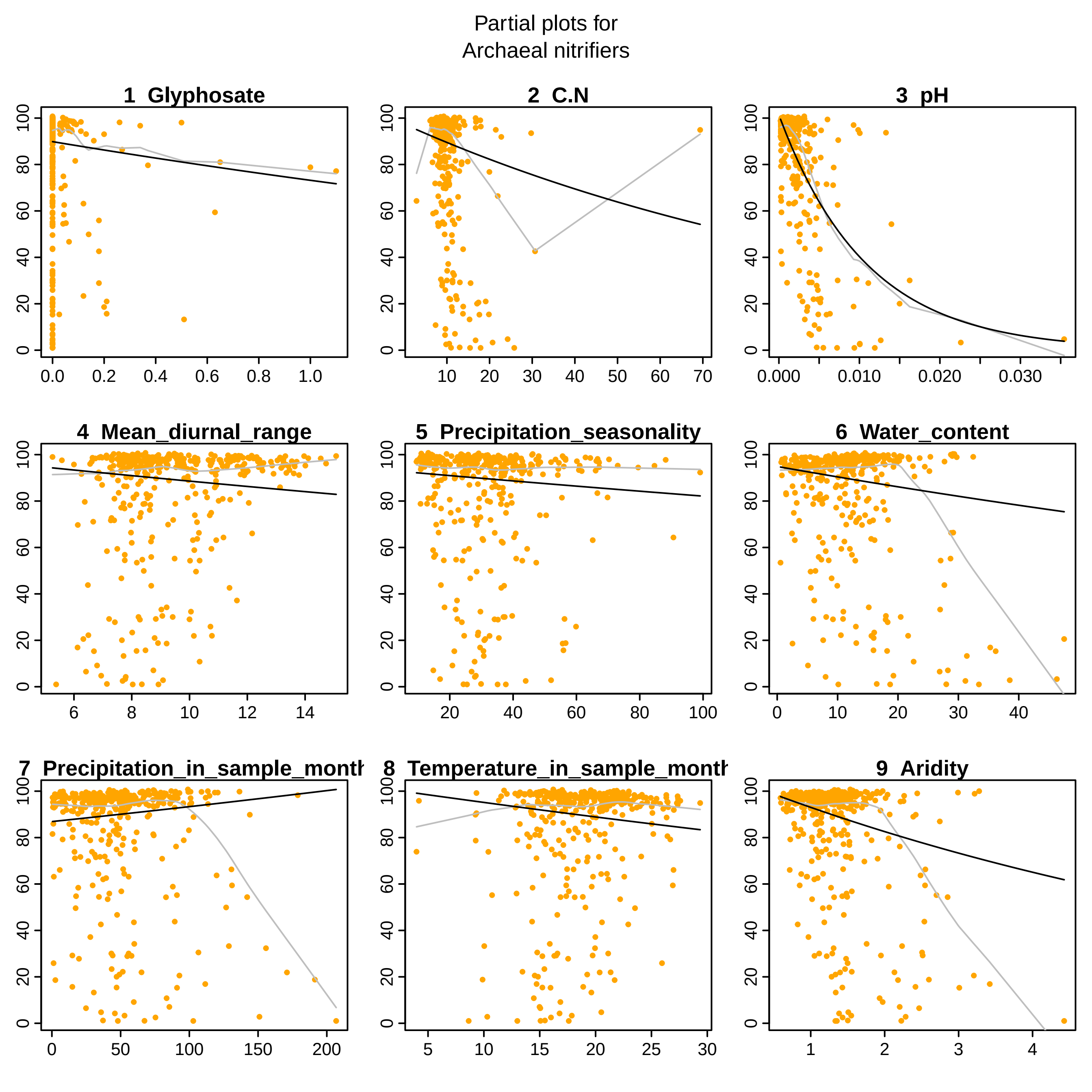

Partial plots for
Archaeal nitrifiers
1 Glyphosate
2 C.N
3 pH
4 Mean_diurnal_range
5 Precipitation_seasonality
6 Water_content
7 Precipitation_in_sample_montl
8 Temperature_in_sample_montl
9 Aridity

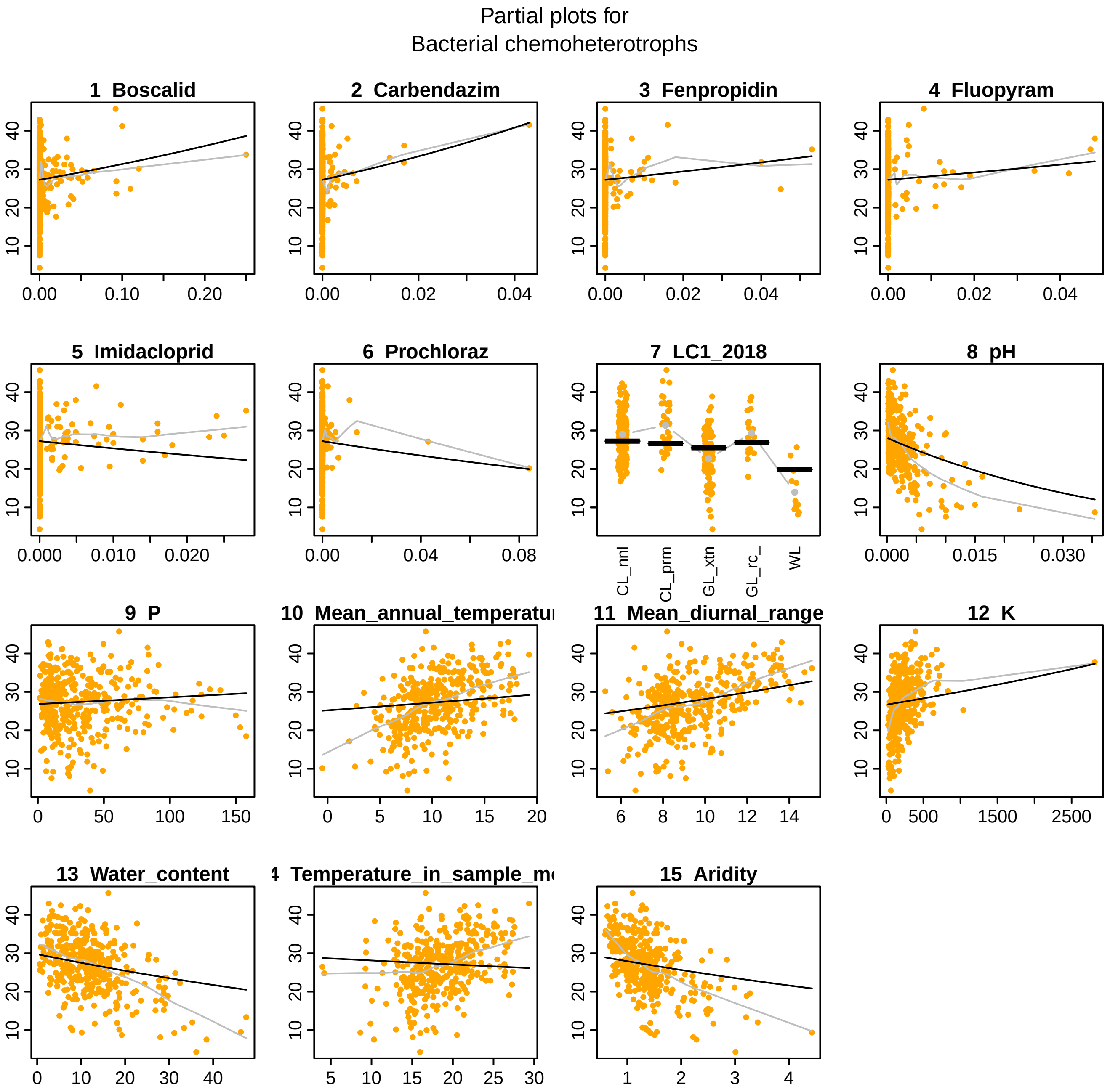
Partial plots for
Bacterial chemoheterotrophs
1 Boscalid
2 Carbendazim
3 Fenpropidin
4 Fluopyram
5 Imidacloprid
6 Prochloraz
7 LC1_2018
8 pH
9 P
10 Mean_annual_temperatu
11 Mean_diurnal_range
12 K
13 Water_content
4 Temperature_in_sample_m
15 Aridity
CL_nnl
CL_prm
GL_xtn
GL_rc_
WL

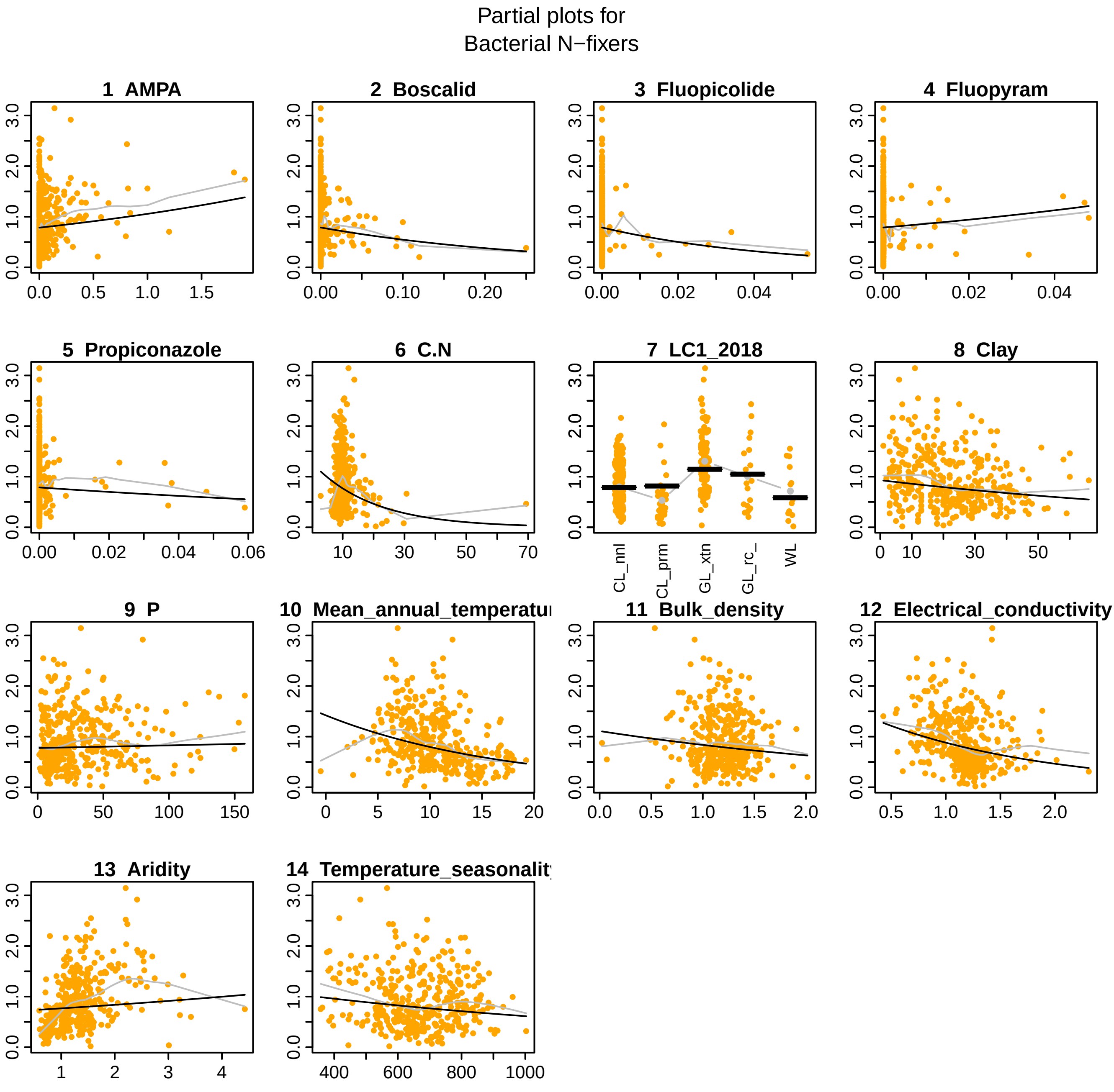
Partial plots for
Bacterial N−fixers
1 AMPA
2 Boscalid
3 Fluopicolide
4 Fluopyram
5 Propiconazole
6 C.N
7 LC1_2018
CL_nnl
CL_prm
GL_xtn
GL_rc_
WL
8 Clay
9 P
10 Mean_annual_temperatur
11 Bulk_density
12 Electrical_conductivity
13 Aridity
14 Temperature_seasonality

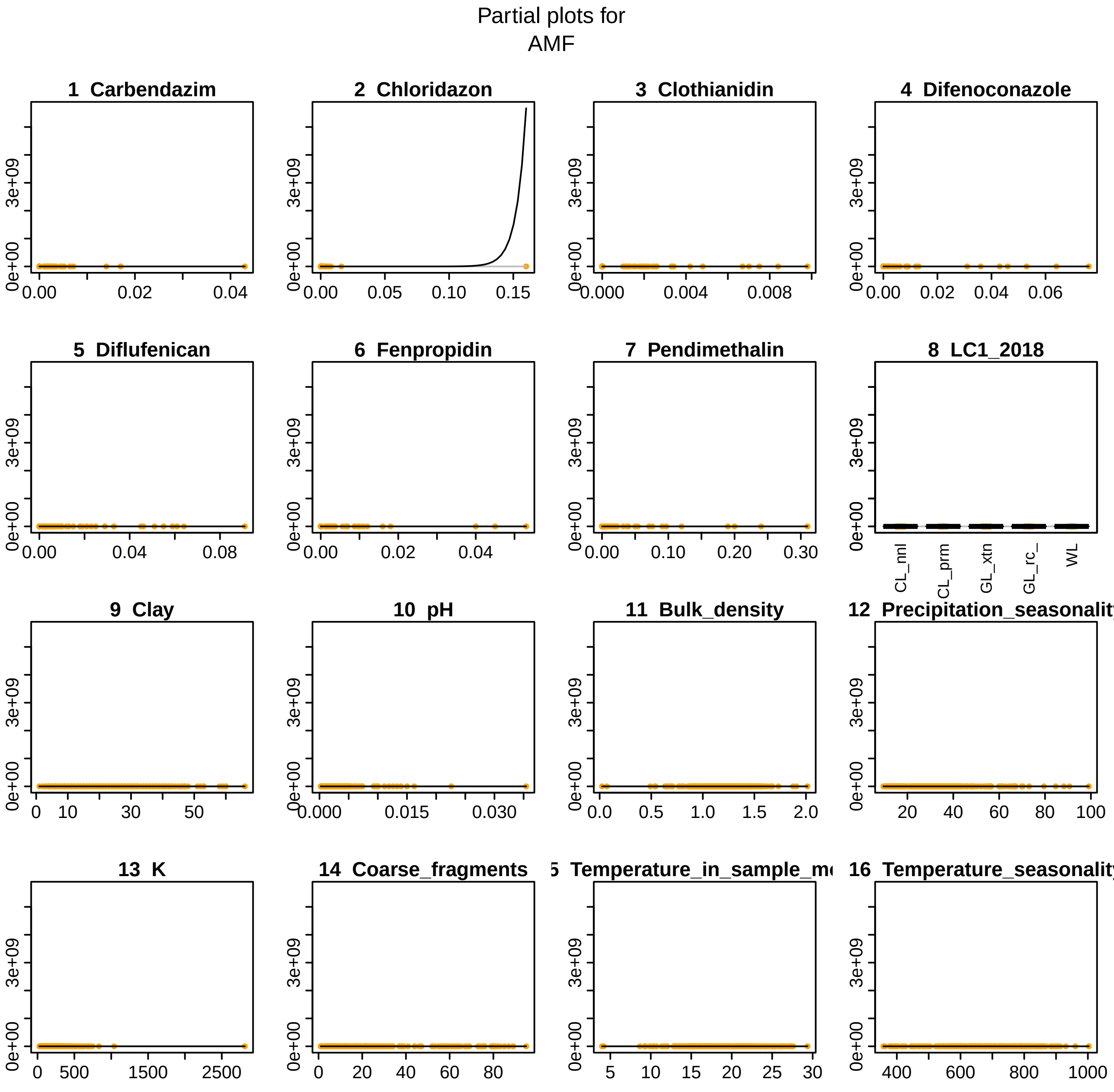

Partial plots for
AMF
1 Carbendazim
2 Chloridazon
3 Clothianidin
4 Difenoconazole
5 Diflufenican
6 Fenpropidin
7 Pendimethalin
8 LC1_2018
9 Clay
10 pH
11 Bulk_density
12 Precipitation_seasonalit
13 K
14 Coarse_fragments
5 Temperature_in_sample_m
16 Temperature_seasonalit
0e+00
3e+09
CL_nnl
CL_prm
GL_xtn
GL_rc_
WL

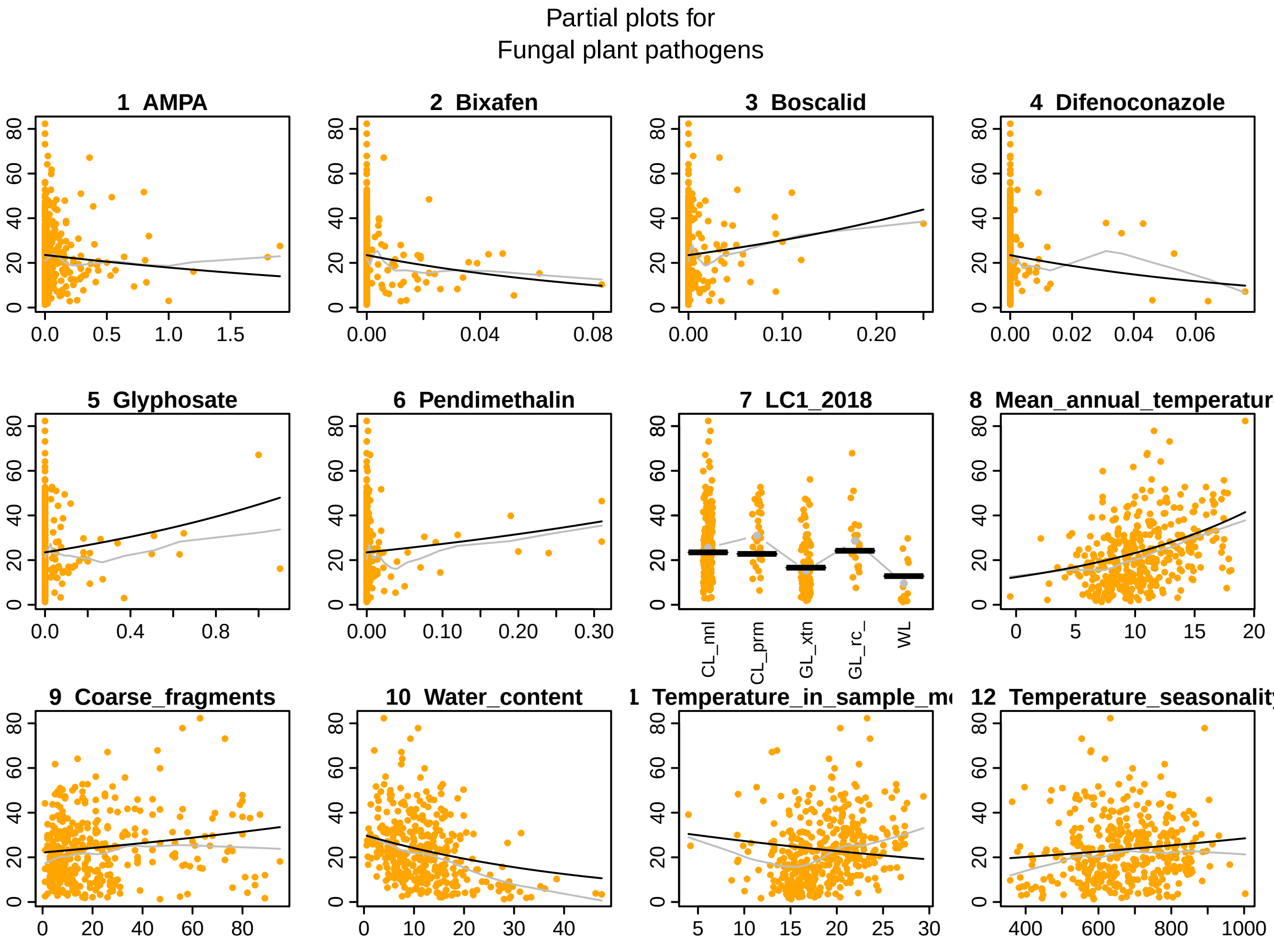
Partial plots for
Fungal plant pathogens
1 AMPA
2 Bixafen
3 Boscalid
4 Difenoconazole
5 Glyphosate
6 Pendimethalin
7 LC1_2018
8 Mean_annual_temperatur
9 Coarse_fragments
10 Water_content
1 Temperature_in_sample_mo
12 Temperature_seasonality
0 20 40 60 80
0.0 0.5 1.0 1.5
0.00 0.04 0.08
0.00 0.10 0.20
0.00 0.02 0.04 0.06
0.0 0.4 0.8
0.00 0.10 0.20 0.30
CL_nnl
CL_prm
GL_xtn
GL_rc_
WL
0 5 10 15 20
0 20 40 60 80
0 10 20 30 40
5 10 15 20 25 30
400 600 800 1000

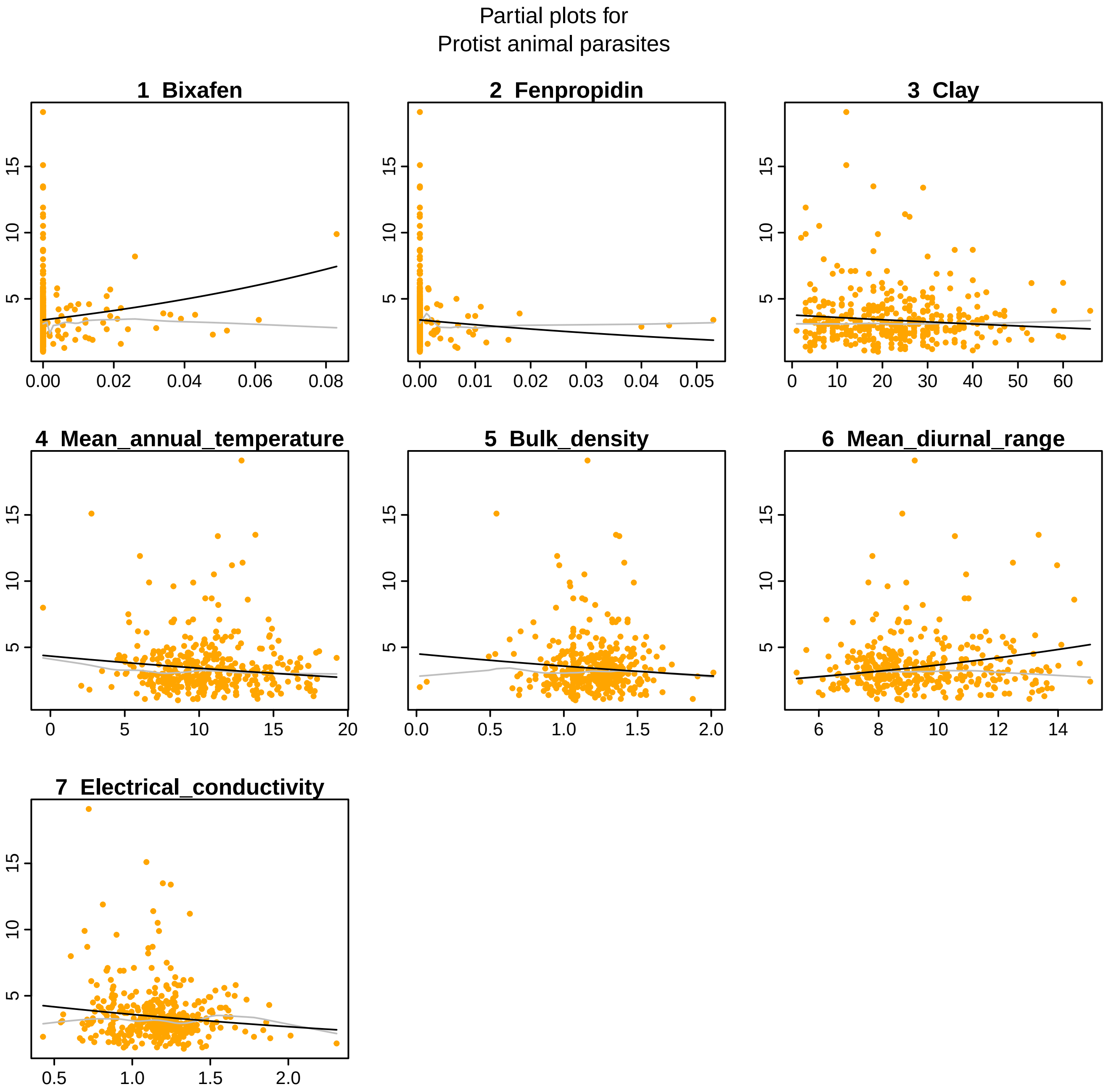
Partial plots for
Protist animal parasites
1 Bixafen
2 Fenpropidin
3 Clay
4 Mean_annual_temperature
5 Bulk_density
6 Mean_diurnal_range
7 Electrical_conductivity
0.00 0.02 0.04 0.06 0.08
0.00 0.01 0.02 0.03 0.04 0.05
0 10 20 30 40 50 60
0 5 10 15 20
0.0 0.5 1.0 1.5 2.0
6 8 10 12 14
0.5 1.0 1.5 2.0
5 10 15

Partial plots for
Protist plant parasites

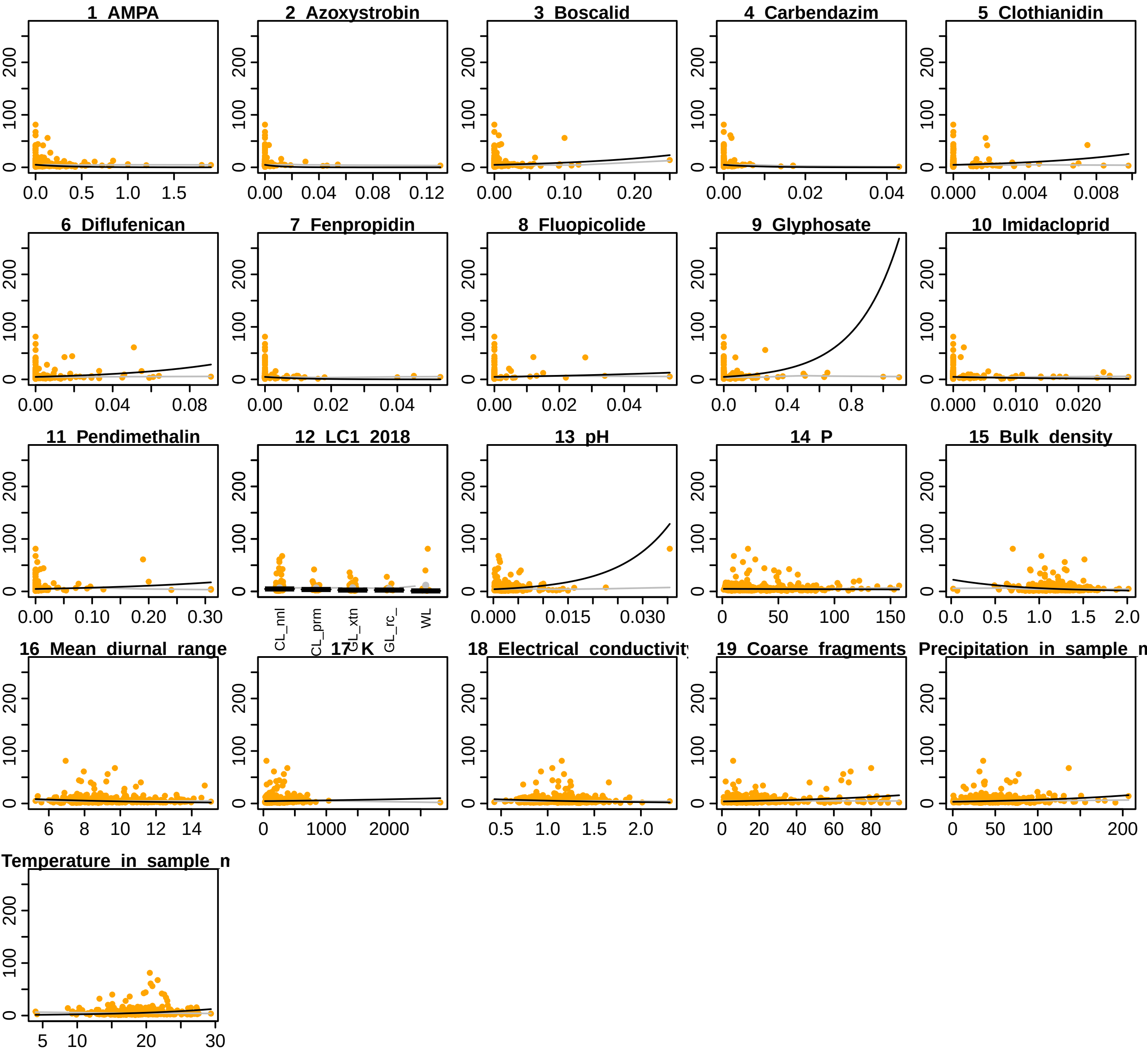

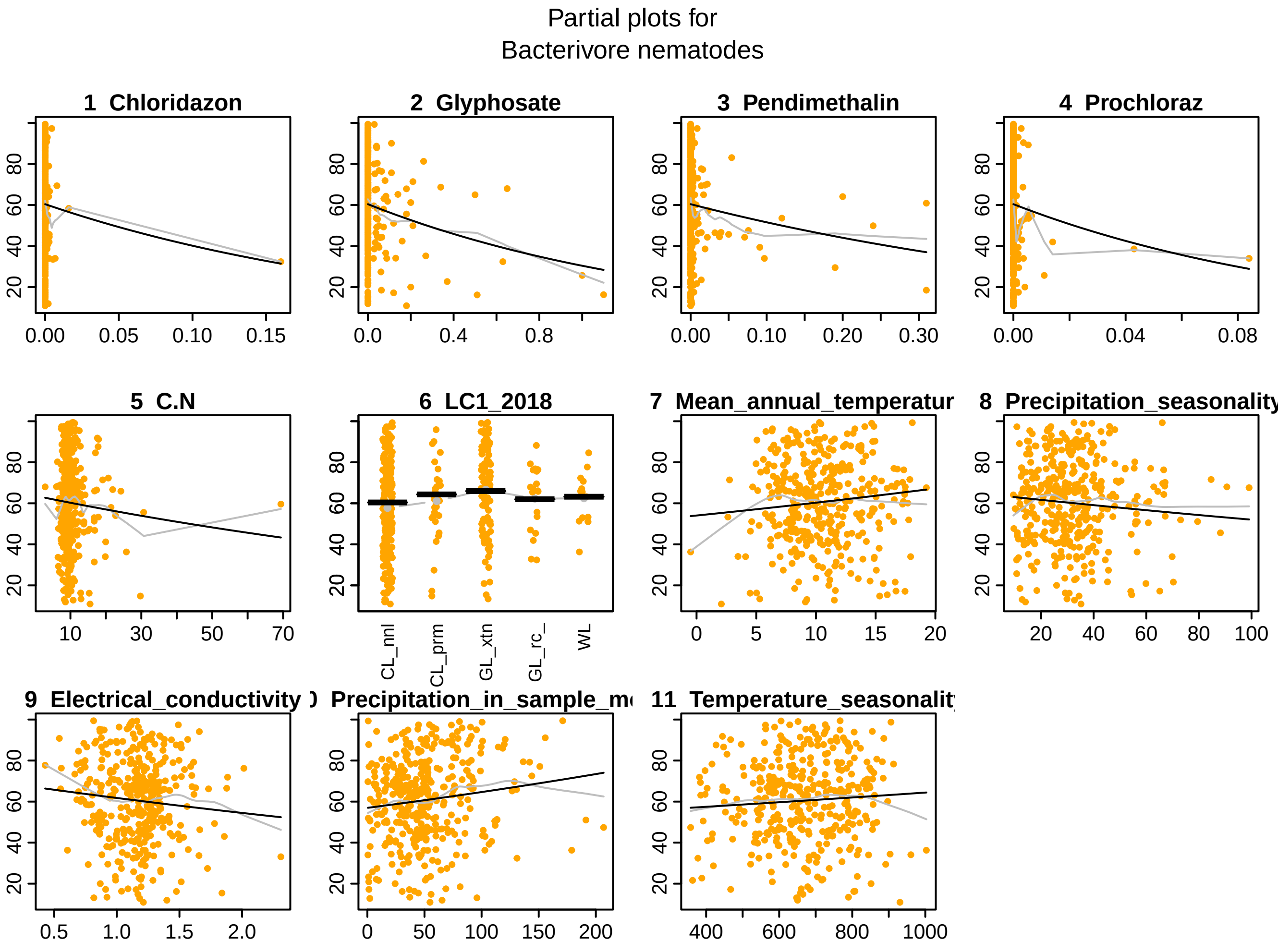
Partial plots for
Bacterivore nematodes
1 Chloridazon
2 Glyphosate
3 Pendimethalin
4 Prochloraz
5 C.N
6 LC1_2018
7 Mean_annual_temperatur
8 Precipitation_seasonality
9 Electrical_conductivity
) Precipitation_in_sample_m
11 Temperature_seasonalit
CL_nnl
CL_prm
GL_xtn
GL_rc_
WL

Partial plots for
Herbivore nematodes

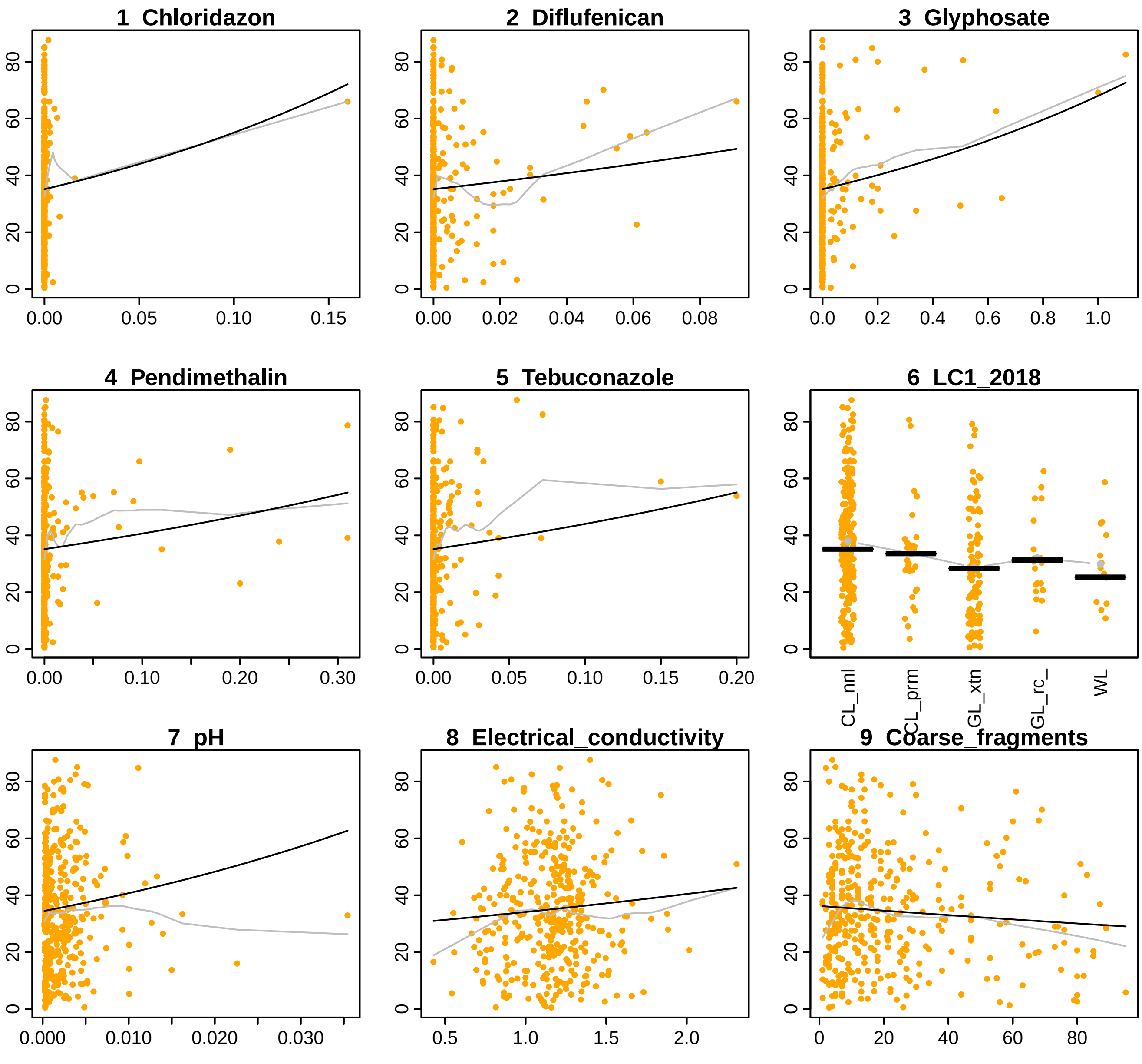

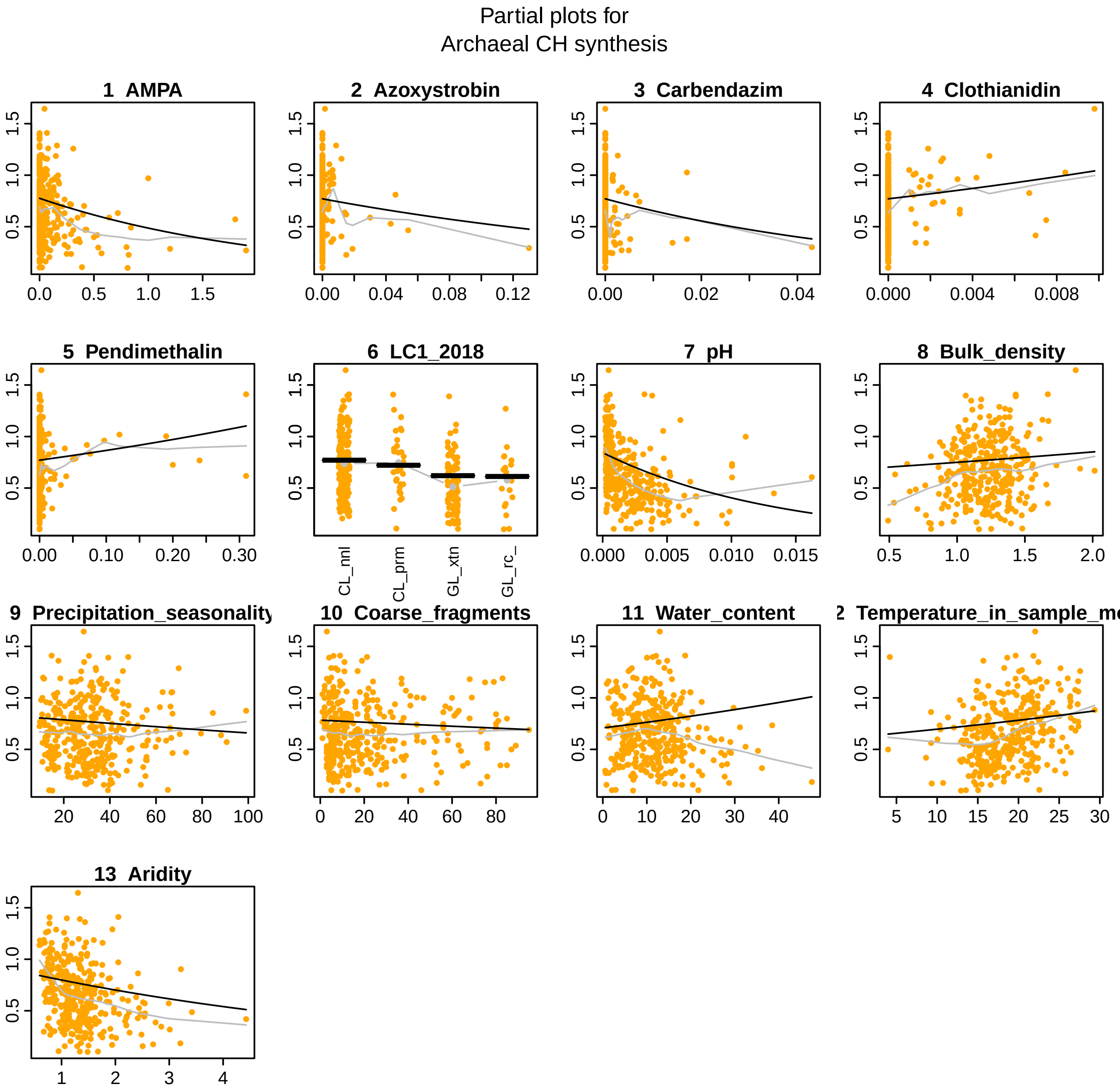
Partial plots for
Archaeal CH synthesis
1 AMPA
2 Azoxystrobin
3 Carbendazim
4 Clothianidin
5 Pendimethalin
6 LC1_2018
7 pH
8 Bulk_density
9 Precipitation_seasonality
10 Coarse_fragments
11 Water_content
2 Temperature_in_sample_mo
13 Aridity
CL_nnl
CL_prm
GL_xtn
GL_rc_

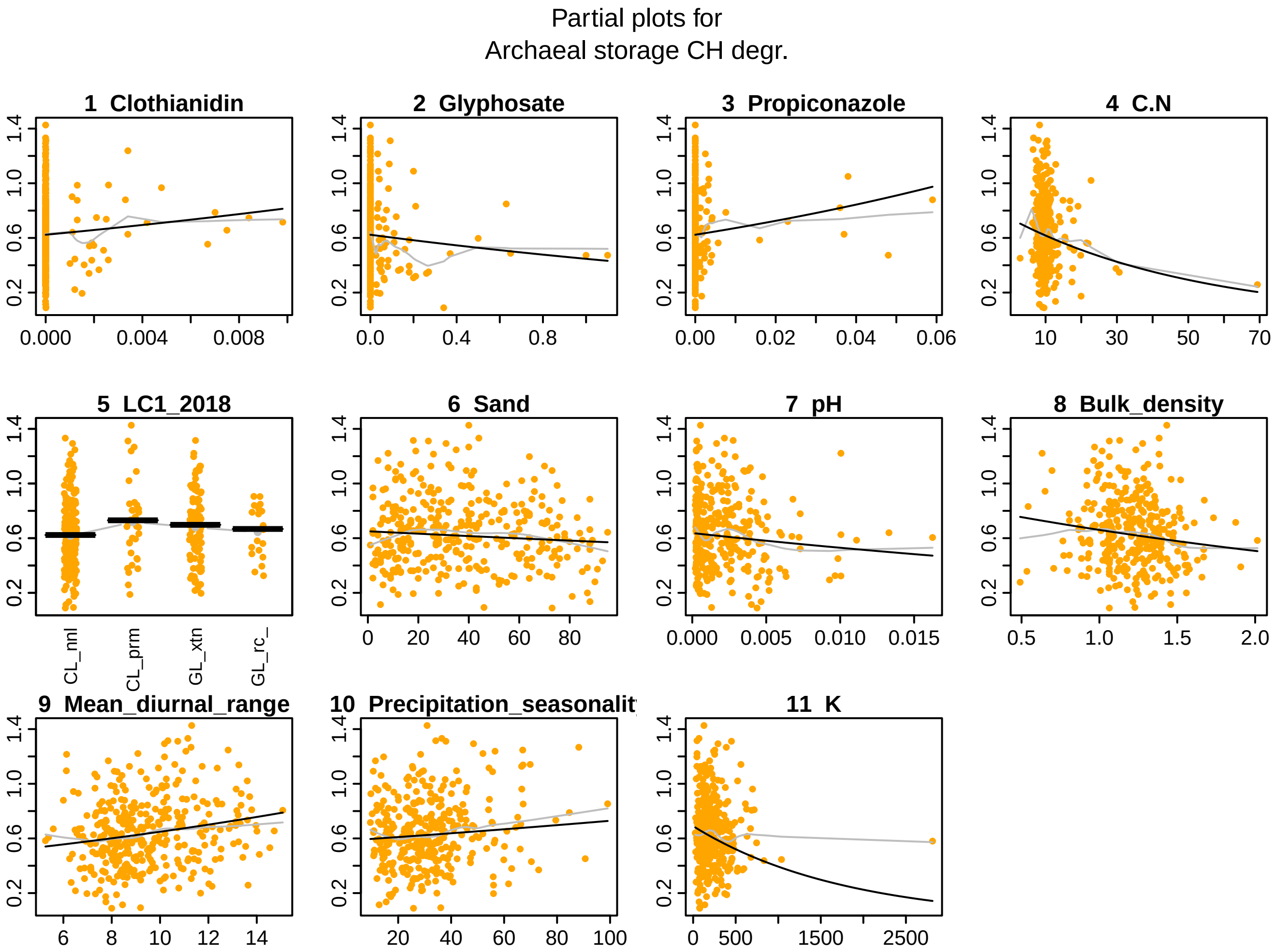
Partial plots for
Archaeal storage CH degr.
1 Clothianidin
2 Glyphosate
3 Propiconazole
4 C.N
5 LC1_2018
CL_nnl
CL_prm
GL_xtn
GL_rc_
6 Sand
7 pH
8 Bulk_density
9 Mean_diurnal_range
10 Precipitation_seasonalit
11 K

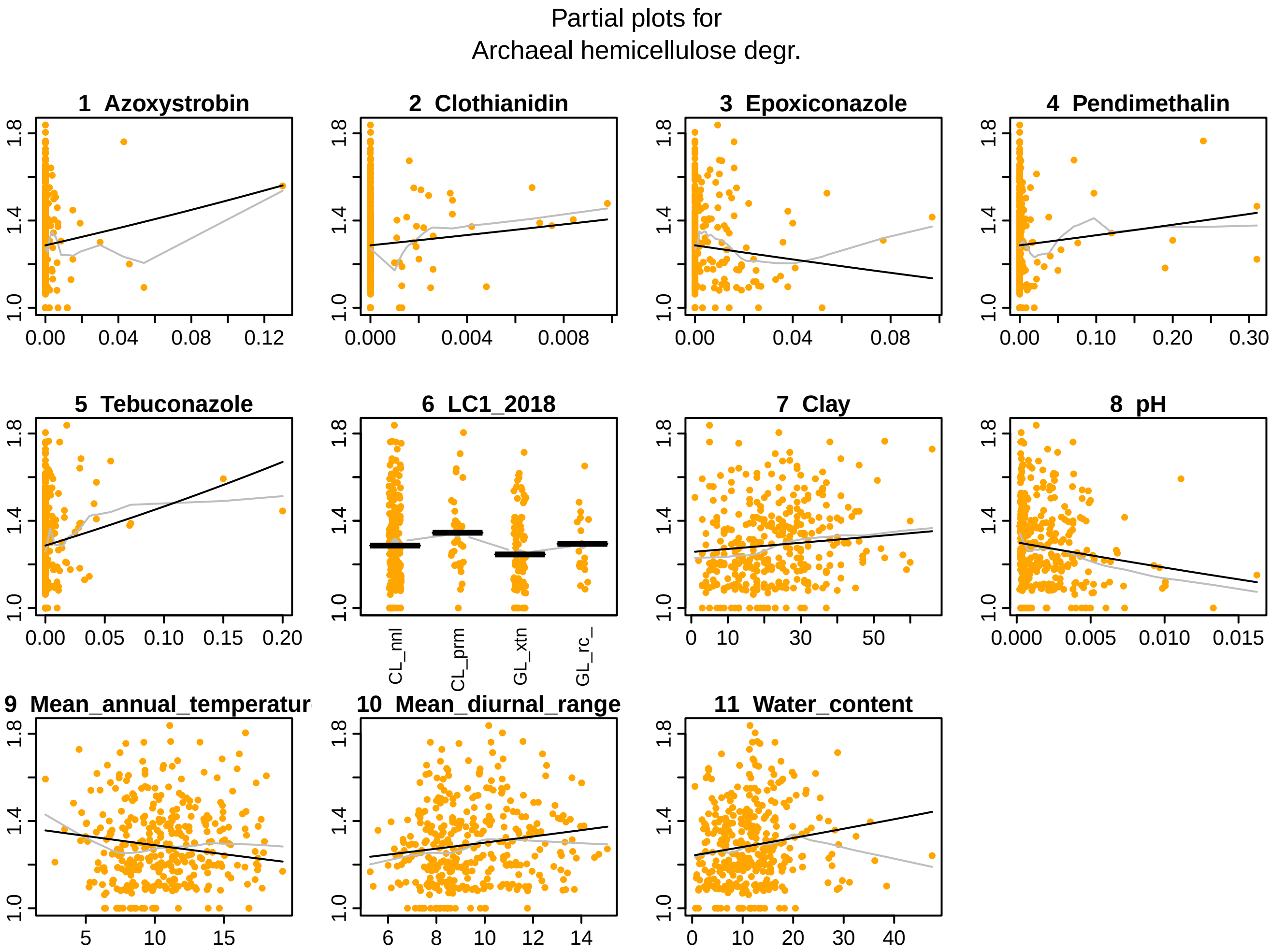

Partial plots for
Archaeal hemicellulose degr.
1 Azoxystrobin
2 Clothianidin
3 Epoxiconazole
4 Pendimethalin
5 Tebuconazole
6 LC1_2018
7 Clay
8 pH
9 Mean_annual_temperatur
10 Mean_diurnal_range
11 Water_content
CL_nnl
CL_prm
GL_xtn
GL_rc_

Partial plots for
Archaeal cellulose degr.

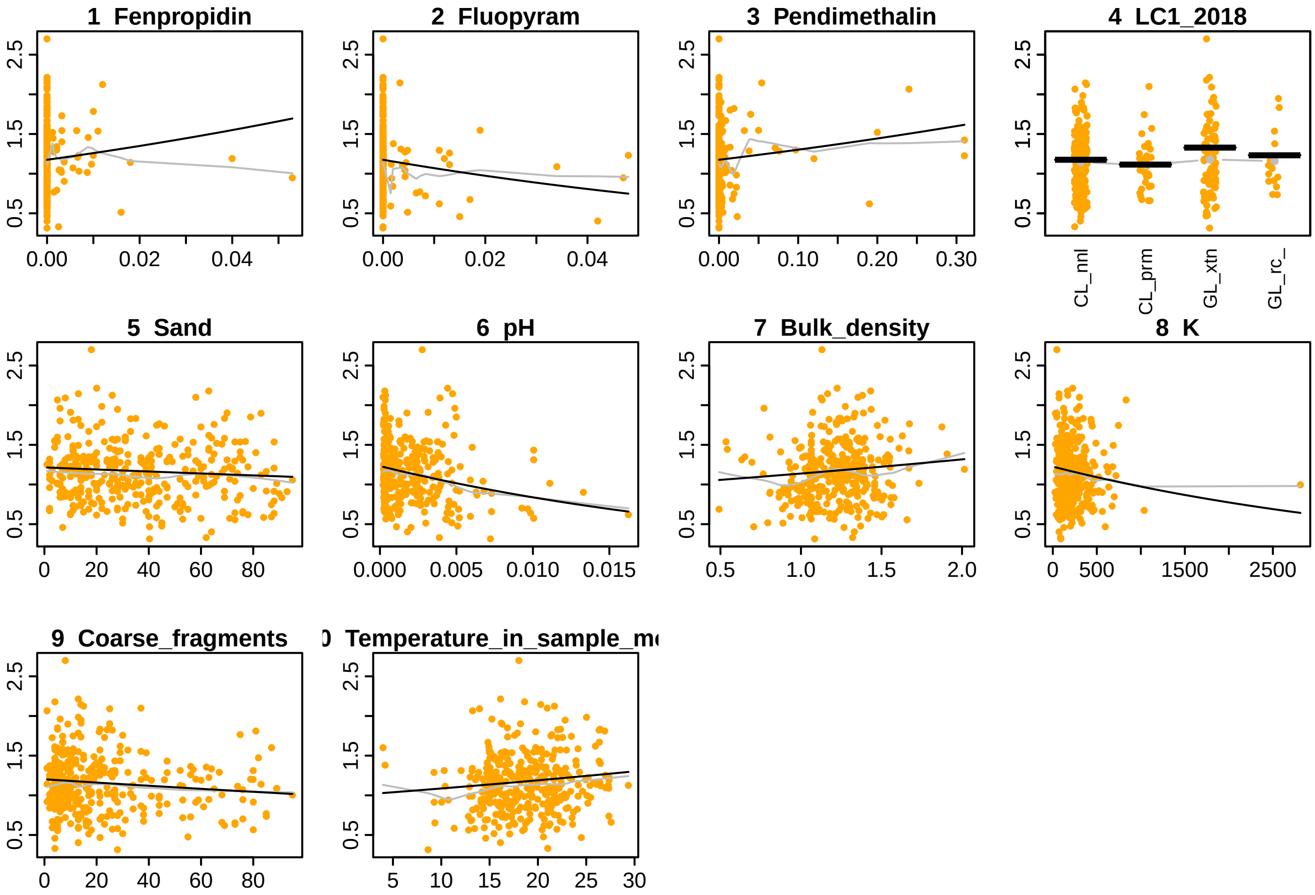

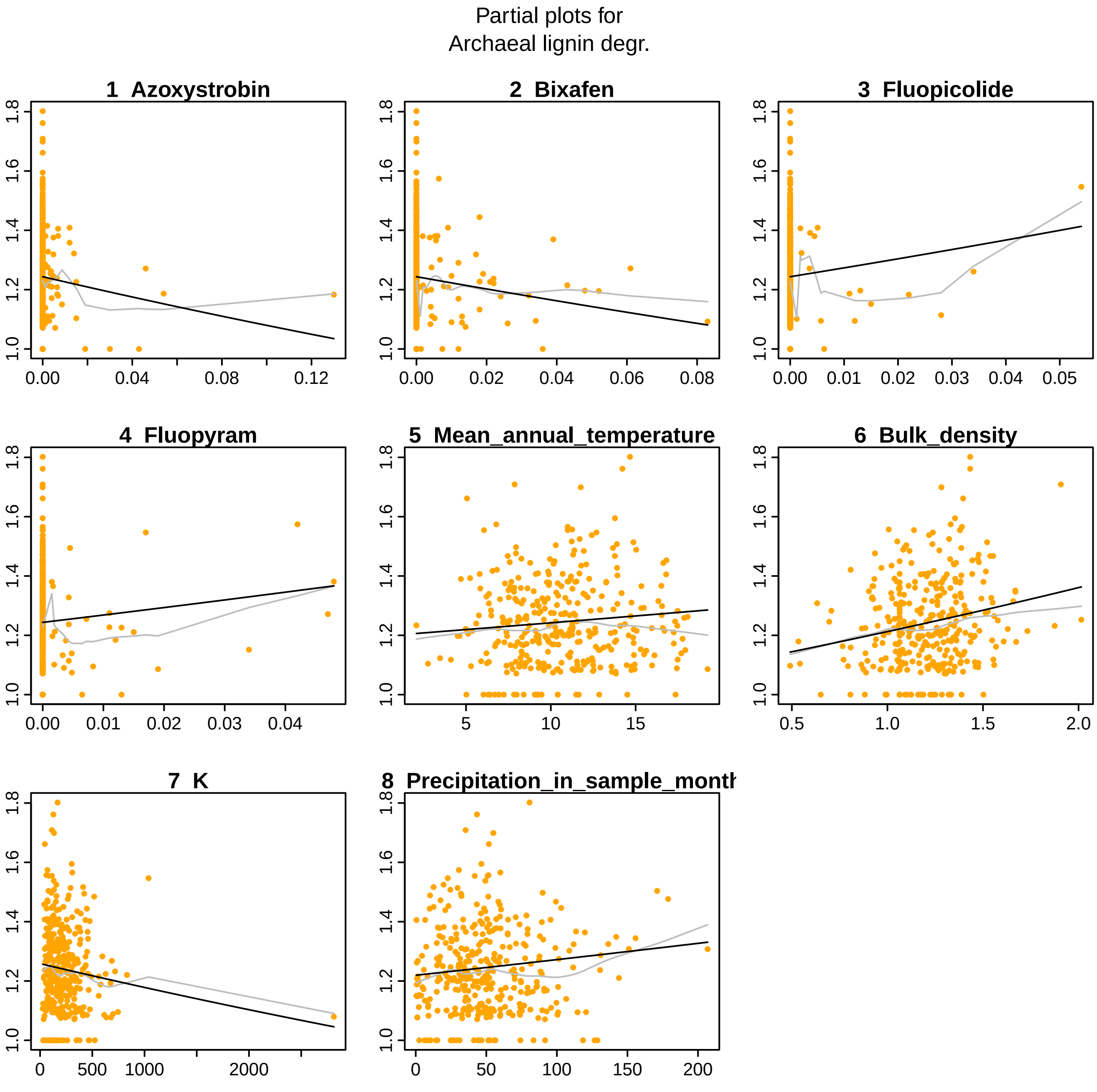

Partial plots for
Archaeal lignin degr.
1 Azoxystrobin
2 Bixafen
3 Fluopicolide
4 Fluopyram
5 Mean_annual_temperature
6 Bulk_density
7 K
8 Precipitation_in_sample_montl

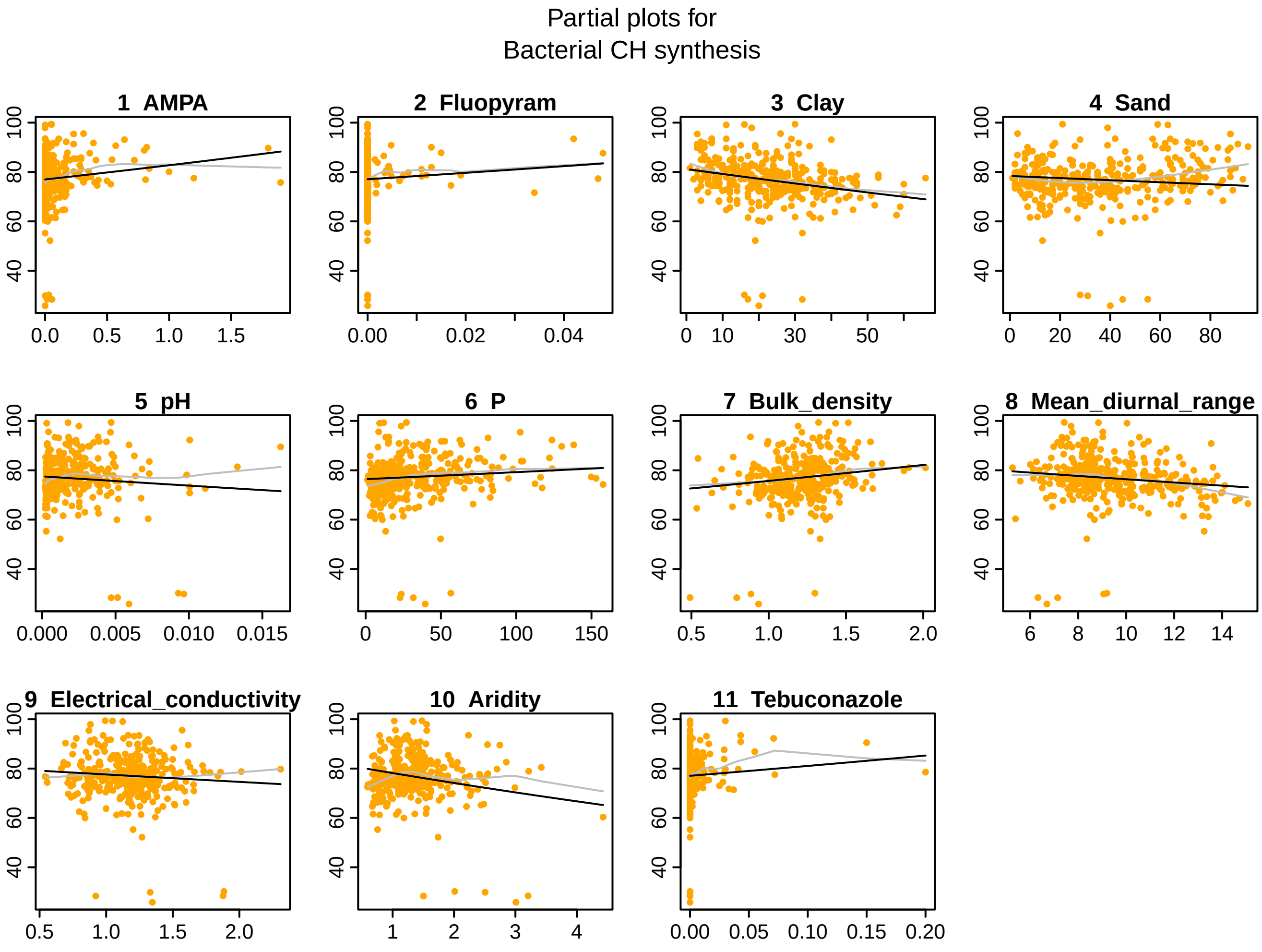

Partial plots for
Bacterial CH synthesis
1 AMPA
2 Fluopyram
3 Clay
4 Sand
5 pH
6 P
7 Bulk_density
8 Mean_diurnal_range
9 Electrical_conductivity
10 Aridity
11 Tebuconazole

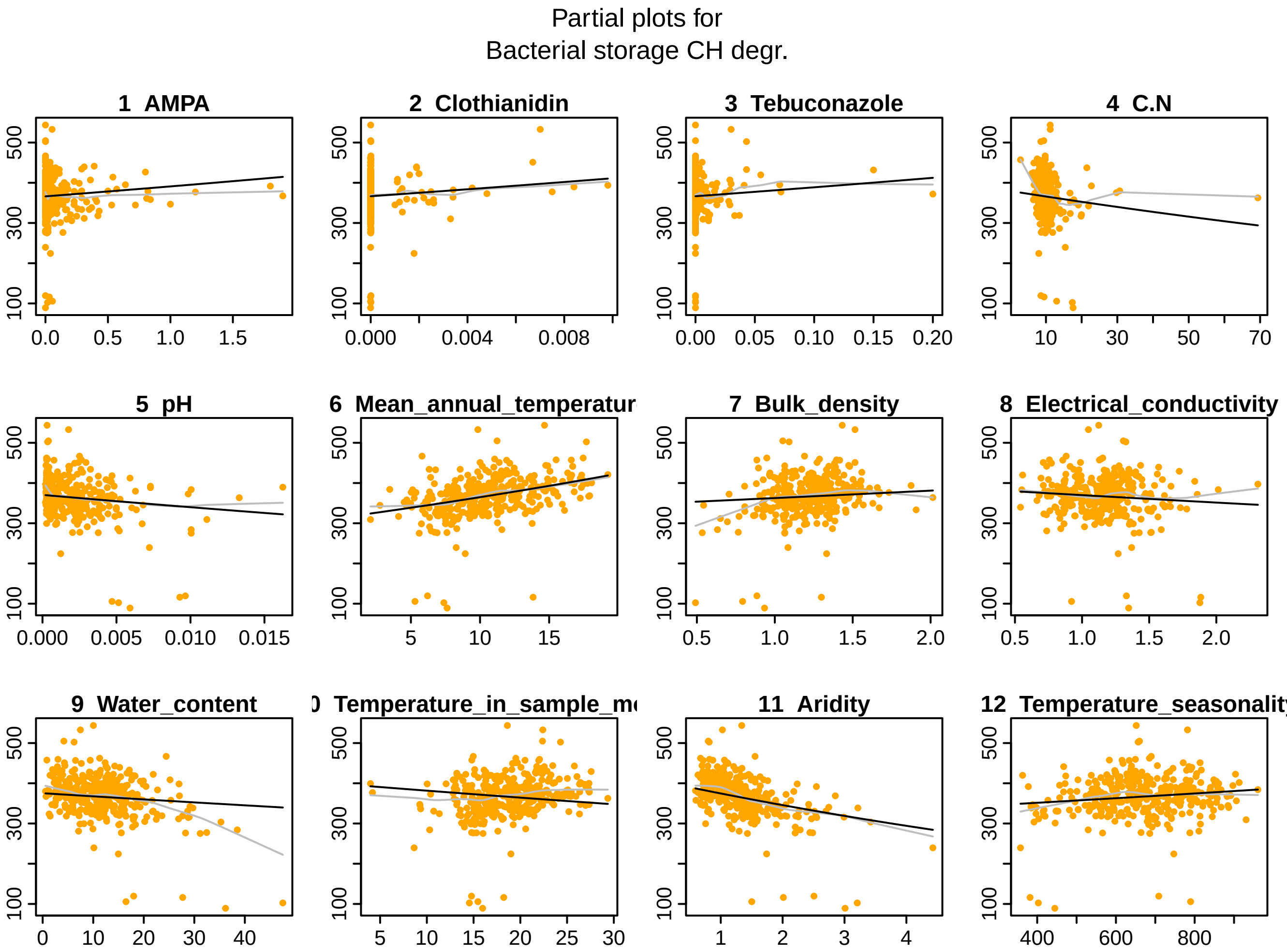

Partial plots for
Bacterial storage CH degr.
1 AMPA
2 Clothianidin
3 Tebuconazole
4 C.N
5 pH
6 Mean_annual_temperatur
7 Bulk_density
8 Electrical_conductivity
9 Water_content
0 Temperature_in_sample_mo
11 Aridity
12 Temperature_seasonality
100
300
500
0.0
0.5
1.0
1.5
0.000
0.004
0.008
0.00
0.05
0.10
0.15
0.20
10
30
50
70
0.005
0.010
0.015
5
15
2.0
0
20
40
25
1
2
3
4
400
600
800

Partial plots for
Bacterial pectin degr.

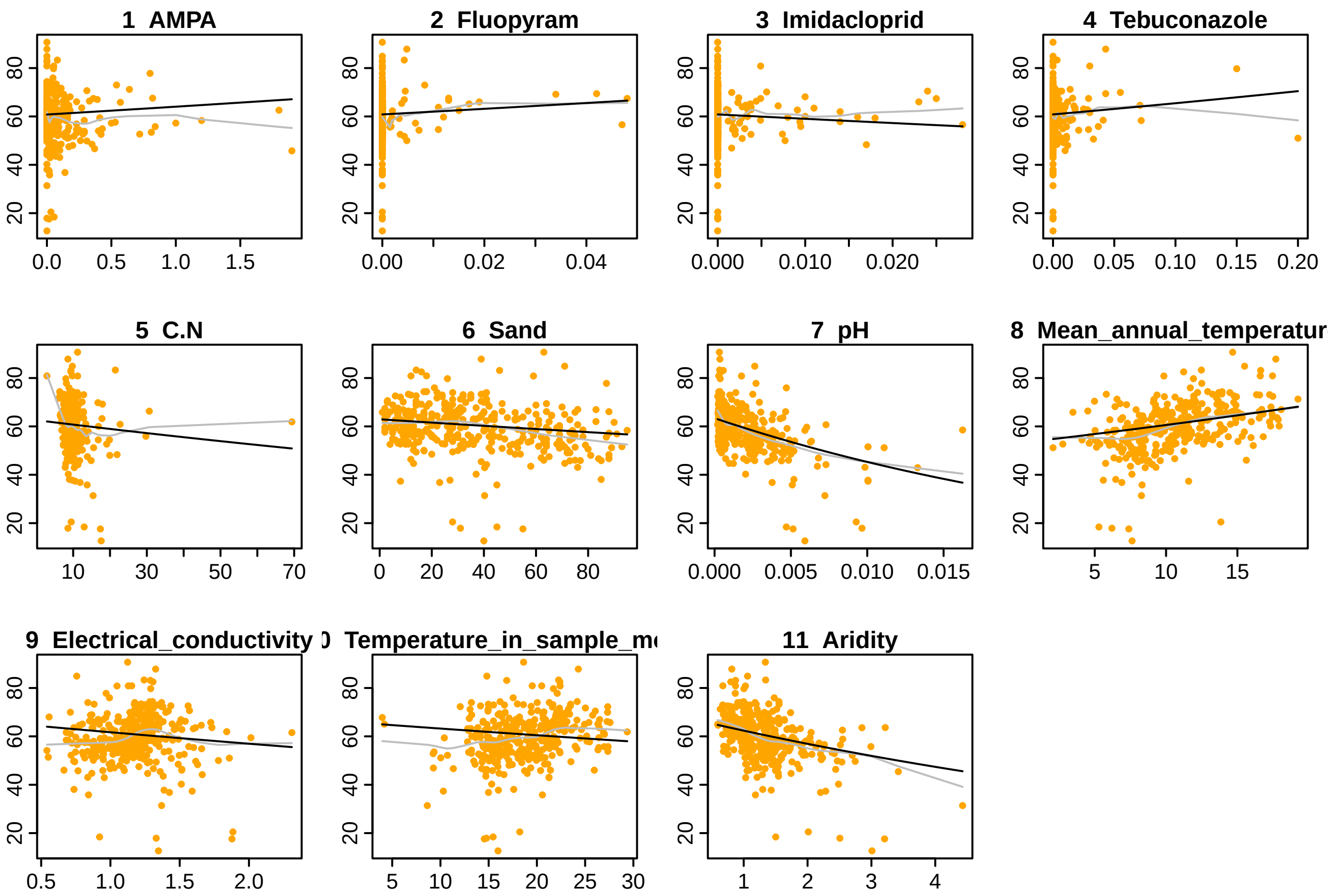

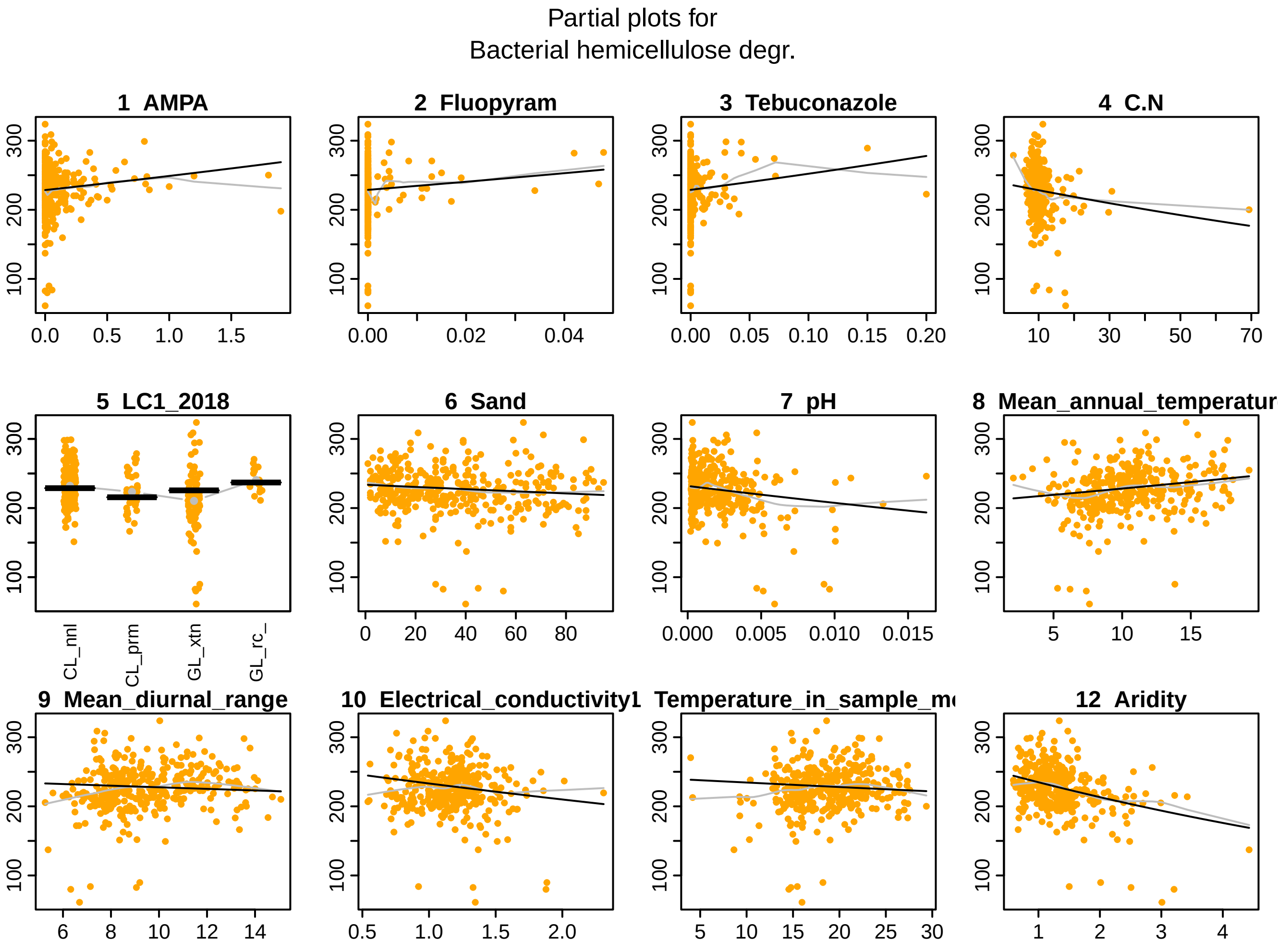
Partial plots for
Bacterial hemicellulose degr.
1 AMPA
2 Fluopyram
3 Tebuconazole
4 C.N
5 LC1_2018
CL_nnl
CL_prm
GL_xtn
GL_rc_
6 Sand
7 pH
8 Mean_annual_temperatur
9 Mean_diurnal_range
10 Electrical_conductivity
Temperature_in_sample_m
12 Aridity

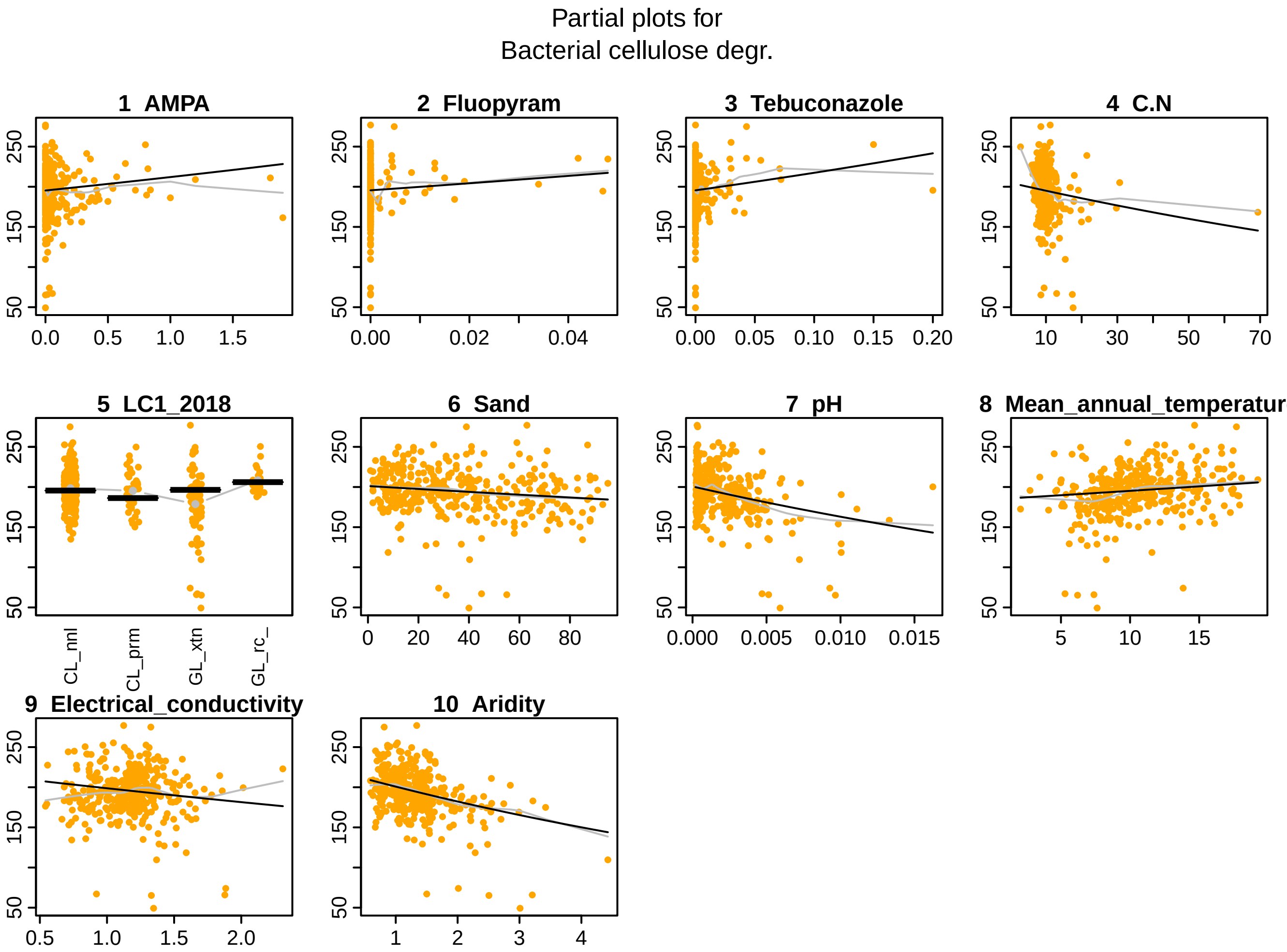
Partial plots for
Bacterial cellulose degr.
1 AMPA
2 Fluopyram
3 Tebuconazole
4 C.N
5 LC1_2018
CL_nnl
CL_prm
GL_xtn
GL_rc_
6 Sand
7 pH
8 Mean_annual_temperatur
9 Electrical_conductivity
10 Aridity

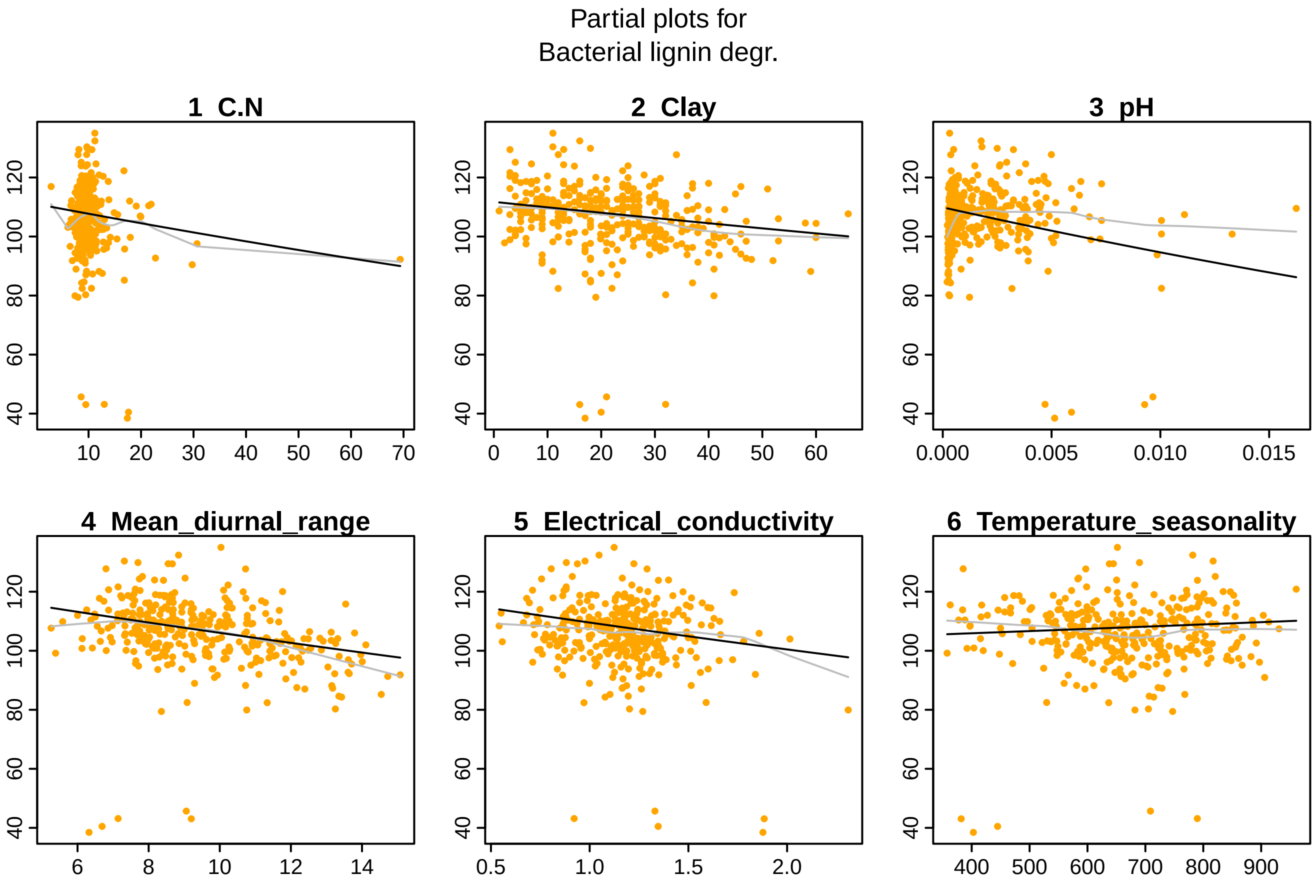
Partial plots for
Bacterial lignin degr.
1 C.N
2 Clay
3 pH
4 Mean_diurnal_range
5 Electrical_conductivity
6 Temperature_seasonality
40
60
80
100
120
10
20
30
40
50
60
70
0
0.000
0.005
0.010
0.015
6
8
10
12
14
0.5
1.0
1.5
2.0
400
500
600
700
800
900

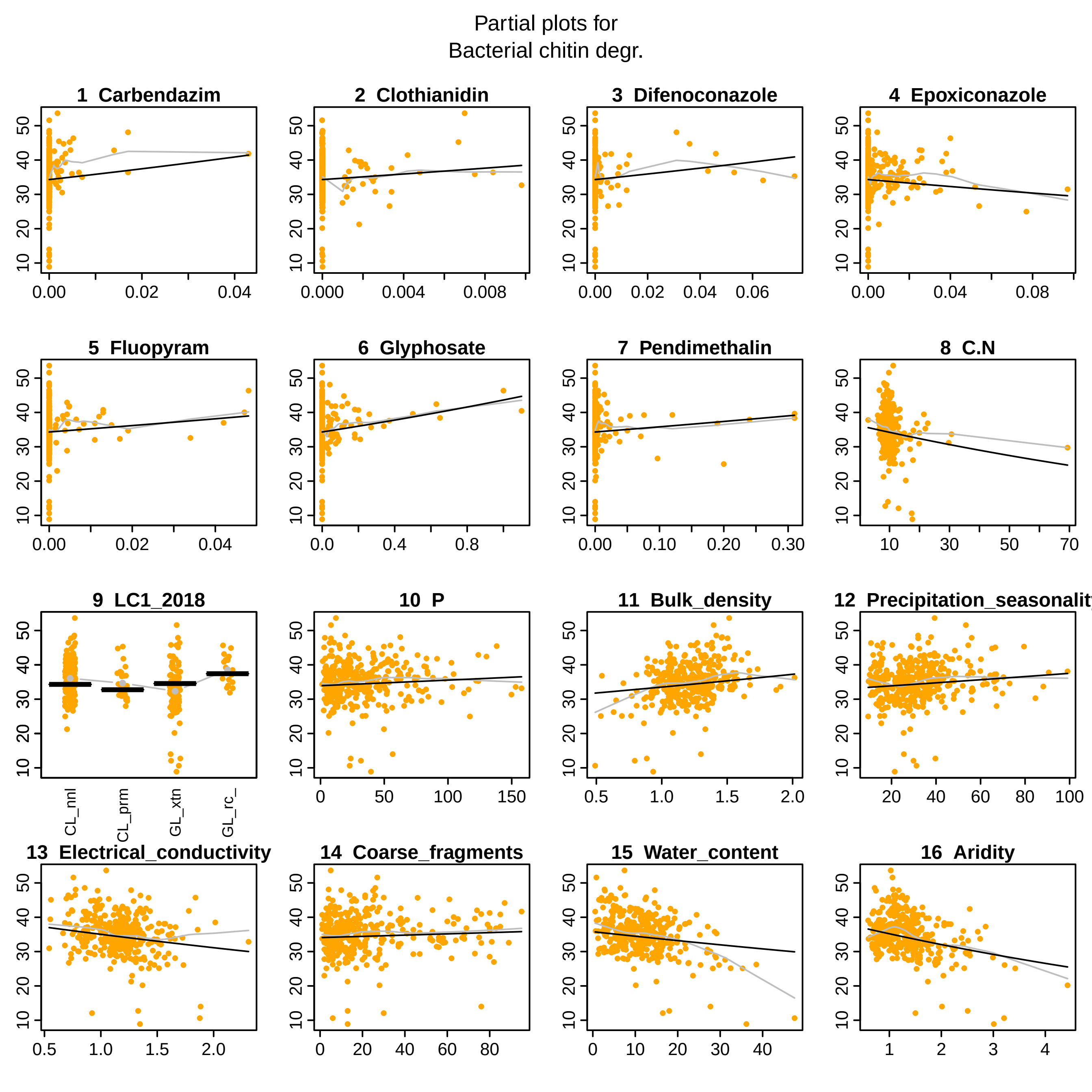
Partial plots for
Bacterial chitin degr.
1 Carbendazim
2 Clothianidin
3 Difenoconazole
4 Epoxiconazole
5 Fluopyram
6 Glyphosate
7 Pendimethalin
8 C.N
9 LC1_2018
10 P
11 Bulk_density
12 Precipitation_seasonalit
13 Electrical_conductivity
14 Coarse_fragments
15 Water_content
16 Aridity
CL_nnl
CL_prm
GL_xtn
GL_rc_
10 20 30 40 50
0.00 0.02 0.04
0.000 0.004 0.008
0.00 0.02 0.04 0.06
0.00 0.04 0.08
0.0 0.4 0.8
0.00 0.10 0.20 0.30
10 30 50 70
0 50 100 150
0.5 1.0 1.5 2.0
20 40 60 80 100
0 20 40 60 80
0 10 20 30 40
1 2 3 4

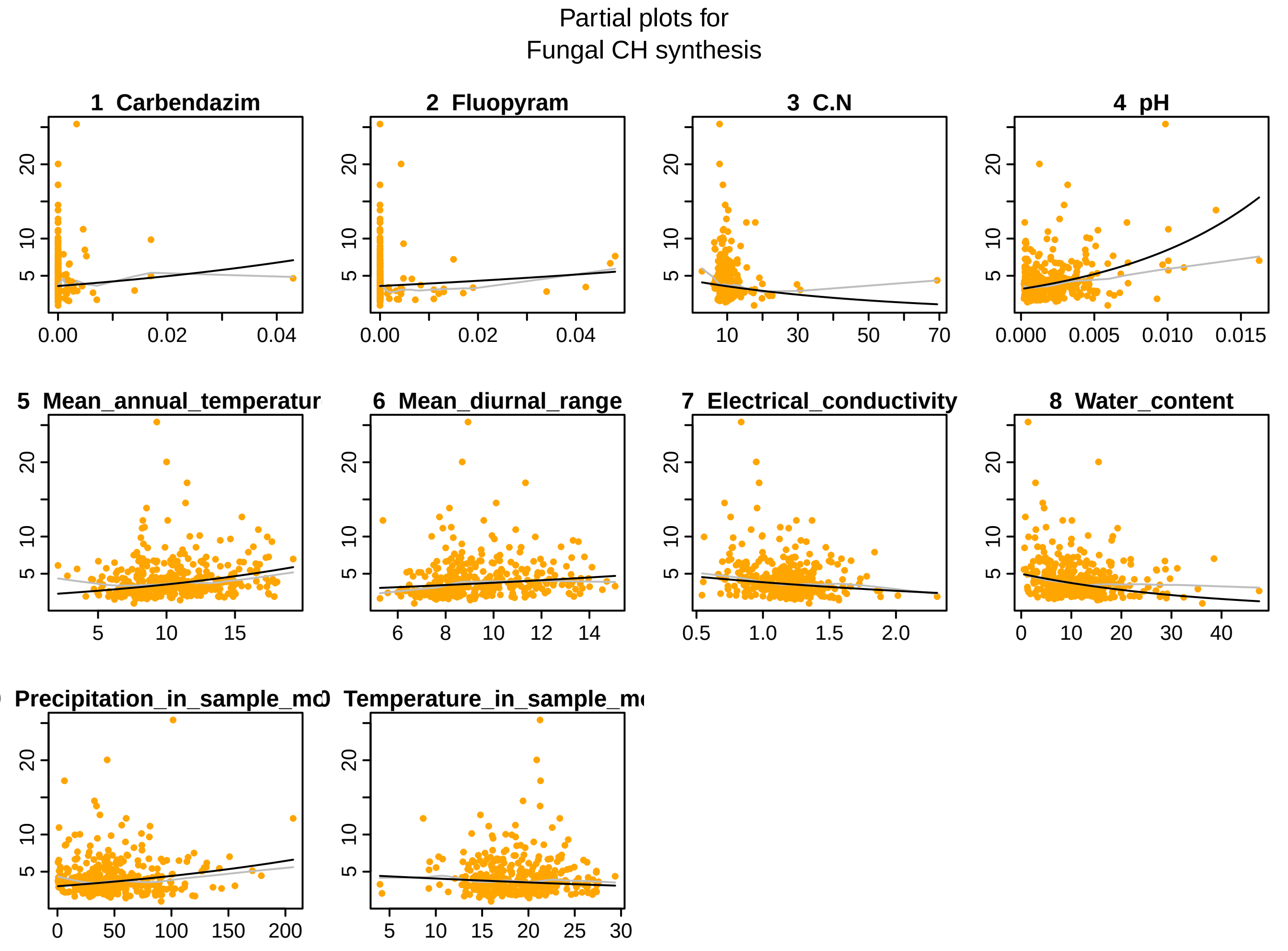
Partial plots for
Fungal CH synthesis
1 Carbendazim
2 Fluopyram
3 C.N
4 pH
5 Mean_annual_temperatur
6 Mean_diurnal_range
7 Electrical_conductivity
8 Water_content
Precipitation_in_sample_mo
Temperature_in_sample_m
5
10
20
0.00
0.02
0.04
10
30
50
70
0.000
0.005
0.010
0.015
5
10
15
6
8
10
12
14
0.5
1.0
1.5
2.0
0
10
20
30
40
0
50
100
150
200
5
10
15
20
25
30

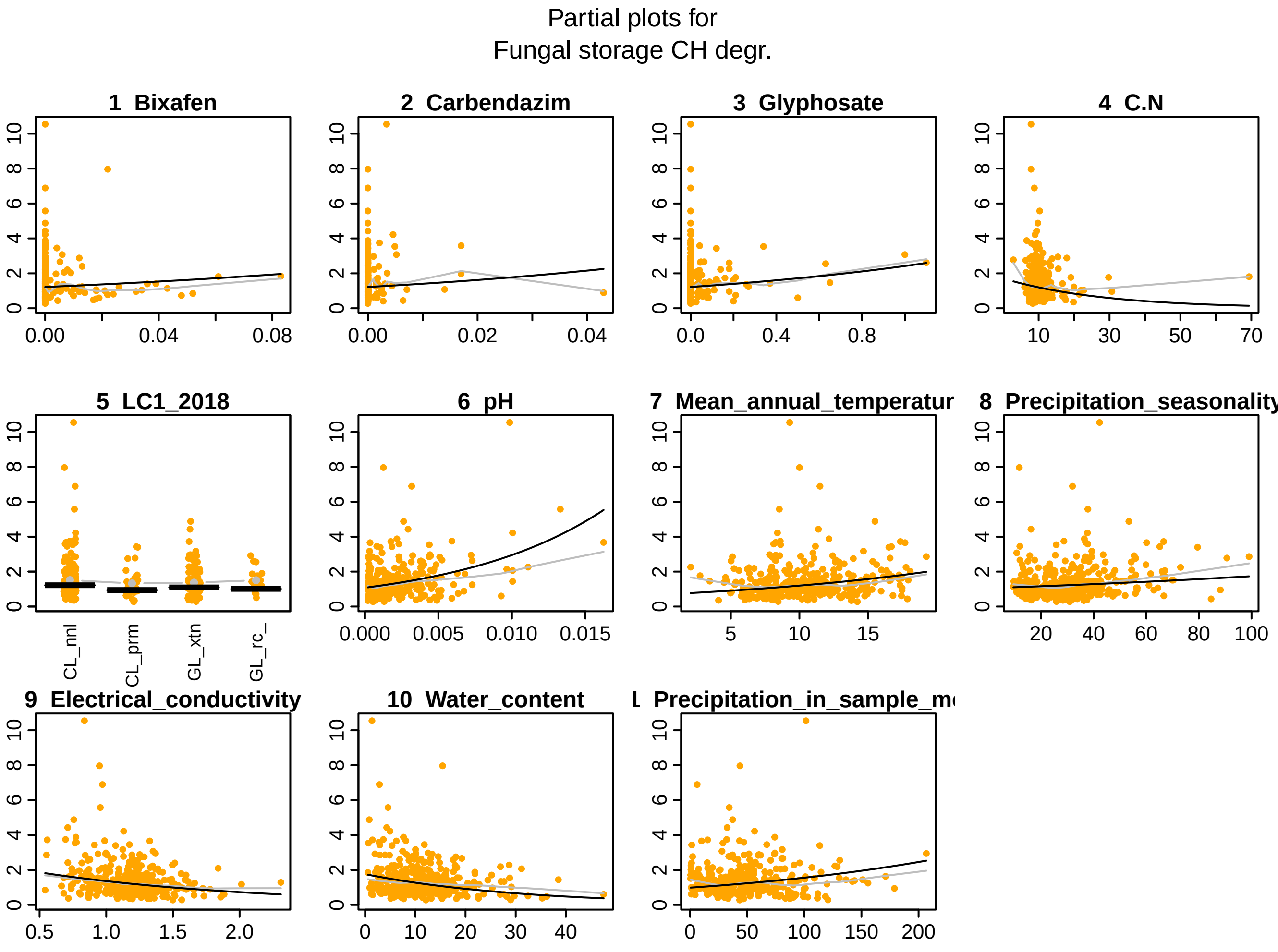
Partial plots for
Fungal storage CH degr.
1 Bixafen
2 Carbendazim
3 Glyphosate
4 C.N
5 LC1_2018
CL_nnl
CL_prm
GL_xtn
GL_rc_
6 pH
7 Mean_annual_temperatur
8 Precipitation_seasonality
9 Electrical_conductivity
10 Water_content
Precipitation_in_sample_m

Partial plots for
Fungal hemicellulose degr.

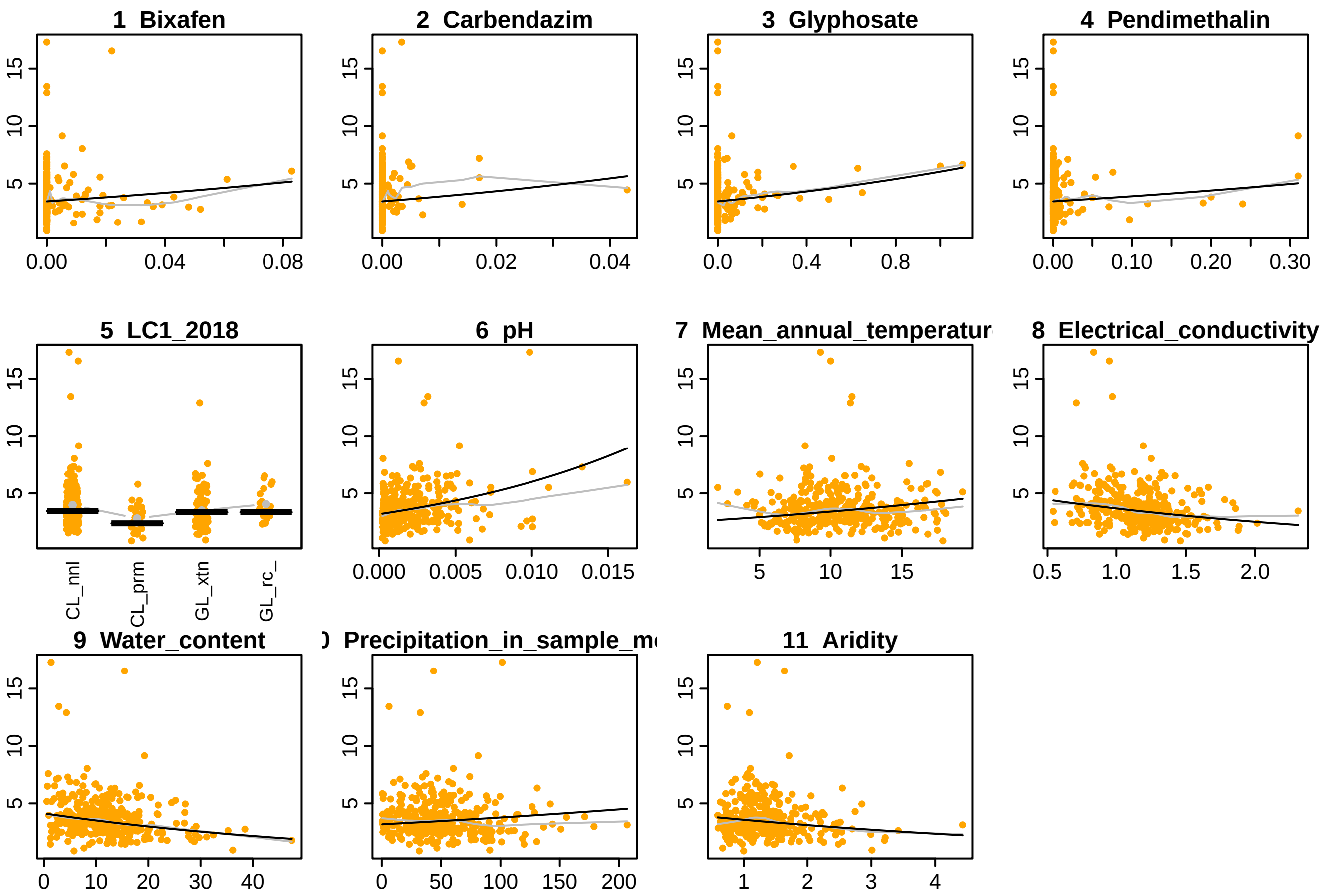

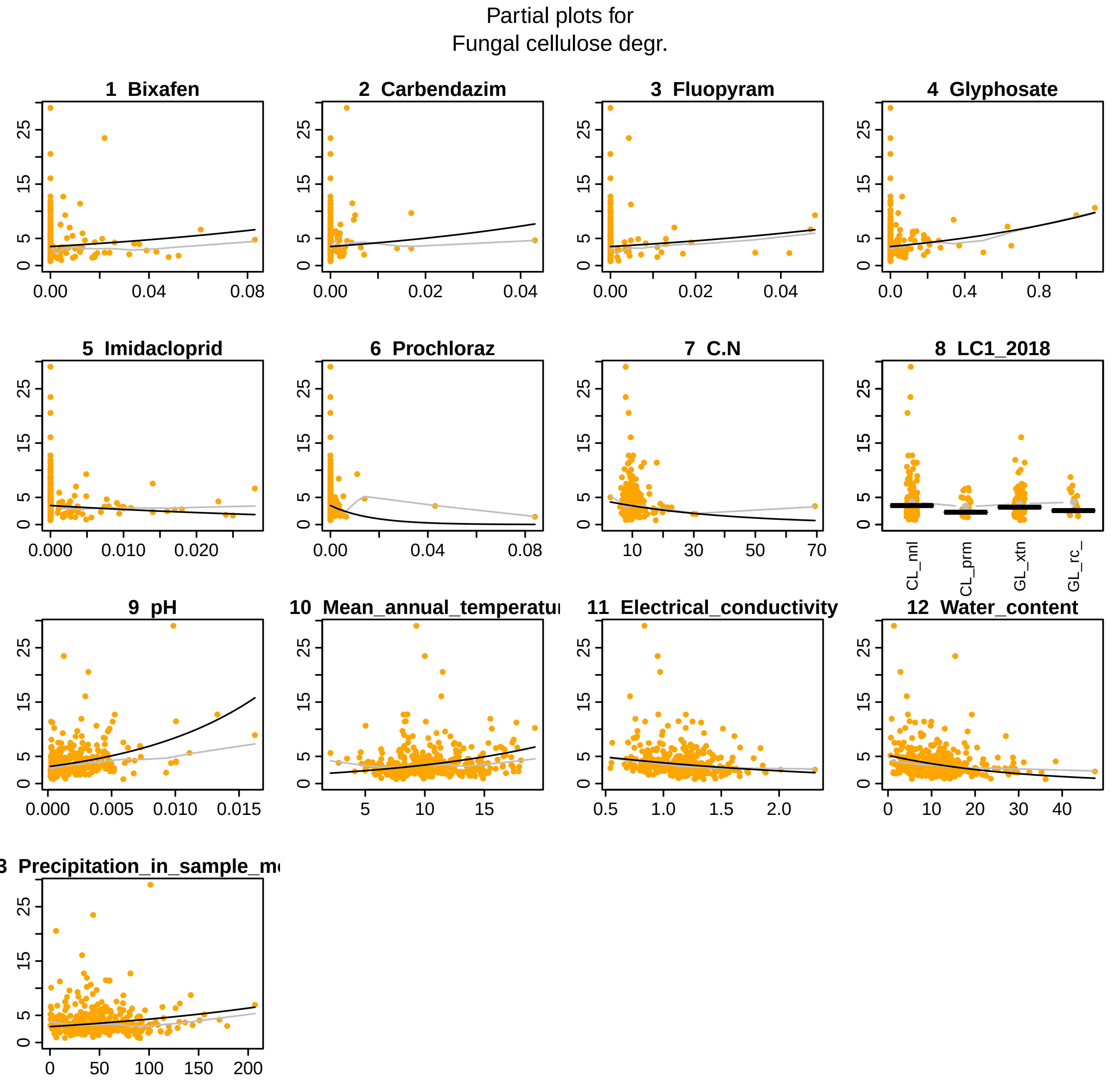
Partial plots for
Fungal cellulose degr.
1 Bixafen
2 Carbendazim
3 Fluopyram
4 Glyphosate
5 Imidacloprid
6 Prochloraz
7 C.N
8 LC1_2018
CL_nnl
CL_prm
GL_xtn
GL_rc_
9 pH
10 Mean_annual_temperatu
11 Electrical_conductivity
12 Water_content
3 Precipitation_in_sample_m

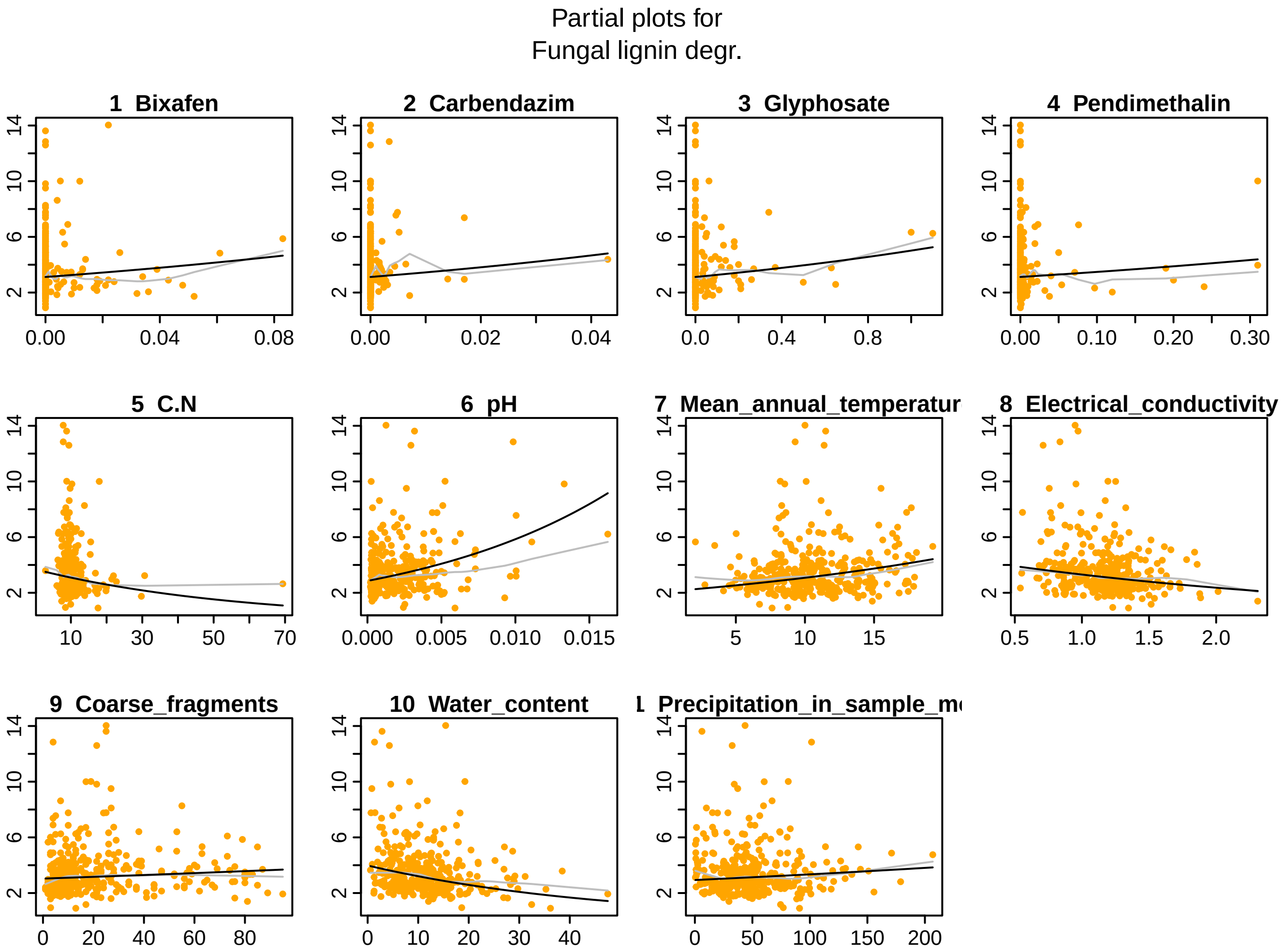

Partial plots for
Fungal lignin degr.
1 Bixafen
2 Carbendazim
3 Glyphosate
4 Pendimethalin
5 C.N
6 pH
7 Mean_annual_temperatur
8 Electrical_conductivity
9 Coarse_fragments
10 Water_content
Precipitation_in_sample_m

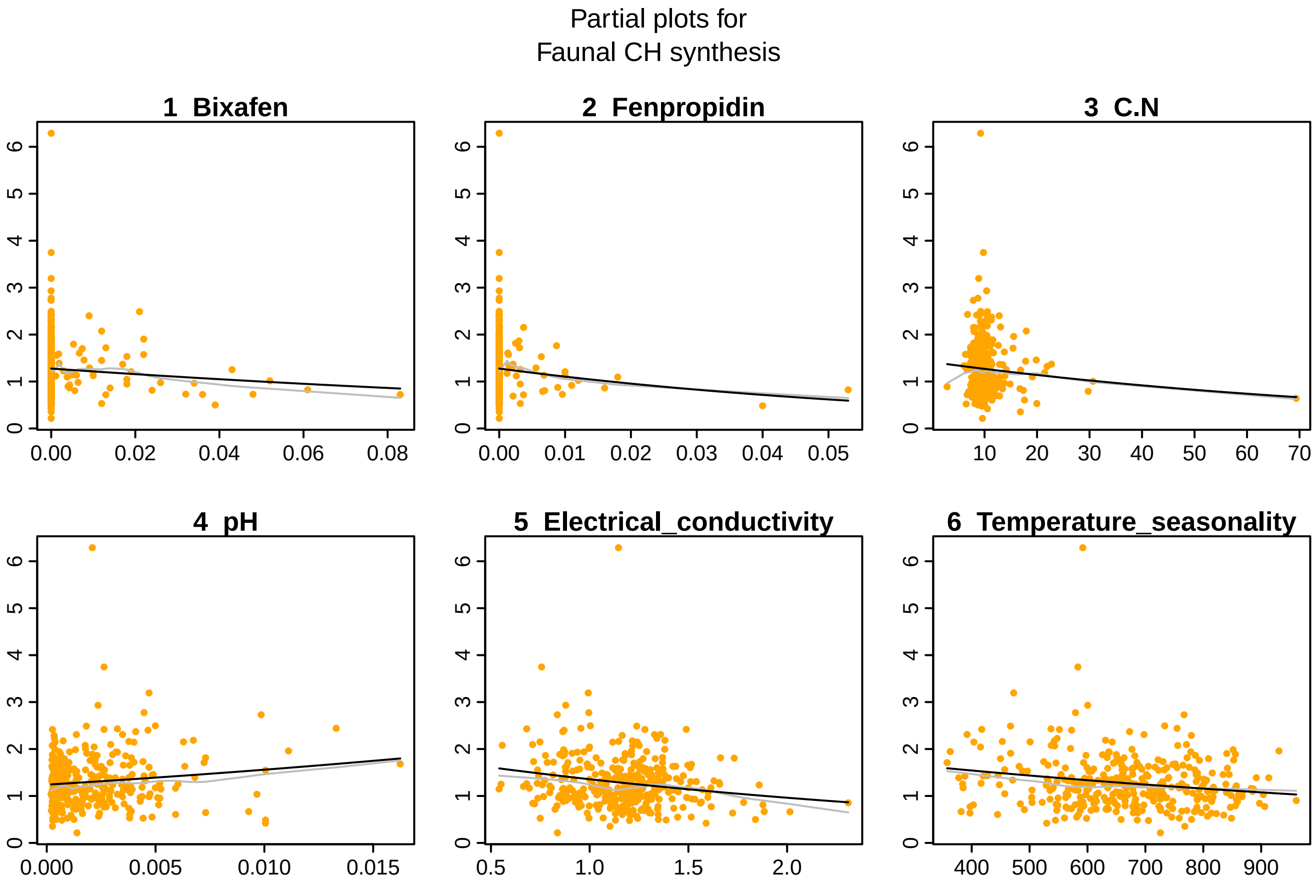

Partial plots for
Faunal CH synthesis
1 Bixafen
2 Fenpropidin
3 C.N
4 pH
5 Electrical_conductivity
6 Temperature_seasonality

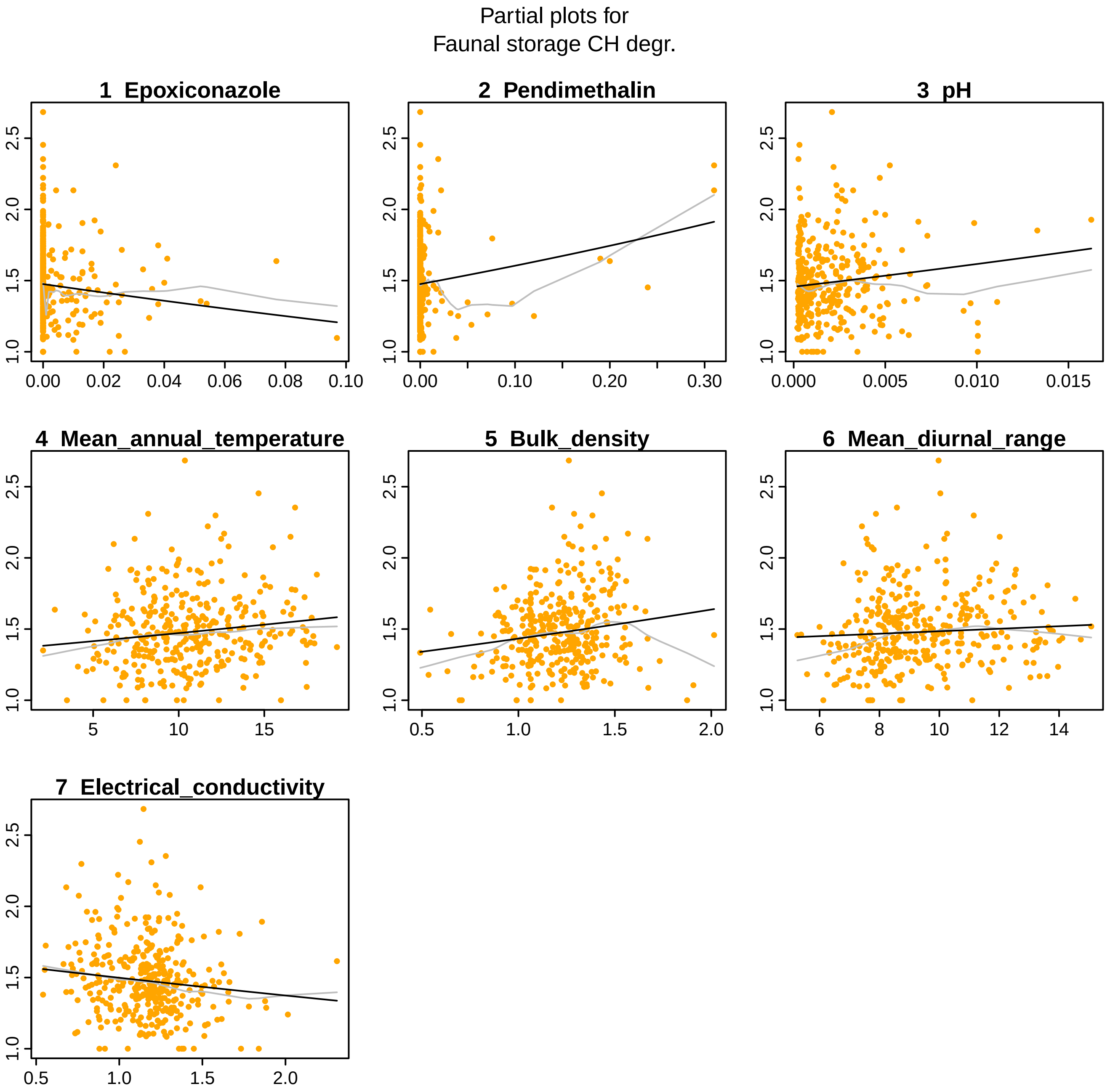

Partial plots for
Faunal storage CH degr.
1 Epoxiconazole
2 Pendimethalin
3 pH
4 Mean_annual_temperature
5 Bulk_density
6 Mean_diurnal_range
7 Electrical_conductivity

Partial plots for
Faunal hemicellulose degr.

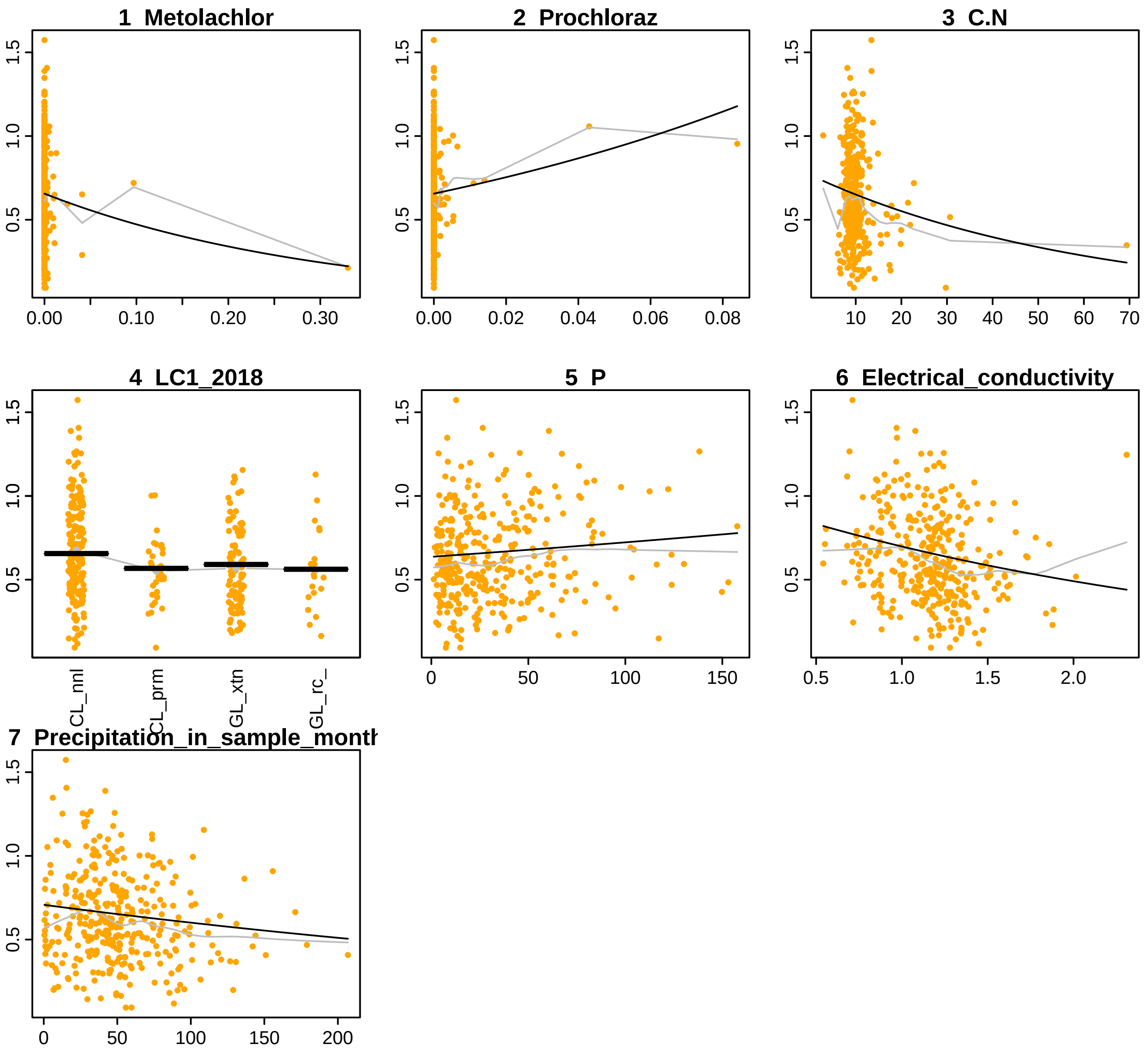

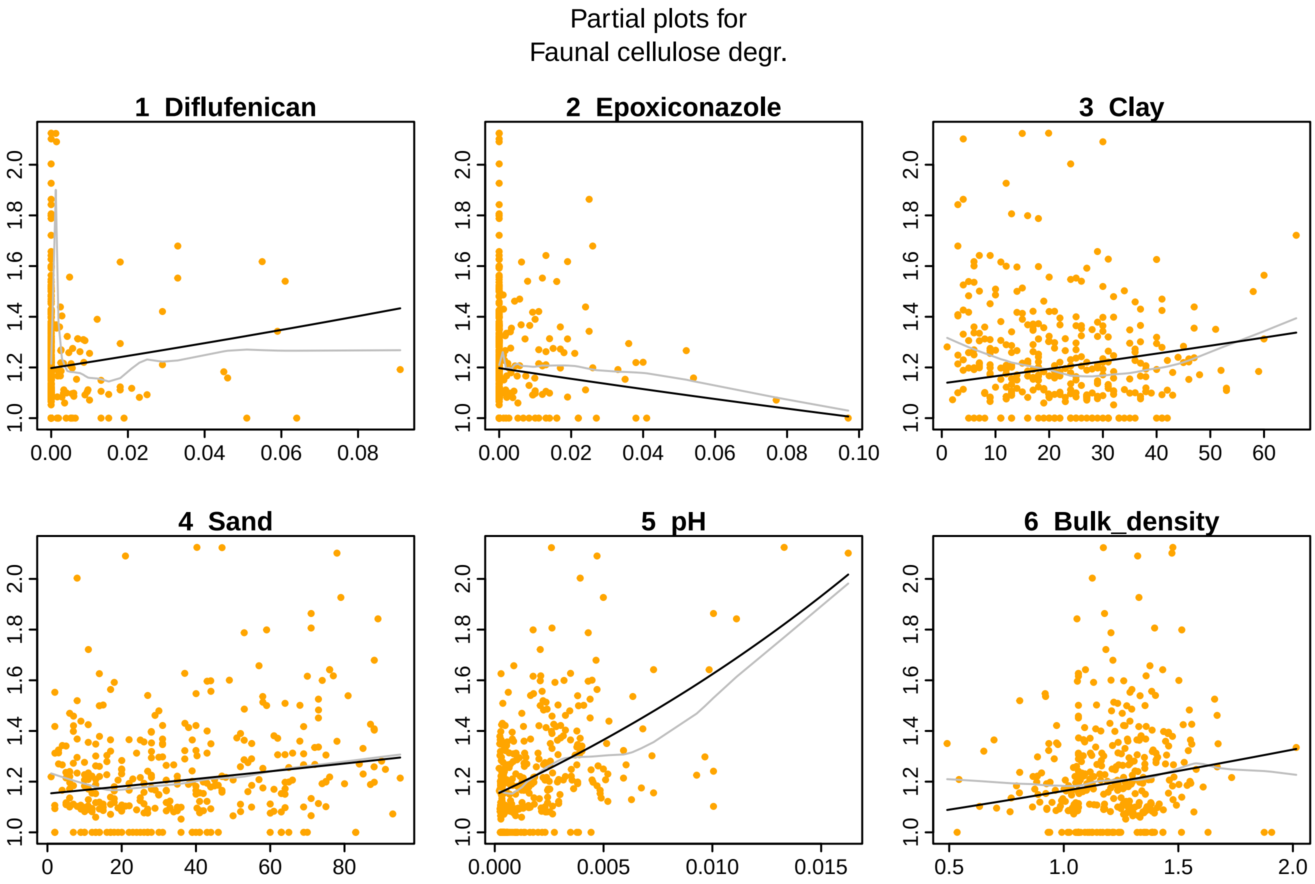

Partial plots for
Faunal cellulose degr.
1 Diflufenican
2 Epoxiconazole
3 Clay
4 Sand
5 pH
6 Bulk_density
1.0
1.2
1.4
1.6
1.8
2.0
0.00
0.02
0.04
0.06
0.08
0.10
0
10
20
30
40
50
60
80
0.000
0.005
0.010
0.015
0.5
1.0
1.5
2.0

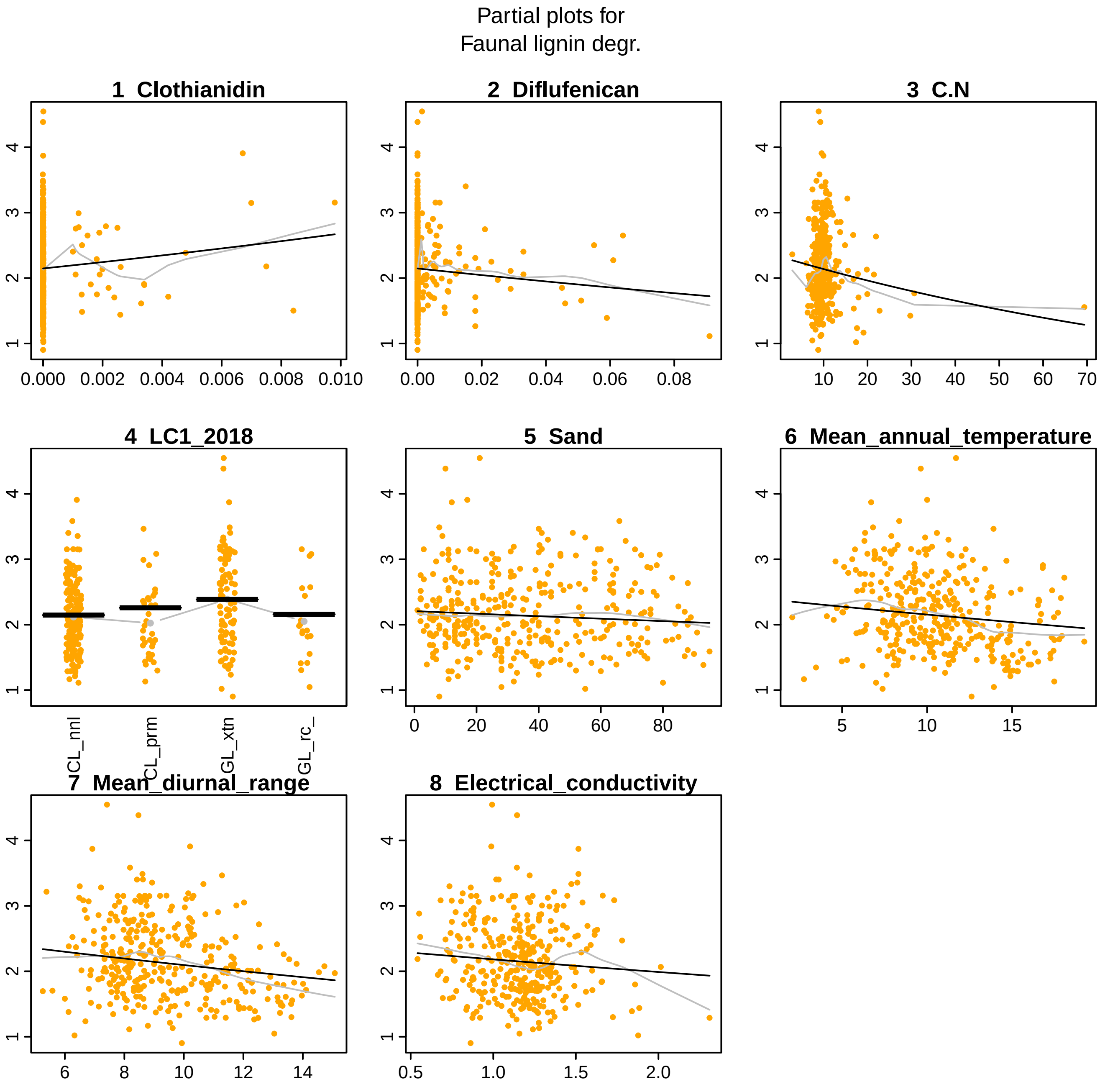

Partial plots for
Faunal lignin degr.
1 Clothianidin
2 Diflufenican
3 C.N
4 LC1_2018
5 Sand
6 Mean_annual_temperature
7 Mean_diurnal_range
8 Electrical_conductivity
CL_nnl
CL_prm
GL_xtn
GL_rc_

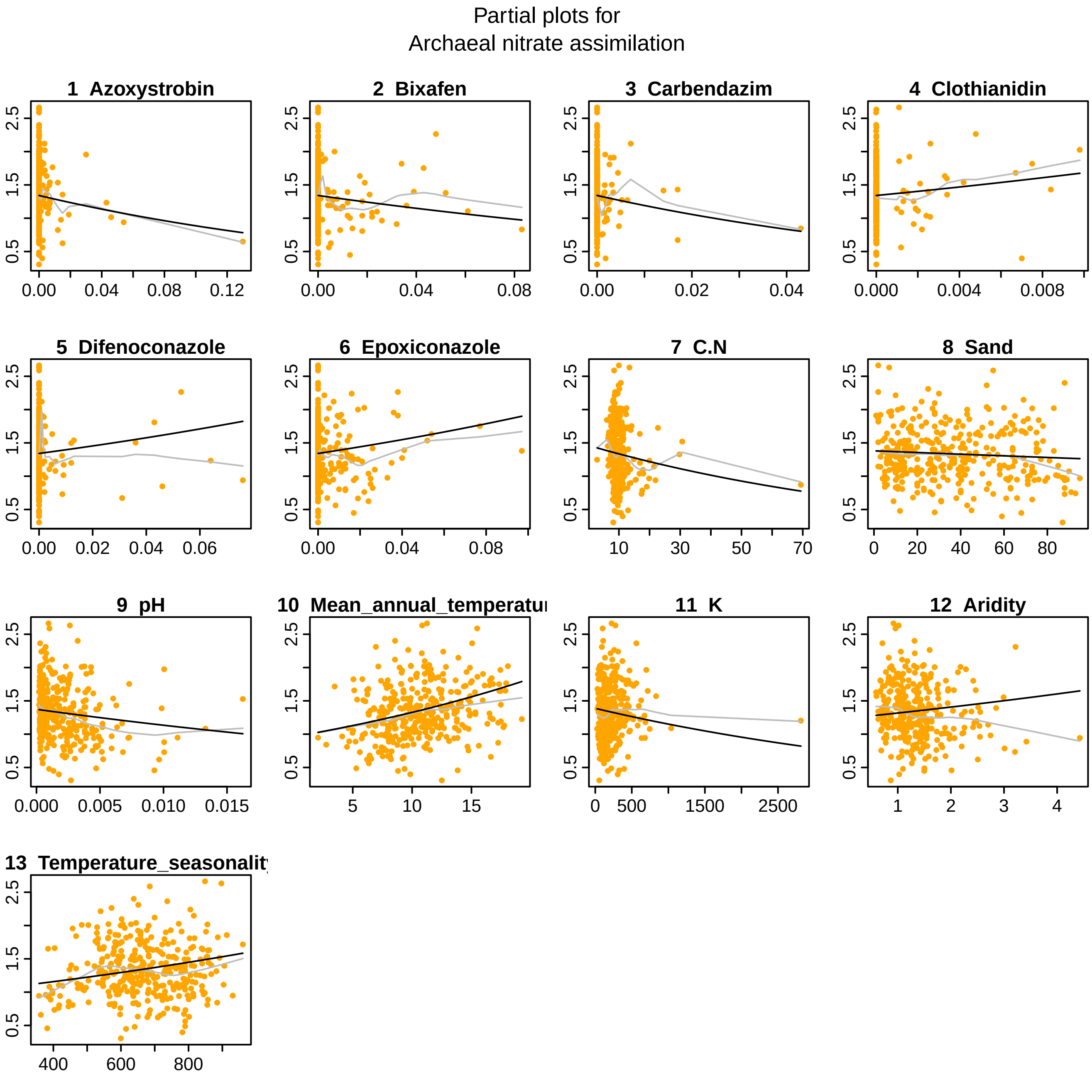

Partial plots for
Archaeal nitrate assimilation
1 Azoxystrobin
2 Bixafen
3 Carbendazim
4 Clothianidin
5 Difenoconazole
6 Epoxiconazole
7 C.N
8 Sand
9 pH
10 Mean_annual_temperatu
11 K
12 Aridity
13 Temperature_seasonalit

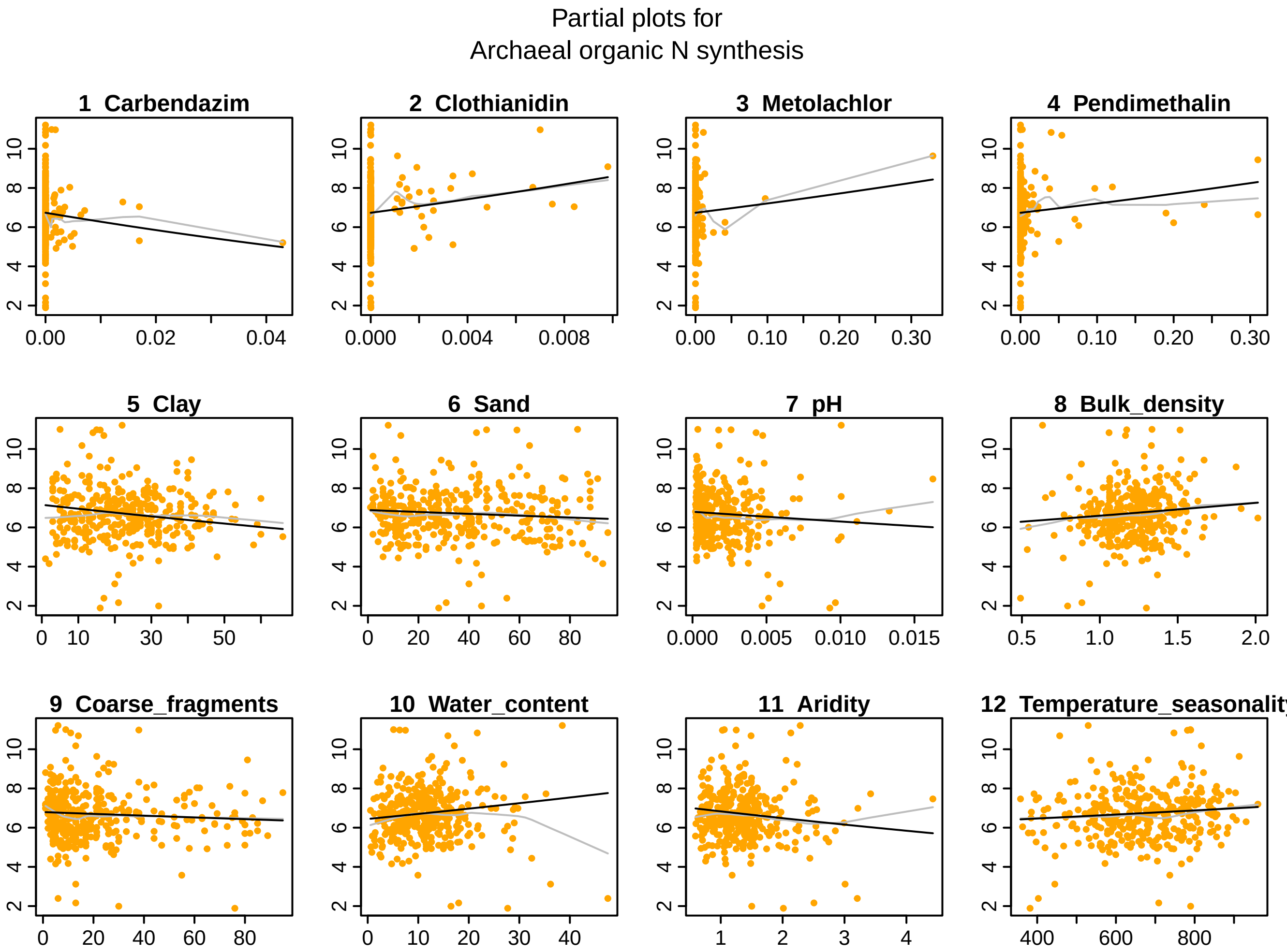
Partial plots for
Archaeal organic N synthesis
1 Carbendazim
2 Clothianidin
3 Metolachlor
4 Pendimethalin
5 Clay
6 Sand
7 pH
8 Bulk_density
9 Coarse_fragments
10 Water_content
11 Aridity
12 Temperature_seasonality

Partial plots for Archaeal organic N degr.

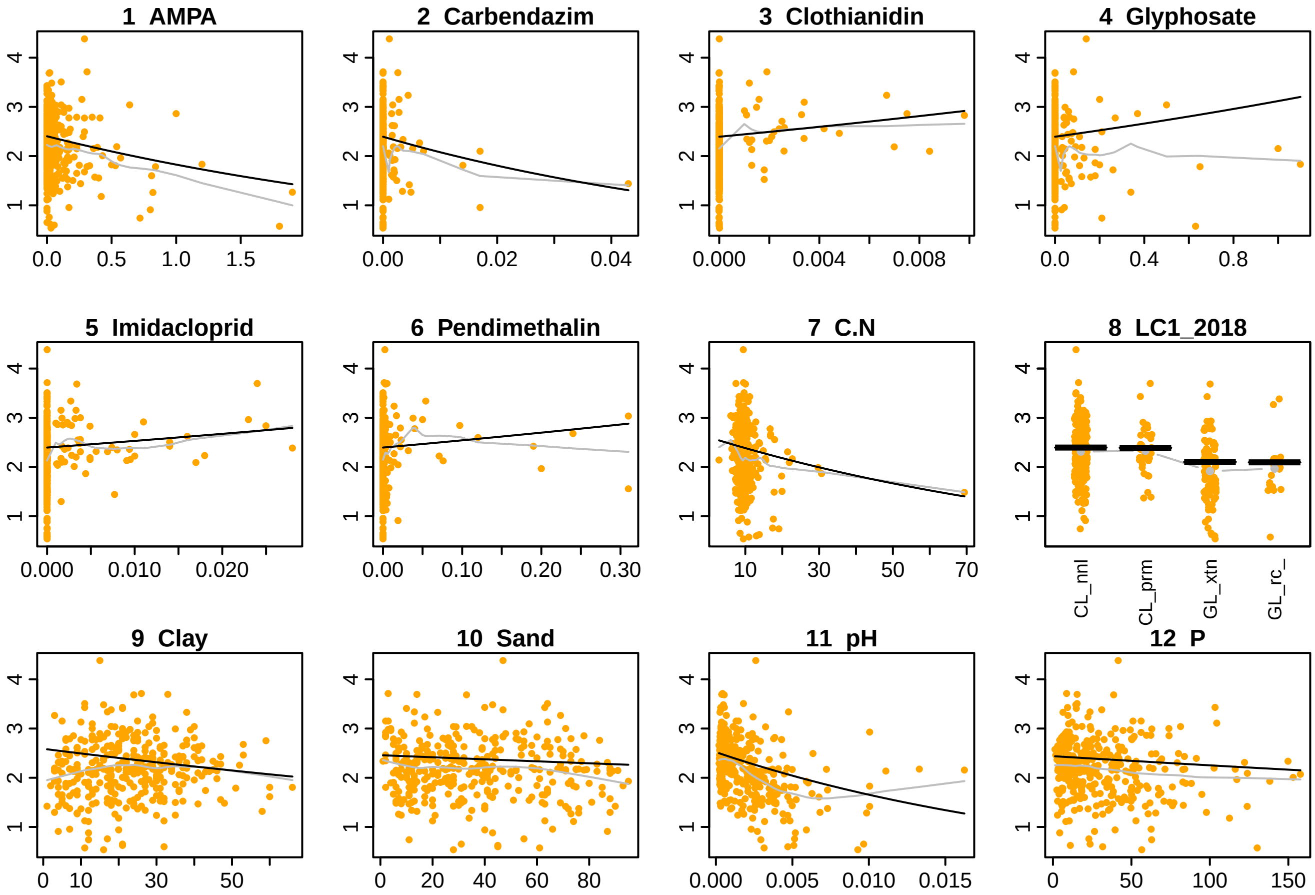

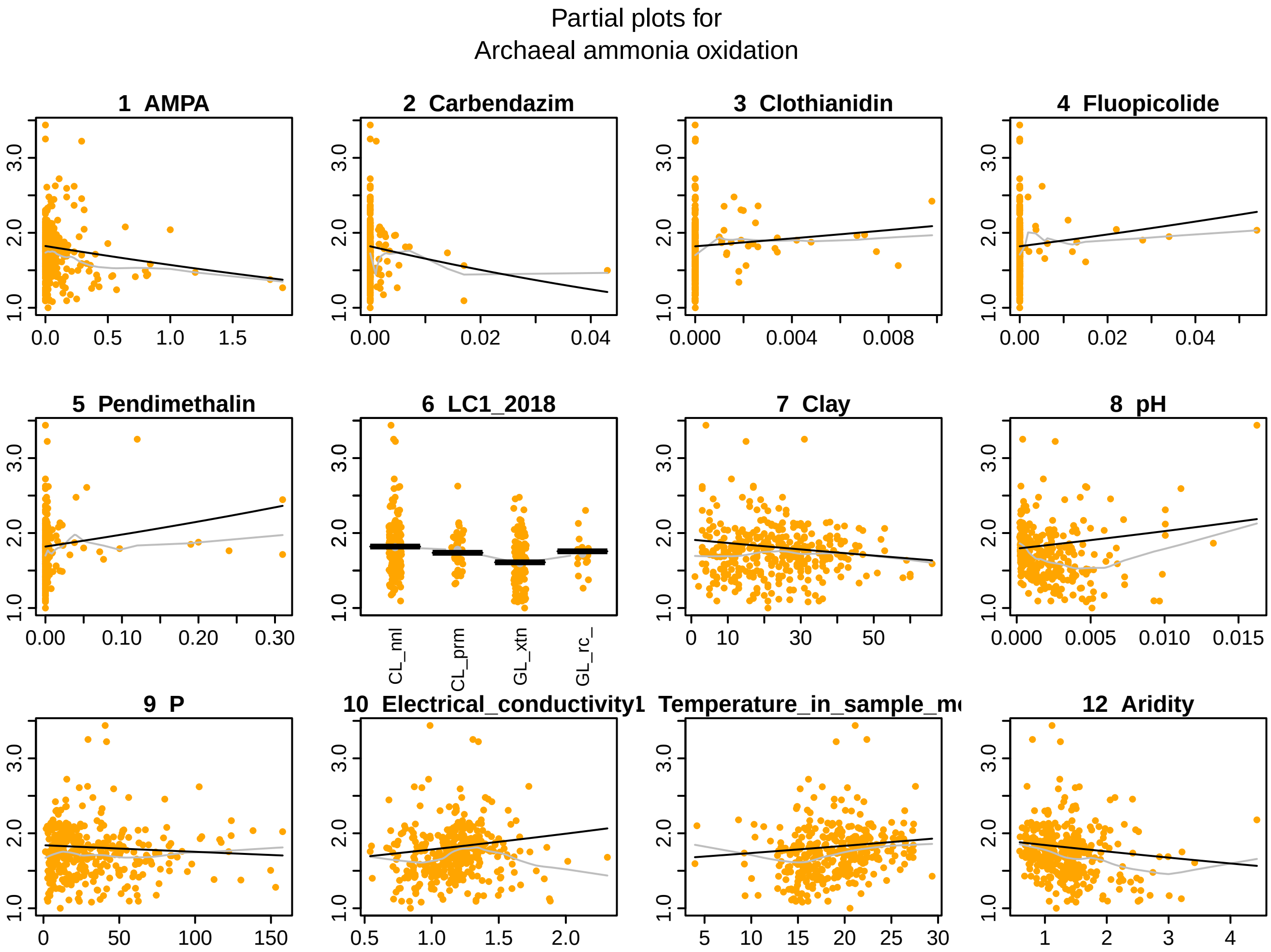

Partial plots for
Archaeal ammonia oxidation
1 AMPA
2 Carbendazim
3 Clothianidin
4 Fluopicolide
5 Pendimethalin
6 LC1_2018
7 Clay
8 pH
9 P
10 Electrical_conductivity1
Temperature_in_sample_mo
12 Aridity
CL_nnl
CL_prm
GL_xtn
GL_rc_

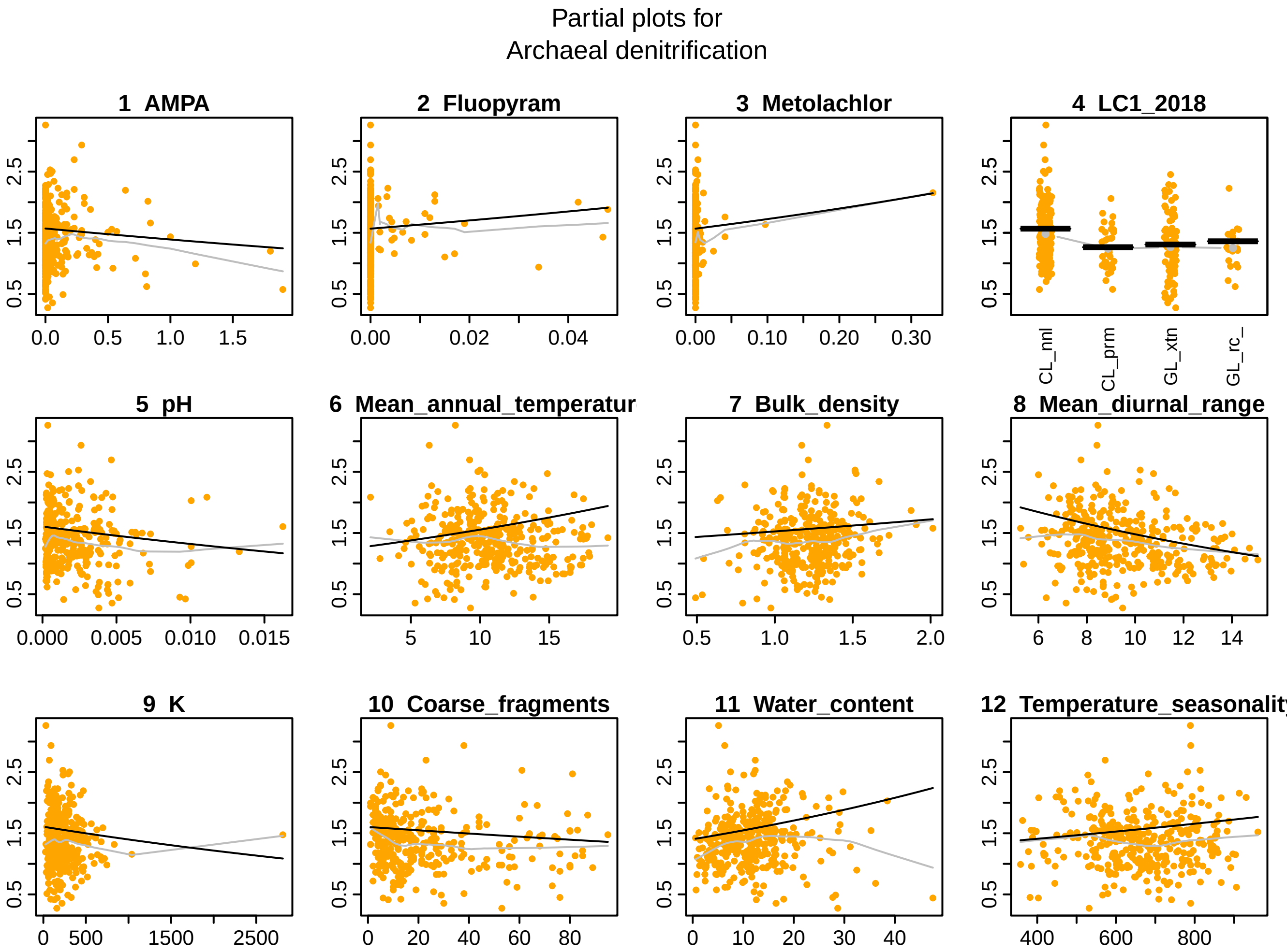
Partial plots for
Archaeal denitrification
1 AMPA
2 Fluopyram
3 Metolachlor
4 LC1_2018
CL_nnl
CL_prm
GL_xtn
GL_rc_
5 pH
6 Mean_annual_temperatur
7 Bulk_density
8 Mean_diurnal_range
9 K
10 Coarse_fragments
11 Water_content
12 Temperature_seasonalit

Partial plots for
Archaeal DNRA

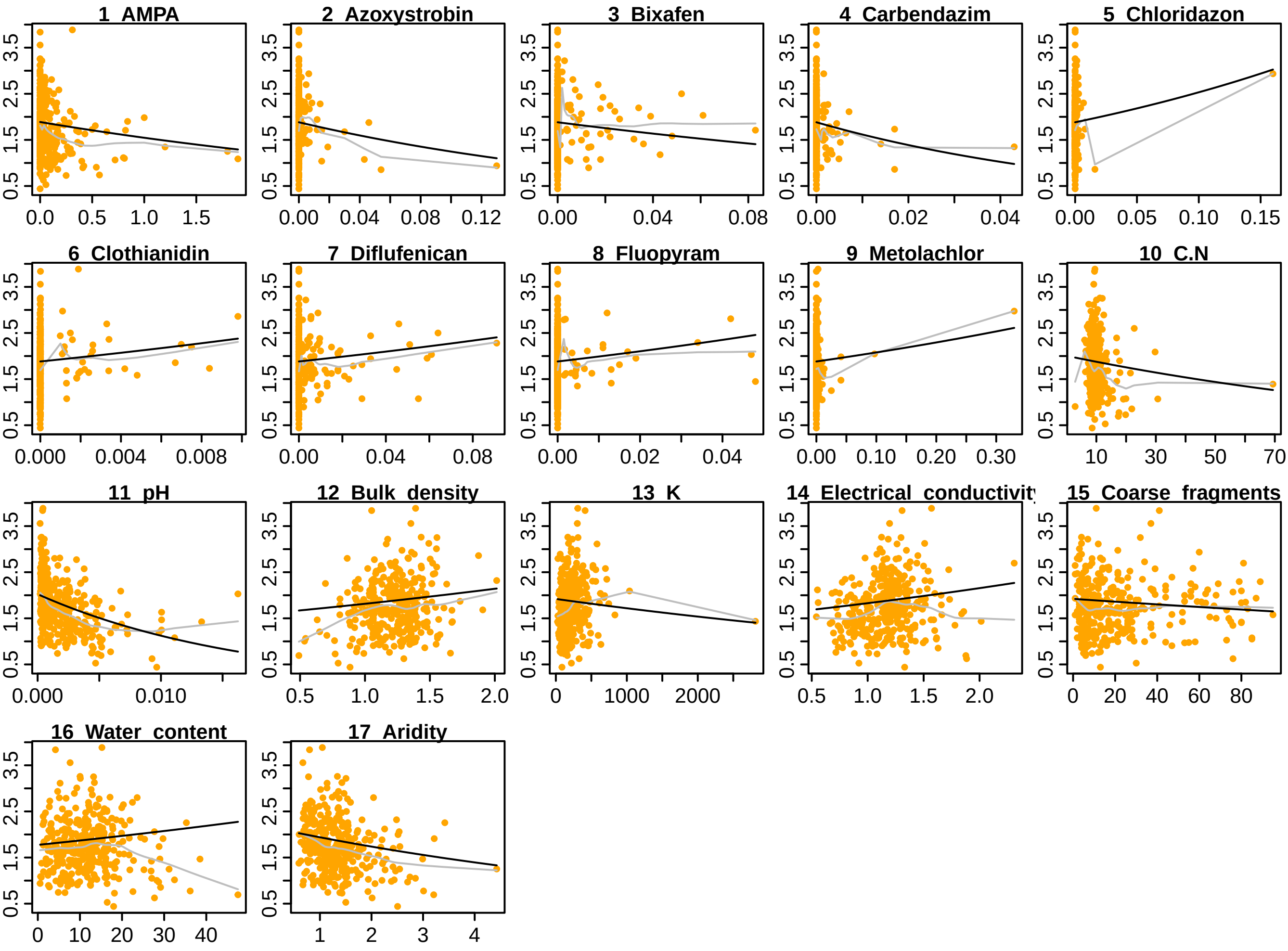

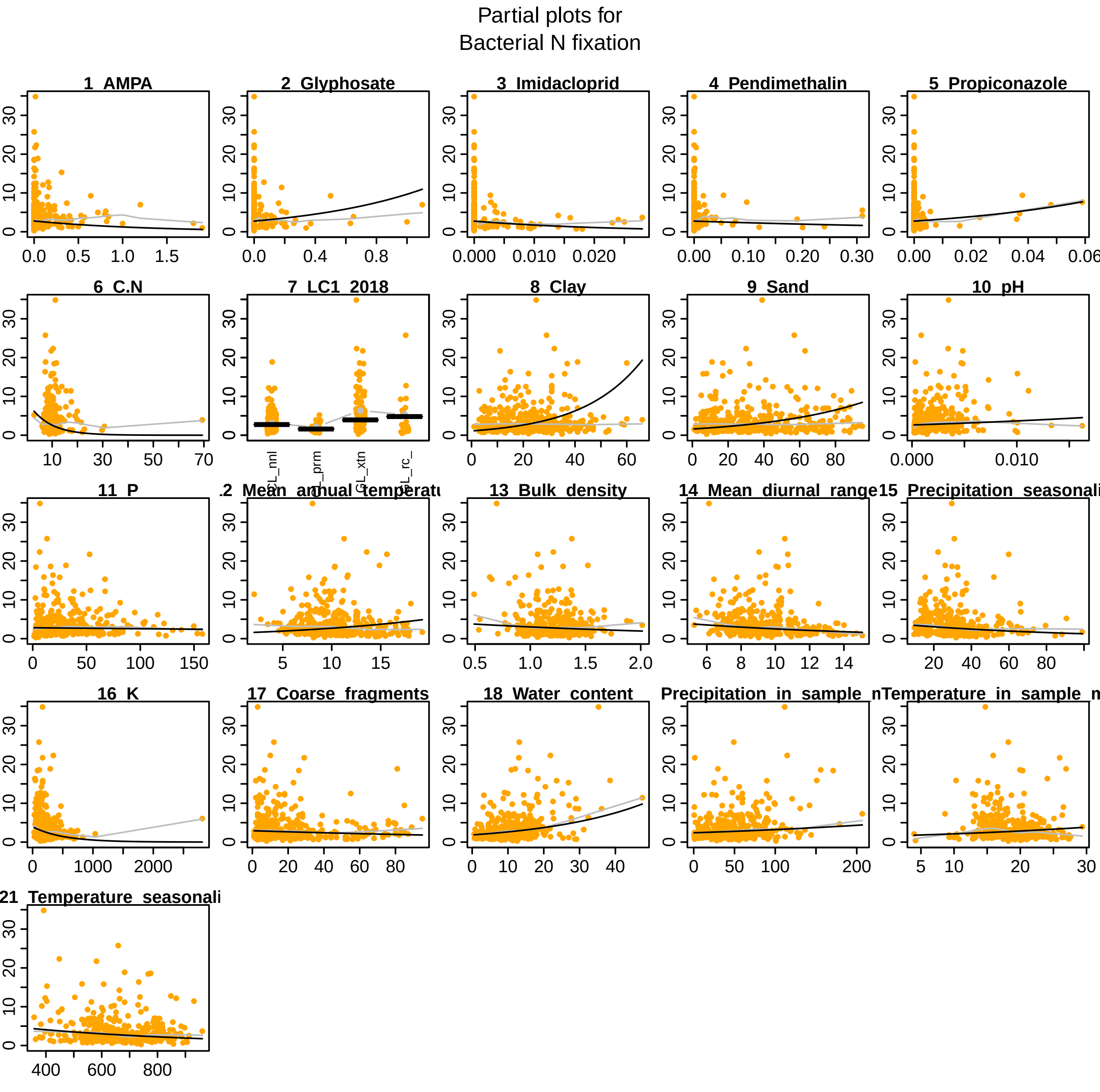
Partial plots for
Bacterial N fixation
1 AMPA
2 Glyphosate
3 Imidacloprid
4 Pendimethalin
5 Propiconazole
6 C.N
7 LC1 2018
8 Clay
9 Sand
10 pH
11 P
.2 Mean annual temperatu
13 Bulk density
14 Mean diurnal range
15 Precipitation seasonali
16 K
17 Coarse fragments
18 Water content
Precipitation in sample n
Temperature in sample m
21 Temperature seasonali

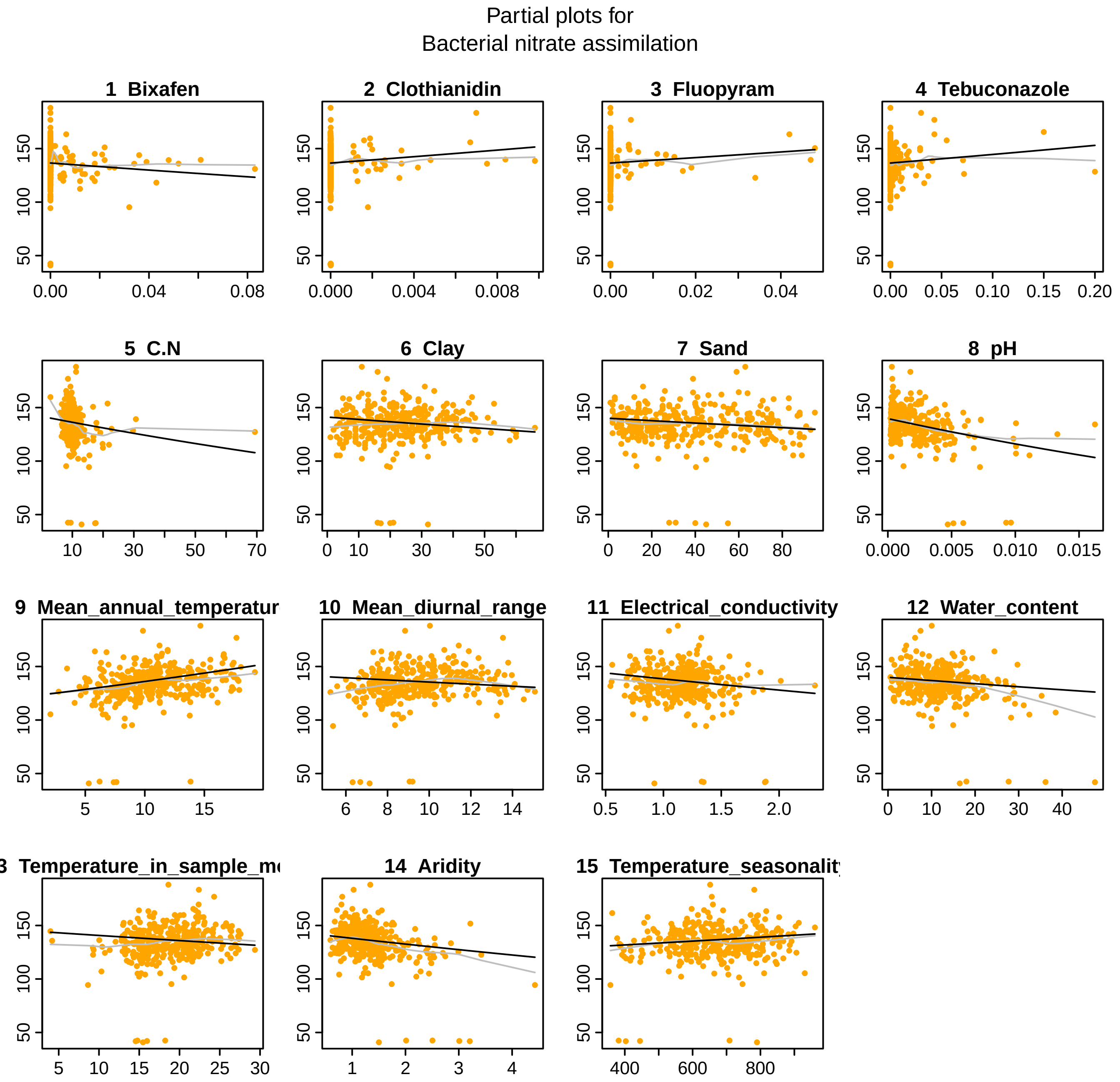
Partial plots for
Bacterial nitrate assimilation
1 Bixafen
2 Clothianidin
3 Fluopyram
4 Tebuconazole
5 C.N
6 Clay
7 Sand
8 pH
9 Mean_annual_temperatur
10 Mean_diurnal_range
11 Electrical_conductivity
12 Water_content
3 Temperature_in_sample_mo
14 Aridity
15 Temperature_seasonality

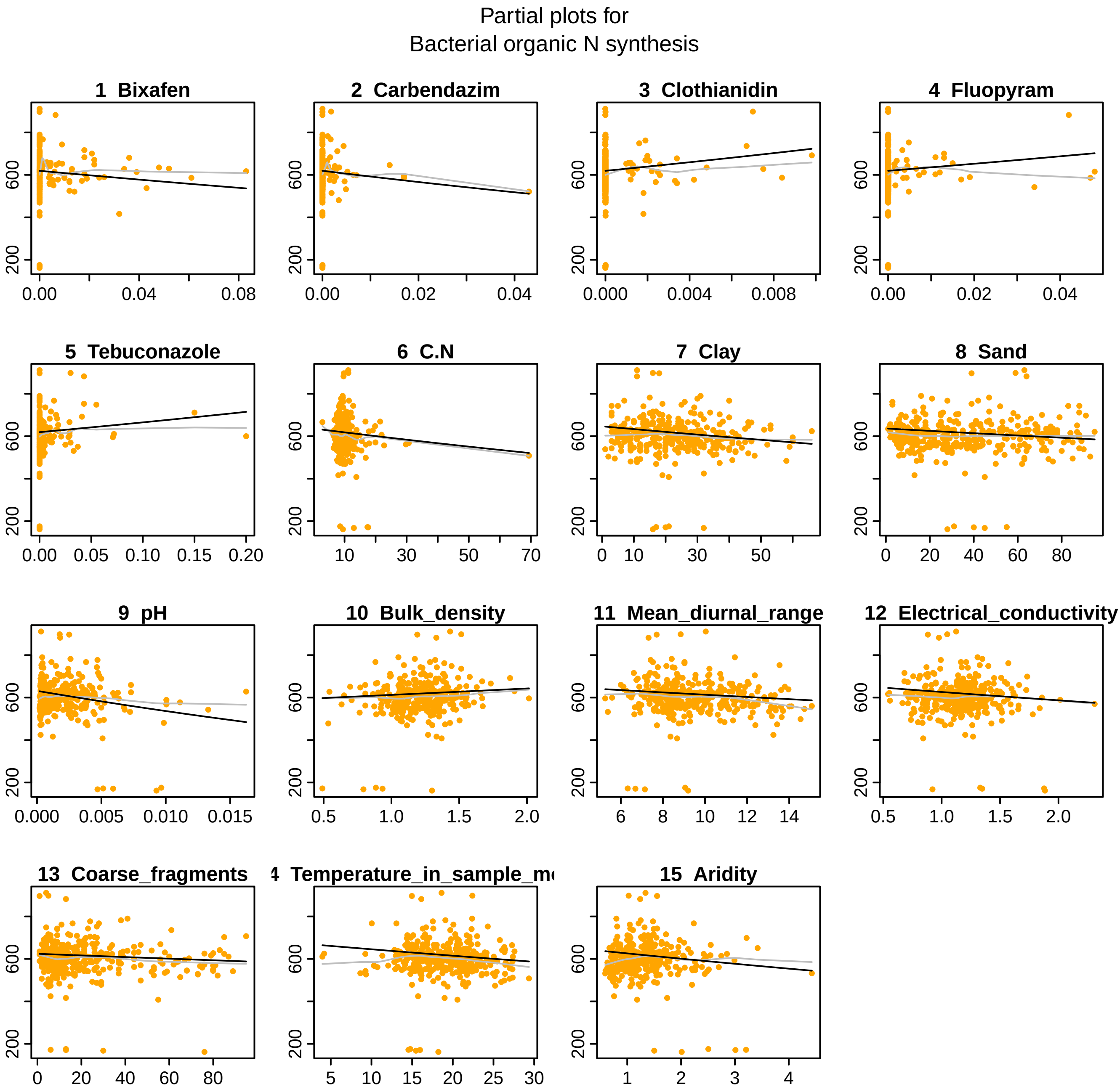
Partial plots for
Bacterial organic N synthesis
1 Bixafen
2 Carbendazim
3 Clothianidin
4 Fluopyram
5 Tebuconazole
6 C.N
7 Clay
8 Sand
9 pH
10 Bulk_density
11 Mean_diurnal_range
12 Electrical_conductivity
13 Coarse_fragments
4 Temperature_in_sample_m
15 Aridity

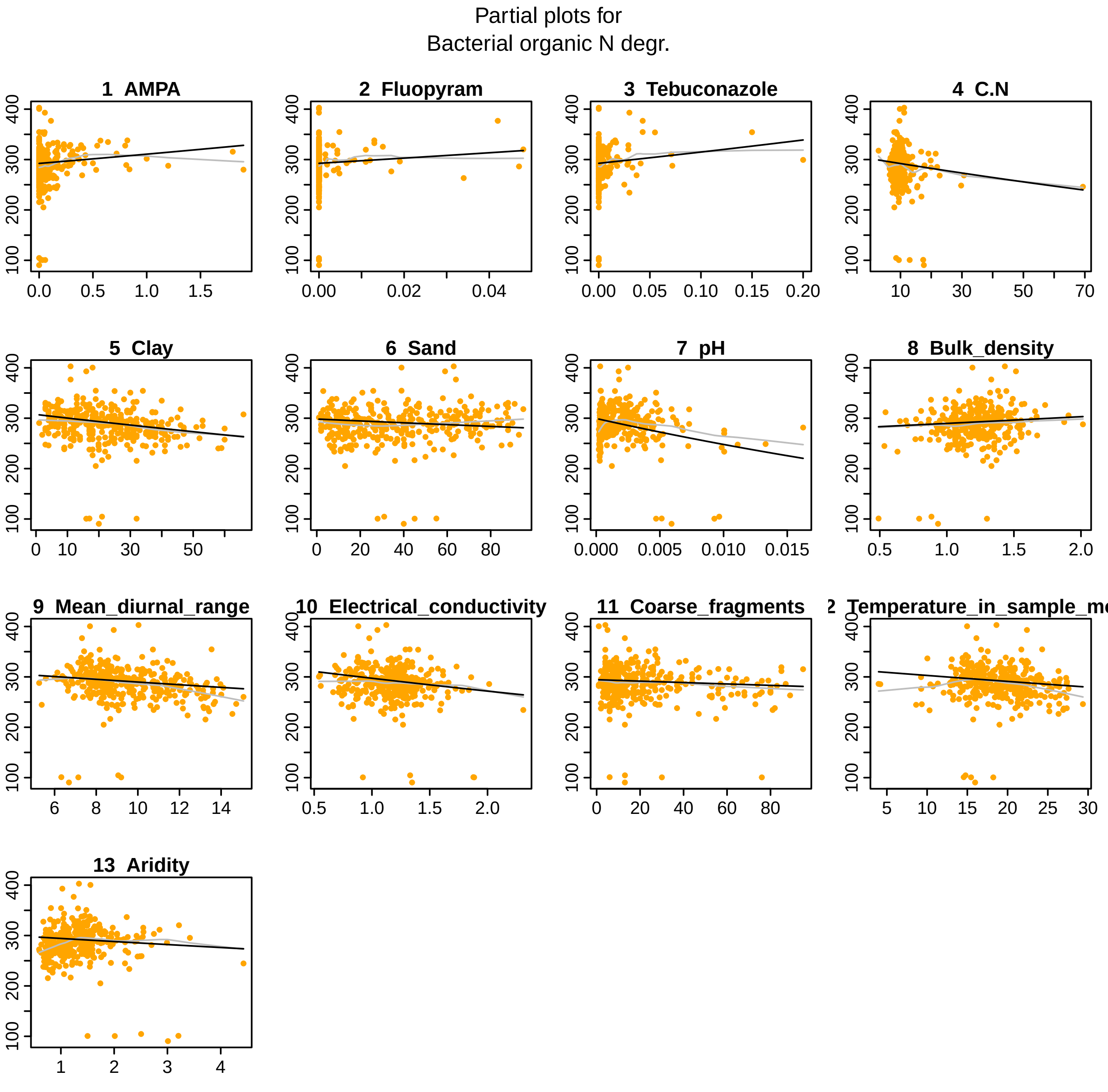
Partial plots for
Bacterial organic N degr.
1 AMPA
2 Fluopyram
3 Tebuconazole
4 C.N
5 Clay
6 Sand
7 pH
8 Bulk_density
9 Mean_diurnal_range
10 Electrical_conductivity
11 Coarse_fragments
2 Temperature_in_sample_mo
13 Aridity

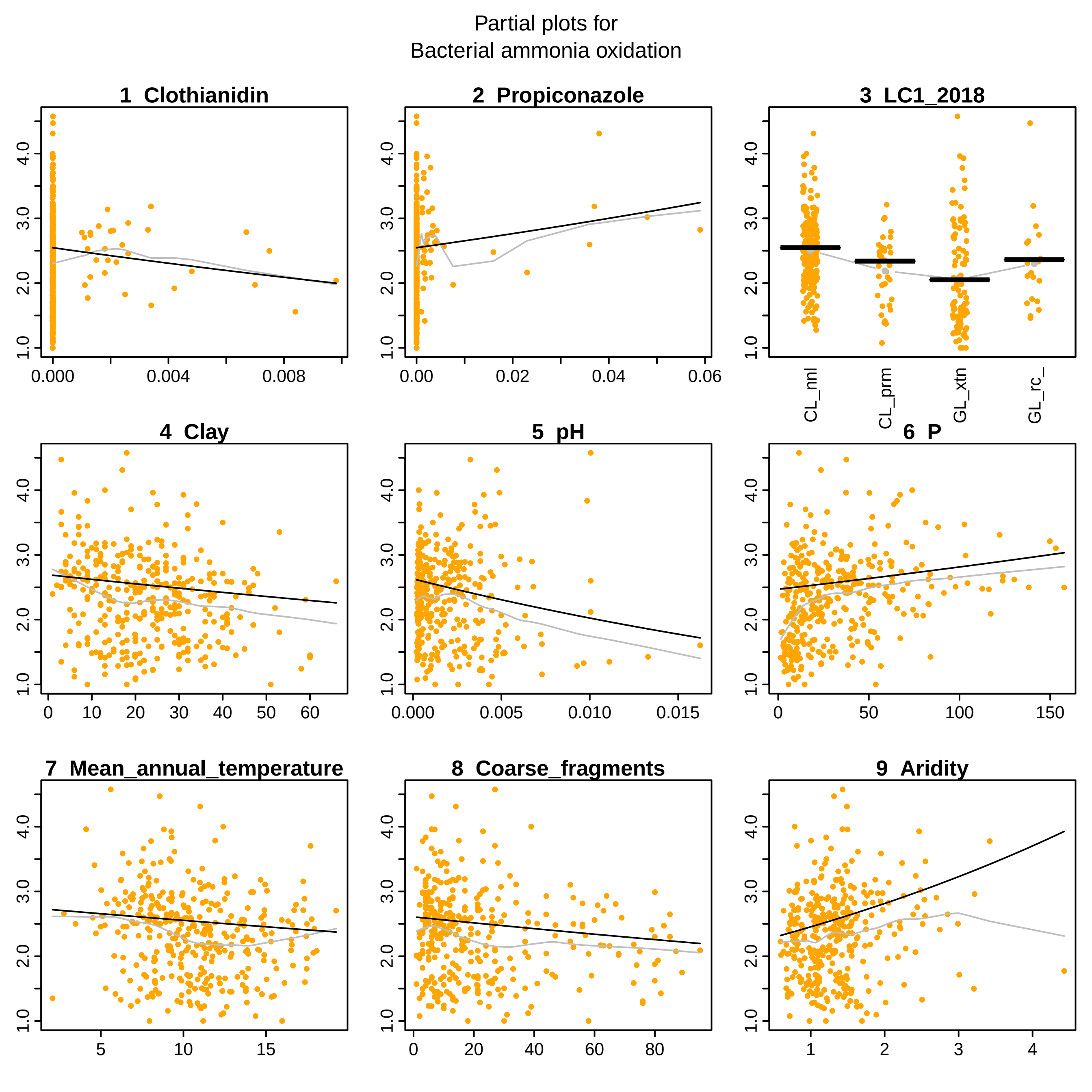

Partial plots for
Bacterial ammonia oxidation
1 Clothianidin
2 Propiconazole
3 LC1_2018
4 Clay
5 pH
6 P
7 Mean_annual_temperature
8 Coarse_fragments
9 Aridity
CL_nnl
CL_prm
GL_xtn
GL_rc_

Partial plots for
Bacterial nitratation

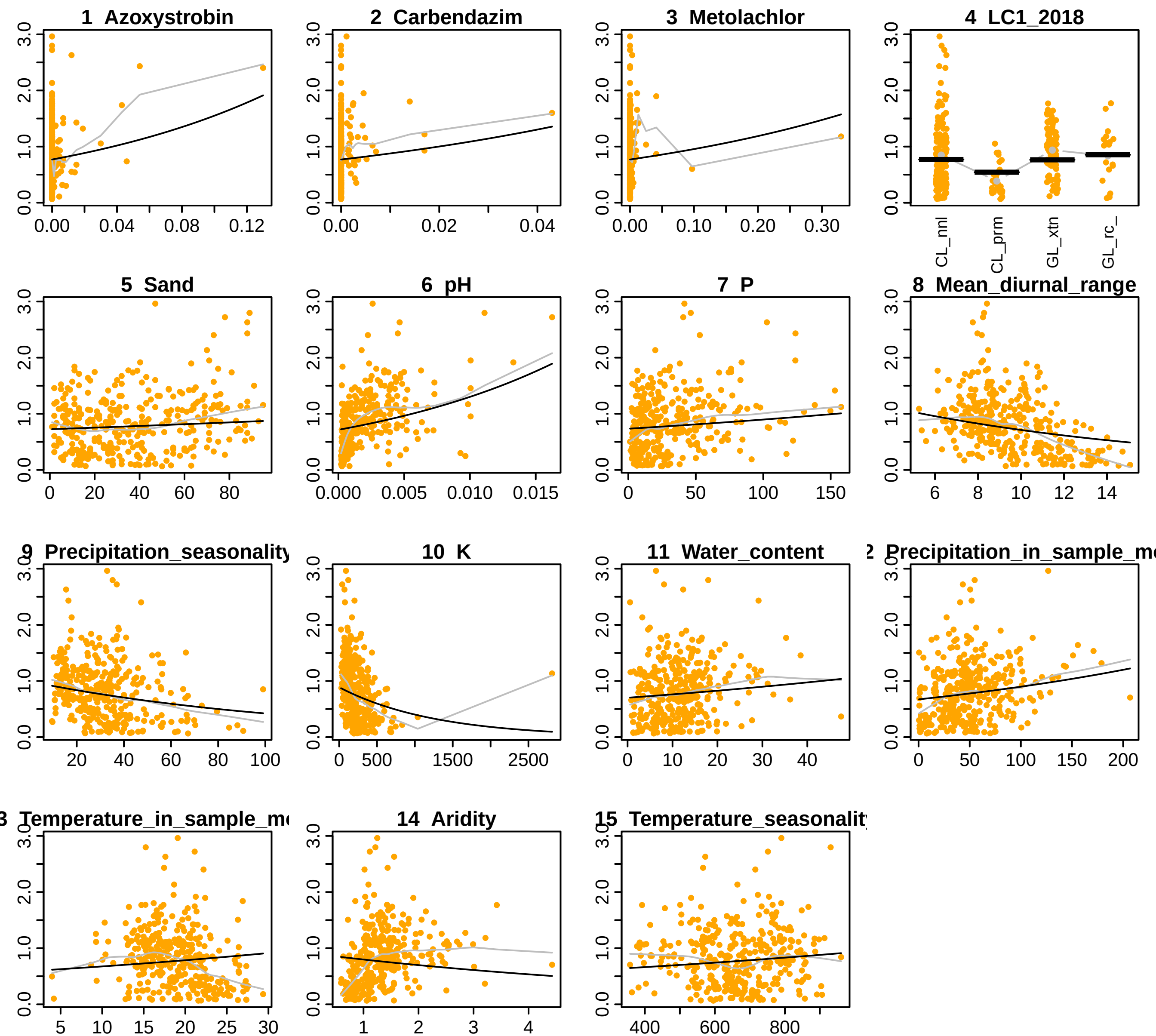

Partial plots for
Bacterial denitrification

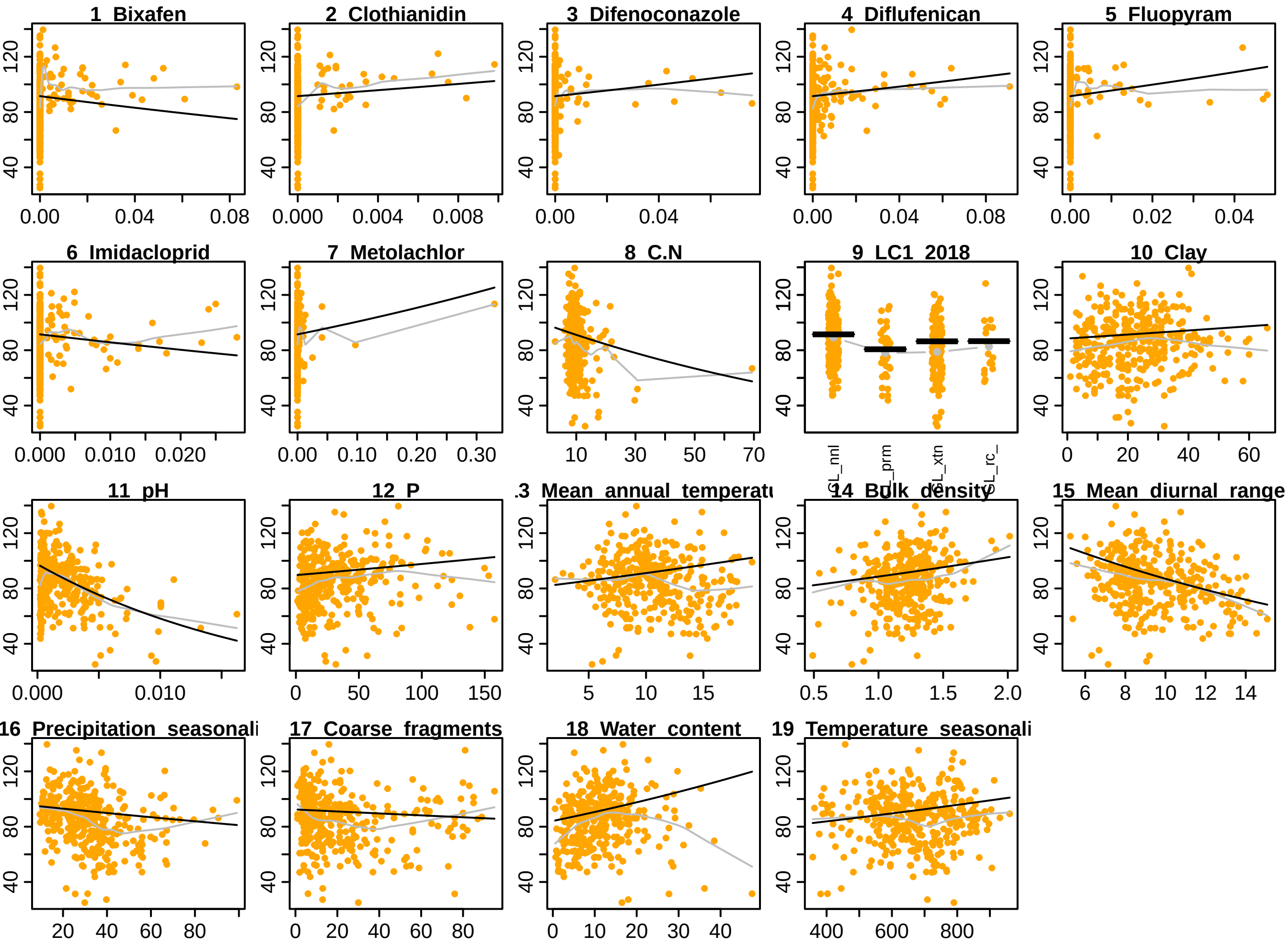

Partial plots for
Bacterial DNRA

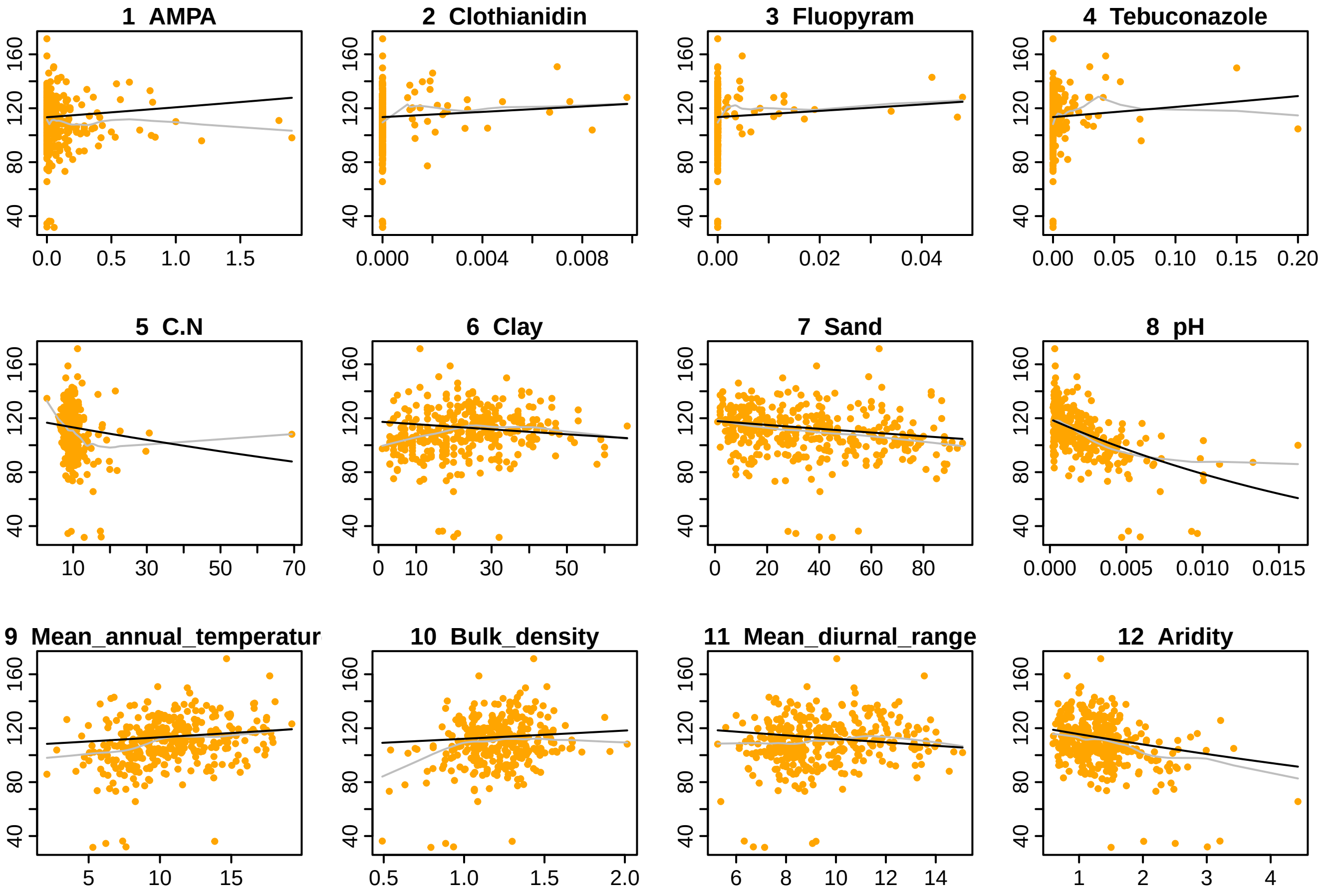

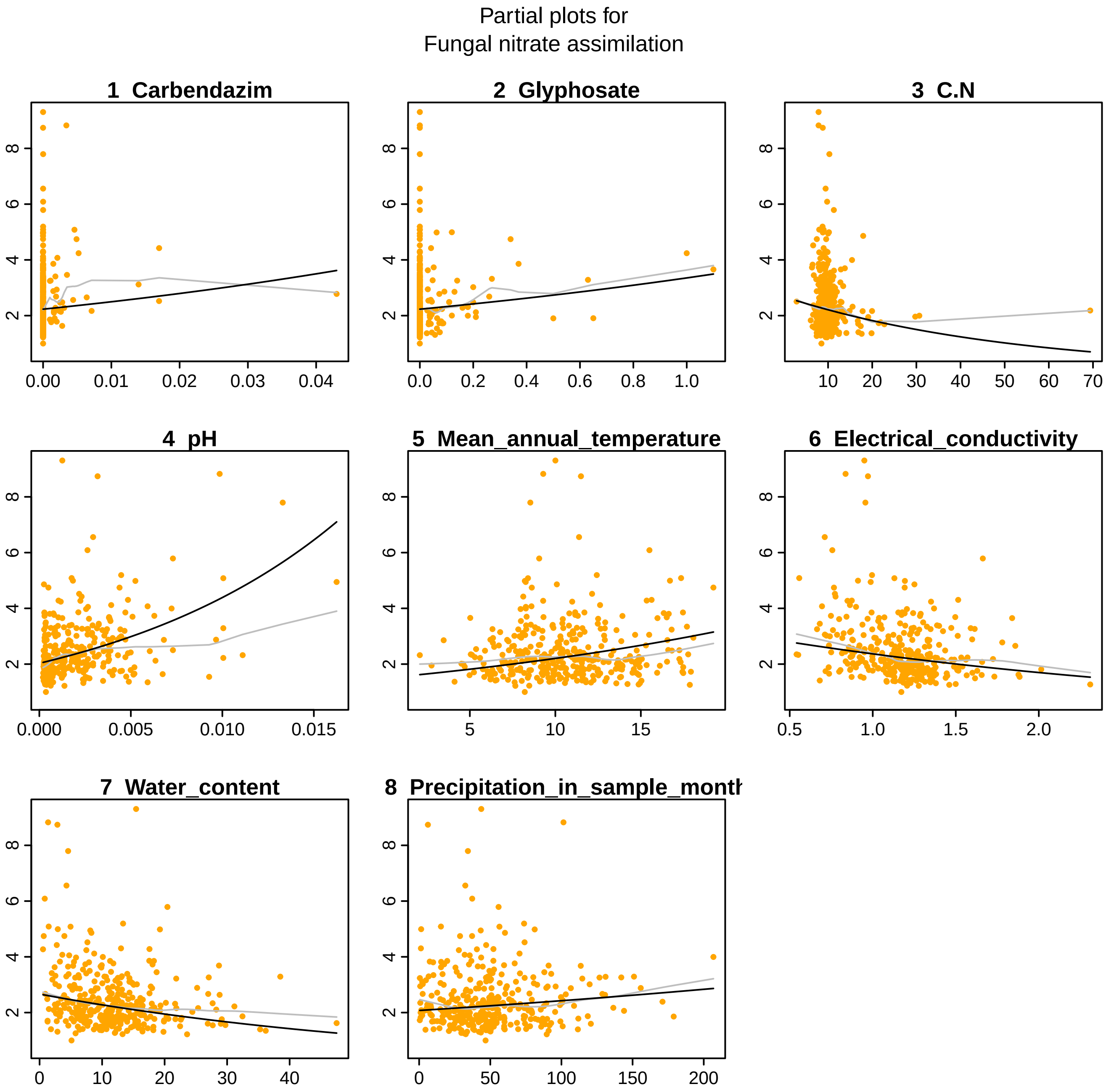

Partial plots for
Fungal nitrate assimilation
1 Carbendazim
2 Glyphosate
3 C.N
4 pH
5 Mean_annual_temperature
6 Electrical_conductivity
7 Water_content
8 Precipitation_in_sample_montl

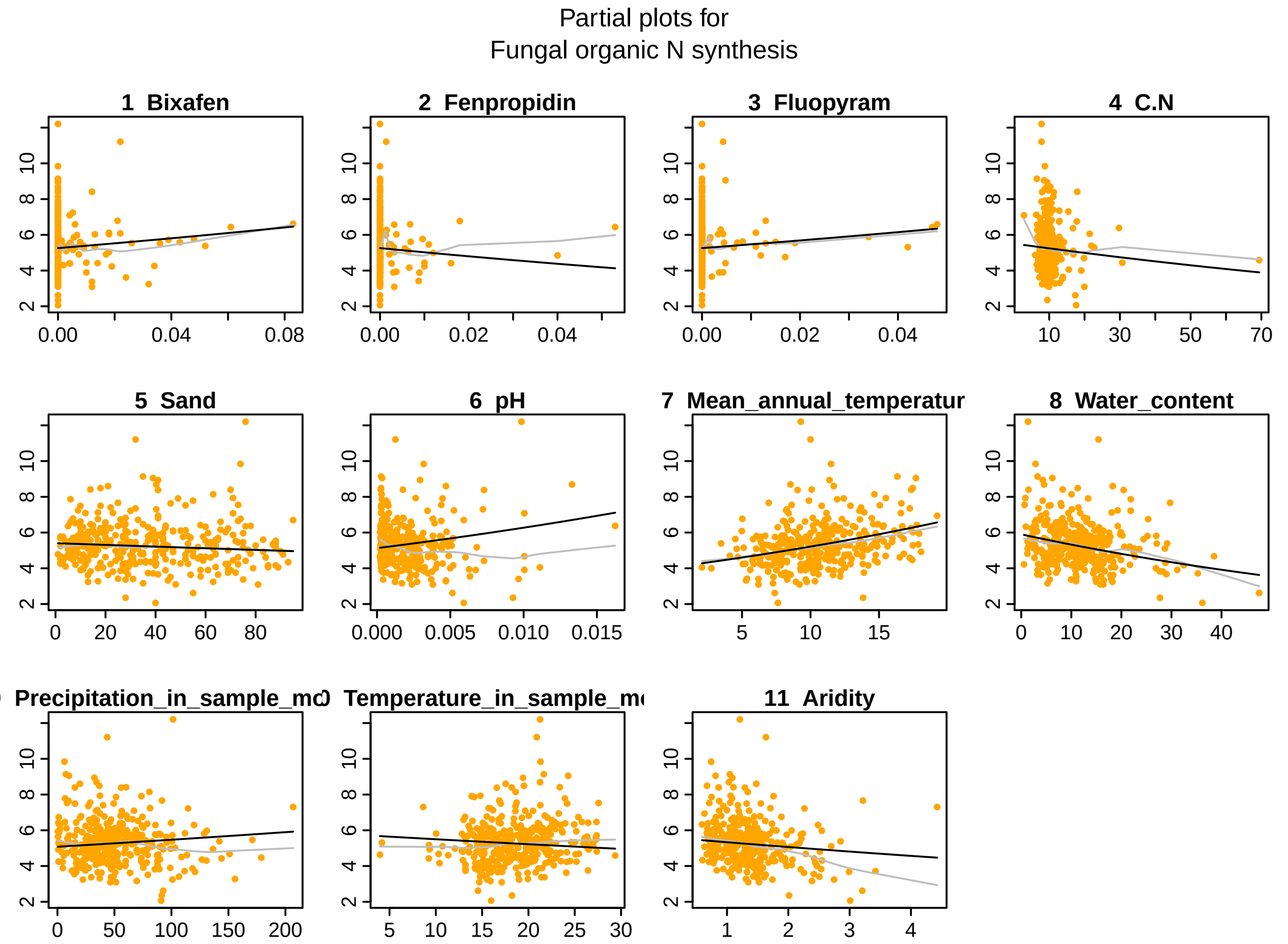
Partial plots for
Fungal organic N synthesis
1 Bixafen
2 Fenpropidin
3 Fluopyram
4 C.N
5 Sand
6 pH
7 Mean_annual_temperatur
8 Water_content
Precipitation_in_sample_mo
Temperature_in_sample_mo
11 Aridity

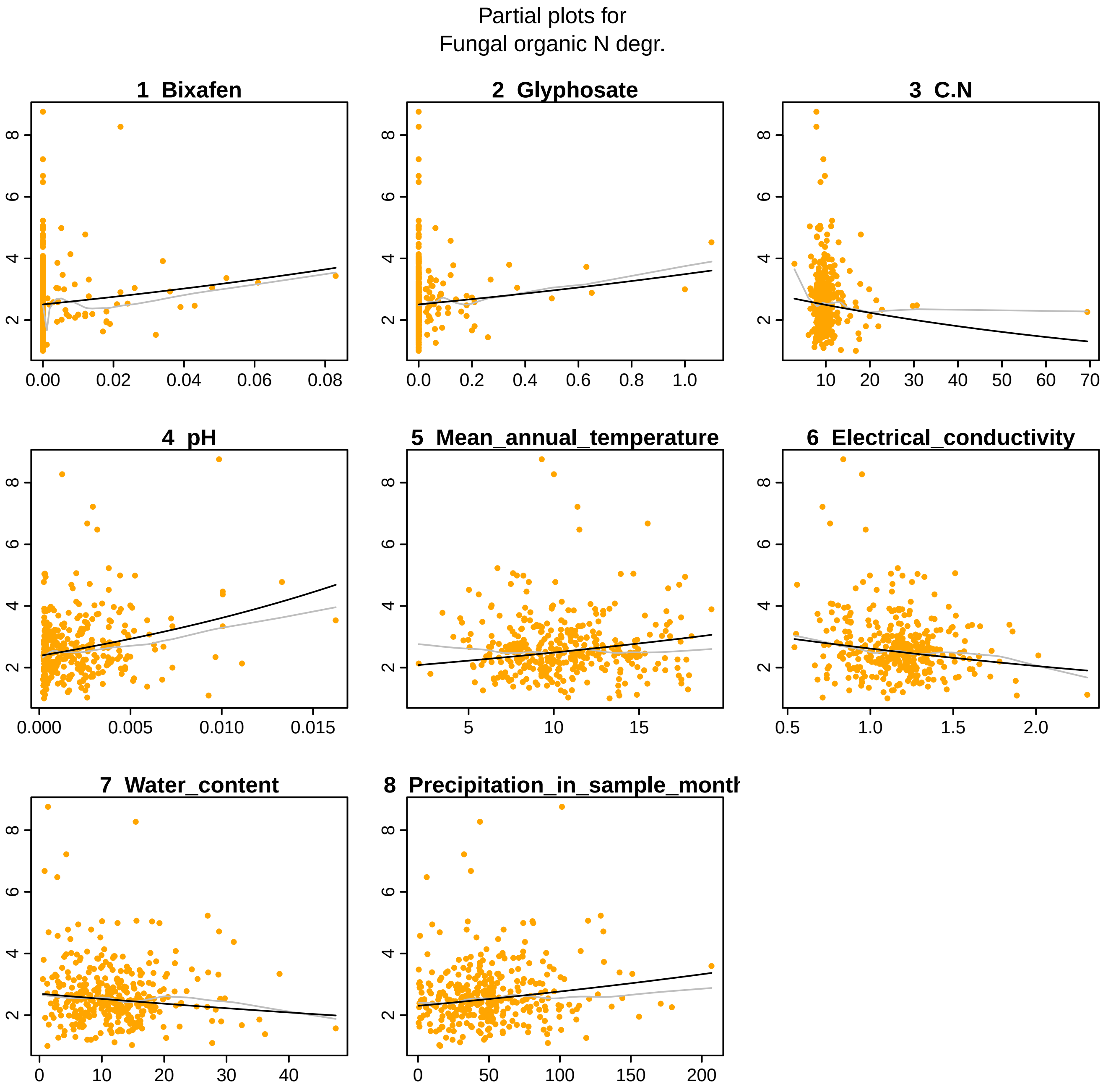
Partial plots for
Fungal organic N degr.
1 Bixafen
2 Glyphosate
3 C.N
4 pH
5 Mean_annual_temperature
6 Electrical_conductivity
7 Water_content
8 Precipitation_in_sample_month
0.00
0.02
0.04
0.06
0.08
0.0
0.2
0.4
0.6
0.8
1.0
10
20
30
40
50
60
70
0.000
0.005
0.010
0.015
5
10
15
0.5
1.0
1.5
2.0
0
10
20
30
40
0
50
100
150
200
2
4
6
8

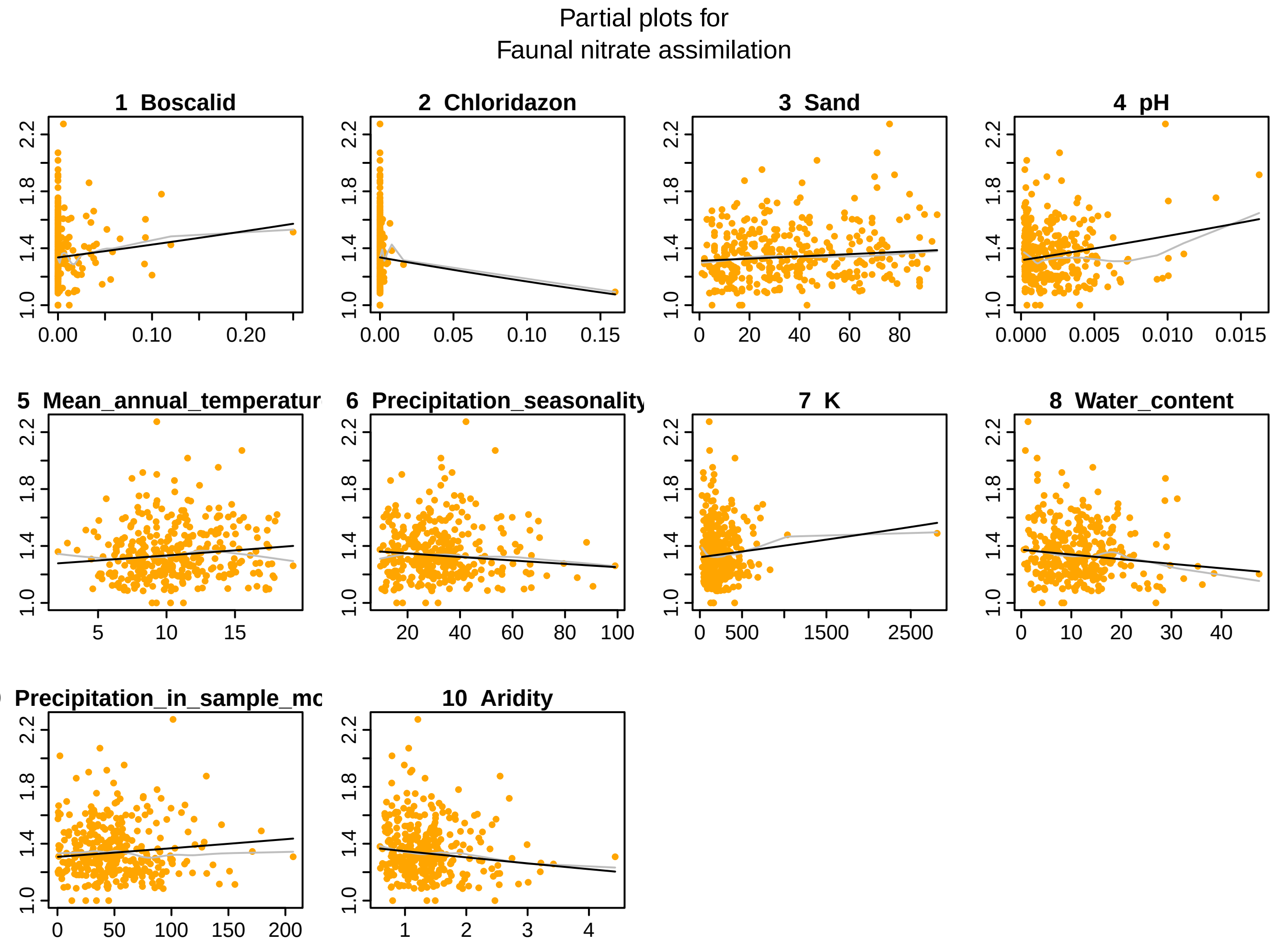
Partial plots for
Faunal nitrate assimilation
1 Boscalid
2 Chloridazon
3 Sand
4 pH
5 Mean_annual_temperatur
6 Precipitation_seasonality
7 K
8 Water_content
Precipitation_in_sample_mo
10 Aridity
1.0
1.4
1.8
2.2
0.00
0.10
0.20
0.05
0.15
0
20
40
60
80
0.000
0.005
0.010
0.015
5
10
15
100
500
1500
2500
30
50
150
200
1
2
3
4

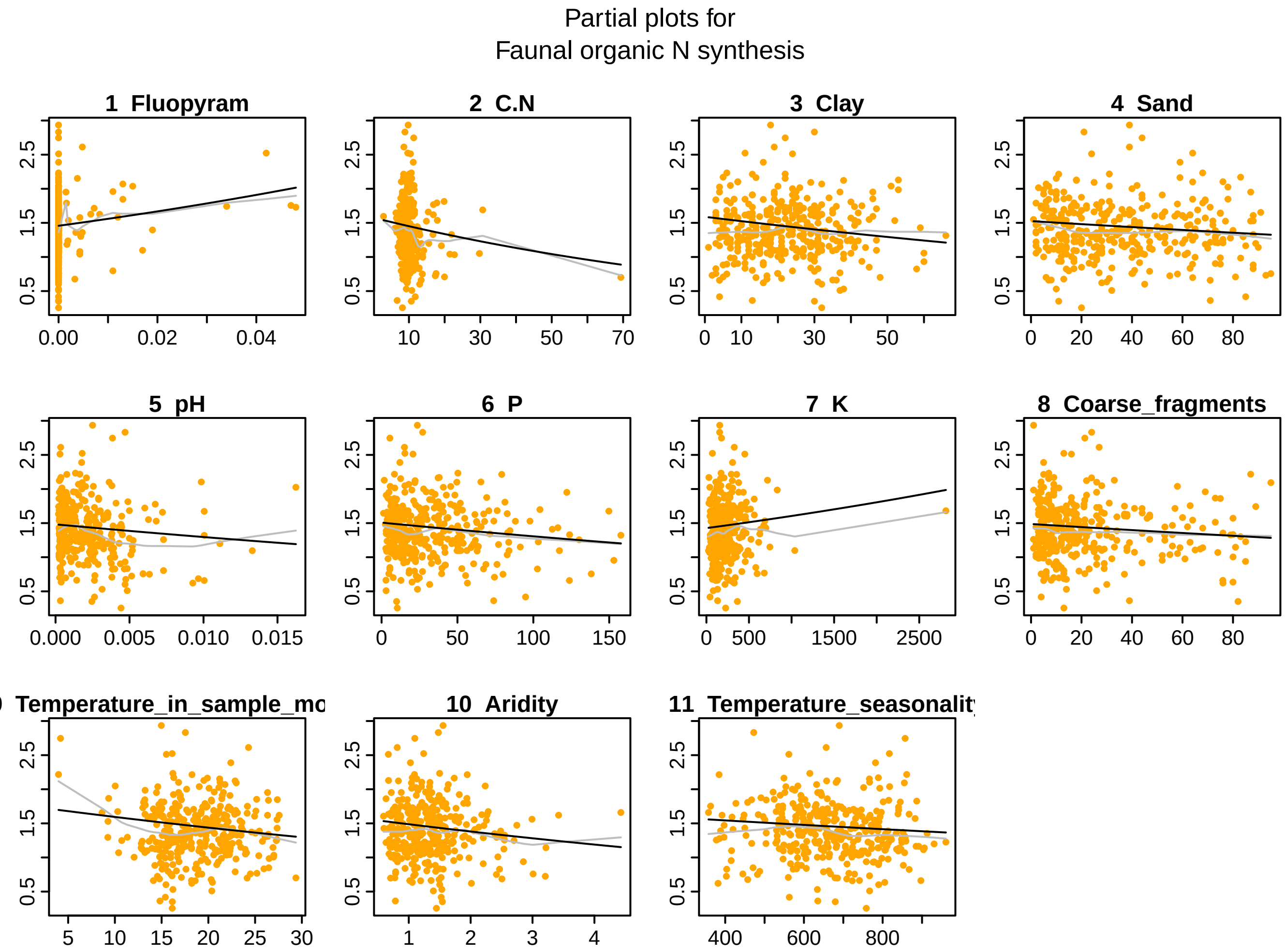
Partial plots for
Faunal organic N synthesis
1 Fluopyram
2 C.N
3 Clay
4 Sand
5 pH
6 P
7 K
8 Coarse_fragments
Temperature_in_sample_mo
10 Aridity
11 Temperature_seasonalit
0.5
1.5
2.5
0.00
0.02
0.04
10
30
50
70
0
20
40
60
80
0.000
0.005
0.010
0.015
100
150
500
1500
2500
5
15
25
1
2
3
4
400
600
800

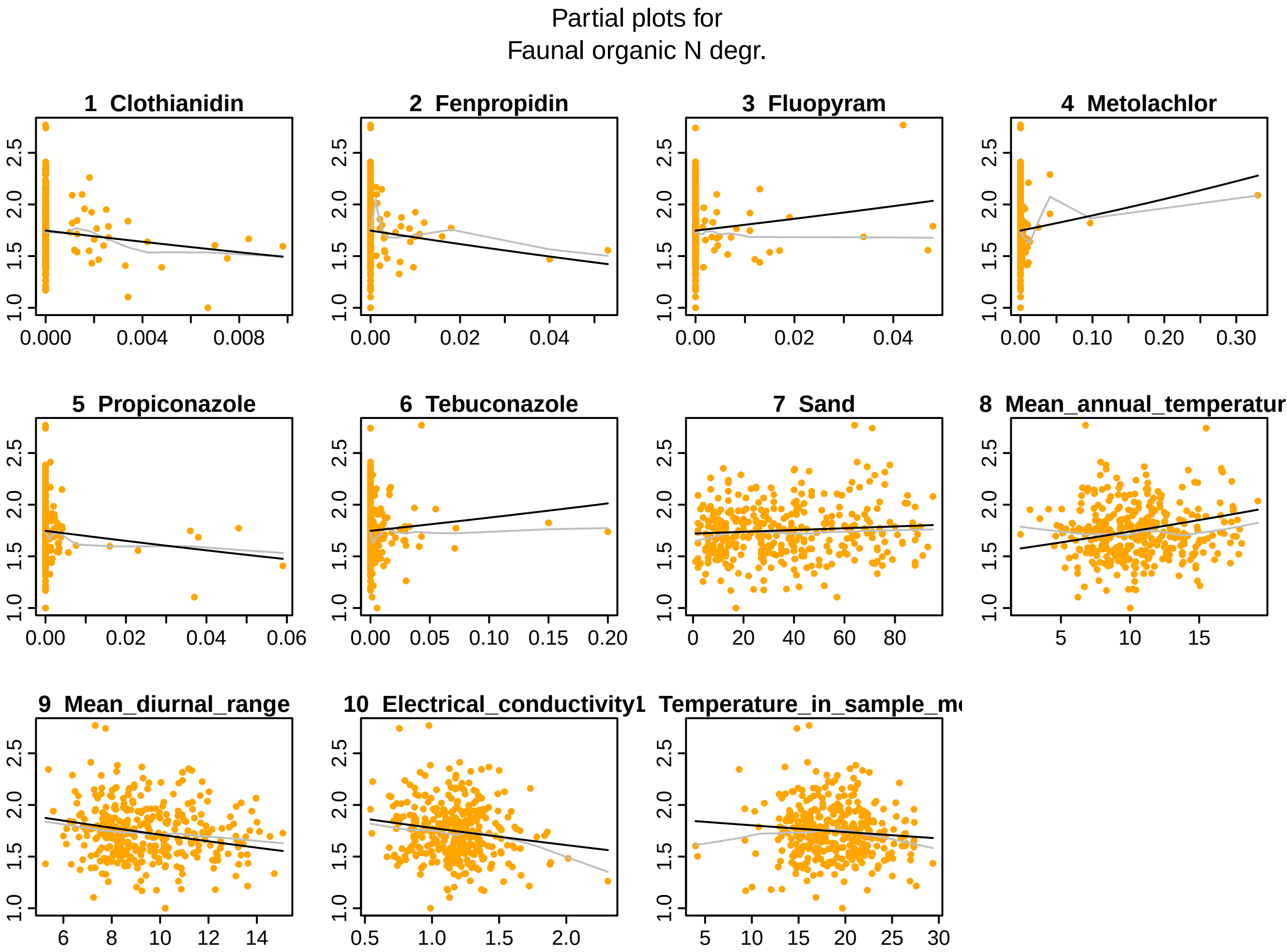
Partial plots for
Faunal organic N degr.
1 Clothianidin
2 Fenpropidin
3 Fluopyram
4 Metolachlor
5 Propiconazole
6 Tebuconazole
7 Sand
8 Mean_annual_temperatur
9 Mean_diurnal_range
10 Electrical_conductivity1
Temperature_in_sample_mo
1.0 1.5 2.0 2.5
0.000 0.004 0.008
0.00 0.02 0.04
0.00 0.10 0.20 0.30
0.00 0.02 0.04 0.06
0.00 0.05 0.10 0.15 0.20
0 20 40 60 80
5 10 15
6 8 10 12 14
0.5 1.0 1.5 2.0
5 10 15 20 25 30

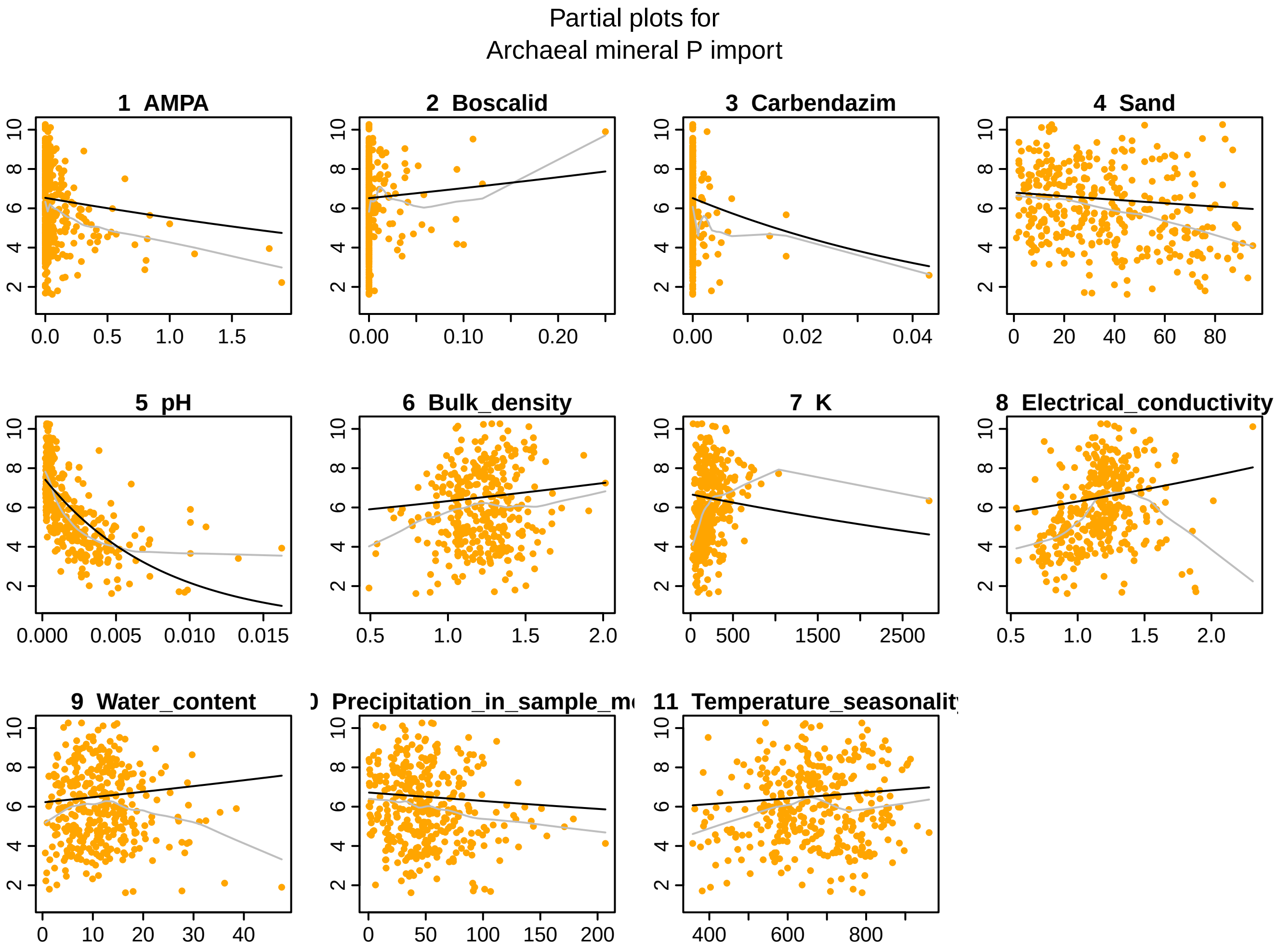
Partial plots for
Archaeal mineral P import
1 AMPA
2 Boscalid
3 Carbendazim
4 Sand
5 pH
6 Bulk_density
7 K
8 Electrical_conductivity
9 Water_content
Precipitation_in_sample_m
11 Temperature_seasonalit

Partial plots for
Archaeal organic P degr.

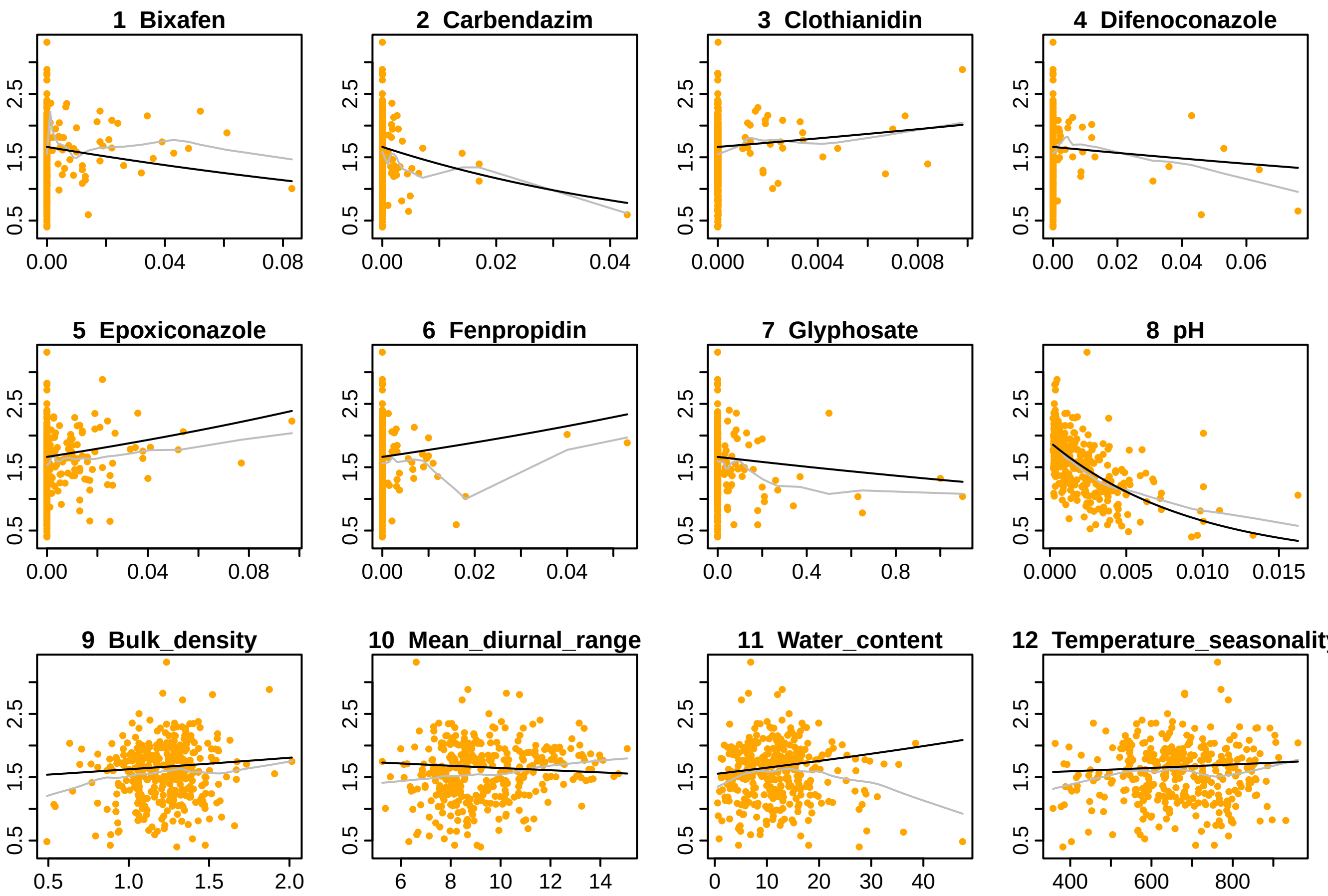

Partial plots for
Bacterial mineral P import

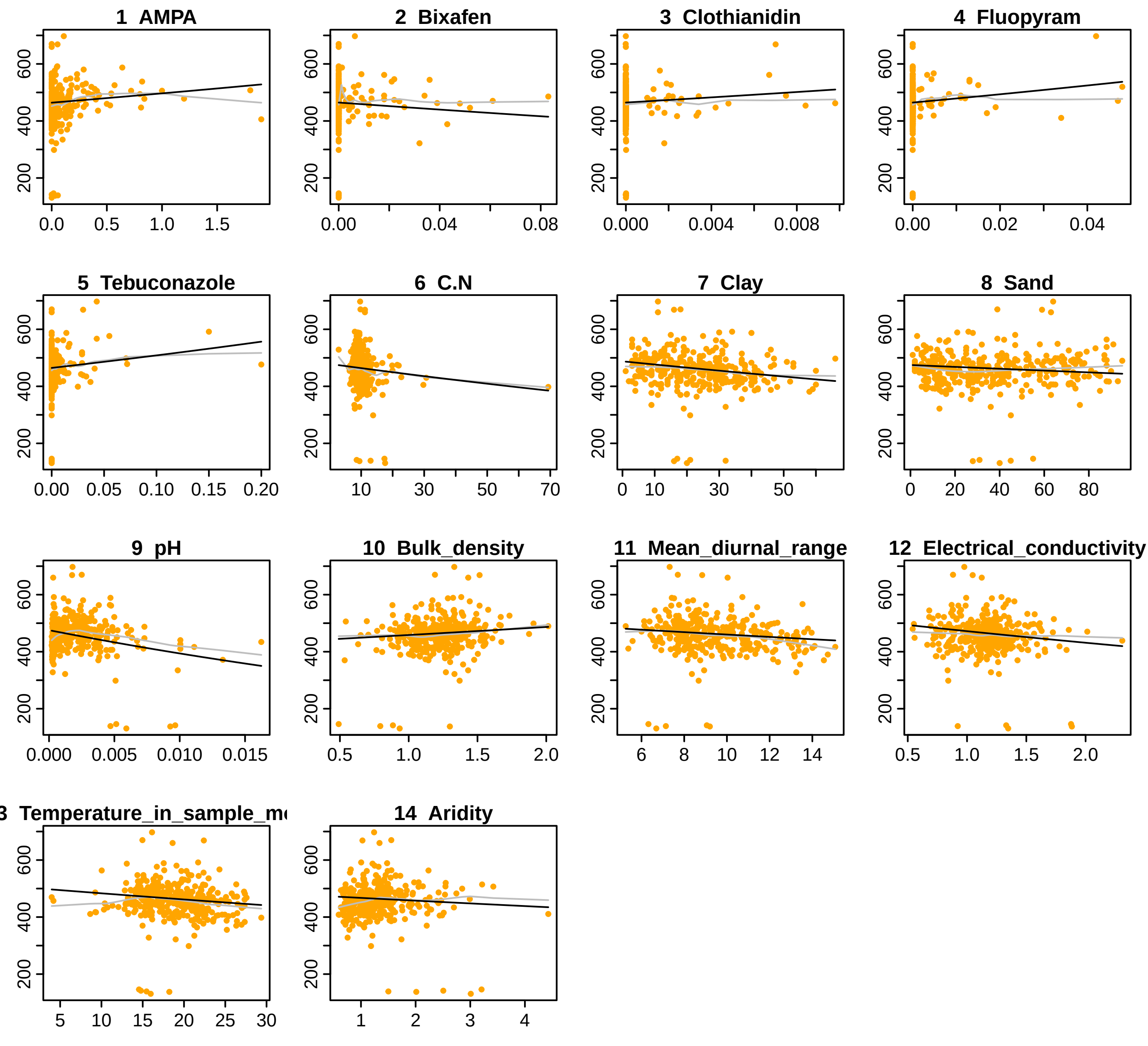

Partial plots for
Bacterial organic P degr.

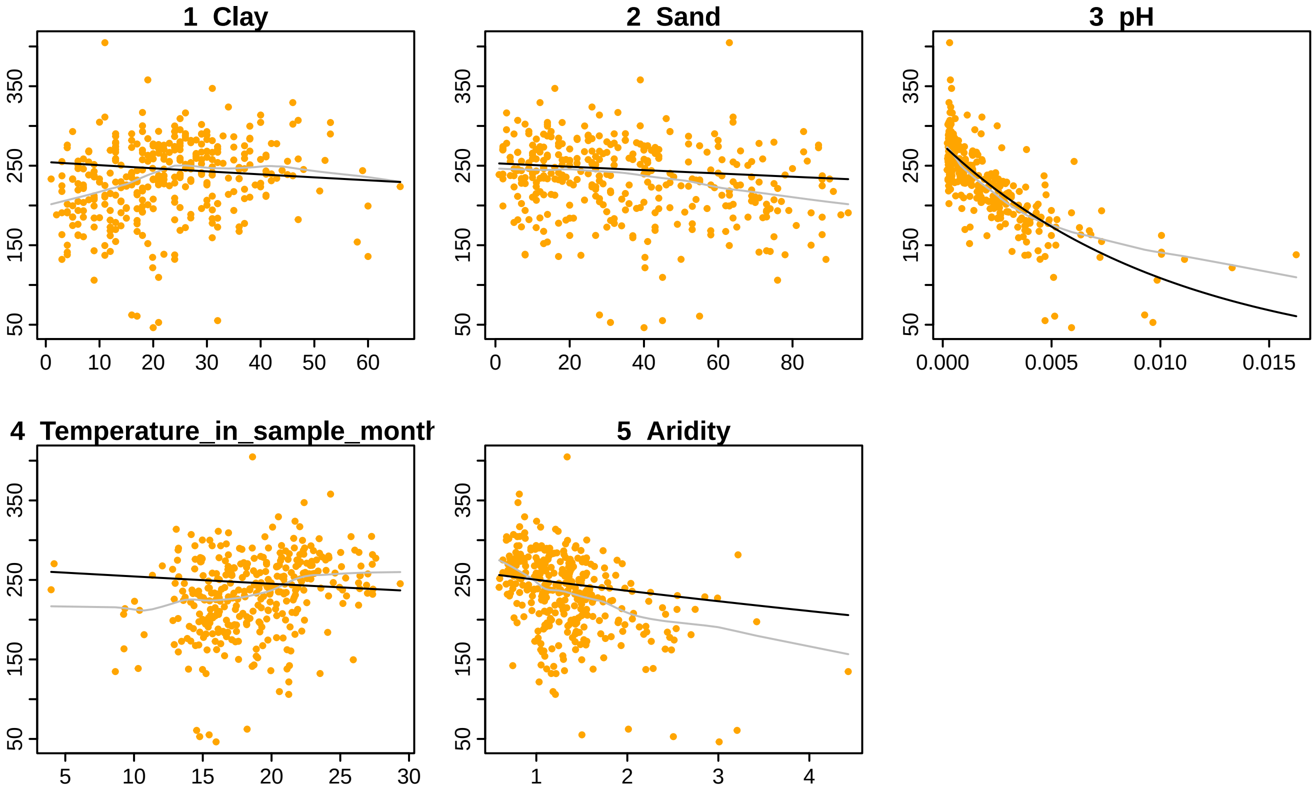

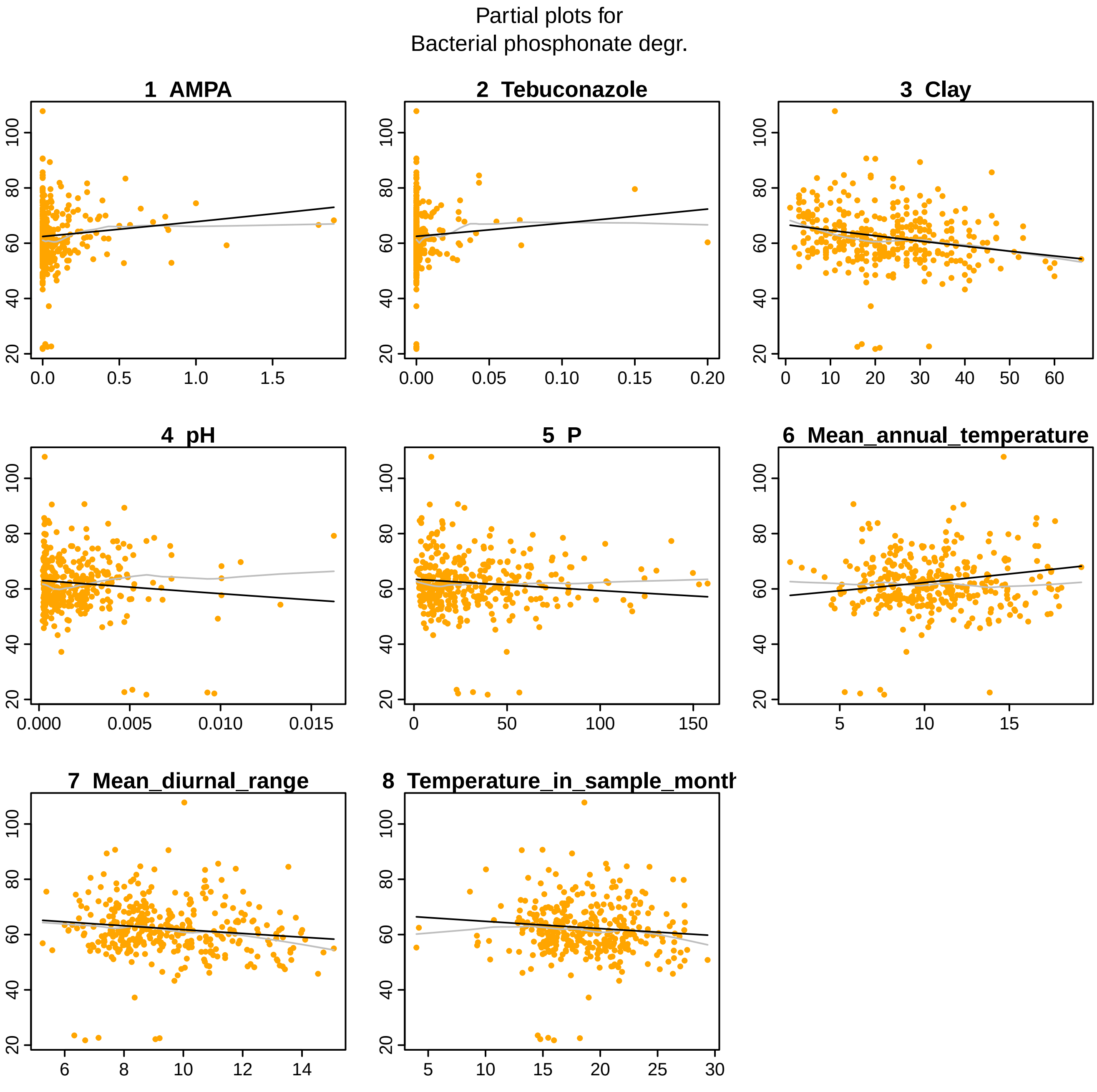
Partial plots for
Bacterial phosphonate degr.
1 AMPA
2 Tebuconazole
3 Clay
4 pH
5 P
6 Mean_annual_temperature
7 Mean_diurnal_range
8 Temperature_in_sample_montl
20
40
60
80
100
0.0
0.5
1.0
1.5
0.00
0.05
0.10
0.15
0.20
0
10
20
30
40
50
60
0.000
0.005
0.010
0.015
0
50
100
150
5
10
15
6
8
10
12
14
5
10
15
20
25
30

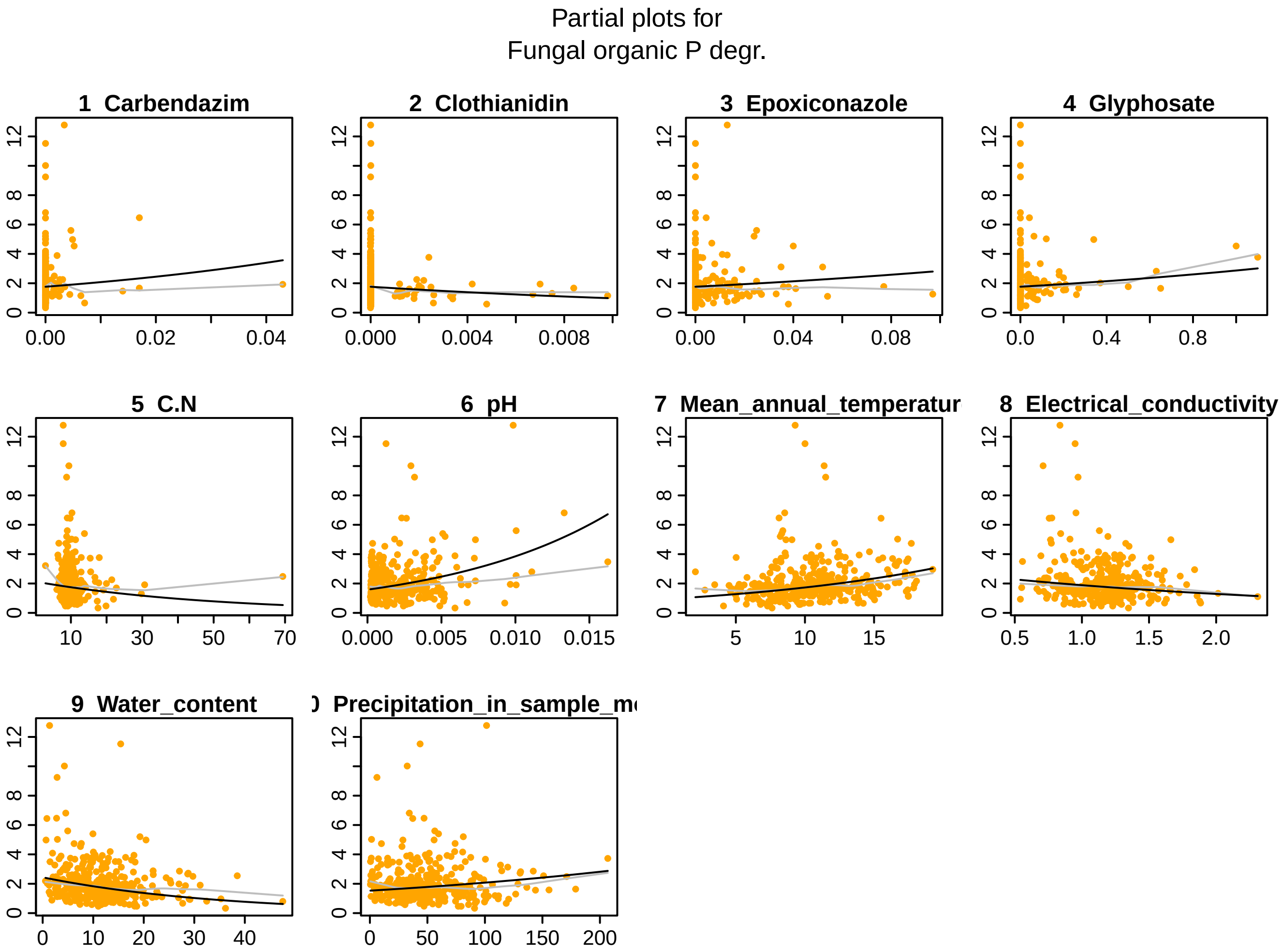

Partial plots for
Fungal organic P degr.
1 Carbendazim
2 Clothianidin
3 Epoxiconazole
4 Glyphosate
5 C.N
6 pH
7 Mean_annual_temperatur
8 Electrical_conductivity
9 Water_content
Precipitation_in_sample_m
